# CRISPR base editor screening identifies spectrum of *MEN1* mutations impacting menin inhibitors in clinical trials

**DOI:** 10.1038/s41467-026-72685-1

**Published:** 2026-05-09

**Authors:** Wallace Bourgeois, Hannah E. Rice, Daniela V. Wenge, Florian Perner, Hong Yue, Brandon D. Regalado, George Wan, Jan C. Schroeder, Alba Sommerschield, Charlie Hatton, Shivendra Singh, Sweta Singh, Shipra Bijpuria, Brian M. McKeever, William H. Miller, Jordan F. Safer, Sumaiya Iqbal, Jennifer A. Perry, Eric S. Fischer, John G. Doench, Gerard M. McGeehan, Jevon A. Cutler, Scott A. Armstrong

**Affiliations:** 1 Department of Pediatric Oncology, Dana-Farber Cancer Institute; Division of Hematology/Oncology, Boston Children’s Hospital; Harvard Medical School, Boston, MA USA; 2 Department of Hematology, Hemostasis, Oncology and Stem Cell Transplantation, Hannover Medical School (MHH), Hannover, Germany; 3 Department of Biological Chemistry and Molecular Pharmacology, Harvard Medical School, Boston, MA USA; 4 Department of Cancer Biology, Dana-Farber Cancer Institute, Boston, MA USA; 5 Syngene International Limited, Bangalore, India; 6 Brian M. McKeever, LLC, Lake Ronkonkoma, NY USA; 7 MedChem Innovations, LLC, Collegeville, PA USA; 8 Ladders To Cures Accelerator, Broad Institute of MIT and Harvard, Cambridge, MA USA; 9 Genetic Perturbation Platform, Broad Institute of MIT and Harvard, Cambridge, MA USA; 10 Syndax Pharmaceuticals, Inc, Waltham, MA USA; 11 Departments of Pediatrics and Cell, Developmental and Cancer Biology, Knight Cancer Institute, Oregon Health and Science University (OHSU), Portland, OR USA

**Keywords:** Cancer therapeutic resistance, Acute myeloid leukaemia, CRISPR-Cas9 genome editing

## Abstract

Menin inhibitors have entered clinical trials for *histone lysine methyltransferase 2 A (KMT2A)*-rearranged and *nucleophosmin 1 (NPM1)*-mutant acute leukemias and are demonstrating promising activity. CRISPR base editor screening previously predicted several *MEN1* (menin) mutations that have arisen in patients receiving SNDX-5613 and confer resistance. The extent to which *MEN1* mutations will impact each menin inhibitor is mostly unknown. Here we show that CRISPR base editor screens can be leveraged to profile the *MEN1* mutations that may impact five different menin inhibitors in clinical trials. We identify shared (M327I/V/T, G331D) and inhibitor-specific (C334R, E368K/V, V372A) resistance mutations. Co-crystal structures of menin bound to each menin inhibitor suggest resistance mechanisms related to how each inhibitor engages the KMT2A binding pocket of menin. Orthogonal in vitro and in vivo *MEN1* mutation generation under therapeutic pressure suggest the *MEN1* mutations identified with CRISPR base editor screening are likely to arise and impact all menin inhibitors.

## Abstract (web summary)

*KMT2A*-rearranged and *NPM1*-mutant acute leukemias treated with menin inhibitors frequently acquire resistance due to *MEN1* mutations. Here, the authors use CRISPR base-editor screens to predict shared and drug-specific *MEN1* resistance mutations across five clinical menin inhibitors.

## Introduction

Acute leukemias with *histone lysine methyltransferase 2 A* (*KMT2A* or *MLL1*) gene rearrangements (*KMT2A*-r) and *nucleophosmin 1* mutations (*NPM1c*) are dependent on the menin-KMT2A protein-protein interaction for leukemogenesis and leukemia maintenance^1,2^. The first small molecule inhibitors of the menin-KMT2A interaction were described in 2012^3^. Pre-clinical work has demonstrated that menin inhibitors induce displacement of menin from chromatin globally and KMT2A from select loci, such as *MEIS1*, followed by differentiation and apoptosis of leukemia cells in vitro and in vivo^4–7^. Substantial drug development has led to 8 menin inhibitors entering clinical trials since 2019^8^. The furthest in clinical development is the reversible, competitive inhibitor SNDX-5613 (revumenib, Syndax Pharmaceuticals). The AUGMENT-101 Phase I/II trial was an open-label, dose escalation study that administered SNDX-5613 monotherapy to patients with heavily pre-treated, relapsed or refractory *KMT2A*-r and *NPM1c* acute leukemias. This study demonstrated a complete remission rate (CR) or complete remission rate with partial hematologic recovery (CRh) of 22.8% for *KMT2A*-r acute leukemias and 23.4% for *NPM1c* AML^9–11^. SNDX-5613 now has U.S. Food and Drug Administration approval for relapsed or refractory *KMT2A*-r acute leukemia and *NPM1c* AML, and KO-539 (ziftomenib, Kura Oncology) is approved for relapsed or refractory *NPM1c* AML^12–14^.

On the Phase I portion of the AUGMENT-101 study, several patients initially responded and then progressed on therapy. Targeted next generation sequencing panels revealed acquisition of *MEN1* mutations at the time of progression^15^. Droplet digital PCR for select *MEN1* mutations (M327V, M327I, G331D, G331R, and T349M) in patients that received at least two cycles of therapy revealed *MEN1* mutations in 38.7% of patients, albeit some at very low allelic frequencies. Mechanistic studies revealed that *MEN1* mutations alter the KMT2A binding pocket on menin, resulting in decreased affinity of menin inhibitor binding without substantially perturbing the menin-KMT2A interaction, thereby allowing the oncogenic activity of the menin-KMT2A interaction to remain intact^15^. The KOMET-001 study, a Phase I/II open-label, dose escalation study with KO-539 also found a *MEN1* M327I mutation in one patient, suggesting *MEN1* mutations may affect the entire class of menin inhibitors^16^.

As menin inhibitors progress in clinical trials, it is mostly unknown to what extent *MEN1* mutations will impact each menin inhibitor and whether individual *MEN1* mutations will attenuate the activity of a subset or all menin inhibitors. In the initial work characterizing *MEN1* mutations, a *MEN1*-focused CRISPR base editor screen utilizing a pre-clinical analogue of SNDX-5613 predicted 3 of the 4 amino acid residues (S160, G331, T349) that were subsequently found to be recurrently mutated in patients on the AUGMENT-101 trial^15^. The prior screening approach utilized base editors capable of only C > T nucleotide transitions and SpCas9 nickases that required an NGG protospacer adjacent motif (PAM) site. SpCas9 nickases fused to deaminases capable of either C > T or A > G editing and that allow a more permissive NG PAM site have recently been developed and benchmarked^17^, allowing for the design of a *MEN1* sgRNA library with ~4x more assayable menin amino acid residues. Thus, we hypothesized that we could leverage advances in CRISPR base editing technology to characterize the profile of *MEN1* mutations likely to affect each menin inhibitor. Our screening approach revealed shared and unique profiles of deleterious *MEN1* mutations for each menin inhibitor, with the co-crystal structure of each menin inhibitor bound to menin suggesting a mechanism of action for most *MEN1* mutations identified. Orthogonal biological validation using in vitro and in vivo models suggests the *MEN1* mutations we uncovered and validated are likely to arise in patients receiving menin inhibitors.

## Results

### Menin inhibitors have similar activity in *MEN1* wild-type cell lines

**Fig. 1:**
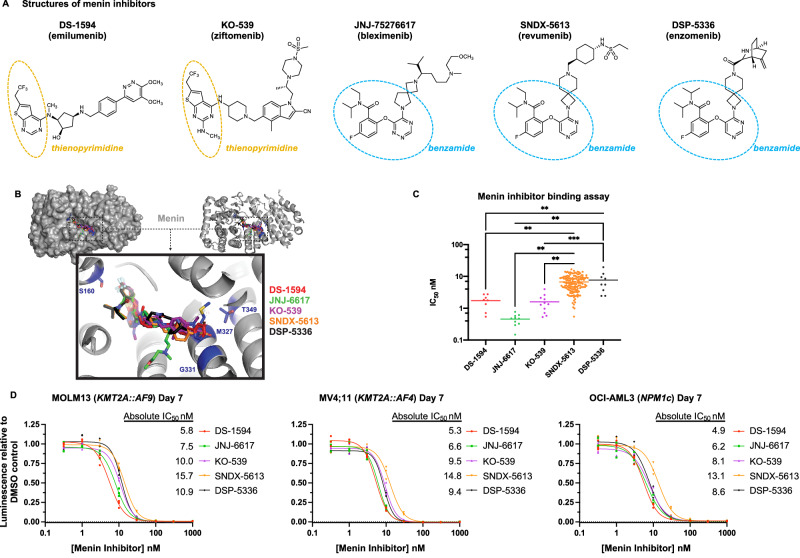
Menin inhibitors have similar activity in *MEN1* wild-type cell lines. **A** Chemical structures of menin inhibitor chemotypes currently in clinical development and used in this study. Key substructures are highlighted by colored ellipses. **B** Co-crystal structure of each menin inhibitor from **A** bound to menin. Amino acid residues where *MEN1* mutations have arisen in patients receiving SNDX-5613 are labeled in blue. **C** TR-FRET competitive binding assay measuring displacement of FITC-conjugated KMT2A-(4-43) peptide from wild-type menin by menin inhibitors. Each point represents an IC_50_ value derived from an 8-point dose-response curve from a single biological experiment (each performed with technical duplicates). The following number of biological replicates were tested: DS-1594 (*n* = 8), JNJ-6617 (*n* = 10), KO-539 (*n* = 11), SNDX-5613 (*n* = 155), DSP-5336 (*n* = 9). Statistical analysis was performed by ANOVA testing IC_50_ values with Tukey’s multiple comparisons test. Horizontal line displays median value. *p < 0.05, **p < 0.01, ***p < 0.001, ****p < 0.0001, only significant comparisons shown. Exact p-values are provided in the source data. **D** Viability assays assessed at Day 7 by CellTiter-Glo. Curves shown from one biological replicate with *n* = 3 technical replicates. Absolute IC_50_ values averaged from two biological replicates. Source data contains 95% confidence intervals of each Absolute IC_50_ as well as ANOVA statistical testing for differences between groups.

Prior to performing genetic screens, we sought to compare the activity of 5 reversible, non-covalent menin inhibitors that had entered clinical trials: DS-1594 (emilumenib, Daiichi Sankyo), JNJ-75276617 (bleximenib, Janssen Pharmaceuticals, referred to as JNJ-6617 hereafter), KO-539 (ziftomenib, Kura Oncology), SNDX-5613 (revumenib, Syndax Pharmaceuticals), and DSP-5336 (enzomenib, Sumitomo Pharma). All menin inhibitors used in this study were prepared based on synthetic routes described in the patent applications for the respective compounds (Supplementary Data 1), with synthesis routes for DS-1594, JNJ-6617, and KO-539 subsequently published^6,16,18,19^. These menin inhibitors can be categorized into two chemotypes: the first, consisting of DS-1594 and KO-539, share a thienopyrimidine core; the second, consisting of JNJ-6617, SNDX-5613, and DSP-5336, all share an aryloxy benzamide core (Fig. 1A). Previously, we published the co-crystal structure of SNDX-5613 bound to menin, and the co-crystal structures of DS-1594, JNJ-6617, and KO-539 bound to menin have since been published by other groups^6,16,18^. We generated co-crystal structures for each compound bound to menin and confirmed that each compound binds the KMT2A binding pocket on menin, though with different proximities to amino acid residues within the KMT2A binding pocket (Fig. 1B).

To determine the capacity of each compound to displace a KMT2A peptide from menin, we performed competitive binding assays. These inhibitors showed a strong binding affinity and inhibited the interaction in the single nanomolar range (Fig. 1C). We next performed viability assays with CellTiter-Glo in MOLM13 (*KMT2A::AF9*), MV4;11 (*KMT2A::AF4*), and OCI-AML3 (*NPM1c*) cell lines. We observed that these 5 menin inhibitors all display low nanomolar sensitivities after 7 days of treatment (Fig. 1D).

Thus, the site of binding to menin, the ability to displace KMT2A from menin, and effects on viability are similar for these competitive, reversible menin inhibitors. This suggests that any differences in the propensity to induce, or be affected by, *MEN1* mutations result from subtle differences in how each menin inhibitor binds in the KMT2A binding pocket of menin as opposed to merely the potency of menin-KMT2A inhibition.

### *MEN1* base editor screen with clinical menin inhibitors

**Fig. 2:**
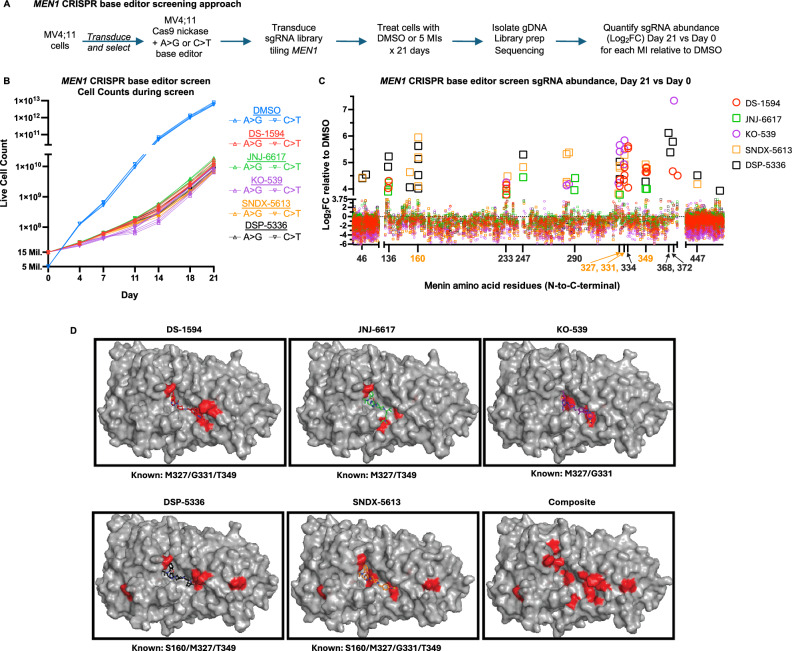
*MEN1* base editor screen with clinical menin inhibitors. **A**. Schematic of *MEN1*-focused CRISPR base editor screen. MI = menin inhibitor. Log_2_FC = Log_2_ fold change. **B**. Cell counts during CRISPR base editor screen. Upright triangles represent cell counts in A > G cell line, inverted triangles represent counts in the C > T cell line. Three technical replicates per condition are displayed individually on the graph. Live cells were counted and split every 3 to 4 days during the screen. **C**. sgRNA guide abundance comparing Day 21 to Day 0 from *MEN1*-focused CRISPR base editor screen, relative to DMSO control. Top portion of graph shows sgRNAs with Log_2_FC > 3.75. sgRNAs are aligned by the predicted menin amino acid that is targeted. Each data point represents the mean of three technical replicates. **D**. Co-crystal structure of menin bound to each menin inhibitor. Labeled in red are the amino acid residues that individual sgRNAs are predicted to target and that enriched >3.75 Log_2_FC in the screen. Known residues refer to sites where *MEN1* mutations have arisen previously in patients receiving SNDX-5613.

We hypothesized that *MEN1*-focused CRISPR base editor screens leveraging improved base editing technology would reveal key similarities and differences in the *MEN1* mutations that impact each compound. We first established two different MV4;11 cell lines constitutively overexpressing SpCas9 nickases fused to deaminase enzymes, one capable of making A > G edits and the other C > T edits, hereafter called MV4;11 A > G and MV4;11 C > T (Fig. 2A).^20,21^, We designed and cloned a guide RNA (sgRNA) library tiling the coding exons of *MEN1*, intergenic control guides, and positive-control guides targeting essential genes. We sought to utilize an equipotent concentration of each menin inhibitor during the screen. As such, in MV4;11 A > G cells we performed dose response curves with DS-1594, JNJ-6617, KO-539, SNDX-5613, and DSP-5336, confirming similar responses in parental MV4;11 cells (Supplementary Fig. 1A). We averaged the 75% growth inhibitory concentration at day 5 and the 97.5% growth inhibitory concentration at day 9 and used this as the concentration during the screen: 13.8 nM DS-1594, 17.3 nM JNJ-6617, 23.4 nM KO-539, 51.6 nM SNDX-5613, 25.2 nM DSP-5336. We then transduced our *MEN1*-targeting library into MV4;11 A > G and MV4;11 C > T cell lines. After 48 hours, the transduction efficiency was 15%, suggesting a low frequency of multiple guide infection of the same cell. Cells were selected with puromycin and expanded, and drug treatment was initiated 10 days after library transduction. During the screen, cells were treated in three technical replicates for each condition (DMSO and each of the 5 menin inhibitors). We maintained 2000x coverage of each sgRNA during the duration of the 21-day screen, during which time cells were split and drug replenished every 3-to-4 days. The screen was terminated after 21 days, at which point the cells exhibited resistance based on their accelerating growth towards the end of the screen (Fig. 2B, Supplementary Fig. 1B). Genomic DNA isolation, library preparation, and sequencing were then performed.

To assess which sgRNAs enriched during the screen and the corresponding domains of menin they targeted, we first calculated sgRNA abundance on day 21 compared to day 0 for each drug relative to DMSO control (Fig. 2C, Supplementary Fig. 1C, Supplementary Fig. 2A+B, Supplementary Data 2). Replicate concordance was high, with an average Pearson correlation coefficient of 0.90 across replicate pairs, and all pairwise correlations ≥ 0.79 (Supplementary Fig. 3A+B). Guide-level beta scores and associated statistics from MAGeCK MLE are reported in Supplementary Data 2. When evaluating highly enriching sgRNAs (Log_2_ fold change (FC) ≥ 3.75), there was substantial clustering of sgRNAs that targeted relatively few amino acid residues. Many of these were at sites where *MEN1* mutations have arisen with SNDX-5613, while others were at previously undescribed residues (E368 or V372). To connect this genetic data with structural data, we used the Genomics2Proteins portal to map the predicted amino acid substitutions that scored in our screens onto the co-crystal structures of each menin inhibitor bound to menin (Fig. 2D, Supplementary Table 1, Supplementary Data 3)^22^. Most of the amino acids predicted to confer resistance, based on sgRNA enrichment ≥3.75 Log_2_FC, occurred at residues whose wild-type side chains are located within the KMT2A binding pocket. The data from the screen suggests that *MEN1* mutations are most likely to be enriched at sites that impair menin inhibitor binding to menin.

### Single guide validation of *MEN1* base editor screen

To validate the results and quantify the effects of each guide that scored in the base editor screen, we identified 40 individual sgRNAs that enriched Log_2_FC ≥ 4.0 in either the A > G or C > T arms of the base editor screen for at least one drug condition relative to DMSO control. Of note, despite strong correlation between technical replicates in the screen (Supplementary Figs. 3A+B), only one positively selecting guide per drug condition reached statistical significance by MAGeCK (p-value < 0.05 and FDR < 0.05; Supplementary Data 2), highlighting the importance for single guide validation of enriching hits from the screen. We then cloned these 40 sgRNAs and 2 intergenic negative control sgRNAs into an RFP-expressing and puromycin resistant vector and transduced each sgRNA into both the MV4;11 A > G and C > T cell lines (Supplementary Fig. 4A). Thus, in total we produced 80 MV4;11 cell lines from 40 *MEN1*-targeting sgRNAs. Cells were selected with puromycin to produce purified cell lines with only genomic edits from a single sgRNA. To assess for drug resistance, we performed competition assays. We mixed a 20% population of cells transduced with an individual sgRNA (PE+ by flow) with 80% unedited cells and assessed for enrichment by flow cytometry (% PE + ) after treating cells for 9 days with the same drug condition of each menin inhibitor that was used for the base editor screens. From the 40 highly enriching sgRNAs from the base editor screen, 32 outcompeted the non-edited wild-type cells in either MV4;11 A > G or C > T cell lines (and 5 sgRNAs outcompeted in both the A > G and C > T cell lines) to a threshold of ≥ 3x enrichment ( > 60% *MEN1* mutant, <40% *MEN1* wild-type on day 9) with at least two menin inhibitors or ≥ 4.25x enrichment ( > 85% *MEN1* mutant, <15% *MEN1* wild-type on day 9) with one menin inhibitor (Supplementary Fig. 4B+C).

**Fig. 3:**
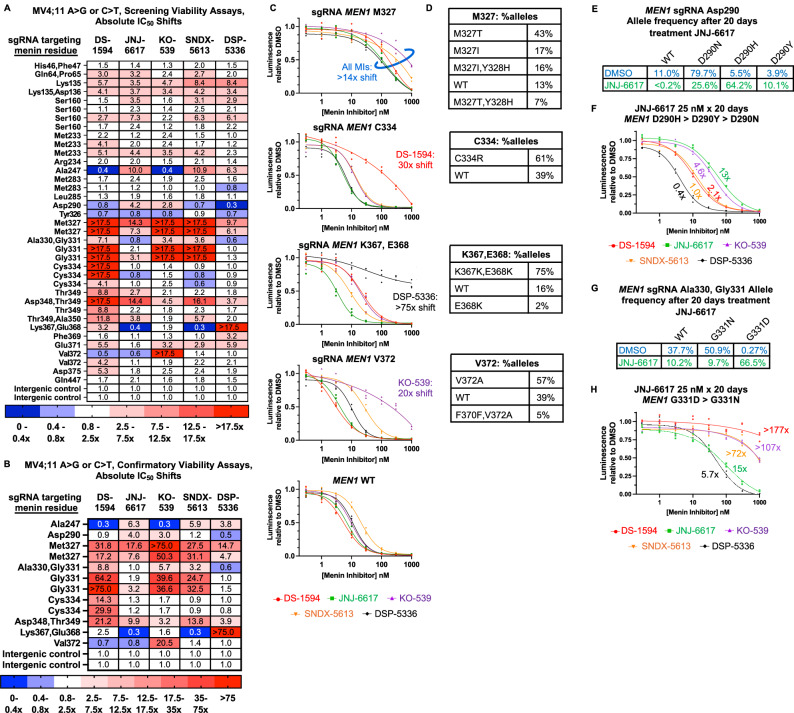
Single guide validation of *MEN1* base editor screen. **A**, **B** Heat map of absolute IC_50_ shifts in *MEN1* mutant versus wild-type (intergenic control) cell lines after 5-day viability (CellTiter-Glo) assays. Absolute IC_50_ shift is the ratio of the absolute IC_50_ in the mutant vs wild-type cell line with the same base editor (A > G vs. C > T) that were plated (Day 0) and read (Day 5) on the same day. Each row represents an MV4;11 *MEN1* mutant cell line generated by an sgRNA that scored in the base editor screen and validated in competition assays. The predicted edited residue(s) for each sgRNA are annotated. **A** Each screening viability assay performed with 5-point dose response curve (3 to 300 nM) and in three technical replicates. The absolute IC_50_ shift is displayed. **B** Confirmatory viability assays performed for each cell line in screening viability assays **A** in which the absolute IC_50_ was shifted ≥ 10x for ≥1 menin inhibitor or ≥7.5x for one menin inhibitor relative to another. Each viability assay performed with 8-point dose response curve (0.3 to 1000 nM), and in three technical replicates with at least two biological replicates (representative example shown), mean absolute IC_50_ shift displayed. **C** Select dose response curves from confirmatory viability assays in **B**. Each graph is a representative of two biological replicates each with three technical replicates. **D** For the cell lines in **C**, the contribution of alleles with ≥2% allelic frequency among all variants detected above a 0.2% calling threshold as determined by amplicon sequencing. **E**, **G** Allelic frequency from amplicon sequencing at Asp290 **E** and Gly331 **G** following 20 days of DMSO versus JNJ-6617 25 nM treatment. **F**,**H** Viability assays following drug washout in cells treated with JNJ-6617 for 20 days. Viability assays performed with 8-point dose response curve, ranging from 0.3 nM to 1000 nM, and in at least technical duplicates with three biological replicates (representative experiment shown), mean absolute IC_50_ shift displayed (see Supplementary Fig. 8D, G). Source data for Fig. 3 contains Absolute IC_50_ values for biological replicates with 95% confidence intervals, as well as ANOVA statistical testing.

For these 37 cell lines that exhibited resistance in competition assays, in addition to the two cell lines with control sgRNAs targeting intergenic control regions, we performed screening viability assays by CellTiter-Glo with 5-dose drug titrations (3 nM to 300 nM). We calculated the absolute IC_50_ for each drug in each cell line (Supplementary Fig. 5A). The absolute IC_50_ shift was calculated by comparing the absolute IC_50_ in the *MEN1* mutant cell line to that in a MV4;11 A > G or C > T *MEN1* wild-type control cell line (sgRNA targeting an intergenic control) that was plated, treated with drug, and read out on the same days (Fig. 3A). 12 different *MEN1* mutant cell lines, inclusive of 8 different amino acid residues, shifted the absolute IC_50_ at least 10-fold relative to control or 7.5-fold relative to another menin inhibitor. For these 12 cell lines, we then performed confirmatory viability assays with 8-dose drug titrations (0.3 nM to 1000 nM, Fig. 3B+C, Supplementary Fig. 5B). These *MEN1* mutations affected known (M327, G331, T349) and previously uncharacterized amino acid residues (A247, D290, C334, E368, V372). To confirm the actual *MEN1* mutation induced in these cell lines, we first performed Sanger sequencing immediately after establishing the cell line and prior to the administration of menin inhibitor (Supplementary Fig. 6+7). Importantly, each cell line displayed polyclonal *MEN1* mutations, ranging from low-allele frequency edits to homozygous edits. Bystander mutations affecting nearby amino acids also occurred with some guides. For the 12 sgRNAs in confirmatory viability assays, when the Sanger sequencing chromatogram did not reveal a near homozygous edit without bystander mutations, we performed amplicon sequencing to characterize the allelic heterogeneity induced in each cell line (Fig. 3D, Supplementary Fig. 7). The *MEN1* edit by the sgRNA predominantly affected the predicted amino acid residue in each instance.

These viability assays demonstrate that each menin inhibitor was differentially affected by *MEN1* mutations. C334R mutations are specific for DS-1594, with the absolute IC_50_ shifted >14-fold for DS-1594 but not greater than 1.7-fold for any other compound. E368K mutations are specific for DSP-5336 with an absolute IC_50_ shift >75-fold, while DS-1594 was shifted 2.5-fold, KO-539 was shifted 1.6-fold, and JNJ-6617 and SNDX-5613 had 0.3-fold lower absolute IC_50_s. V372A mutations were specific to KO-539, with the absolute IC_50_ shifted >20-fold for KO-539 while the other menin inhibitors were shifted <1.4-fold, with DS-1594 and JNJ-6617 showing 0.7-fold and 0.8-fold lower absolute IC_50_s. This increased sensitivity, i.e., lower absolute IC_50_s, was also seen in competition assays: *MEN1* E368K cells depleted faster than wild-type with JNJ-6617 and SNDX-5613, and *MEN1* V372A cells depleted faster with DS-1594 and JNJ-6617 (Supplementary Fig. 4B, C). Consistent with this, *MEIS1* expression was more strongly reduced in E368K mutant cells than wild-type cells after SNDX-5613 treatment (10 or 50 nM) and in V372A mutant cells after JNJ-6617 treatment (10 nM) (Supplementary Fig. 8A). Conversely, *MEN1* mutations at M327 appear capable of conferring pan-class resistance, which was particularly demonstrated in the cell line that made predominantly M327T edits (absolute IC_50_ shifted at least 14-fold for each menin inhibitor). Nevertheless, M327 mutations affect DS-1594, KO-539, and SNDX-5613 to a greater degree than JNJ-6617 and DSP-5336.

We hypothesized that *MEN1* residues with modest absolute IC_50_ shifts in viability assays might have greater impact with alternate amino acid substitutions. To test this, we exposed MV4;11 C > T cells (*MEN1* wild-type, sgRNA D290-targeting, or sgRNA A330/G331-targeting) to DMSO, JNJ-6617 (25 nM), or KO-539 (25 nM) for 20 days (Supplementary Fig. 8B, E). Without drug, these guides primarily generated D290N (79.7% of alleles) and G331N (50.9% of alleles) mutations (Fig. 3E, G, Supplementary Fig. 8C, F). Both *MEN1*-mutant lines continued to grow under menin inhibitor pressure, and amplicon sequencing after 20 days showed that JNJ-6617 selected for D290H (5.5 → 64.2%) > D290Y (3.9 →10.1%) > D290N (79.7 → 25.6%) and G331D (0.27 → 66.5%) > G331N (50.9 → 9.7%), versus KO-539 which selected for D290Y > D290H (3.9 → 35.1% and 5.5 → 21.3%) and G331N (50.9 → 89.1%). After drug washout, D290H cells showed a > 13-fold absolute IC_50_ shift for JNJ-6617, with no other inhibitor shifting >5-fold (Fig. 3F, Supplementary Fig. 8D). G331D conferred pan-resistance, shifting DS-1594 > 177x, SNDX-5613 > 72x, KO-539 > 107x, JNJ-6617 15x, and DSP-5336 5.7x (Fig. 3H). In contrast, G331N largely retained sensitivity to JNJ-6617 (1.3x shift), showed increased sensitivity to DSP-5336 (0.5x), and strong resistance to DS-1594 (65x), KO-539 (37x), and SNDX-5613 (23x) (Supplementary Fig. 8G). These results underscore that the particular amino acid substitution at a given residue can critically influence the magnitude of menin inhibitor resistance.

### Biochemical and structural insights into *MEN1* mutation effects

**Fig. 4:**
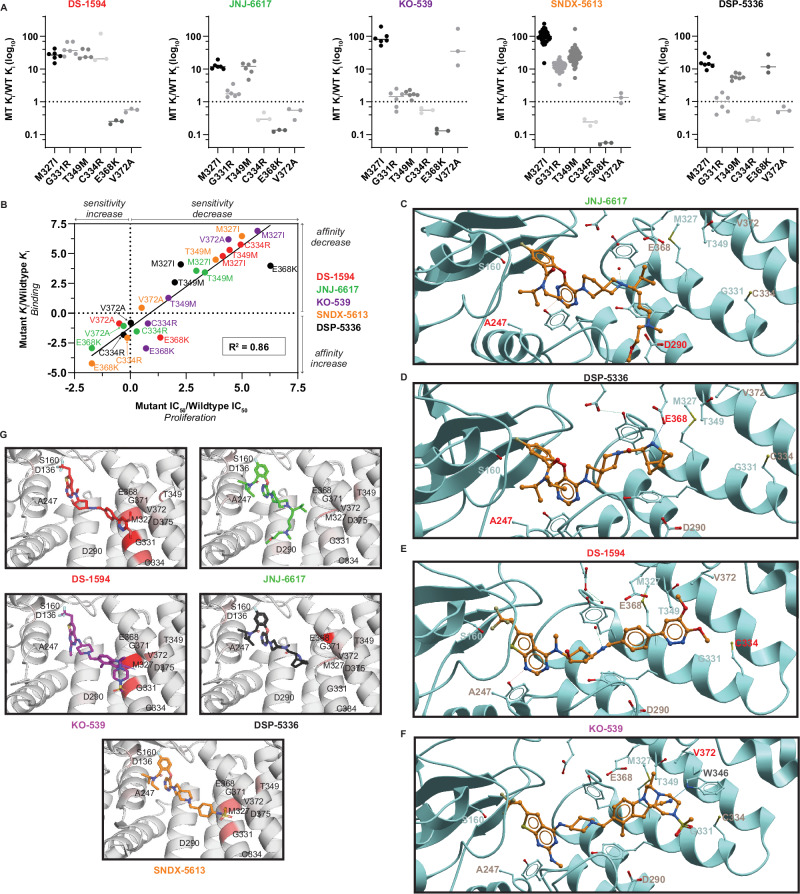
Biochemical and structural insights into *MEN1* mutation effects. **A** Dot plots showing ratios of mutant (MT) *K*_i_ to wild-type (WT) *K*_i_ values derived from KMT2A/B peptide-menin TR-FRET competitive displacement binding assays for each indicated menin inhibitor and mutant menin protein (M327I, G331R, T349M, C334R, E368K and V372A). Each point represents an individual IC_50_ determination used to calculate *K*_i_. Each point represents an IC_50_ value derived from an 8-point dose-response curve with technical duplicates, normalized to WT (WT values are the same as in Main Fig. 1C). For M327I, G331R, and T349M, data include *n* = 6 biological replicates for DS-1594, JNJ-6617, KO-539, and DSP-5336, except *n* = 7 for KO-539 and DSP-5336 for M327I and T349M. For SNDX-5613, biological replicates include *n* = 85 for M327I and T349M and *n* = 87 for G331R. For C334R, E368K, and V372A, *n* = 3 biological replicates were performed for each drug. **B** XY-plot comparing log_2_ fold changes in binding affinity (Kᵢ) measured by TR-FRET assays (Fig. 4A) with log₂ fold changes in absolute IC_50_ values from viability assays (Fig. 3B). Each data point represents the mutant-to-wild-type ratio. A linear regression line is shown, with the corresponding R^2^ value indicated. **C-F** Co-crystal structure of menin (gray ribbon) bound to either, **C** JNJ-6617 (PDB ID#: 9WKU), **D** DSP-5336 (PDB ID#: 9WN9), **E** DS-1594 (PDB ID#: 9WKW), **F** KO-539 (PDB ID# 9WNI). Side chains of labelled residues are shown. Mutations at residues previously reported in patients receiving SNDX-5613 (M327, G331, S160, and T349) are labelled in cyan and W346 is additionally labeled in black in **F**. All newly discovered sites liable to resistance mutations are shown in light brown or, in the case where they confer resistance to the inhibitor pictured, in red. **G** Co-crystal structures of menin bound to each menin inhibitor. Residues D136, S160, A247, D290, M327, G331, C334, T349, E368, G371, V372, and D375 are colored red with intensity proportional to the shift in the absolute IC_50_ from viability assays (Fig. 3A, B) for each menin inhibitor. All other residues are colored in a uniform baseline grey.

Previously, we showed that *MEN1* mutations arising in patients receiving SNDX-5613 encode mutant menin proteins that have decreased affinity for menin inhibitors^15^. We hypothesized that *MEN1* mutations induced by sgRNAs in the CRISPR base editor screen would correlate with the affinity shift for menin inhibitors to mutant menin protein. To test this, we performed KMT2A/B peptide-menin TR-FRET displacement assays for all 5 inhibitors comparing wild-type menin proteins to menin mutant proteins found in patients receiving SNDX-5613^15^ (I327, R331, and M349) and unique resistance mutations from the base editor screen (R334, K368, and V372). The T349M and M327I substitutions caused a reduction of binding affinity for all compounds, though with varying affinity shifts (Fig. 4A, Supplementary Fig. 9A-C, Supplementary Tables 2-4, Supplementary Data 4+5). The T349M substitution shifted affinities for DS-1594 (30x), SNDX-5613 (28x), and JNJ-6617 (12x) to a greater extent than DSP-5336 (5.9x) and KO-539 (1.7x). The G331R substitution shifted affinities for DS-1594 (43x) and SNDX-5613 (13x) to a greater extent than JNJ-6617 (2.0x), KO-539 (1.4x), or DSP-5336 (1.0x). The M327I substitution produced the most profound effect on all menin inhibitors, reducing the binding affinity of SNDX-5613 (102x), KO-539 (96x), DS-1594 (27x), DSP-5336 (17x), and JNJ-6617 (13x). The three unique resistance mutations each had unique binding affinity shifts, with DS-1594 exhibiting a 53x shift for C334R, DSP-5336 exhibiting a 16x shift for E368K, and KO-539 exhibiting a 72x shift for V372A, despite no other MIs having a greater than 1.5x shift for these menin mutant proteins and many exhibiting shifts <1x. The shift in the menin inhibitor binding affinity was highly correlated with the absolute IC_50_ shift in individual cell lines with *MEN1* mutations introduced by sgRNAs in MV4;11 A > G and C > T cell lines (Fig. 4B).

Given the binding and viability assay data demonstrating that M327I conferred resistance to all five inhibitors, though with varying degrees of resistance, we co-crystallized the I327 mutant menin protein bound to each menin inhibitor to gain structural insights into these differential resistance effects (Supplementary Fig. 10A, Supplementary Table 1). Superimposing M327 and I327 ligand-bound structures revealed that the backbone of both mutant and wild-type menin protein remains largely unchanged with Root Mean Square Deviation (RMSD) values below 0.4 Å. The β-branching methyl substituent of I327 pointed directly toward the inhibitors in all co-crystal structures similar to what was observed with SNDX-5613 bound to I327^15^, however, adjustments in the relative positions of the inhibitors compared to wild-type varied. DS-1594, KO-539, and SNDX-5613 all bind in an extended conformation, reaching across the M327 position toward the distal end of the binding pocket, which results in more pronounced distortion of the binding pose seen in wild-type (Supplementary Fig. 10B-F). Neither JNJ-6617 nor DSP-5336 showed significant changes to their overall binding pose. Consistent with previous observations about JNJ-6617^6^, and now seen with DSP-5336, both bind in conformations that avoid M327, which likely explains why the I327 protrusion causes much less displacement and binding similar to wild-type. This structural analysis suggests that the magnitude of M327I resistance depends on how each inhibitor uniquely engages the binding pocket, with KO-539 and SNDX-5613 most affected, followed by DS-1594, and JNJ-6617 and DSP-5336 least affected, consistent with the hierarchy observed in our binding and cellular systems (Fig. 4B).

We next hypothesized that investigating these co-crystal structures could elucidate the mechanisms by which certain *MEN1* mutations confer resistance based on menin inhibitor chemotype. For example, mutations at S160 and an A247T mutation disproportionately affect JNJ-6617, SNDX-5613, and DSP-5336 while sparing DS-1594 and KO-539 (Fig. 3A, B). JNJ-6617, SNDX-5613, and DSP-5336 share a common aryloxy benzamide core (Fig. 1A) that positions each of these menin inhibitors in the same orientation deeper in the KMT2A binding pocket of menin. A247 sits directly below the benzamide isopropyl group and any branching of the mutant threonine residue will likely yield a steric interaction with the aryloxy benzamide core. Similarly, the branching of the benzamide and the edge of the aryl group on this scaffold will likely have a steric clash with any substitution at S160 (Fig. 4C–F).

We further hypothesized that investigating the co-crystal structures could suggest mechanisms by which some *MEN1* mutations produce mutations that are unique to individual menin inhibitors. In the validation of the CRISPR base editor screen, the three *MEN1* mutations that caused the most unique resistance were C334R mutations for DS-1594, V372A mutation for KO-539, and E368K mutation for DSP-5336 (Fig. 3A–D). For DSP-5336, the compound forms a direct ion pair with E368, a feature unique among the inhibitors in this study (Fig. 4D). Interestingly, E368 also forms a hydrogen bond with R12 of the KMT2A peptide (Supplementary Fig. 10G)^23^. For DS-1594, the compound extends the farthest along alpha helix 12, positioning it near C334 (Fig. 4E). Thus, the C334R mutation likely confers unique resistance to DS-1594 by causing a steric clash with the molecule. Lastly, the co-crystal reveals that KO-539 makes a hydrogen bond with W346 through a nitrile group (-C ≡ N) aligned in a nearly collinear 180° orientation, however this angle is slightly reduced (Fig. 4F). The V372A substitution, which uniquely causes resistance to KO-539, likely works through weakening this bond as V372 is positioned above W346. This constrains its alignment and strengthens the hydrogen bond to form with the nitrile. We mapped the absolute IC_50_ shifts in viability assays (Fig. 3A, B) onto the co-crystal structure of each menin inhibitor bound to menin (Fig. 4G). Amino acids that conferred greater resistance were labeled with a more intense color of red. Taken together, investigating the co-crystal structure of each menin inhibitor bound to menin suggests a mechanism of action for most *MEN1* mutations identified in our CRISPR base editor screen.

### De novo *MEN1* mutation emergence validates base editor screen

**Fig. 5:**
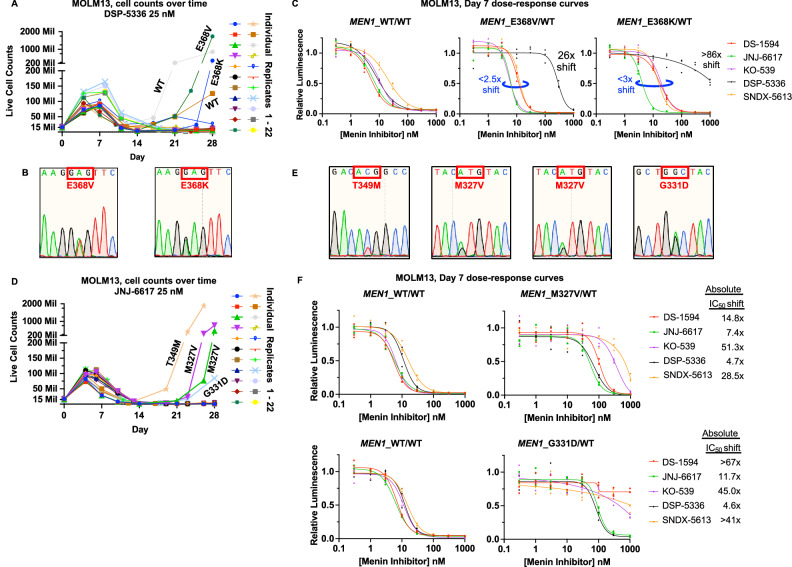
De novo *MEN1* mutation emergence in vitro corroborates screen. **A**,**D** 12.5-to-15 million MOLM13 cells per replicate across 22 individual replicates were seeded on Day 0. Live cell counts tracked over time. Cells continuously grown in 25 nM DSP-5336 (**A**) or 25 nM JNJ-6617 (**D**). Data shown from *n* = 4 independent experiments, each with 3-to-9 individual replicates. **B**,**E** *MEN1* Sanger sequencing for *MEN1* mutations detected in exponentially growing clones annotated. **C**,**F** Viability (CellTiter-Glo) assays assessed at 7 days for *MEN1* mutant clones detected in **A** and **D** respectively. Cells were taken from experiments in **A** and **D**, removed from drug, and treated with each of the 5 menin inhibitors. The shift in the absolute IC_50_ was quantified by dividing the absolute IC_50_ in the *MEN1* mutant cell line versus the absolute IC_50_ in a wild-type cell line seeded on the same day and measured on Day 7. For E368 data, representative examples from two biological replicates are shown, each with technical triplicates. For M327V data, four experimental replicates were performed across WT control cells and each M327V clone (*n* = 4 WT experiments, *n* = 2 for each M327V clone), each with technical triplicates. For G331D data, representative example from three biological replicates are shown, each with technical triplicates. Source data contains Absolute IC_50_ values for biological replicates with 95% confidence intervals, as well as ANOVA statistical testing.

To assess whether the *MEN1* mutations predicted by the screen will arise spontaneously, we utilized in vitro and in vivo models of *MEN1* mutation generation. Utilizing the MOLM-13 cell line, we seeded >12.5 million cells in each of 22 replicates and treated with 25 nM DSP-5336 (Fig. 5A). Cells expanded over the first 4 to 7 days, followed by cell death and decreased cell numbers. By Day 24, four of the 22 replicates were rapidly growing despite the presence of drug. Sanger sequencing was performed, finding an E368K mutation in one replicate, an E368V mutation in another replicate, and two replicates with wild-type sequences only (Fig. 5B). The early outgrowth of these *MEN1* resistant clones suggests that the mutations either arose shortly after initiation of menin inhibitor therapy or were pre-existing at very low levels prior to treatment. Remarkably, the CRISPR base editor screen and validation identified E368 as the most deleterious residue for the DSP-5336 compound (Figs. 2C, 3A–D). Both replicates with E368 mutations were subsequently removed from drug and treated in CellTiter-Glo viability assays with all 5 menin inhibitors (Fig. 5C). Consistent with mutation at E368K in MV4;11 C > T cells, this *MEN1* mutation essentially selectively affected DSP-5336 (>86x shift), conferred increased sensitivity to JNJ-6617 and SNDX-5613 (each 0.7x and 0.8x respectively), and shifted the absolute IC_50_ of DS-1594 and KO-539 less than 3x. Two of the four resistant clones did not have a *MEN1* mutation hotspot detected, suggesting other resistance mechanisms may play a role.

We performed a similar experiment with JNJ-6617 and in two replicates found M327V mutations, in a third replicate a T349M mutation, and in a fourth found a G331D mutation (Fig. 5D). Interestingly, the T349M mutation did not appear as a heterozygous mutation and therefore was likely not the only resistant clone in the culture (Fig. 5E). When drug was washed out of the M327V and G331D clones, viability assays demonstrated pan-resistance, with the absolute IC_50_ shifted at least 4.5x for each menin inhibitor for each mutation. For M327V, the most deleterious effects were observed for KO-539 (51x) and SNDX-5613 (29x), less of an effect for DS-1594 (15x), and JNJ-6617 (7.4x) and DSP-5336 (4.7x) were the least affected (Fig. 5F). For G331D, DS-1594 (>67x), SNDX-5613 (>41×), and KO-539 (45x) were the most affected, with JNJ-6617 (12×) and DSP-5336 (4.6×) affected to a lesser extent. Remarkably, for JNJ-6617, mutations at M327 and T349 induced by sgRNAs from the CRISPR base editor screen shifted the absolute IC_50_ to the greatest extent in the base editor screen validation (Fig. 3A, B). These in vitro experiments thus establish that the most deleterious sgRNAs from the CRISPR base editor screen and validation are likely to arise and impact these compounds. In contrast to menin inhibitor monotherapy selection of resistant clones, we also treated twelve replicates with the combination of JNJ-6617 25 nM and DSP-5336 25 nM. Although limited by sample size, we found no outgrowth of resistant clones (Supplementary Fig. 11A, B). However, it should be noted that the suppression of resistant clones observed with 25 nM of each compound may reflect the higher total menin inhibitor exposure, 50 nM combined, rather than a true benefit from combining two menin inhibitors with different *MEN1* mutation resistance profiles.

**Fig. 6:**
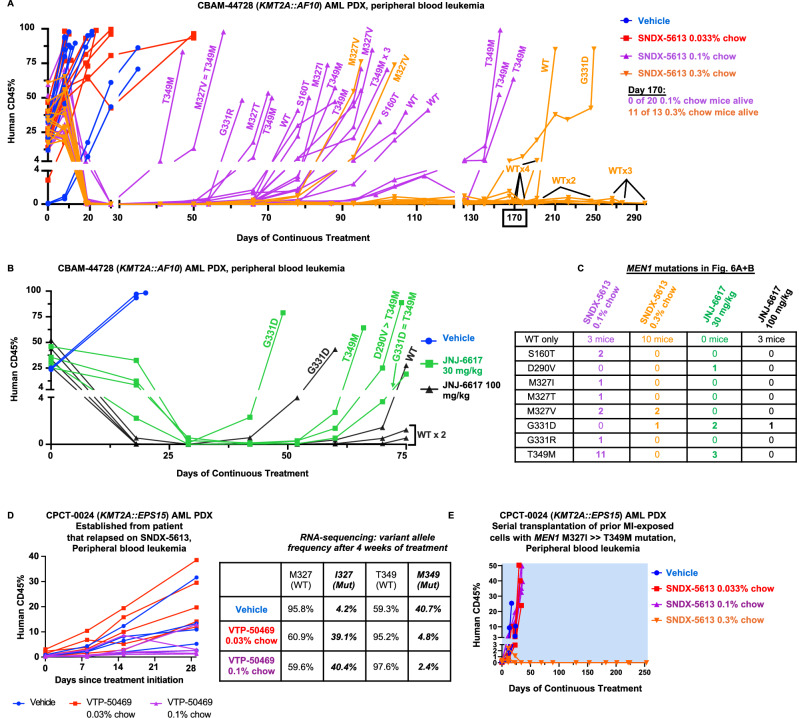
De novo *MEN1* mutation emergence in vivo corroborates screen. **A**, **B** CBAM-44728 (*KMT2A::AF10*) AML PDX experiments. Mice euthanized at humane endpoint, upon reaching 30% disease in the peripheral blood after an initial response, or after 75 days of treatment (JNJ-6617 experiment, **B**, only). *MEN1* mutation status, assessed by Sanger sequencing of bone marrow specimens at time of death, is annotated on the graph with the largest *MEN1* mutation shown. **A** Data inclusive of two separate experiments, for a total of *n* = 10 vehicle mice, *n* = 10 SNDX-5613 0.033% chow mice, *n* = 20 SNDX-5613 0.1% chow mice, and *n* = 13 SNDX-5613 0.3% chow mice. No *MEN1* mutations found in vehicle or SNDX-5613 0.033% cohorts. **B** *n* = 2 vehicle, *n* = 4 JNJ-6617 30 mg/kg/day PO gavage, *n* = 4 JNJ-6617 100 mg/kg/day PO gavage. No *MEN1* mutations found in vehicle mice. **C** *MEN1* mutations detected by Sanger sequencing in each cohort shown in Fig. 6A, B. For each mouse, the predominant *MEN1* mutation is annotated. Mice harboring two mutations of comparable size are represented in multiple rows (see Supplementary Fig. 13A+B for all *MEN1* mutations detected). **D** CPCT-0024 (*KMT2A::EPS15*) AML PDX established from a patient that acquired resistance to SNDX-5613. Treatment groups included vehicle control (*n* = 5), VTP-50469 (pre-clinical analogue of SNDX-5613) 0.033% chow (*n* = 5), and VTP-50469 0.1% chow (*n* = 5). Peripheral blood leukemia burden after 4 weeks of treatment is shown on the left. RNA-sequencing interrogated for variant allele frequency at Day 28 for *n* = 2 mice per cohort for M327 (WT, wild-type) versus M327I (Mut, mutant) and T349 (wild-type) versus T349M (mutant). **E** CPCT-0024 (*KMT2A::EPS15*) AML PDX, engrafted with cells having had previous VTP-50469 exposure and resultant pre-existing, heterozygous M327I mutation (Fig. 6B, Supplementary Fig. 13C). Once the PDX engrafted and treatment was initiated, mice received continuous menin inhibitor treatment until a humane endpoint was reached. Treatment groups included vehicle control (*n* = 3), SNDX-5613 0.033% chow (*n* = 4), SNDX-5613 0.1% chow (*n* = 4), and SNDX-5613 0.3% chow (*n* = 4).

The other way we sought to orthogonally validate the screen was through utilization of patient derived xenograft (PDX) models. We engrafted the CBAM-44728 PDX (*KMT2A::AF10*) model and treated with three different doses of SNDX-5613 in two separate PDX experiments (combined results shown in Fig. 6A, C, Supplementary Fig. 12A, B). For SNDX-5613, the lowest dose (0.033% chow) confers plasma concentrations below what is observed in patients, 0.1% chow confers a dose that is similar to plasma concentrations in patients, and 0.3% chow provides higher concentrations than achieved in patients^9^. Mice treated with 0.033% chow (*n* = 10) were refractory to treatment. At the 0.1% chow dose, 17 of 20 mice relapsed with *MEN1* mutations within 170 days of treatment, inclusive of S160T, M327I/V/T, G331R/D, and T349M mutations, with 3 of 20 mice having only wild-type sequences at these residues (Supplementary Fig. 13A). Of note, an M327T mutation has not yet been observed in patients but was quite deleterious in viability assays (Fig. 3B, C). At the 0.3% chow dose, only 2 of 13 mice relapsed with *MEN1* mutations within 170 days of treatment initiation, both with M327V mutations, and a third relapsed with a G331D mutation after 250 days of continuous treatment. 10 mice in this 0.3% chow cohort died after 170 days on treatment without *MEN1* mutations at these residues. In a separate experiment in this PDX model, we treated mice with JNJ-6617 at 30 or 100 mg/kg/day by oral gavage for 75 days (Fig. 6B, C). All four mice treated with 30 mg/kg/day developed *MEN1* mutations (G331D, T349M, or D290V), whereas only one of four mice treated with 100 mg/kg/day developed a mutation (G331D) (Supplementary Fig. 13B). For SNDX-5613, the four residues mutated in vivo were among the six most deleterious in viability screens. For JNJ-6617, the three residues mutated in vivo ranked among the nine most deleterious (Fig. 3A). These experiments demonstrate that the mutations identified by the CRISPR base editor screen that shift the absolute IC_50_ in validation experiments are likely to be the mutations that arise in PDX models and patients. Further, this model demonstrates that *MEN1* mutations can be prevented or overcome with highly potent menin inhibitor therapy (SNDX-5613 0.3% chow or JNJ-6617 100 mg/kg/day).

The capacity for the dose of menin inhibitor chow to preferentially select for, or even overcome, *MEN1* mutations is further illustrated by the CPCT-0024 (*KMT2A::EPS15*) AML PDX model (Fig. 6D, E, Supplementary Fig. 12A), which we established from a patient that initially responded but then relapsed on menin inhibitor therapy. After 28 days of treatment with a pre-clinical analogue of SNDX-5613 (VTP-50469)^4^, RNA-sequencing was performed from *n* = 2 mice in each cohort. The vehicle mice contained both *MEN1* T349M (40.7% of all transcripts) and M327I mutations (4.2% of all transcripts), suggesting these *MEN1* mutations had arisen in the patient (Fig. 6D). In the mice treated with either dose of VTP-50469, the *MEN1* T349M mutation was nearly eradicated ( < 5% of all transcripts) and the dominant mutation was *MEN1* M327I ( ~ 40% of all transcripts). When a cohort of mice was subsequently engrafted with cells harvested from mice that previously received menin inhibitor therapy in vivo (i.e., cells that were predominantly M327I mutant) the mice were refractory to 0.033% and 0.1% SNDX-5613 chow but cured with 0.3% chow (Fig. 6E, Supplementary Fig. 13C). These results demonstrate that increasing potency of menin inhibitor therapy can select for more deleterious *MEN1* mutations in vivo and that higher potencies of menin inhibition can overcome even the most deleterious *MEN1* mutations.

## Discussion

The emergence of acquired *MEN1* mutations in response to SNDX-5613 treatment supports the importance of the menin-KMT2A interaction in both *KMT2A-r* and *NPM1c* acute leukemias^15^, in addition to other *MEIS1/HOX* expressing leukemias that have demonstrated acquisition of *MEN1* mutations with menin inhibitor therapy, such as *UBTF*-TD AML^24,25^. Utilizing a combined approach of CRISPR base editor screening, biochemical and structural analyses, and in vitro and in vivo validation methods, this work demonstrates that the unique binding of each menin inhibitor is likely to give rise to a different profile of *MEN1* mutations. Overall, our data suggest that while some *MEN1* mutations may attenuate activity to most menin inhibitors, such as M327 (in particular M327T mutations) and G331D mutations, there might be opportunities to switch patients with select *MEN1* mutations to alternative menin inhibitor therapy. For example, the *MEN1* E368K mutation, which was the top hit for the DSP-5336 compound in the CRISPR base editor screen and spontaneously arose in vitro with DSP-5336 treatment, shifts the absolute IC_50_ > 75-fold to DSP-5336 yet demonstrates increased sensitivity to JNJ-6617 and SNDX-5613. However, the data presented also demonstrates that low allele frequency *MEN1* mutations should be considered in the context of switching menin inhibitor therapy, as a less abundant and more deleterious *MEN1* mutation may arise when successfully treating the more abundant and less deleterious *MEN1*-mutant cells.

Utilizing in vitro and in vivo models, we found that many of the top hits from the CRISPR base editor screen can be spontaneously generated with continuous menin inhibitor treatment. For SNDX-5613, 4 of the 6 *MEN1* mutations induced by CRISPR base editing that shifted the absolute IC_50_ > 5x recurrently arose in vivo in a PDX model and have arisen in patients (S160, M327, G331, and T349). This strongly suggests that the *MEN1* mutations we identified that confer similar resistance to other menin inhibitors are likely to arise in patients as well. Intriguingly, our in vivo work in PDX models also suggests that *MEN1* mutations can be prevented and overcome with higher potency menin inhibitor therapy. Whether sufficiently high dose menin-KMT2A inhibition to suppress *MEN1* mutation development can be achieved in patients remains to be seen. Our work suggests that higher dose levels may be helpful with regard to suppressing *MEN1* mutation-driven resistance.

There are several limitations to this CRISPR base editor screening approach. For example, it is likely that mutations at clinically relevant amino acids were not made even with the enhanced base editing technology utilized. For example, G331D mutations have arisen in patients receiving SNDX-5613 on the AUGMENT-101 trial and recent work has demonstrated that this mutation may be particularly difficult to overcome given the slow dissociation of *MEN1* G331D from KMT2A^26^. In our sgRNA library, while three sgRNAs targeted G331, they predominantly caused G331N mutations. However, our orthogonal validation in vitro and in vivo demonstrated that G331D mutations arose spontaneously with SNDX-5613 and JNJ-6617, with this mutation causing pan-resistance to menin inhibitors. The base editor screen alone did not directly predict this key mutation. Furthermore, comparisons between different *MEN1* mutations affecting each compound are limited by bystander mutations at adjacent amino acid residues to those targeted by the sgRNA, the efficiency with which an sgRNA induces a *MEN1* mutation, and the percentage of alleles mutated.

Overall, this work demonstrates the capacity of CRISPR base editing to accurately predict the *MEN1* mutational landscape that arises with SNDX-5613 and offers a prediction of the *MEN1* mutations that may arise with the other menin inhibitors in clinical trials. This work may guide discovery of next generation menin inhibitors and should empower clinical investigators to look for *MEN1* mutations beyond those reported with SNDX-5613. Furthermore, there may be opportunities to enroll patients with select *MEN1* mutations onto alternative menin inhibitor clinical trials and catalogue their responses. While this work focuses on *MEN1* mutation-driven resistance, resistance mechanisms without *MEN1* mutations are actively being investigated and warrant further characterization in preclinical models and patient samples to inform the rational design of combination therapies to overcome resistance^15,27–29^.

## Methods

The research in this manuscript complies with all relevant ethical standards. Animal experiments were approved by Dana-Farber Cancer Institute’s Institutional Animal Care and Use Committee (protocol number 16-021). We have complied with all relevant ethical regulations outlined in our animal protocol.

### Menin inhibitor synthesis

The structures of SNDX-5613, KO-539, DS-1594 and JNJ-6617 have been described^6,15,16,18,19^. All menin inhibitors used in this study were prepared based on synthetic routes described in the patent applications for the respective compounds. See Supplementary Data 1 for menin inhibitor analytical data.

### Generation of MV4;11 cell lines overexpressing base editor enzymes and sgRNAs

MV4;11 cell lines constitutively overexpressing one of two distinct base editor enzymes (C > T or A > G enzymes) were generated using lentiviral transduction. Viral supernatants for the base editors were produced by transfecting HEK293T cells with packaging plasmids pMD2.G (Addgene plasmid #12259) and psPAX2 (Addgene plasmid #12260) along with plasmids encoding either base editor enzyme (A > G editor: ABE8e (*V106W*), SpG-SpyoCas9 (*D10A*), pRDA_867, Addgene plasmid #231109; C > T editor: TadA-CDd, SpG-SpyoCas9 (*D10A*), pRDB_092, Addgene plasmid #231110)^20,21^ using XtremeGene transfection reagent (Millipore-Sigma-Roche), following the manufacturer’s instructions. After 48 hours of incubation the viral supernatants were harvested, filtered and used to infect MV4;11 cells. Viral transduction was carried out by spinfection. Cells were first incubated for 45 minutes at 37 °C in viral supernatants supplemented with polybrene, then cells were centrifuged at 1100 g for 1 h and 50 min at 37 °C. Viral supernatants were then removed and cells were allowed to recover for 24 h and then selected in culture medium containing blasticidin.

*MEN1* sgRNA library viral production was carried out as described above. However, the resulting viral supernatant was additionally concentrated using a 100 kDa centrifugal filter (Sigma Aldrich, Amicon Ultra Centrifugal Filter, UFC 9100024), frozen, thawed, and titered for multiplicity of infection (MOI) estimations. Single sgRNAs for validation cell lines were cloned into pUSEPR-RFP-IRES-Puro lentiviral expression vectors using standard methods, inserting oligonucleotides synthesized by Integrated DNA Technologies and ligated into the BsmBI cloning site. To introduce single guide RNAs, lentiviral supernatants were produced in 293T cells using the transfection reagent TransIT-L1 (Mirus Bio). The resulting viral supernatants were used for a second round of spinfection, as described above, in MV4;11 cells expressing base editor enzymes (A > G or C > T). After transduction, cells were selected with puromycin, and successful transduction was assessed by flow cytometry using an LSR Fortessa (Becton Dickinson), with RFP expression as a reporter. MV4;11 cells exhibiting RFP expression were established as stable cell lines expressing the base editor enzymes and their respective guide RNAs, resulting in *MEN1* mutations. For the establishment of individual MV4;11 cell lines with sgRNAs that enriched log_2_FC ≥ 4 in the CRISPR base editor screen, 41 sgRNAs in either the A > G or C > T arm of the screen reached this threshold. Cloning failed, however, for an sgRNA (ACCTACTGGGCTCCAACCTG) predicted to make a *MEN1* Val153 edit in both the A > G and C > T cell lines.

### CRISPR base editor screen

An sgRNA library targeting *MEN1* exons (target transcript: NM_000244.4) was designed by the Broad Institute, inclusive of 1239 sgRNAs, 124 onesite sgRNAs targeting intergenic (negative) control sequences, and 32 sgRNAs targeting splice donor sequences in 10 common essential genes (*RPA3*; *PCNA*; *DBR1*; *PLK1*; *RPL3*; *GAPDH*; *KIF11*; *PSMB1*; *EEF2*; *POLR2B*). The guide library was cloned into library cloning vector pMV_AA013 (Addgene plasmid #215975), which contains puromycin resistance and EGFP.

Viral supernatant for the *MEN1* sgRNA library were produced by transfecting HEK293T cells as described above. Test infections in MV4;11 C > T and A > G cells found that 37.5 uL of concentrated virus for 1 million cells conferred about a 15% infectivity rate after 48 hours, as assessed by GFP positivity by flow cytometry. To ensure >2000x library coverage, 33.3 million MV4;11 A > G and 33.3 million MV4;11 C > T cells were resuspended separately in 50 mL of media with 42 uL of polybrene and 600 uL of concentrated virus. ~2 mL of cells, media, polybrene, and virus was split between 24 conical tubes per cell line and incubated for 45 minutes at 37 °C prior to spinfection at 2200 RPM for 1 hour and 45 minutes. 48 hours after spinfection, the transduction efficiency was 15.1% for the MV4;11 A > G base editor and 15.7% for the MV4;11 C > T base editor, at which point puromycin was added. Cells were then expanded in puromycin until day 10 following library transduction, and then split into 6 different conditions per cell line, each with three individual replicates: 1. DMSO control, seeded at 5 million cells per flask, 2. DS-1594, seeded at 15 million cells per flask, 3. JNJ-6617, seeded at 15 million cells per flask, 4. KO-539, seeded at 15 million cells per flask, 5. SNDX-5613, seeded at 15 million cells per flask, 6. DSP-5336, seeded at 15 million cells per flask. Additionally, 5 million cells were harvested for a Day 0 early timepoint per cell line, in triplicates. We averaged the 75% growth inhibitory concentration at day 5 and the 97.5% growth inhibitory concentration at day 9 (Supplementary Fig. 1A) and used these as the concentration during the screen: 13.8 nM DS-1594, 17.3 nM JNJ-6617, 23.4 nM KO-539, 51.6 nM SNDX-5613, 25.2 nM DSP-5336. Drug stocks were prepared so equal volumes of DMSO were administered per treatment flask and DMSO control. Cells were split and drug replenished every 3 to 4 days during the screen. To ensure equal drug exposure, the cells being passaged forward were spun down, the prior media was aspirated, and cells were resuspended in fresh media with fresh drug. No cells were discarded during the first 7 days of the screen, and thereafter cells were split to maintain at least 2000x coverage.

At day 21, cell pellets containing 5 million cells per condition were collected, washed, and frozen down. DNA was extracted following the Broad Institutes Genetic Perturbation Platform’s recommendations, which utilizes the Machery Nagel NucleoSpin Blood Kit (Takara Cat. #740951). Samples were then purified with a PCR inhibitor removal column (Zymo Research, #D6030). PCR pre-check to confirm presence of library vector and PCR efficiency was performed for one of each technical triplicate (P5 primer sequence: 5’-TTGTGGAAAGGACGAAACACCG-‘3’; P7 primer sequence: 5’-ACCGACTCGGTGCCACTTTTTCAA-3’). Up to 10 µg of DNA per technical triplicate was submitted in a PCR plate to the Broad for sequencing (HiSeq 2500). Samples sequenced included DNA pellets harvested at day 0 as a baseline, in three technical replicates, in addition to DMSO control and each of the 5 menin inhibitor conditions harvested at Day 21, also in three technical replicates.

Data output from sequencing was in the form of log normalized count matrix that standardized read counts based on reads per million. The Log_2_ fold change (Log_2_FC) was then calculated by subtracting the day 0 log-normalized count from the day 21 log-normalized count. In generating Fig. 2C and Supplementary Fig. 1C, the y-axis depicts the Log_2_FC and the x-axis the amino acid the sgRNA targets. For some guides, two or three amino acids were possibly targeted, and in these instances, the average amino acid number was chosen for graphical display (e.g., the average of amino acid edit 510 and 511 would be displayed at 510.5). For some guides, an amino acid edit was predicted in either the A > G or C > T cell line but not both. In these instances, the predicted amino acid edit in the other cell line was chosen for graphical display (e.g., if a guide was not predicted to make an amino acid edit in the A > G cell line but was predicted to make an edit at amino acid 510 in the C > T cell line, amino acid 510 was chosen for graphical display in the A > G cell line). For some guides, an amino acid edit was not predicted in either the A > G or C > T cell line. In these instances, for graphical display the average of the adjacent sgRNA predictions based on the sgRNA target sequence start position was taken (e.g., if a guide was not predicted to make an edit both the A > G and C > T cell lines, but adjacent sgRNAs were predicted to make an edit at 510 and 512 respectively based on sgRNA target sequence start position, amino acid 511 was plotted on the graph).

Guide-level enrichment statistics were computed using MAGeCK. Because a given sgRNA can yield different editing outcomes in the MV4;11 A > G and C > T base editor cell lines, the library and samples were analyzed as two independent screens. Separate MAGeCK library definition files were constructed for the A > G and C > T datasets, each containing 1. *MEN1*-targeting sgRNAs, 2. intergenic control sgRNAs, and 3. positive control sgRNAs. For guide-level inference, each unique sgRNA sequence was assigned a unique “gene” identifier (for example, MEN1-1_AG or MEN1-1_CT), enabling MAGeCK’s gene-level models to return per-guide effect estimates and statistics. For samples sequenced across two wells representing the same biological replicate, FASTQ files were merged prior to counting. Read counting was performed with mageck count using the corresponding split library file. Differential selection was then modeled with the MAGeCK maximum likelihood estimation framework (mageck mle) using a design matrix comparing day 21 samples to the matched day 0 baseline within each condition. MAGeCK MLE output statistics (beta, z-score, P value, Wald P value, and Wald FDR) were reported for each guide-level “gene” and are provided in Supplementary Data 2.

### CRISPR base edited cell lines with individual guides competition assay

MV4;11 C > T and A > G cells expressing individual sgRNAs that scored highly during the screen were introduced into single cell lines as described above. On day 0, 80% MV4;11 C > T or A > G cells without an sgRNA were mixed with 20% MV4;11 C > T or A > G cells with a high-scoring sgRNA from the screen. Cells were split and drug was replenished at Day 4 and the percentage of RFP+ (PE + ) cells on day 9 was calculated by flow cytometry. All conditions had at least three technical replicates.

### Purification of recombinant menin and crystallography methods

The wild-type and mutant (I327 and M349) human menin proteins were purified at the same time as our previously published study on acquired *MEN1* resistance related to SNDX-5613 and the associated methods have previously been described^15^. The co-crystal structures presented here of JNJ-6617, DSP-5336, KO-539 and DS-1594 bound to wild-type M327 and mutant I327 menin were obtained under similar crystallization conditions at the same time as those with SNDX-5613 but were withheld from publication until now. Briefly, crystals were grown via sitting-drop vapor diffusion with protein at 10-11 mg/mL, in buffers containing various PEG and salt concentrations, and supplemented with inhibitor. Crystals typically formed over 7-10 days at 21 °C. Data collection, structure solution, and refinement were carried out by standard methods. The majority of the structures are of high resolution. Specifically, 6 out of the 8 structures have a resolution of ≤ 2.0 Å, with the best resolution being 1.5 Å for one of the structures (Supplementary Table 1). An image of a portion of the electron density map (including contour level) for each crystal structure can be found in Supplementary Data 6. Omit maps (including contour level) for all ligand-bound crystal structures can be found in Supplementary Data 7. Overall, the ligand and the specific mutation site are well-resolved and show a good fit in the electron density maps. The co-crystal structures of SNDX-5613 bound menin proteins have previously been uploaded to PDB with the following accession numbers Protein Data Bank (PDB) ID# 7UJ4 (wild-type) and PDB ID#8E90 (I327) and are analyzed here. Co-crystal structures for other inhibitors have also been uploaded to PDB with the following accession numbers: DS-1594 bound menin ID# 9WKW (wild-type) and ID# 9WKX (I327), DSP-5336 bound menin ID# 9WN9 (wild-type) and ID# 9WNA (I327), JNJ-6617 bound menin ID# 9WKU (wild-type) and ID# 9WKV (I327), and KO-539 bound menin ID# 9WNI (wild-type) and ID# 9WNJ (I327).

### M327 vs. I327 menin inhibitor positional analysis and visualization

M327 vs. I327 menin inhibitor positional analysis and visualization was performed in PyMOL (Schrödinger, LLC). Each unique menin inhibitor bound M327 and I327 protein co-crystal structure pairings were aligned using the ‘align’ command. These alignments compared the positions of all Cα atoms deriving Root Mean Square Deviation (RMSD) values to quantify backbone similarity. Derived RMSD values for M327-I327 pairs were as follows: KO-539/0.368 Å, DSP-5336/0.273 Å, DS-1594/0.401 Å, SNDX-5613/0.338 Å, and JNJ-6617/0.321 Å. To enable menin inhibitor positional comparisons due the M327I substitution, distances between the matched atom pairs of the bound ligands were measured in Euclidean distance (Å) using the coordinates derived from the ‘cmd.get_atom_coords’ function. For Supplementary Fig. 10B-F these values were used to generate heatmap coloring of the mutant bound ligand, using the ‘spectrum b, blue white_red’ function with a minimum of 0 Å and maximum of 1 Å (values greater than 1 Å were capped to enhance smaller displacements) and then the wild-type bound ligands were colored with the ‘set stick_transparency’ function to illustrate the movement in the pocket.

### Mapping and visualization of CRISPR base editor screen hits onto protein structures

Mapping and visualization of base-editing readouts (or “hits”) on protein structures was performed using the Genomics 2 Proteins portal^22^ after preprocessing the screening results using in-house scripts. During the preprocessing, we filtered out all synonymous and noncoding hits. Guides resulting in both single and multiple point mutations at a protein residue were analyzed. Next, the predicted base edited sites from the sgRNA library were aligned to the protein residues from UniProt “long” isoform sequence (O00255-2) and the three-dimensional coordinates of the residues in the structures. An alignment step was performed for all menin inhibitor bound menin structures to account for a residue indexing discrepancy starting at residue 464 and ending at position 557 (i.e., there is a gap in the structure which is not reflected in the residue numbering within the structure file). Additionally, base-editing readouts at residue position 4 were discarded because all analyzed structures had a mutation at this position. Once all readouts are successfully aligned to the structure (a total of 762 A > G hits and 1026 C > T hits), they are mapped to 5 separate PyMol session (.PSE) files as separate annotations. The annotations were named (*i*) “mutations_all”: captures all base-editable sites, (*ii*) “mutation_over_1” and “mutations_under_−1”: annotate hits with screening scores over 1 and under −1 respectively, (*iii*) “mutations_over_3.75”: annotates hits with screening scores over 3.75 from the screen, and (*iv*) “mutations_over_any_screen_3.75”: annotates hits from all screens over 3.75. An additional 5.PSE files were generated with the process mentioned above but with along relative to DMSO IC_50_ shift results derived from single sgRNA expressing MV4;11 base editor expressing cells from validation experiments from selected amino acid residues with all other residues set to 0. The command “spectrum b, grey_red, minimum=0, maximum=17.5” was used to quantitatively color select amino acids and generate Fig. 4G. The resulting 10.PSE files, 2 per menin inhibitor bound structures, that were generated in this process are presented as Supplementary Data 3.

### KMT2A-menin TR-FRET displacement assays

Purified wild-type and mutant menin (M327I, G331R, and T349M) proteins were evaluated for KMT2A-menin displacement using a TR-FRET assay based on a FITC-KMT2A-4-43 probe using similar methods as previously described^15^. Briefly, menin proteins were labelled with anti-HIS-Tb and diluted in a buffer containing 50 mM Tris, pH 7.4, 50 mM NaCl, 0.02% BSA, 1 mM DTT, and 0.005% Triton X-100. Menin/anti-HIS-Tb was then incubated with the FITC-KMT2A-4-43 probe at 0.2 nM for IC_50_ assays. DSP-5336, JNJ-6617, DS-1594, KO-539, and SNDX-5613 were serially diluted in DMSO then added to protein/probe combinations using an experimental setup including at least 8 dose concentrations with two technical replicates per dose, followed by incubation overnight. An Envision multimode plate reader was used to measure both 320 nm excitation and 520/490 nm emission. The data was presented as percent inhibition plotted against compound concentration to derive IC_50_ values using four parameter logit non-linear curve fitting (XLFit, IDBS).

To estimate *K*_i_ values, we first performed Surface Plasmon Resonance (SPR) binding studies using a T200 SPR system (Cytiva) to estimate the *K*_d_ of the wild-type menin/FITC-KMT2A(4-43) interaction. Binding of FITC-KMT2A(4-43) was assessed by capture, multi-cycle kinetics using a high-density nickel chip (NIHC1500M, Xantec Bioanalytics). N-terminal His6-tagged menin was immobilized at two different surface densities (either 580 RU or 1250 RU) prior to injection of peptide. After each injection of peptide, the His6-tagged menin was removed from the chip with 0.35 M EDTA followed by recharge with 5 mM NiCl2. Flow cell 1 was activated and blocked as a control surface. FITC-KMT2A(4-43) was injected from 1-100 nM using half-log (1:3.16) dilutions, in duplicate. Association time was 90 s, dissociation time was 600 s, flow rate was 30 uL/min. The running buffer contained 10 mM Tris, pH 7.4, 150 mM NaCl, 0.2 mM TCEP, 0.025% Tween20. Data were collected at 25 ^o^C. Global fits were determined for the data on each different density surface using a 1:1 interaction model. The *K*_d_ for 580 RU was 170 pM and for 1250 RU was 200 pM. We thus used a *K*_d_ of 185 pM for wild-type menin. See Supplementary Table 3 and Supplementary Data 4 for a summary of *K*_d_ and raw data underlying SPR.

To determine k_off_ of the FITC-KMT2A-4-43 peptide for WT and mutant menin, HIS-Menin (2 nM) was pre-incubated with anti-HIS-Tb antibody (1 nM) for 30 min at room temperature. The FITC-MLL-4-43 (2 nM final) was added and the resulting HTRF signal at 520/620 was measured until the binding reached equilibrium ( ~ 45 min). To initiate dissociation, excess SNDX-5613 (10 µM final) was added and the HTRF signal was measured every 90 s for 30 minutes. The fraction bound was calculated based on the 0% and 100% bound controls for each time point. These percent bound values were plotted versus time and fit to a one-site exponential decay model. To estimate the relative differences in *K*_D_ among wild-type and mutant menin forms, we determined k_off_ of the FITC-KMT2A(4-43) peptide. HIS-Menin (2 nM) was pre-incubated with anti-HIS-Tb antibody (1 nM) for 30 min at room temperature. The FITC-KMT2A(4-43) (2 nM final) was added and the resulting HTRF signal at 520/620 was measured until the binding reached equilibrium ( ~ 45 min). To initiate dissociation, excess SNDX-5613 (10 µM final) was added and the HTRF signal was measured every 90 s for 30 minutes. The fraction bound was calculated based on the 0% and 100% bound controls for each time point. These percent bound values were plotted versus time and fit to a one-site exponential decay model. Based on the K_off_ ratio for each menin mutant relative to wild-type menin, *K*_D_ adjustments relative to wild-type were calculated as 0.63x (117 pM) for M327I, 0.55x (102 pM) for T349M and 1.9x (352 pM) for G331R. Finally, *K*_i_ values were calculated based on the following equation: *K*_i_ = IC_50_ / (1 + [FITC-KMT2A(4-43)] / *K*_D_). See Supplementary Table 4 and Supplementary Data 5 for summary of off-rate kinetics and raw data underlying these calculations.

### KMT2B-menin TR-FRET displacement assays

For TR-FRET assays involving mutant menin proteins E368K, C334R and V372A experimental conditions differed from above and more closely resembled previously published methods from Kwon et al.^6^. Here, and as previously described, we purified IPTG induced expression of StrepII-tagged mutant and wild-type menin proteins from Escherichia coli overnight cultures by lysing, sonicating and ultracentrifuging resulting cell pellets. We then subjected lysate to Strep-Tactin XT (IBA) affinity resin incubation, washing and elution. Resulting protein purifications were subjected to ion-exchange chromatography (PorosHQ) and menin proteins were further purified by size exclusion chromatography (Superdex200 10/300 GL) and then concentrated using ultrafiltration (Millipore). Purified menin proteins were then conjugated with biotin using an in-house purified BirA enzyme^30^. To enable drug binding affinity estimations, we leveraged our previously published N-terminally FITC-labelled and unmodified KMT2B(MLL2) peptides (KMT2B(15-48) SARGRFPGRPRGAGGGGGRGGRGNGAERVRVALR) that were synthesized by GenScript. Titrations of compounds in FITC-KMT2B(15-48) displacement assays were carried out by mixing 1 nM Tb (CoraFluor-1)-labeled streptavidin (R&D Systems, Cat#7920/20U), 4 nM of biotinylated menin variants and 1 nM FITC-KMT2B(15-48) in assay buffer containing 25 mM HEPES, pH 7.5, 150 mM NaCl, 0.05% (Sigma Aldrich P9416), 0.5% BSA (Cell Signaling Technology 9998S) and 2 mM TCEP and 0.05% Tween-20 [Sigma-Aldrich, Cat#9005-64-5]). Assay mixture was then combined with increasing concentrations of unlabeled KMT2B(15-48), DSP-5336, JNJ-6617, DS-1594, KO-539 and SNDX-5613 in 384-well format using a D300e Digital Dispenser (HP). Excitation and emission detection was carried out as previously described using a PHERAstar FS microplate reader (BMG Labtech). The TR-FRET signal was calculated by the 520/490 nm ratio. IC_50_ values were estimated using the variable slope equation in GraphPad Prism 10. All TR-FRET results were normalized to the percent inhibition relative to DMSO control and plotted as mean ± standard deviation. from three independent replicates (*n* = 3) derived from 2 technical replicates per concentration point. The calculation of *K*_i_ values were performed using the *K*_i_ calculator available at http://websites.umich.edu/~shaomengwanglab/software/calc_ki/index.html.

### Menin isoform annotations

Multiple *MEN1* and menin protein isoforms of have been used previously to annotated *MEN1* mutations^31^. For our *MEN1* base editor library we used NCBI Reference Sequence NM_000244.4. For our general numbering of amino-acid position we used the UniProt isoform O00255-1 which consists of 615-amino-acids which has a five-residue offset from the often used 610-residue isoform O00255-2. For our crystallographic data the 610-residue isoform O00255-2 was used as the standard reference for reporting these.

### Cell lines

Cell lines were purchased from DSMZ (Deutsche Sammlung von Mikroorganismen und Zellkulturen) or ATCC: MOLM13 (DSMZ, ACC554), MV4;11 (ATCC, CRL-9591), OCI-AML3 (DSMZ, ACC-582). Cell lines were cultured in RPMI 1640 (Gibco, catalog numbers: 11875119, 11875135) supplemented with 10% fetal bovine serum (FBS), 1% penicillin/streptomycin (Gibco 15140163), and 1% L-glutamine (Gibco 25030164) at 37 °C with 5% CO2. HEK293T was cultured in DMEM with 10% FBS. Cell lines were not authenticated during the duration of this study, but responded to menin inhibitors in dose-response curves as previously reported.

### Drug treatments, in vitro

Drug stocks were diluted in DMSO, stored at −80 °C, and then diluted into tissue culture medium (RPMI, 10% FBS, 1% penicillin/streptomycin, and 1% glutamine) prior to use.

### Animals for patient derived xenografts models, drug treatments in vivo

For patient derived xenograft (PDX) experiments, female NOD.Cg-Prkdc^scid^Il2^rgtm1Sug^/JicTac mice were purchased from Taconic or NOD.Cg-*Prkdc*^*scid*^ *Il2rg*^*tm1Wjl*^/SzJ from The Jackson Laboratory. Mice were between 8 and 12 weeks old at the time of xenotransplantation. Animal experiments were approved by Dana-Farber Cancer Institute’s Institutional Animal Care and Use Committee (protocol number 16-021). PDX samples were obtained from the Center for Pediatric Cancer Therapeutics at DFCI or Public Repository of Xenografts (ProXe)^32^. We have complied with all relevant ethical regulations outlined in our animal protocol. The mice used in this study were housed in solid-bottom, polysulfone 75 square inch microisolator cages. The cages are used in conjunction with the Optimice® rack systems with integrated automatic watering (Lixit water bottles). Temperature and humidity in the animal facilities are controlled at 72 + / − 2 °F and a target range of 35–55% relative humidity. A standard photoperiod of 12 hours light/12 hours dark is controlled by an automated system (6 AM to 6 PM).

Mouse tail veins were injected with 100,000-to-1,000,000 cells on day 0 based on prior patterns of time-to-engraftment. After detection of human CD45+ cells in the peripheral blood, mice were randomized to treatment with VTP-50469 or SNDX-5613. VTP-50469 and SNDX-5613 chow was provided by Syndax Pharmaceuticals.

JNJ-6617/JNJ-75276617 (bleximenib) oxalate was purchased from MedChem Express and prepared for in vivo dosing as described in Kwon MC, et al.^6^, JNJ-6617 was administered by daily PO gavage at 30 mg/kg or 100 mg/kg daily for up to 75 days.

The leukemia burden in the peripheral blood was monitored by submandibular bleeding, calculating the percentage of mouse CD45+ and human CD45+ cells that were human CD45 + . Red blood cell lysis was performed using RBC Lysis Buffer 10X (BioLegend Cell Staining Buffer Cat. No. 420201) before staining with PE-conjugated anti-human CD45 (BioLegend, 304058 or 304039, 1 μl antibody per 100 μl solution; https://www.biolegend.com/fr-fr/products/pe-anti-human-cd45-antibody-708) and APC-Cy7-conjugated anti-mouse CD45 (BioLegend, 304014 or 103116, 1 μl antibody per 100 μl solution; https://www.biolegend.com/fr-lu/products/apc-cyanine7-anti-human-cd45-antibody-1914). These studies were performed over several years with multiple different lot numbers and consistent staining results.

Mice were euthanized when humane endpoints were reached (CBAM-44728 Main Fig. 6A, CPCT-0024), when leukemia burden increased to greater than 30% following peripheral blood remission (CBAM-44728 Main Fig. 6A, B), or after 75 days of daily JNJ-6617 oral gavage (Main Fig. 6B). For survival experiments, mice were euthanized when showing signs of illness in accordance with animal protocol 16-021 filed with Dana-Farber Cancer Institute’s Institutional Animal Care and Use Committee. Humane endpoints included: tumors measuring 2 cm; weight loss exceeding 15% of the animal’s body weight, or interference with the animal’s ability to eat, drink, urinate or defecate; and anorexia. Select mice that had survived for extended periods of time (CBAM-44728, Main Fig. 6A and Supplementary Fig. 12B) were euthanized to harvest cells for further analysis prior to reaching humane endpoint. These mice are censored from survival analysis and noted in the source data file. At time of death, leukemic burden was confirmed and quantified in the bone marrow, spleen, and peripheral blood. Burden of disease analysis in the spleen and bone marrow was performed by homogenizing the spleen and crushing the bones (femurs, tibia, fibula, pelvic bones, and vertebral bodies), passing samples through a 40 µm filter, and then staining with human and mouse CD45 antibodies as detailed above.

Cells were then analyzed on an LSR Fortessa flow cytometer (Becton Dickinson).

### CellTiter-Glo® Luminescent Cell Viability Assays

Cells were plated in 96-well, flat bottom, non-tissue culture treated plates at a confluency of 2000-to-2500 cells/well on day 0 of the experiment. Cells were cultured in 200 µL of media. For the experiments in Figs. 1D, 5C, and 5F, cells were split at day 4 roughly back to their original seeding density and the assay was measured at day 7, or the cells were plated on Day 0 and measured on Day 7. For all other viability assays, cells were seeded at day 0 and read out on day 5.

Viability normalized to DMSO control and dose-response curves were plotted in GraphPad Prism Version 10, utilizing variable slope (four-parameter) analysis. Absolute IC_50_ was calculated using GraphPad Version 10.

Shifts in the absolute IC_50_ were calculated by dividing the absolute IC_50_ in *MEN1* mutant cell lines versus a *MEN1* wild-type cell line. For each absolute IC_50_ shift, the mutant and wild-type cell lines were plated on the same day and measured on the same day.

### In vitro models generating *MEN1* mutations

 ~ ≥12.5 million cells per flask were seeded on day 0 and treated with 25 nM of menin inhibitor. The experiments in Fig. 5 are inclusive of four separate experiments (for monotherapy treatments, *n* = 3, *n* = 9, *n* = 5, and *n* = 5) for a total of *n* = 22 total replicates. For combination experiments, there were *n* = 6 replicates per experiment across two separate experiments for *n* = 12 replicates. Live cells (DAPI (4′,6-diamidino-2-phenylindole) negative) were counted and expanded on day 4 with 1X drug added. Cell counts plateaued at day 4-to-7 and were followed every 3-to-4 days until about day 11-14, at which point the entire culture volume was spun down and resuspended in 1X drug. Counts were then assessed every 3-to-7 days with cell splitting and passaging at this point based on the number of live cells. Cells were pelleted, DNA extracted, and Sanger sequencing performed for control (DMSO treated) and menin inhibitor clones with exponential growth.

### *MEN1* mutation detection: Sanger sequencing and Amplicon sequencing

Genomic DNA extraction was performed using either the DNeasy Blood and Tissue Kit (Qiagen catalog number 69504) or Quick-DNA Plus Kits, Zymo Research - Quick-DNA Microprep Plus Kit (Catalog number D4074). To assess for *MEN1* mutations, the following primers were used:

- Menin amino acids 80 to 153:
  - ◦ Forward primer: CCAGAAGACGCTGTTCCCGCTG
  - ◦ Reverse primer: AAATCTGCTGCCGCCCTCTGTG
  - ◦ Sequencing primer: ACGTTCCCGAGCTCACCTTCCA
- Menin amino acids 154 to 255:
  - ◦ Forward primer: ACACGTGGGAGGGAAGGGATGG
  - ◦ Reverse primer: AGAAGCTCCAGCGAGTCGGTGT
  - ◦ Sequencing primer: GGAGGGAGTGTGGCCCATCACT
- Menin amino acids 281 to 309:
  - ◦ Forward primer: CTCCTCCCTGTTCCGTGGCTCA
  - ◦ Reverse primer: GCACATTGCGGTTGCGACAGTG
  - ◦ Sequencing primer: GCACATTGCGGTTGCGACAGTG
- Menin amino acids 310 to 355:
  - ◦ Forward primer: CCCTCAGCCCTGCCTTTTCTGC
  - ◦ Reverse primer: AGTCCTGGACGAGGGTGGTTGG
  - ◦ Sequencing primer: CTGGGATCTTCCTGTGGCCCCT
- Menin amino acids 356 to 400:
  - ◦ Forward primer: GCTGACCCAGACAGCATCAT
  - ◦ Reverse primer: GCCATCCCTAATCCCGTACA
  - ◦ Sequencing primer: GCCATCCCTAATCCCGTACA
- Menin amino acids 436 to 610:
  - ◦ Forward primer: GCCCACCTGCTGCGATTCTACG
  - ◦ Reverse primer: CTCCTACAAGCTGGGAGGAGCC
  - ◦ Sequencing primer: CTCCTACAAGCTGGGAGGAGCC

PCR amplification was performed using the Q5 High-Fidelity 2X Master Mix (New England BioLabs, Catalog number: M0492L) and the primers above. PCR clean-up was performed with the NucleoSpin DNA Clean-up kit (Takara Bio catalog #740609.250). Sanger sequencing was performed at Genewiz.

Amplicon sequencing was performed at Plasmidsaurus with long read sequencing with Oxford Nanopore Technologies. Allele frequencies were calculated using CRISPResso2^33^.

For *MEN1* mutation detection in vivo in Fig. 6A, B (CBAM44728), a tiered Sanger sequencing approach was taken wherein we first evaluated for M327, G331, and T349 mutations by sequencing an amplicon covering amino acids 310 to 355. If mutations were detected that approached a 50% peak by Sanger sequencing, suggestive of a heterozygous mutation, further *MEN1* sequencing was not performed. If negative, S160 and A247 mutations were then assessed by Sanger sequencing of an amplicon covering amino acids 154 to 256. If negative, D136 mutations were then assessed by Sanger sequencing of an amplicon covering amino acids 77 to 153 and D290 mutations were then assessed by Sanger sequencing of an amplicon covering amino acids 281 to 309.

For *MEN1* wild-type, M327I, and T349M variant allele frequency from RNA-sequencing in Fig. 6D (CPCT-0024), BAM files were analyzed and the number of mutant and wild-type reads at M327 and T349 were counted, with the variant allele frequency calculated as the number of reads for either wild-type or mutant reads divided by the total number of reads.

### RNA isolation, real-time quantitative PCR, and bulk RNA sequencing

RNA was isolated from human cells using the RNeasy Mini Kit (Qiagen). Complementary DNA (cDNA) synthesis for real-time quantitative PCR (RT-qPCR) was performed using the AzuraFlex cDNA Synthesis Kit (Azura Genomics). The TaqMan Gene Expression Assay (FAM, #4351370) targeting *MEIS1* (Hs00180020_m1) and *GAPDH* (Hs02786624_g1) was used for the RT-qPCR reactions performed on a ViiA 7 Real-Time PCR System (Applied Biosystems).

For bulk RNA sequencing, RNA quality was assessed using the Agilent TapeStation 4200 (Agilent Technologies). Library preparation was performed using the NEBNext Ultra II RNA Library Prep Kit for Illumina (New England Biolabs). Sequencing was performed on an Illumina NextSeq 550 system (Illumina), generating 37-bp paired-end reads.

### Statistics & Reproducibility

In this manuscript, statistical analyses were performed using GraphPad Prism 10 and MAGeCK, as detailed in the relevant Methods sections. For viability assays and TR-FRET displacement assays, dose-response curves were fit using a variable slope, four-parameter nonlinear regression model, and absolute IC_50_ or *K*_i_ values were derived as described above. No statistical method was used to predetermine sample size for these assays, however, sufficient data points and replicates were included to enable reliable curve fitting and estimation of IC_50_ values. In general, the large shifts in IC_50_ values we observed had non-overlapping 95% confidence intervals.

Exact replicate numbers are provided in the figure legends and corresponding Methods sections. In general, cell-based and biochemical assays were performed with at least two technical replicates, and key findings were confirmed in independent biological replicates (the number of technical and biological replicates is indicated in each figure in addition to being shown in the Source Data). However, Supplementary Fig. 4B+C and Main Fig. 3A and Supplementary Fig. 5A represent individual experiments with only technical replication to screen for cell lines harboring *MEN1* mutations that confer resistance to menin inhibitors. Given 80 cell lines were tested in competition assays (Supplementary Fig. 4B+C) and 40 cell lines in screening viability assays (Main Fig. 3A and Supplementary Fig. 5A), experimental replication was not feasible. Rather, cell lines demonstrating resistance were further validated as single guides in confirmatory viability assays (Main Fig. 3B and Supplementary Fig. 5B). Additionally, Supplementary Fig. 1A was performed with only technical replication to establish a dose for the subsequent CRISPR screen.

For in vivo PDX studies, cohort sizes were based on established experience with these leukemia xenograft models and practical considerations of engraftment, treatment response, and animal use. No statistical method was used to predetermine sample size.

For cell culture and biochemical experiments, samples were not randomized. For PDX experiments, mice were randomized to treatment after confirmation of engraftment, as described above. Blinding was not performed primarily due to feasibility. The majority of experiments involved in vitro assays and quantitative readouts where blinding is less of an issue. In vivo experiments were conducted using predefined objective endpoints, and animal health and humane endpoints were independently monitored by veterinary and technical staff in addition to the non-blinded study team.

Minimal data exclusion was applied and only in cases of clear technical error (e.g., pipetting or instrument error). All excluded data points are transparently indicated in the source data, either as blank cells or with a note indicating exclusion.

### Reporting summary

Further information on research design is available in the Nature Portfolio Reporting Summary linked to this article.

## Supplementary information

Supplementary Information

Descriptions of Additional Supplementary Files

Supplementary Data 1

Supplementary Data 2

Supplementary Data 3

Supplementary Data 4

Supplementary Data 5

Supplementary Data 6

Supplementary Data 7

Reporting Summary

Transparent Peer Review file

## Source data

Source Data

## Data Availability

The co-crystal structures of SNDX-5613 bound menin proteins have previously been uploaded to PDB with the following accession numbers Protein Data Bank (PDB) ID# 7UJ4 (wild-type) and PDB ID#8E90 (I327) and are analyzed in this study. Co-crystal structures for other inhibitors have also been uploaded to PDB with the following accession numbers, DS-1594 bound menin ID# 9WKW (wild-type) and ID# 9WKX (I327), DSP-5336 bound menin ID# 9WN9 (wild-type) and ID# 9WNA (I327), JNJ-6617 bound menin ID# 9WKU (wild-type) and ID# 9WKV (I327), and KO-539 bound menin ID# 9WNI (wild-type) and ID# 9WNJ (I327). RNA-sequencing data is available under the following GEO accession code: GSE294623. https://www.ncbi.nlm.nih.gov/geo/query/acc.cgi?acc=GSE294623. The CRISPR base editing screen data is available under the following GEO accession code: GSE294624. https://www.ncbi.nlm.nih.gov/geo/query/acc.cgi?acc=GSE294624. Source data are provided as a Source Data file (Source Data for all Main and Supplementary Figs.). Source data are provided with this paper.

## Code availability

No custom code for analysis was generated for this manuscript.
