# Supplementary material for: CRISPR base editor screening identifies spectrum of MEN1 mutations impacting menin inhibitors in clinical trials
Source: Nat Commun. 2026 May 9;17:6265. doi: 10.1038/s41467-026-72685-1 (PMC13377036; doi:10.1038/s41467-026-72685-1)
Supplement: Supplementary file 1 — Supplementary Information [file 41467_2026_72685_MOESM1_ESM.pdf]

Supplementary Fig. 1: *MEN1* base editor screen with clinical menin inhibitors

1A. Viability assays (CellTiter-Glo) in MV4;11 cells with Cas9 nickase fused to A>G base editor. The average of the Day 5 absolute IC_{75} and Day 9 absolute $IC_{97.5}$ was used in the CRISPR base editor screen. Data shown are from a single experiment with technical triplicates, with individual data points shown.

1B. Raw cell counts (top) and viability (bottom) during *MEN1* CRISPR base editor screen. Data shown are from a single experiment with technical triplicates, with individual data points shown. The raw cell count data was pooled to generate Main Figure 2B.

1C. sgRNA guide abundance comparing Day 21 to Day 0 from *MEN1* CRISPR base editor screen for each compound. Blue represents guides in MV4;11 C>T cells and red represents guides in MV4;11 A>G cells. sgRNAs are aligned by the predicted menin amino acid that is targeted. Each data point displays the mean of three technical replicates.

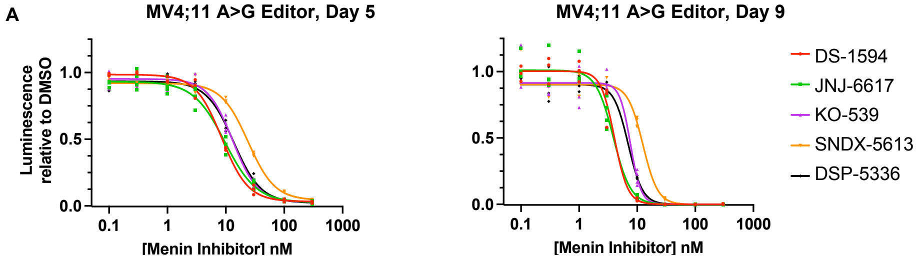

Average of Day 5 Absolute IC_{75} and Day 9 Absolute $IC_{97.5}$ = dose used in CRISPR base editor screen (nM)

DS-1594	JNJ-6617	KO-539	SNDX-5613	DSP-5336
13.8	17.3	23.4	51.9	25.2

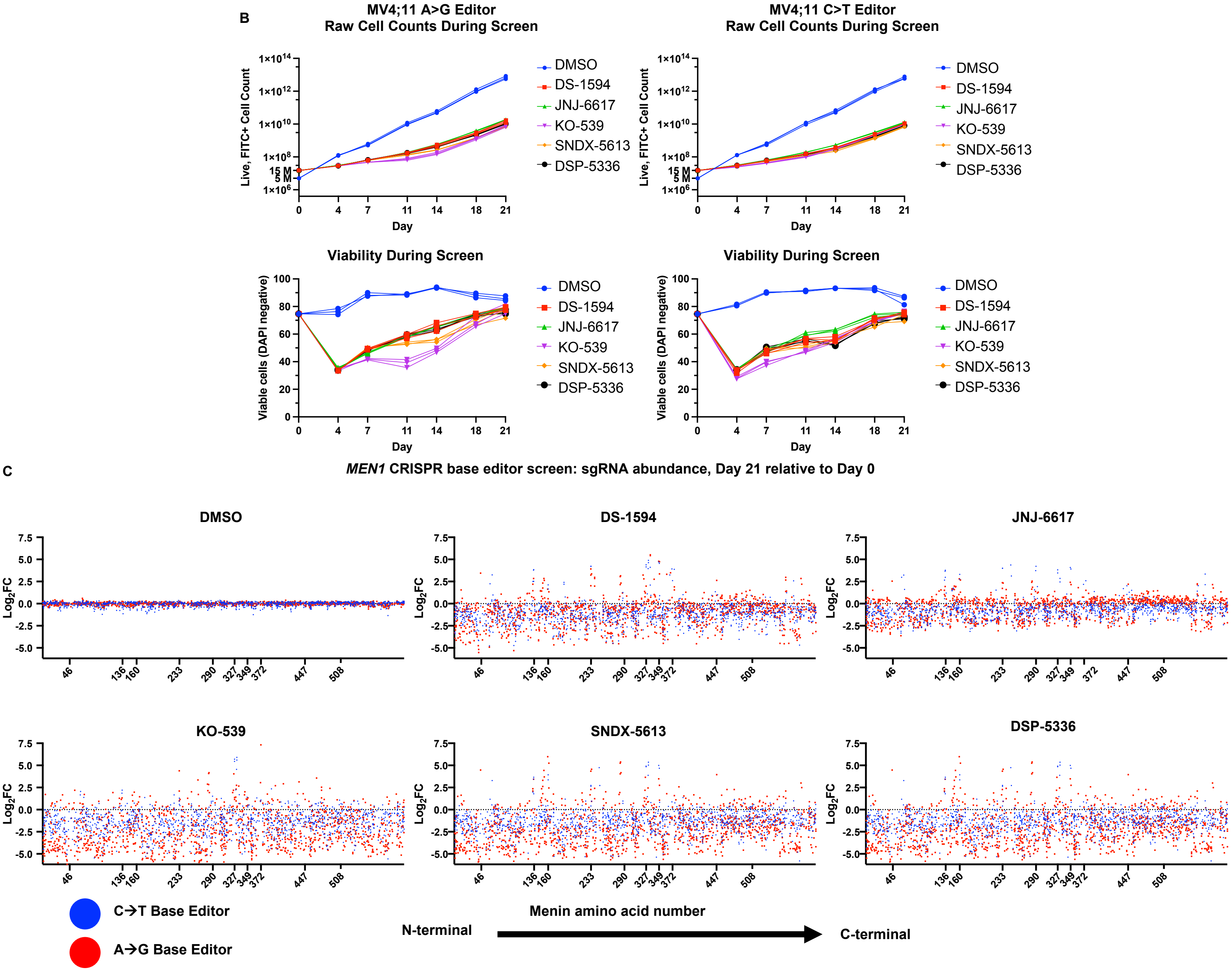

Supplementary Fig. 2: *MEN1* base editor screen with clinical menin inhibitors

2A. Performance of guides targeting intergenic controls (n = 124) and positive control guides targeting dependencies (n = 32, *RPA3*, *PCNA*, *DBR1*, *PLK1*, *RPL3*, *GAPDH*, *KIF11*, *PSMB1*, *EEF2*, *POLR2B*). Guides targeting *MEN1* shown as comparison. Log_2 Fold Change (FC) shown comparing guide abundance at Day 21 versus Day 0 of the screen in the DMSO arm of the screen. Each data point displays the mean of three technical replicates. Horizontal line displays the median value.

2B. Performance of guides in Supplementary Fig. 2A in each drug arm of the screen, relative to DMSO control. Horizontal line displays the median value.

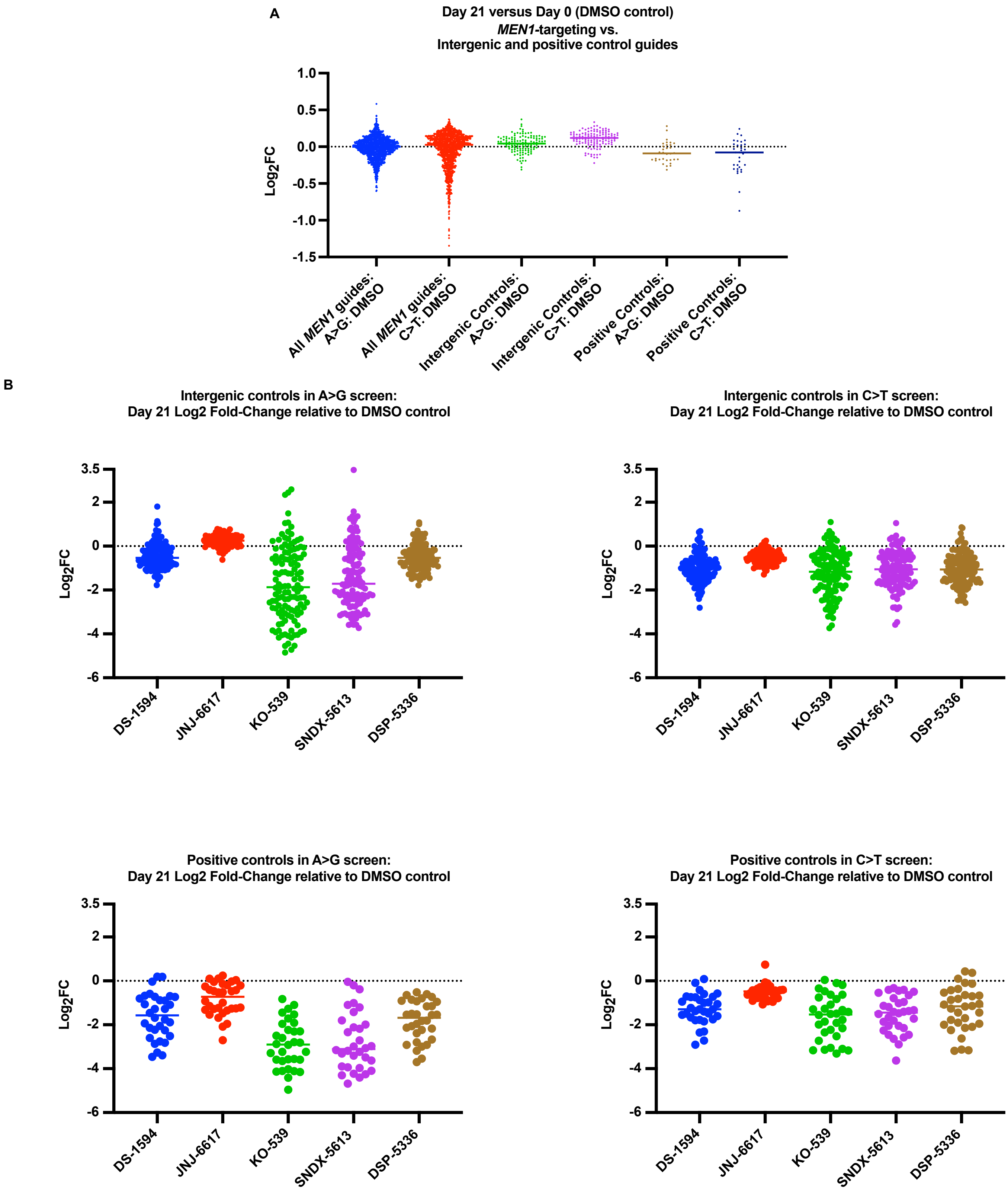

Supplementary Fig. 3: *MEN1* base editor screen with clinical menin inhibitors

3A+B: Scatter plots showing concordance between technical replicates in the MEN1 base editor screens. Plots show $\log_2$ fold-change values for individual sgRNAs across the three technical replicates in the A>G (**A**) and C>T (**B**) editing cell lines. Log_2 fold-change values were calculated as Day 21 minus Day 0 abundance relative to DMSO control. Each point represents a single sgRNA. The average of the technical replicates is shown in Main Figure 2C. Pearson correlation coefficients (r) are indicated in each panel.

A

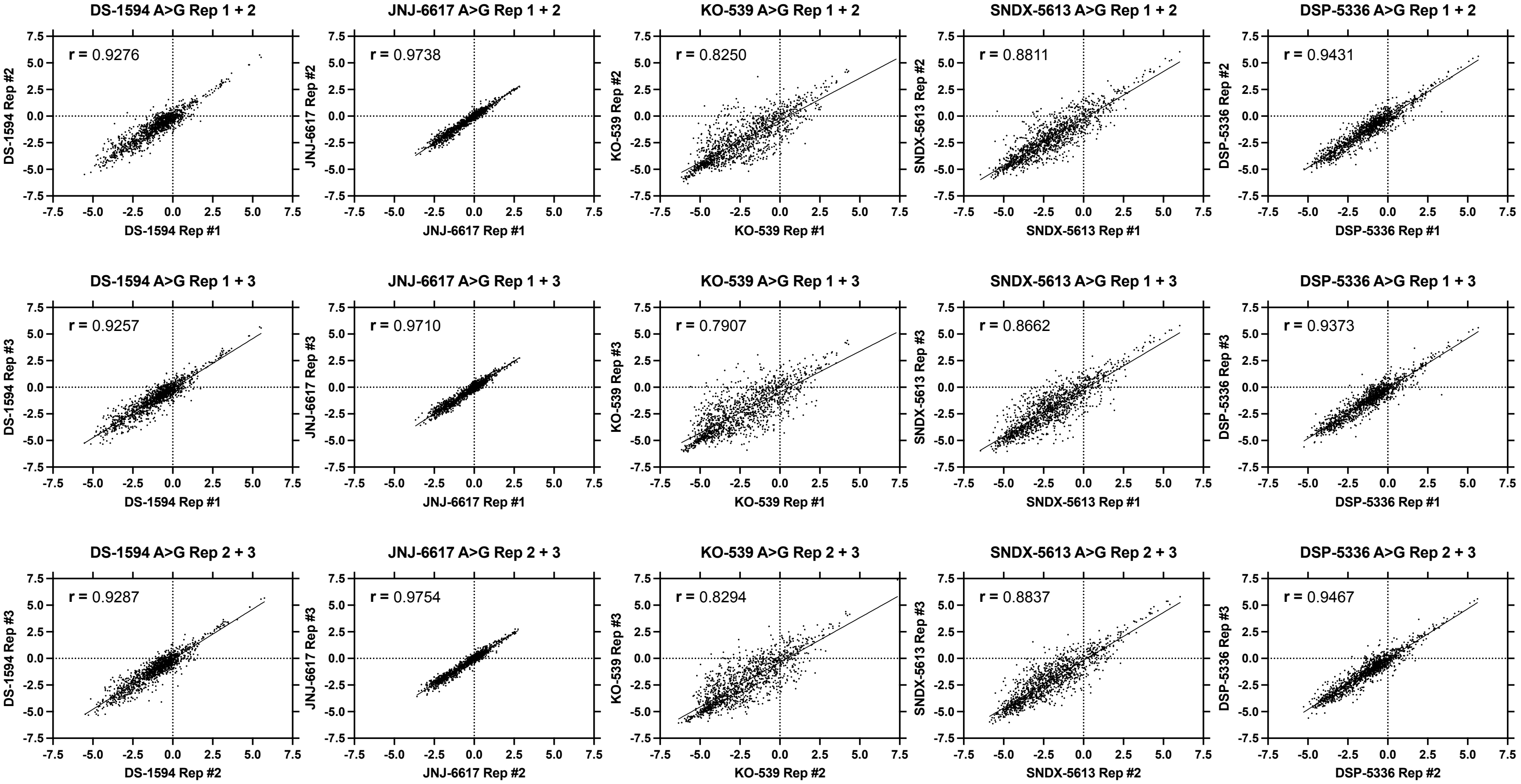

B

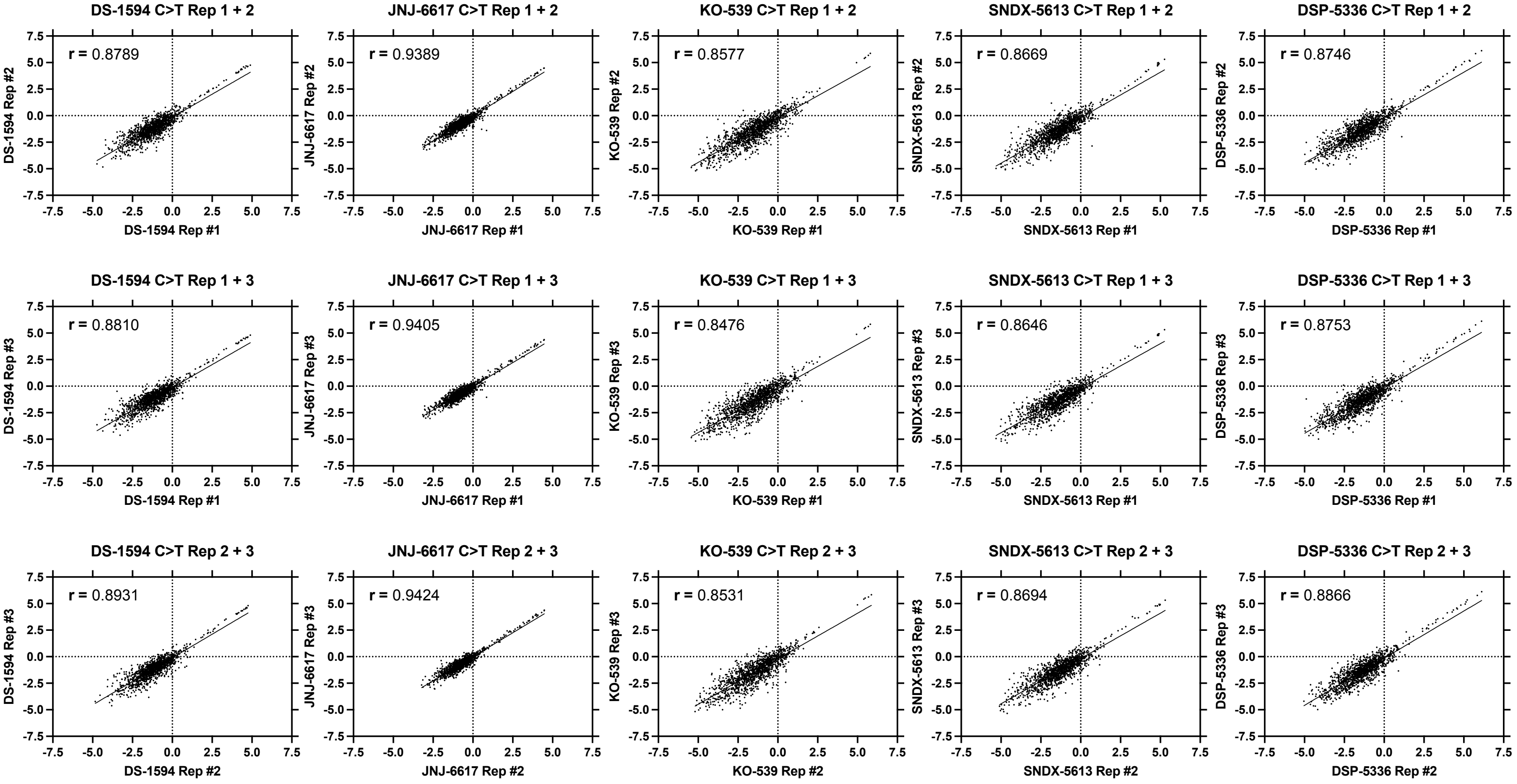

Supplementary Fig. 4: Single guide validation of *MEN1* base editor screen, competition assays

4A. Schematic of validation approach to *MEN1* CRISPR base editor screen.

4B. Example of two competition assays where cells were seeded on Day 0 with 20% cells containing sgRNAs targeting *MEN1* that were PE+ and 80% wild-type cells that were PE-. Cells were passaged, drug replenished, and PE% quantified at Day 4 and Day 9. Each condition seeded in three technical replicates. Data shown are from a single experiment with technical triplicates, with individual data points shown.

4C. Heat map displaying enrichment of sgRNAs targeting *MEN1* after 9 days of treatment after initial seeding of 20% PE+ *MEN1* mutant cells with 80% PE- *MEN1* wild-type cells. The heat map on the left is the results in MV4;11 cells with A>G base editing capacity and the right is the results in MV4;11 cells with C>T base editing capacity. The y-axis is annotated with the predicted amino acid edit from each sgRNA in the A>G and C>T cell lines respectively. The sgRNA sequence is shown on the left side of the figure. Each column represents DMSO control or a different menin inhibitor. Each condition seeded in at least three technical replicates from a single experiment with mean value for each condition displayed.

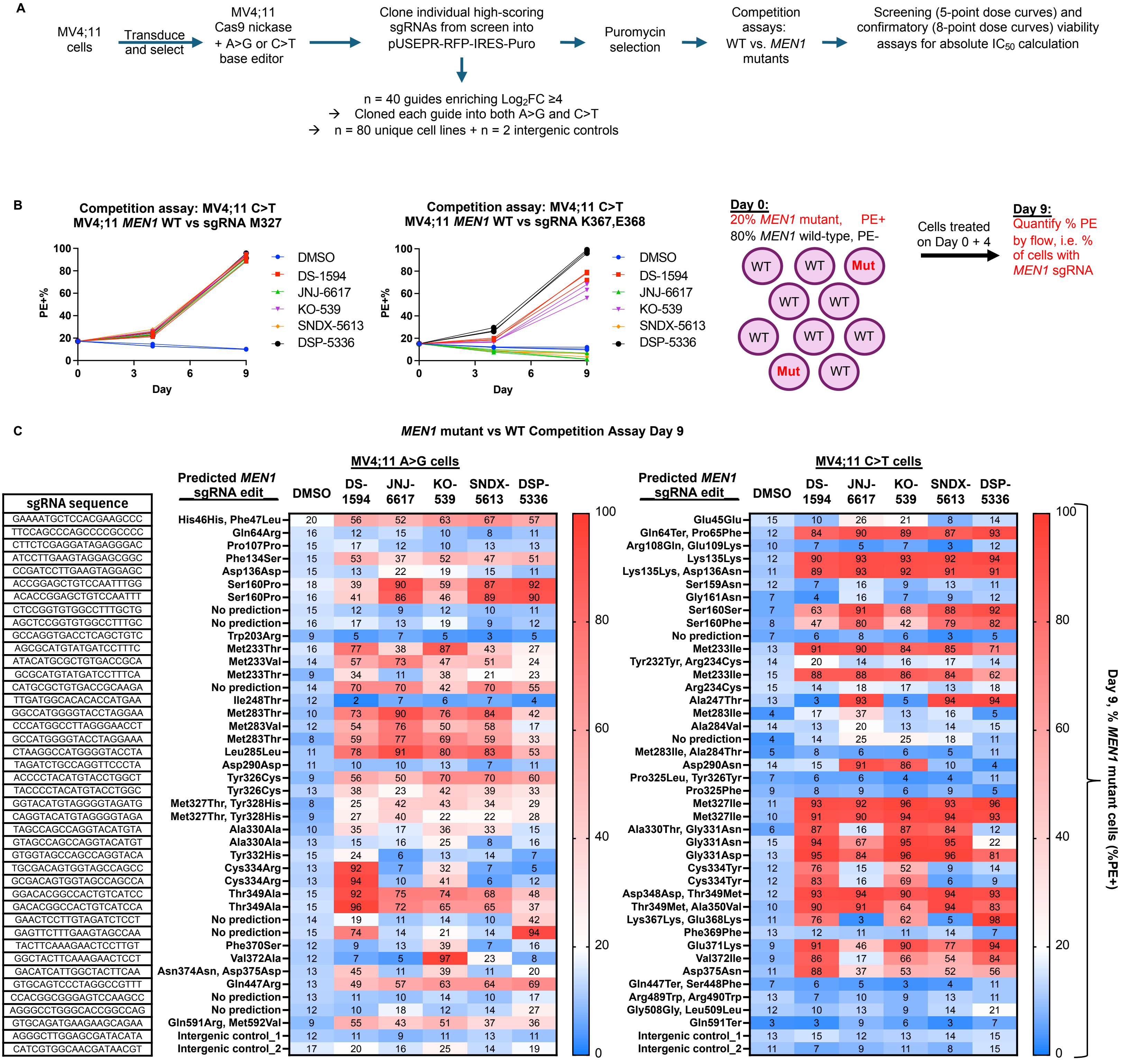

Supplementary Fig. 5: Single guide validation of *MEN1* base editor screen, viability assays

5A. Absolute IC_{50} table from screening viability (CellTiter-Glo) assays performed after 5 days of treatment in MV4;11 cells with *MEN1* mutants predicted for each sgRNA on the left and treated with each of 5 MIs in 5-point dose-response curves. Viability assay performed for each sgRNA that scored in screen (Log_2FC >4, Fig. 2C) and validated in *MEN1* mutant vs wild-type competition assays (≥3x enrichment for ≥2 sgRNAs or ≥4.25x enrichment for ≥1 sgRNA, Supplementary Fig. 4C). Each viability assay performed in three technical replicates with mean value for each condition displayed. This data was used to generate absolute IC_{50} heat maps in Main Figure 3A.

5B. Absolute IC_{50} table from confirmatory viability assays performed after 5 days of treatment in MV4;11 cells. Viability assay performed for each sgRNA from screening viability assay (Fig. 3A) where the absolute IC_{50} was shifted ≥ 10x for ≥1 MI or ≥7.5x for one MI relative to another. Each viability assay performed in three technical replicates and at least two biological replicates with mean value for each condition displayed. This data was used to generate absolute IC_{50} heat maps in Main Figure 3B. Source data contains Absolute IC_{50} values for biological replicates with 95% confidence intervals.

A

MV4;11 A>G or C>T cell lines
Screening Viability Assays
Absolute IC_{50} Table (nM)

sgRNA targeting amino acid residue	DS-1594	JNJ-6617	KO-539	SNDX-5613	DSP-5336
His46, Phe47	7	5	8	24	11
Gln64, Pro65	16	14	18	45	19
Lys135	25	16	33	115	60
Lys135, Asp136	20	19	26	65	32
Ser160	9	24	16	59	32
Ser160	7	16	14	49	23
Ser160	12	33	15	86	43
Ser160	8	14	10	26	20
Met233	13	8	23	28	11
Met233	20	10	18	23	8
Met233	27	19	26	71	22
Arg234	12	14	15	41	16
Ala247	2	43	3	183	59
Met283	8	9	12	31	11
Met283	7	8	9	19	8
Leu285	6	7	10	22	8
Asp290	4	18	20	11	3
Tyr326	4	6	8	18	8
Met327	126	62	>300	>300	90
Met327	94	33	271	>300	46
Ala330, Gly331	34	4	25	49	4
Gly331	>300	11	282	>300	8
Gly331	>300	14	>300	>300	9
Cys334	>300	7	13	18	11
Cys334	193	3	9	10	6
Cys334	18	6	20	9	8
Thr349	54	18	20	43	19
Asp348,Thr349	116	65	31	219	26
Thr349	53	15	18	44	19
Thr349, Ala350	52	17	14	78	14
Lys367, Glu368	17	2	14	6	>300
Phe369	10	8	12	18	34
Glu371	29	7	23	48	55
Val372	2	2	>300	17	7
Val372	18	7	15	31	19
Asp375	23	10	20	34	17
Gln447	10	14	16	35	17
Intergenic control	5	5	8	16	9
Intergenic control	5	5	7	14	8

0-2 | 2-4 | 4-8 | 8-16 | 16-32 | 32-64 | 64-128 | 128-256 | 256->300

B

MV4;11 A>G or C>T cell lines
Confirmatory Viability Assays
Absolute IC_{50} Table (nM)

sgRNA targeting amino acid residue	DS-1594	JNJ-6617	KO-539	SNDX-5613	DSP-5336
Ala247	3	56	4	117	38
Asp290	7	34	31	23	5
Met327	228	148	901	512	134
Met327	123	64	528	579	43
Ala330, Gly331	69	9	59	64	6
Gly331	505	17	412	490	10
Gly331	704	28	381	644	15
Cys334	158	7	15	15	8
Cys334	76	8	15	16	9
Asp348,Thr349	218	107	44	390	50
Lys367, Glu368	26	3	22	9	>1000
Val372	4	5	181	24	9
Intergenic control	5	6	9	17	9
Intergenic control	8	9	12	21	10

0-4 | 4-8 | 8-16 | 16-32 | 32-64 | 64-128 | 128-256 | 256-512 | 512->1000

Supplementary Fig. 6: Sanger sequencing of sgRNAs validated from screening viability assays

For each cell line tested in viability assays (Main Figure 3A+B), the column on the left in the table below lists the cell line the screening viability assay was performed in (MV4;11 A>G vs C>T), the sgRNA making the *MEN1* edit, the predicted *MEN1* edit, and whether the edit was predicted to occur with the A>G versus C>T editor. The middle column displays a picture of the chromatogram from Sanger sequencing. In the right column, the mutations inferred from Sanger sequencing are listed, as well as the shift in the Absolute IC_{50} from screening +/- confirmatory viability assays. In this supplementary figure, all sgRNAs in screening viability assays are depicted, with sgRNAs in confirmatory viability assays shown in Supplementary Fig. 7. The exception is a guide targeting Ala247Thr, which was used in confirmatory viability assays, but makes a homozygous edit by Sanger sequencing and thus amplicon sequencing was not performed.

Cell line tested, sgRNA, and predicted *MEN1* edit	Sanger Chromatogram	Sanger sequencing edit and Absolute IC_{50} shifts (Main Figures 3A+B)
**Cell line in viability assay:**<br>MV4;11 A>G<br>**sgRNA:**<br>GAAAATGCTCCACGAAGCCC<br>**Predicted *MEN1* edit (A>G):**<br>His46His, Phe47Leu	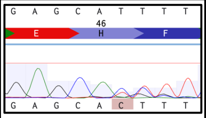	**Sanger sequencing inferred edit:**<br>His46His, Phe47 → Leu or Val<br>**Absolute IC_{50} shift in screening viability assay:**<br>DS-1594: 1.5x, JNJ-6617: 1.4x, KO-539: 1.3x, SNDX-5613: 2.0x, DSP-5336: 1.5x
**Cell line in viability assay:**<br>MV4;11 C>T<br>**sgRNA:**<br>TTCCAGCCCAGCCCCGCCCC<br>**Predicted *MEN1* edit (C>T):**<br>Gln64Ter, Pro65Phe	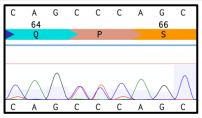	**Sanger sequencing inferred edit:**<br>Gln64Ter, Pro65 → Pro, Phe, Ser, or Leu<br>**Absolute IC_{50} shift in screening viability assay:**<br>DS-1594: 3.0x, JNJ-6617: 3.2x, KO-539: 2.4x, SNDX-5613: 2.7x, DSP-5336: 2.0x
**Cell line in viability assay:**<br>MV4;11 C>T<br>**sgRNA:**<br>ATCCTTGAAGTAGGAGCGGC<br>**Predicted *MEN1* edit (A>G):**<br>Lys135Lys (no C>T pred.)	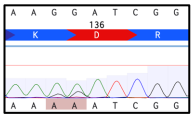	**Sanger sequencing inferred edit:**<br>Lys135Lys, Asp136Asn<br>**Absolute IC_{50} shift in screening viability assay:**<br>DS-1594: 5.7x, JNJ-6617: 3.5x, KO-539: 4.7x, SNDX-5613: 8.4x, DSP-5336: 8.4x
**Cell line in viability assay:**<br>MV4;11 C>T<br>**sgRNA:**<br>CCGATCCTTGAAGTAGGAGC<br>**Predicted *MEN1* edit (C>T):**<br>Lys135Lys, Asp136Asn	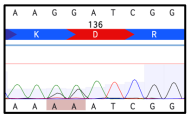	**Sanger sequencing inferred edit:**<br>Lys135Lys, Asp136Asn<br>**Absolute IC_{50} shift in screening viability assay:**<br>DS-1594: 4.1x, JNJ-6617: 3.7x, KO-539: 3.4x, SNDX-5613: 4.2x, DSP-5336: 3.4x
**Cell line in viability assay:**<br>MV4;11 A>G<br>**sgRNA:**<br>ACCGGAGCTGTCCAATTTGG<br>**Predicted *MEN1* edit (A>G):**<br>Ser160Pro	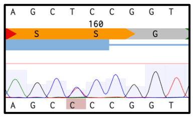	**Sanger sequencing inferred edit:**<br>Ser160Pro<br>**Absolute IC_{50} shift in screening viability assay:**<br>DS-1594: 1.5x, JNJ-6617: 3.5x, KO-539: 1.6x, SNDX-5613: 3.1x, DSP-5336: 2.9x
**Cell line in viability assay:**<br>MV4;11 A>G<br>**sgRNA:**<br>ACACCGGAGCTGTCCAATTT<br>**Predicted *MEN1* edit (A>G):**<br>Ser160Pro	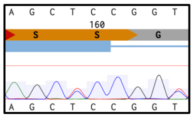	**Sanger sequencing inferred edit:**<br>Ser160Pro, Gly161Gly<br>**Absolute IC_{50} shift in screening viability assay:**<br>DS-1594: 1.1x, JNJ-6617: 2.3x, KO-539: 1.4x, SNDX-5613: 2.5x, DSP-5336: 2.1x
**Cell line in viability assay:**<br>MV4;11 C>T<br>**sgRNA:**<br>CTCCGGTGTGGCCTTTGCTG<br>**Predicted *MEN1* edit (C>T):**<br>Ser160Ser	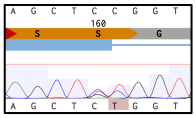	**Sanger sequencing inferred edit:**<br>Ser160 → Ser, Phe, or Cys<br>**Absolute IC_{50} shift in screening viability assay:**<br>DS-1594: 2.7x, JNJ-6617: 7.3x, KO-539: 2.2x, SNDX-5613: 6.3x, DSP-5336: 6.1x
**Cell line in viability assay:**<br>MV4;11 C>T<br>**sgRNA:**<br>AGCTCCGGTGTGGCCTTTGC<br>**Predicted *MEN1* edit (C>T):**<br>Ser160Phe	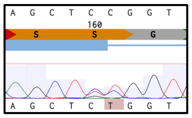	**Sanger sequencing inferred edit:**<br>Ser160 → Ser, Phe, or Cys<br>**Absolute IC_{50} shift in screening viability assay:**<br>DS-1594: 1.7x, JNJ-6617: 2.4x, KO-539: 1.2x, SNDX-5613: 1.8x, DSP-5336: 2.2x
**Cell line in viability assay:**<br>MV4;11 A>G<br>**sgRNA:**<br>AGCGCATGTATGATCCTTTC<br>**Predicted *MEN1* edit (A>G):**<br>Met233Thr	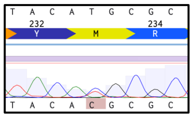	**Sanger sequencing inferred edit:**<br>Met233Thr<br>**Absolute IC_{50} shift in screening viability assay:**<br>DS-1594: 2.2x, JNJ-6617: 1.2x, KO-539: 2.4x, SNDX-5613: 1.5x, DSP-5336: 1.0x
**Cell line in viability assay:**<br>MV4;11 C>T<br>**sgRNA:**<br>AGCGCATGTATGATCCTTTC<br>**Predicted *MEN1* edit (C>T):**<br>Met233Ile	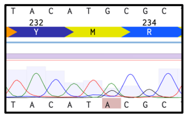	**Sanger sequencing inferred edit:**<br>Met233Ile<br>**Absolute IC_{50} shift in screening viability assay:**<br>DS-1594: 4.1x, JNJ-6617: 2.0x, KO-539: 2.4x, SNDX-5613: 1.7x, DSP-5336: 1.2x
**Cell line in viability assay:**<br>MV4;11 C>T<br>**sgRNA:**<br>GCGCATGTATGATCCTTTCA<br>**Predicted *MEN1* edit (C>T):**<br>Met233Ile	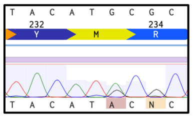	**Sanger sequencing inferred edit:**<br>Met233Ile<br>**Absolute IC_{50} shift in screening viability assay:**<br>DS-1594: 5.1x, JNJ-6617: 4.4x, KO-539: 3.5x, SNDX-5613: 4.2x, DSP-5336: 2.3x
**Cell line in viability assay:**<br>MV4;11 A>G<br>**sgRNA:**<br>CATGCGCTGTGACCGCAAGA<br>**Predicted *MEN1* edit (C>T):**<br>Arg234Cys (no A>G pred.)	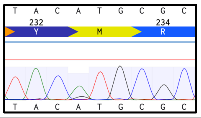	**Sanger sequencing inferred edit:**<br>Met233Val<br>**Absolute IC_{50} shift in screening viability assay:**<br>DS-1594: 2.0x, JNJ-6617: 2.0x, KO-539: 1.5x, SNDX-5613: 2.1x, DSP-5336: 1.4x
**Cell line in viability assay:**<br>MV4;11 C>T<br>**sgRNA:**<br>TTGATGGCACACACCATGAA<br>**Predicted *MEN1* edit (C>T):**<br>Ala247Thr	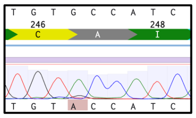	**Sanger sequencing inferred edit:**<br>Ala247Thr<br>**Absolute IC_{50} shift in screening / confirmatory viability assays:**<br>DS-1594: 0.4/0.3x, JNJ-6617: 10.0/6.3x, KO-539: 0.4/0.3x, SNDX-5613: 10.9/5.9x, DSP-5336: 6.3/3.8x
**Cell line in viability assay:**<br>MV4;11 A>G<br>**sgRNA:**<br>GGCCATGGGGTACCTAGGAA<br>**Predicted *MEN1* edit (A>G):**<br>Met283Thr	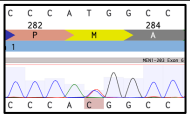	**Sanger sequencing inferred edit:**<br>Met283Thr<br>**Absolute IC_{50} shift in screening viability assay:**<br>DS-1594: 1.7x, JNJ-6617: 2.4x, KO-539: 1.9x, SNDX-5613: 2.5x, DSP-5336: 1.6x
**Cell line in viability assay:**<br>MV4;11 A>G<br>**sgRNA:**<br>GCCATGGGGTACCTAGGAAA<br>**Predicted *MEN1* edit (A>G):**<br>Met283Thr	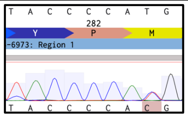	**Sanger sequencing inferred edit:**<br>Tyr281His, Met283Thr<br>**Absolute IC_{50} shift in screening viability assay:**<br>DS-1594: 1.1x, JNJ-6617: 1.2x, KO-539: 1.0x, SNDX-5613: 1.0x, DSP-5336: 0.8x
**Cell line in viability assay:**<br>MV4;11 A>G<br>**sgRNA:**<br>CTAAGGCCATGGGGTACCTA<br>**Predicted *MEN1* edit (A>G):**<br>Leu285Leu	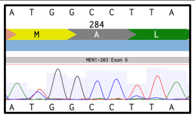	**Sanger sequencing inferred edit:**<br>Met283Thr, Leu285Leu<br>**Absolute IC_{50} shift in screening viability assay:**<br>DS-1594: 1.3x, JNJ-6617: 1.9x, KO-539: 1.6x, SNDX-5613: 1.8x, DSP-5336 1.1x
**Cell line in viability assay:**<br>MV4;11 A>G<br>**sgRNA:**<br>ACCCCTACATGTACCTGGCT<br>**Predicted *MEN1* edit (A>G):**<br>Tyr326Cys	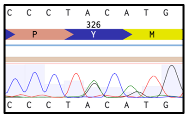	**Sanger sequencing inferred edit:**<br>Tyr326Cys, Met327Val<br>**Absolute IC_{50} shift in screening viability assay:**<br>DS-1594: 0.7x, JNJ-6617: 0.8x, KO-539: 0.8x, SNDX-5613: 0.9x, DSP-5336: 0.7x
**Cell line in viability assay:**<br>MV4;11 C>T<br>**sgRNA:**<br>GCGACAGTGGTAGCCAGCCA<br>**Predicted *MEN1* edit (C>T):**<br>Cys334Tyr	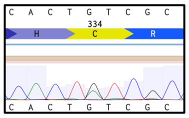	**Sanger sequencing inferred edit:**<br>Cys334Tyr<br>**Absolute IC_{50} shift in screening viability assay:**<br>DS-1594: 4.1x, JNJ-6617: 1.0x, KO-539: 2.5x, SNDX-5613: 0.6x, DSP-5336: 0.9x
**Cell line in viability assay:**<br>MV4;11 A>G<br>**sgRNA:**<br>GGACACGGCCACTGTCATCC<br>**Predicted *MEN1* edit (A>G):**<br>Thr349Ala	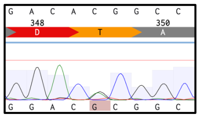	**Sanger sequencing inferred edit:**<br>Thr349Ala<br>**Absolute IC_{50} shift in screening viability assay:**<br>DS-1594: 8.8x, JNJ-6617: 2.7x, KO-539: 2.1x, SNDX-5613: 2.2x, DSP-5336: 1.8x
**Cell line in viability assay:**<br>MV4;11 A>G<br>**sgRNA:**<br>GACACGGCCACTGTCATCCA<br>**Predicted *MEN1* edit (A>G):**<br>Thr349Ala	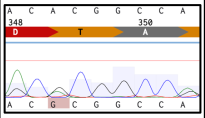	**Sanger sequencing inferred edit:**<br>Ala348Gly, Thr349Ala, Thr351Ala<br>**Absolute IC_{50} shift in screening viability assay:**<br>DS-1594: 8.8x, JNJ-6617: 2.2x, KO-539: 1.8x, SNDX-5613: 2.3x, DSP-5336: 1.7x
**Cell line in viability assay:**<br>MV4;11 C>T<br>**sgRNA:**<br>GACACGGCCACTGTCATCCA<br>**Predicted *MEN1* edit (C>T):**<br>Thr349Met, Ala350Val	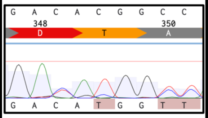	**Sanger sequencing inferred edit:**<br>Asp348Asp, Thr349Met, Ala350 → Ala or Val<br>**Absolute IC_{50} shift in screening viability assay:**<br>DS-1594: 11.8x, JNJ-6617: 3.8x, KO-539: 1.9x, SNDX-5613: 5.7x, DSP-5336: 2.0x
**Cell line in viability assay:**<br>MV4;11 A>G<br>**sgRNA:**<br>GAGTTCTTTGAAGTAGCCAA<br>**Predicted *MEN1* edit (A>G):**<br>Phe369Phe	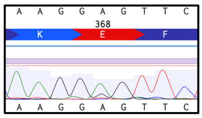	**Sanger sequencing inferred edit:**<br>Glu368Gly<br>**Absolute IC_{50} shift in screening viability assay:**<br>DS-1594: 1.6x, JNJ-6617: 1.1x, KO-539: 1.3x, SNDX-5613: 1.0x, DSP-5336: 3.2x
**Cell line in viability assay:**<br>MV4;11 C>T<br>**sgRNA:**<br>TACTTCAAAGAACTCCTTGT<br>**Predicted *MEN1* edit (A>G):**<br>Glu371Lys	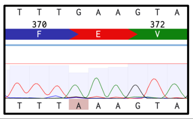	**Sanger sequencing inferred edit:**<br>Glu371Lys<br>**Absolute IC_{50} shift in screening viability assay:**<br>DS-1594: 5.5x, JNJ-6617: 1.6x, KO-539: 3.2x, SNDX-5613: 2.9x, DSP-5336: 5.9x
**Cell line in viability assay:**<br>MV4;11 C>T<br>**sgRNA:**<br>GGCTACTTCAAAGAACTCCT<br>**Predicted *MEN1* edit (C>T):**<br>Val372Ile	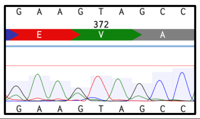	**Sanger sequencing inferred edit:**<br>Glu371Lys, Val372Ile, Ala373Thr<br>**Absolute IC_{50} shift in screening viability assay:**<br>DS-1594: 4.2x, JNJ-6617: 1.1x, KO-539: 1.9x, SNDX-5613: 2.2x, DSP-5336: 2.1x
**Cell line in viability assay:**<br>MV4;11 C>T<br>**sgRNA:**<br>GACATCATTGGCTACTTCAA<br>**Predicted *MEN1* edit (C>T):**<br>Asp375Asn	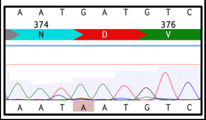	**Sanger sequencing inferred edit:**<br>Asn374Asn, Asp375Asn, Val376Ile<br>**Absolute IC_{50} shift in screening viability assay:**<br>DS-1594: 5.3x, JNJ-6617: 1.8x, KO-539: 2.5x, SNDX-5613: 2.4x, DSP-5336: 1.9x
**Cell line in viability assay:**<br>MV4;11 A>G<br>**sgRNA:**<br>GTGCAGTCCCTAGGCCGTTT<br>**Predicted *MEN1* edit (A>G):**<br>Gln447Arg	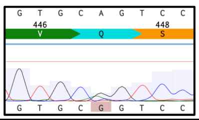	**Sanger sequencing inferred edit:**<br>Gln447Arg<br>**Absolute IC_{50} shift in screening viability assay:**<br>DS-1594: 1.7x, JNJ-6617: 2.1x, KO-539: 1.6x, SNDX-5613: 1.8x, DSP-5336: 1.5x

Supplementary Fig. 7: Sanger and amplicon sequencing of sgRNAs validated in confirmatory viability assays

For each cell line tested in viability assays (Main Figure 3A+B), the column on the left in the table below lists the cell line the screening viability assay was performed in (MV4;11 A>G vs C>T), the sgRNA making the *MEN1* edit, the predicted *MEN1* edit, and whether the edit was predicted to occur with the A>G versus C>T editor. The 2nd column displays a picture of the chromatogram from Sanger sequencing. In the 3rd column, the mutations inferred from Sanger sequencing are listed, as well as the shift in the Absolute IC_{50} from screening +/- confirmatory viability assays. In the 4th column, the contribution of alleles with ≥2% allelic frequency among all variants detected above a 0.2% calling threshold as determined by amplicon sequencing.

Cell line tested, sgRNA, and predicted *MEN1* edit	Sanger Chromatogram	Sanger sequencing edit and Absolute IC_{50} shifts (Main Figures 3A+B)	Amplicon Sequencing Results. Contribution of alleles with ≥2% allelic frequency among all variants detected above a 0.2% calling threshold is displayed. Outputs are from CRISPResso2. Mutant nucleotides are in red.
**Cell line in viability assay:**<br>MV4;11 C>T<br>**sgRNA:**<br>TAGATCTGCCAGGTTCCCTA<br>**Predicted *MEN1* edit (C>T):**<br>Asp290Asn	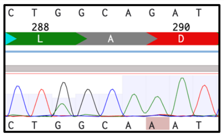	**Sanger sequencing inferred edit:**<br>Leu288Leu, Asp290Asn<br><br>**Absolute IC_{50} shift in screening / confirm. viability assays:**<br>DS-1594: 0.8/0.9x, JNJ-6617: 4.2/4.0x, KO-539: 2.8/3.0x, SNDX-5613: 0.7/1.2x, DSP-5336: 0.3/0.5x	GCCTTAGGGAACCTGGCAAATCTAGAGGAGCTGG --> D290N (36%)<br>GCCTTAGGGAACCTAGCAAATCTAGAGGAGCTGG --> L288L, D290N (34%)<br>GCCTTAGGGAACCTGGCAGATCTAGAGGAGCTGG --> WT (5%)<br>GCCTTAGGGAACCTAACAAATCTAGAGGAGCTGG --> L288L, A289T, D290N (5%)<br>GCCTTAGGGAACCTGGCACATCTAGAGGAGCTGG --> D290H (5%)<br>GCCTTAGGGAACCTGGCATATCTAGAGGAGCTGG --> D290Y (3%)
**Cell line in viability assay:**<br>MV4;11 C>T<br>**sgRNA:**<br>GGTACATGTAGGGGTAGATG<br>**Predicted *MEN1* edit (C>T):**<br>Met327Ile	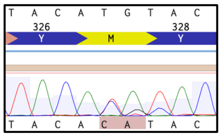	**Sanger sequencing inferred edit:**<br>Met327 → Ile or Thr; Tyr328His<br><br>**Absolute IC_{50} shift in screening / confirm. viability assays:**<br>DS-1594: >17.5/31.8x, JNJ-6617: 14.3/17.6x, KO-539: >17.5/>75x, SNDX-5613: >17.5/27.5x, DSP-5336: 9.7/14.7x	AACACATCTACCCCTACACATACCTGGCTGGCTA --> M327T (43%)<br>AACACATCTACCCCTACATACACCTGGCTGGCTA --> M327I, Y328H (16%)<br>AACACATCTACCCCTACATATACCTGGCTGGCTA --> M327I (14%)<br>AACACATCTACCCCTACATGTACCTGGCTGGCTA --> WT (13%)<br>AACACATCTACCCCTACACACACCTGGCTGGCTA --> M327T, Y328H (7%)<br>AACACATCTACCCCTACATTTACCTGGCTGGCTA --> M327I (3%)
**Cell line in viability assay:**<br>MV4;11 C>T<br>**sgRNA:**<br>CAGGTACATGTAGGGGTAGA<br>**Predicted *MEN1* edit (C>T):**<br>Met327Ile	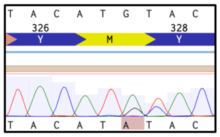	**Sanger sequencing inferred edit:**<br>Met327Ile, Tyr328His<br><br>**Absolute IC_{50} shift in screening / confirm. viability assays:**<br>DS-1594: >17.5/17.2x, JNJ-6617: 7.3/7.6x, KO-539: >17.5/50.3x, SNDX-5613: >17.5/31.1x, DSP-5336: 6.1/4.7x	CACATCTACCCCTACATATACCTGGCTGGCTACC --> M327I (38%)<br>CACATCTACCCCTACATACACCTGGCTGGCTACC --> M327I, Y328H (28%)<br>CACATCTACCCCTACATGTACCTGGCTGGCTACC --> WT (21%)<br>CACATCTACCCCTACACATACCTGGCTGGCTACC --> M327T (3%)<br>CACATCTACCCCTACATGCACCTGGCTGGCTACC --> Y328H (2%)<br>CACATCTACCCCTACATTTACCTGGCTGGCTACC --> M327I (2%)<br>CACATCTACCCCTACATATACCTAGCTGGCTACC --> M327I, L329L (2%)
**Cell line in viability assay:**<br>MV4;11 C>T<br>**sgRNA:**<br>TAGCCAGCCAGGTACATGTA<br>**Predicted *MEN1* edit (C>T):**<br>Ala330Thr, Gly331Asn	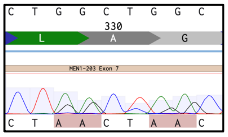	**Sanger sequencing inferred edit:**<br>Leu329Leu, Ala330Thr, Gly331 → Asn, Ser, or Asp<br><br>**Absolute IC_{50} shift in screening / confirm. viability assays:**<br>DS-1594: 7.1/8.8x, JNJ-6617: 0.8x/1.0x, KO-539: 3.4/5.7x, SNDX-5613: 3.6/3.2x, 0.6/0.6x	CCCCTACATGTACCTAACTAACTACCACTGTCGC --> L329L, A330T, G331N (30%)<br>CCCCTACATGTACCTAACCAACTACCACTGTCGC --> L329L, A330T, G331N (20%)<br>CCCCTACATGTACCTGGCTGGCTACCACTGTCGC --> WT (16%)<br>CCCCTACATGTACCTAGCTAACTACCACTGTCGC --> L329L, G331N (14%)<br>CCCCTACATGTACCTAGCTAGCTACCACTGTCGC --> L329L, G331S (3%)<br>CCCCTACATGTACCTAACTAGCTACCACTGTCGC --> L329L, A330T, G331S (3%)<br>CCCCTACATGTACCTAGCCAACTACCACTGTCGC --> L329L, A330A, G331N (2%)
**Cell line in viability assay:**<br>MV4;11 C>T<br>**sgRNA:**<br>GTAGCCAGCCAGGTACATGT<br>**Predicted *MEN1* edit (C>T):**<br>Gly331Asn	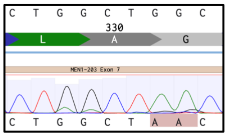	**Sanger sequencing inferred edit:**<br>Leu329Leu, Ala330Thr, Gly331Asn<br><br>**Absolute IC_{50} shift in screening / confirm. viability assays:**<br>DS-1594: >17.5/64.2x, JNJ-6617: 2.1/1.9x, KO-539: >17.5/39.6x, SNDX-5613: >17.5/24.7x, DSP-5336: 1.0/1.0x	CCCTACATGTACCTGGCTAACTACCACTGTCGCA --> G331N (44%)<br>CCCTACATGTACCTAGCTAACTACCACTGTCGCA --> L329L, G331N (15%)<br>CCCTACATGTACCTGGCTAACCACCACTGTCGCA --> G331N, Y332H (8%)<br>CCCTACATGTACCTGGCTGGCTACCACTGTCGCA --> WT (8%)<br>CCCTACATGTACCTGACTAACTACCACTGTCGCA --> A330T, G331N (7%)<br>CCCTACATGTACCTGGCTACCTACCACTGTCGCA --> G331T (4%)<br>CCCTACATGTACCTAACTAACTACCACTGTCGCA --> L329L, A330T, G331N (4%)<br>CCCTACATGTACCTGGCCAACTACCACTGTCGCA --> A330A, G331N (3%)
**Cell line in viability assay:**<br>MV4;11 C>T<br>**sgRNA:**<br>GTGGTAGCCAGCCAGGTACA<br>**Predicted *MEN1* edit (C>T):**<br>Gly331Asp	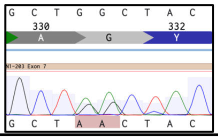	**Sanger sequencing inferred edit:**<br>Gly331 → Asn, Ser, or Asp; Tyr332His<br><br>**Absolute IC_{50} shift in screening / confirm. viability assays:**<br>DS-1594: >17.5/>75x, JNJ-6617: 3.1/3.2x, KO-539: >17.5/36.6x, SNDX-5613: >17.5/32.5x, DSP-5336: 1.3/1.5x	TACATGTACCTGGCTAACTACCACTGTCGCAACC --> G331N (46%)<br>TACATGTACCTGGCTGGCTACCACTGTCGCAACC --> WT (20%)<br>TACATGTACCTGGCTAACCACCACTGTCGCAACC --> G331N, Y332H (13%)<br>TACATGTACCTGGCTGACTACCACTGTCGCAACC --> G331D (7%)<br>TACATGTACCTGGCTAGCTACCACTGTCGCAACC --> G331S (5%)<br>TACATGTACCTGGCTGACCACCACTGTCGCAACC --> G331D, Y332H (3%)
**Cell line in viability assay:**<br>MV4;11 A>G<br>**sgRNA:**<br>TGCGACAGTGGTAGCCAGCC<br>**Predicted *MEN1* edit (A>G):**<br>Cys334Arg	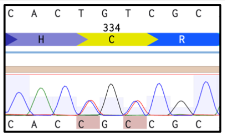	**Sanger sequencing inferred edit:**<br>Cys334Arg<br><br>**Absolute IC_{50} shift in screening / confirm. viability assays:**<br>DS-1594: >17.5/14.3x, JNJ-6617: 1.0/1.3x, KO-539: 1.4/1.7x, SNDX-5613: 0.9/0.9x, DSP-5336: 1.0x/1.0x	ACCTGGCTGGCTACCACTGTCGCAACCGCAATGT --> WT (49%)<br>ACCTGGCTGGCTACCACCGCCGCAACCGCAATGT --> C334R (46%)<br>ACCTGGCTGGCTACCACCGTCGCAACCGCAATGT --> C334R (5%)
**Cell line in viability assay:**<br>MV4;11 A>G<br>**sgRNA:**<br>GCGACAGTGGTAGCCAGCC<br>A<br>**Predicted *MEN1* edit (A>G):**<br>Cys334Arg	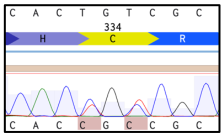	**Sanger sequencing inferred edit:**<br>Cys334Arg<br><br>**Absolute IC_{50} shift in screening / confirm. viability assays:**<br>DS-1594: >17.5/29.9x, JNJ-6617: 0.8/1.2x, KO-539: 1.5/1.7x, SNDX-5613: 0.8/0.9x, DSP-5336: 0.9/0.8x	TACCTGGCTGGCTACCACTGTCGCAACCGCAATG --> WT (39%)<br>TACCTGGCTGGCTACCACCGCCGCAACCGCAATG --> C334R (39%)<br>TACCTGGCTGGCTACCACCGTCGCAACCGCAATG --> C334R (21%)
**Cell line in viability assay:**<br>MV4;11 C>T<br>**sgRNA:**<br>GGACACGGCCACTGTCATCC<br>**Predicted *MEN1* edit (C>T):**<br>Asp348Asp, Thr349Met	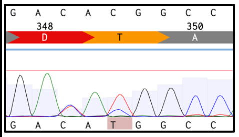	**Sanger sequencing inferred edit:**<br>Asp348Asp, Thr349Met, Ala350 → Ala or Val<br><br>**Absolute IC_{50} shift in screening / confirm. viability assays:**<br>DS-1594: >17.5/21.2x, JNJ-6617: 14.4/9.9x, KO-539: 4.5/3.2x, SNDX-5613: 16.1/13.8x, DSP-5336: 3.7/3.9x	GGGCGGATATGGCCACTGTCATCCAGGAGTGAGG --> D348D, T349M (33%)<br>GGGCGGACATGGCCACTGTCATCCAGGAGTGAGG --> T349M (24%)<br>GGGCGGACACGGCCACTGTCATCCAGGAGTGAGG --> WT (19%)<br>GGGCGGACATGGCTACTGTCATCCAGGAGTGAGG --> T349M, A350A (4%)<br>GGGCGGACATGGTCACTGTCATCCAGGAGTGAGG --> T349M, A350V (3%)<br>GGGCGGATACGGCCACTGTCATCCAGGAGTGAGG --> D348D (3%)<br>GGGCGGATATGGCTACTGTCATCCAGGAGTGAGG --> D348D, T349M, A350A (3%)<br>GGGCGGATATGGTCACTGTCATCCAGGAGTGAGG --> D348D, T349M, A350V (2%)<br>GGGCGGACGTGGCCACTGTCATCCAGGAGTGAGG --> T349V
**Cell line in viability assay:**<br>MV4;11 C>T<br>**sgRNA:**<br>GAACTCCTTGTAGATCTCCT<br>**Predicted *MEN1* edit (C>T):**<br>Lys367Lys, Glu368Lys	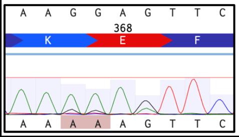	**Sanger sequencing inferred edit:**<br>Lys367Lys, Glu368 → Glu or Lys<br><br>**Absolute IC_{50} shift in screening / confirm. viability assays:**<br>DS-1594: 3.2/2.5x, JNJ-6617: 0.4/0.3x, KO-539: 1.9/1.6x, SNDX-5613: 0.3/0.3x, DSP-5336: >17.5/>75x	GACGAGGAGATCTACAAAAAGTTCTTTGAAGTAG --> K367K, E368K (49%)<br>GACGAGGAGATCTACAAAAAATTCTTTGAAGTAG --> K367K, E368K (26%)<br>GACGAGGAGATCTACAAGGAGTTCTTTGAAGTAG --> WT (16%)<br>GACGAGGAGATCTACAAGAAGTTCTTTGAAGTAG --> E368K (2%)
**Cell line in viability assay:**<br>MV4;11 A>G<br>**sgRNA:**<br>GGCTACTTCAAAGAACTCCT<br>**Predicted *MEN1* edit (A>G):**<br>Val372Ala	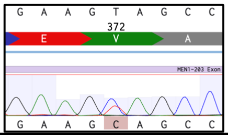	**Sanger sequencing inferred edit:**<br>Val372Ala<br><br>**Absolute IC_{50} shift in screening viability assay:**<br>DS-1594: 0.5/0.7x, JNJ-6617 0.6/0.8x, KO-539: >17.5/20.5x, SNDX-5613: 1.4/1.4x, DSP-5336: 1.0/1.0x	TACAAGGAGTTCTTTGAAGCAGCCAATGATGTCA --> V372A (57%)<br>TACAAGGAGTTCTTTGAAGTAGCCAATGATGTCA --> WT (38%)<br>TACAAGGAGTTCTTCGAAGCAGCCAATGATGTCA --> F370F, V372A (5%)

Supplementary Fig. 8: Single guide validation of *MEN1* base editor screen

8A. Quantitative PCR analysis of *MEIS1* expression following 72 hours of treatment in MV4;11 wild-type and *MEN1* mutant (E368K or V372A) cell lines. Data represent three independent experiments, each performed with technical triplicates, with individual replicates shown. Horizontal lines indicate the median. Statistical analysis was performed using two-way ANOVA (genotype × treatment interaction; for example, E368K comparison: interaction $F(4, 75) = 153$, $p < 0.0001$), followed by Sidak's multiple comparisons test comparing WT and mutant cells within each treatment condition. Adjusted p values are indicated as ns, *$p < 0.05$, **$p < 0.01$, ***$p < 0.001$, ****$p < 0.0001$.

8B-G. Cell counts over time in MV4;11 C>T wild-type versus **B.** C>T cell line with sgRNA targeting Asp290 **E.** C>T cell line with sgRNA targeting Ala330, Gly331. n ≥ 4 technical replicates per data point from a single experiment. **C+F.** After 20 days of treatment, cell pellets were harvested and DNA extracted and amplicon sequencing performed, with allelic frequency at Asp290 (**C**) and G331 (**F**) displayed. **D+G.** Viability assays in wild-type cells, cells treated with JNJ-6617 for 20 days, or cells treated with KO-539 for 20 days. Viability assays performed with 8-point dose response curve, ranging from 0.3 nM to 1000 nM, and in at least technical duplicates with three biological replicates (representative experiment shown). Each dose point displays each technical replicate. Absolute IC_{50} shifts relative to *MEN1* wild-type cells shown. Of note, dose response curves with prior JNJ-6617 treatment contained in Supplementary Fig. 8D+G are also presented in Main Figures 3F +H, and are presented here again for ease of comparison. Source data contains Absolute IC_{50} values for biological replicates with 95% confidence intervals, as well as statistical testing.

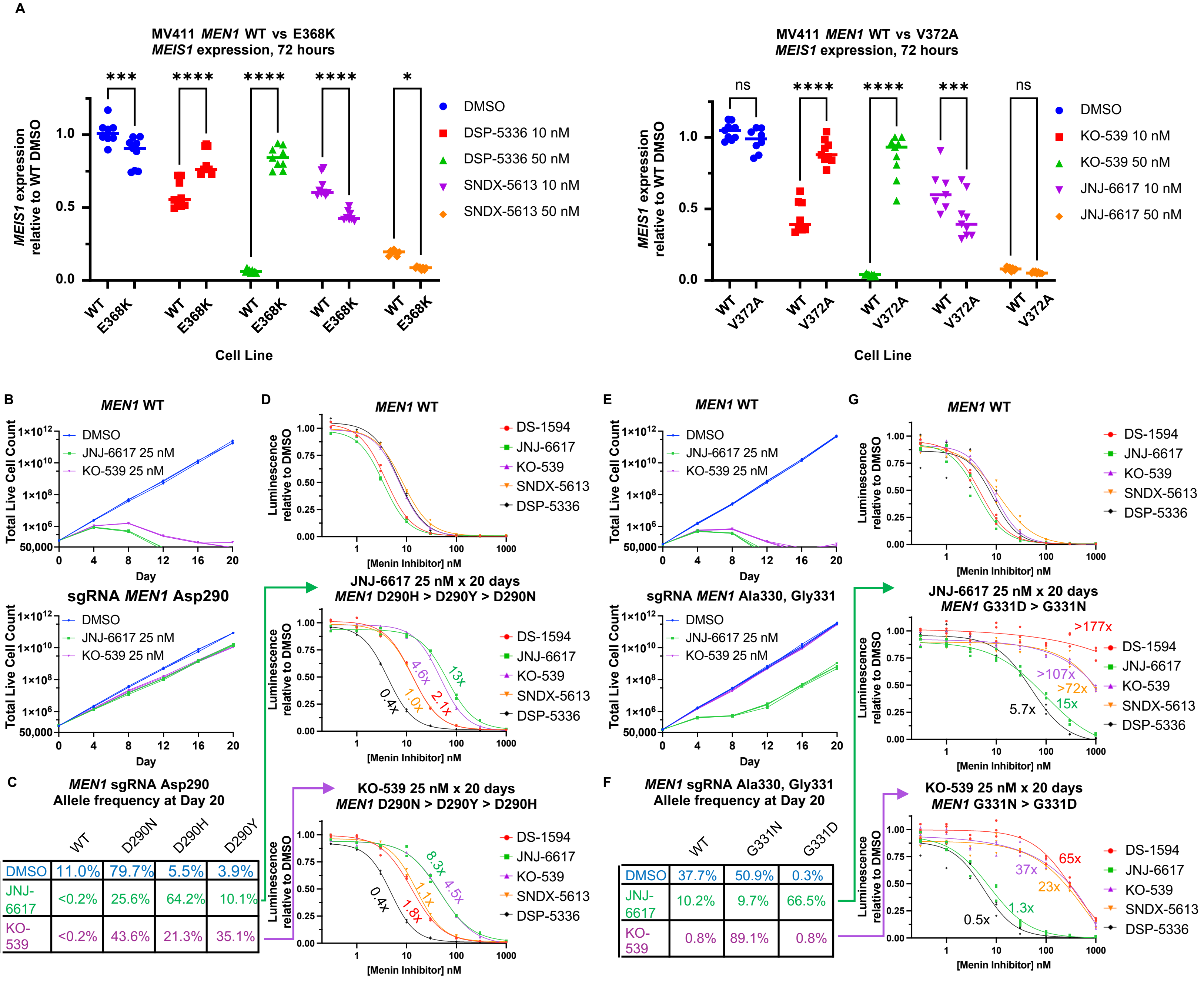

Supplementary Fig. 9: Biochemical and structural insights into *MEN1* mutation effects

9A-C: Data shown in Main Figure 4A and Supplementary Figure 9A-C are derived from the same experiments but presented in different formats for clarity.

9A. Representative dose-response curves from TR-FRET displacement assays measuring inhibition of FITC-conjugated KMT2A or KMT2B peptide binding to menin. Top panels show KMT2A-FITC displacement assays using wild-type and mutant menin proteins (M327I, T349M, G331R). Bottom panel shows KMT2B-FITC probe displacement assays using wild-type and mutant menin proteins (C334R, E368K, and V372A). Curves and IC_{50} values (nM) shown in legend boxes represent averages from $n \geq 6$ independent experimental replicates (top) or $n = 3$ independent experiments (bottom), each performed with two technical replicates per concentration. Individual replicate IC_{50} values are displayed in **9B**. IC_{50} estimates were used to calculate K_i values for Main Figure 4A and Supplementary Figure **9C**. The dose-response curve for wild-type menin (top left) is the same dataset as that shown in Main Figure 1C.

9B. Dot plots of IC_{50} values derived from TR-FRET displacement assays shown in **9A** for five menin inhibitors tested against recombinant wild-type (WT) and mutant menin proteins (M327I, G331R, T349M, C334R, E368K, V372A). Top panels show absolute IC_{50} values, and bottom panels show fold-change values expressed as the ratio of mutant (MT) IC_{50} to wild-type (WT) IC_{50}. Each point represents an independent experiment. Horizontal lines indicate median values.

9C. Dot plots depicting log10-transformed Ki values derived from KMT2A- or KMT2B-FITC TR-FRET displacement assays (see 9A). Each point represents an independent experiment, with horizontal lines indicating the median. Statistical analysis was performed using ordinary one-way ANOVA on log10-transformed values (for example, DS-1594: $F(3, 8) = 50.1$, $p < 0.0001$), followed by Dunnett's multiple comparisons test relative to WT. Adjusted p values are indicated as ns, $*p < 0.05$, $**p < 0.01$, $***p < 0.001$, $****p < 0.0001$. Data are the same as in Main Fig. 4A, separated by KMT2A- versus KMT2B-FITC probe to enable statistical comparison.

Supplementary Figure 9

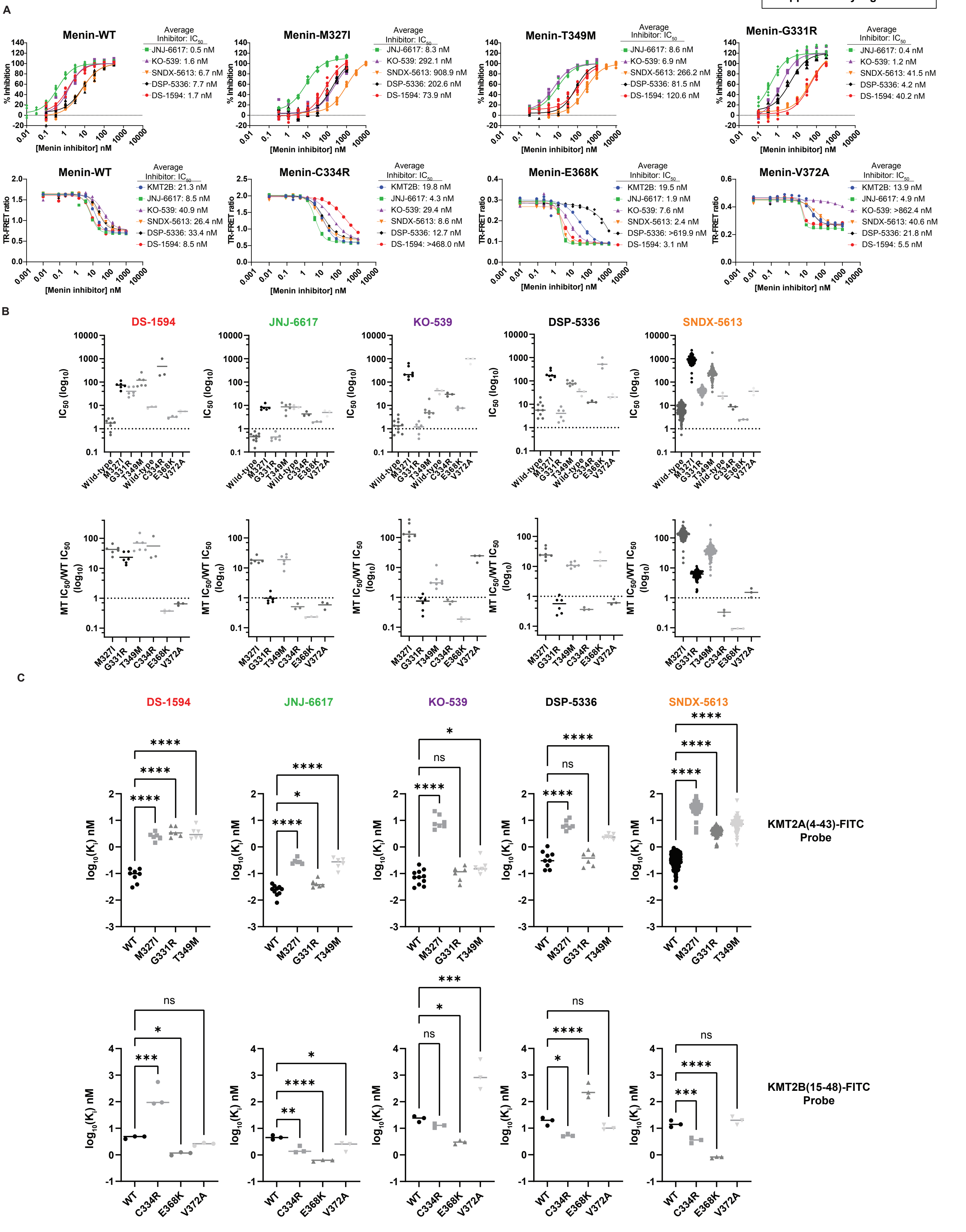

Supplementary Fig. 10: Biochemical and structural insights into *MEN1* mutation effects

10A. Co-crystal structure overlays of menin inhibitors bound to wild-type menin (M327) and mutant menin (I327). **10B-F**. Co-crystal structure overlays of menin inhibitors bound to wild-type menin (M327) and mutant menin (I327) either **B.** JNJ-6617, **C.** DSP-5336, **D.** DS-1594, **E.** KO-539, or **F.** SNDX-5613, with quantitative coloring reflecting the magnitude of positional changes due to the M327I mutation mapped onto the atoms (excluding hydrogens) of each inhibitor. Mapping is only displayed on inhibitors bound to the I327 mutant structures, and the wild-type bound position is translucently colored in grey to display the positional changes. Only amino acid side chains I327 (magenta) and M327 (grey) from the corresponding proteins are displayed. DS-1594 bound menin PDB ID# 9WKW (wild-type) and PDB ID# 9WKX (I327), DSP-5336 bound menin PDB ID# 9WN9 (wild-type) and PDB ID# 9WNA (I327), JNJ-6617 bound menin PDB ID# 9WKU (wild-type) and PDB ID# 9WKV (I327), and KO-539 bound menin PDB ID# 9WNI (wild-type) and PDB ID# 9WNJ (I327). **10G.** Co-crystal structure of KMT2A peptide bound to menin (PDB entry: 3U88).[23]

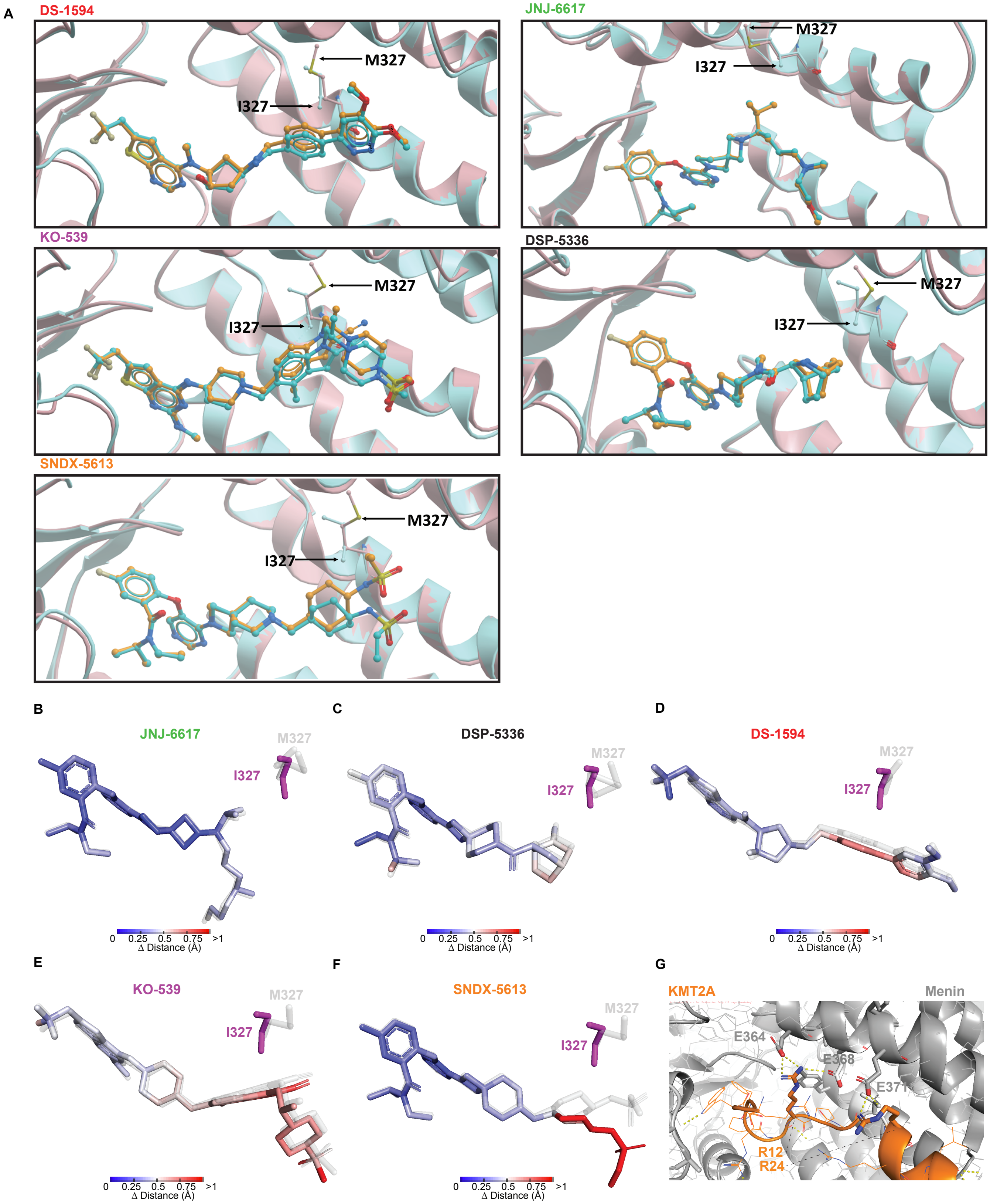

Supplementary Fig. 11: De novo *MEN1* mutation emergence *in vitro* corroborates screen 11A+B. This data corresponds to Main Figure 5A and 5D and contributes 10 of the 22 experimental replicates for each of the single-agent menin inhibitor experiments shown in those figures. In these two independent experiments (A+B), 12.5 million MOLM13 cells were seeded per replicate, with 5 flasks per experiment treated with JNJ-6617 25 nM, 5 flasks per experiment treated with DSP-5336 25 nM, and 6 flasks per experiment treated with both JNJ-6617 25 nM and DSP-5336 25 nM. Individual replicates were seeded on Day 0. Live cell counts tracked over time. Individual replicates from each experiment are shown.

A

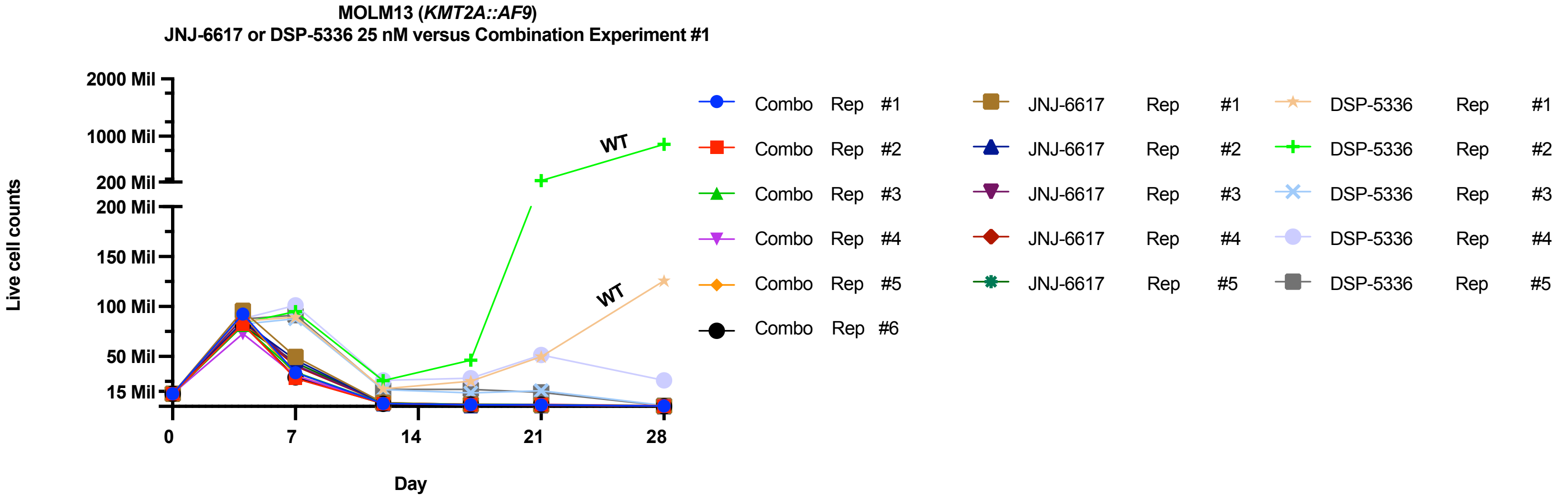

B

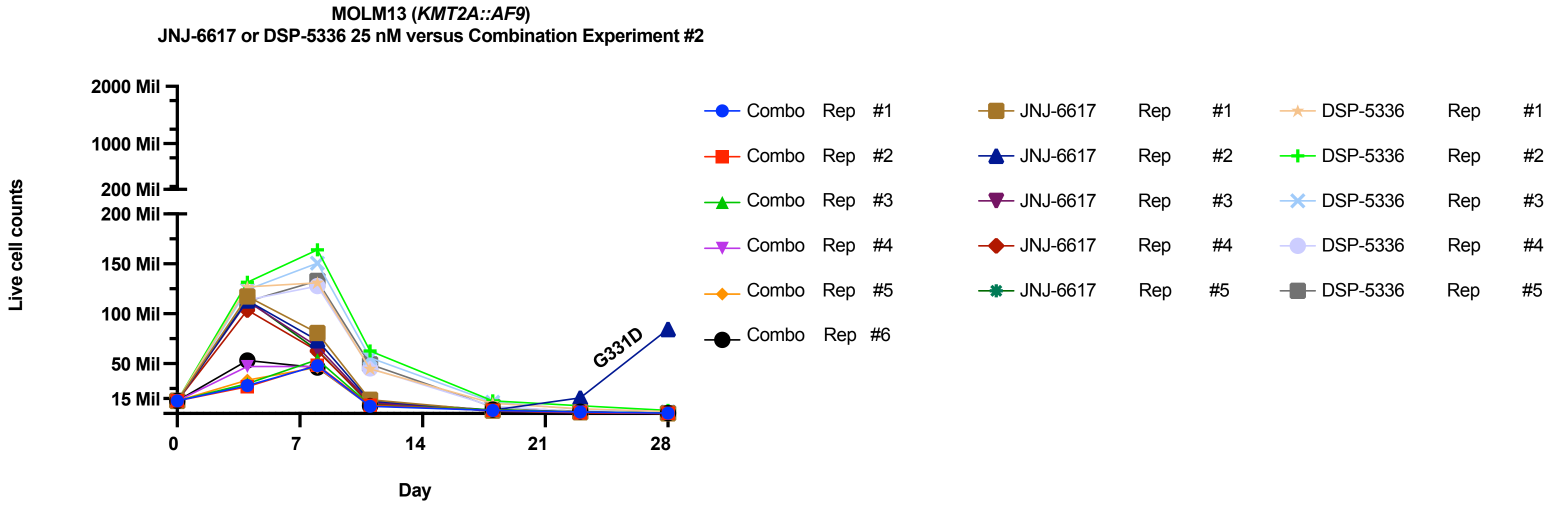

Supplementary Fig. 12: De novo *MEN1* mutation emergence *in vivo* corroborates screen

12A. Table describing the *KMT2A*-fusion in each PDX model as assessed by RNA-sequencing and the co-mutations present, as assessed by Brigham and Women's Hospital rapid heme panel.

12B. CBAM44728 PDX. Kaplan-Meier curve reflecting the time to leukemic progression (human CD45% > 30% in peripheral blood after initial remission) and/or humane endpoint being reached. p <0.0001 for vehicle or 0.033% chow vs 0.1% or 0.3% chow, p <0.0001 for 0.1% vs. 0.3% chow, p >0.05 for vehicle vs. 0.033% chow. Mice reflect two independent PDX experiments with the CBAM-44728 (*KMT2A::AF10*) model with the same passage of cells: n = 5 per group for the first experiment; n = 5 vehicle, n = 5 0.033% chow, n = 15 0.1% chow, and n = 8 0.3% chow for the second experiment.

A

PDX Model	Fusion	Mutations on rapid heme panel (***MEN1* not on panel**)
CBAM-44728	*KMT2A::AF10*	*ASXL1* G642fs* 50.2%, *SETD2* R393* 58.0%, *TP53* T125M 59.0%, *TP53* D281N 49.8%
CPCT-0024	*KMT2A::EPS15*	*ASXL1* P701Lfs*2 42.0%, *BRAF* D594G 40.1%, *NRAS* G12A 50.3%

B

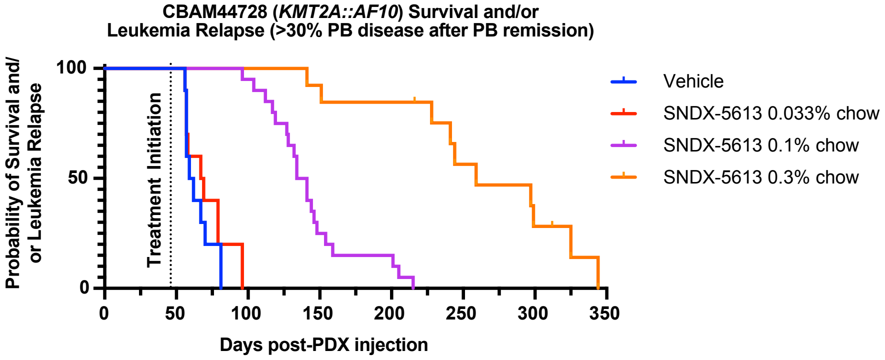

Supplementary Fig. 13: De novo *MEN1* mutation emergence *in vivo* corroborates screen

13A+B. Sanger sequencing chromatograms from bone marrow specimens at the time of death from Figure 6A (**13A**) and Figure 6B (**13B**). Detected *MEN1* mutations shown and annotated. Each specimen labeled with the cage number, mouse number, the date the PDX was transplanted, and the number of days the mouse was on SNDX-5613 chow at the time of death. All samples were from the CBAM-44728 PDX.

13C. CPCT-0024 PDX. Sanger sequencing corresponding to mice in Figure 6E was taken from bone marrow specimens at the time of death from one vehicle mouse, three mice treated with 0.033% chow, and one mouse treated with 0.1% chow. M327 and T349 codons highlighted.

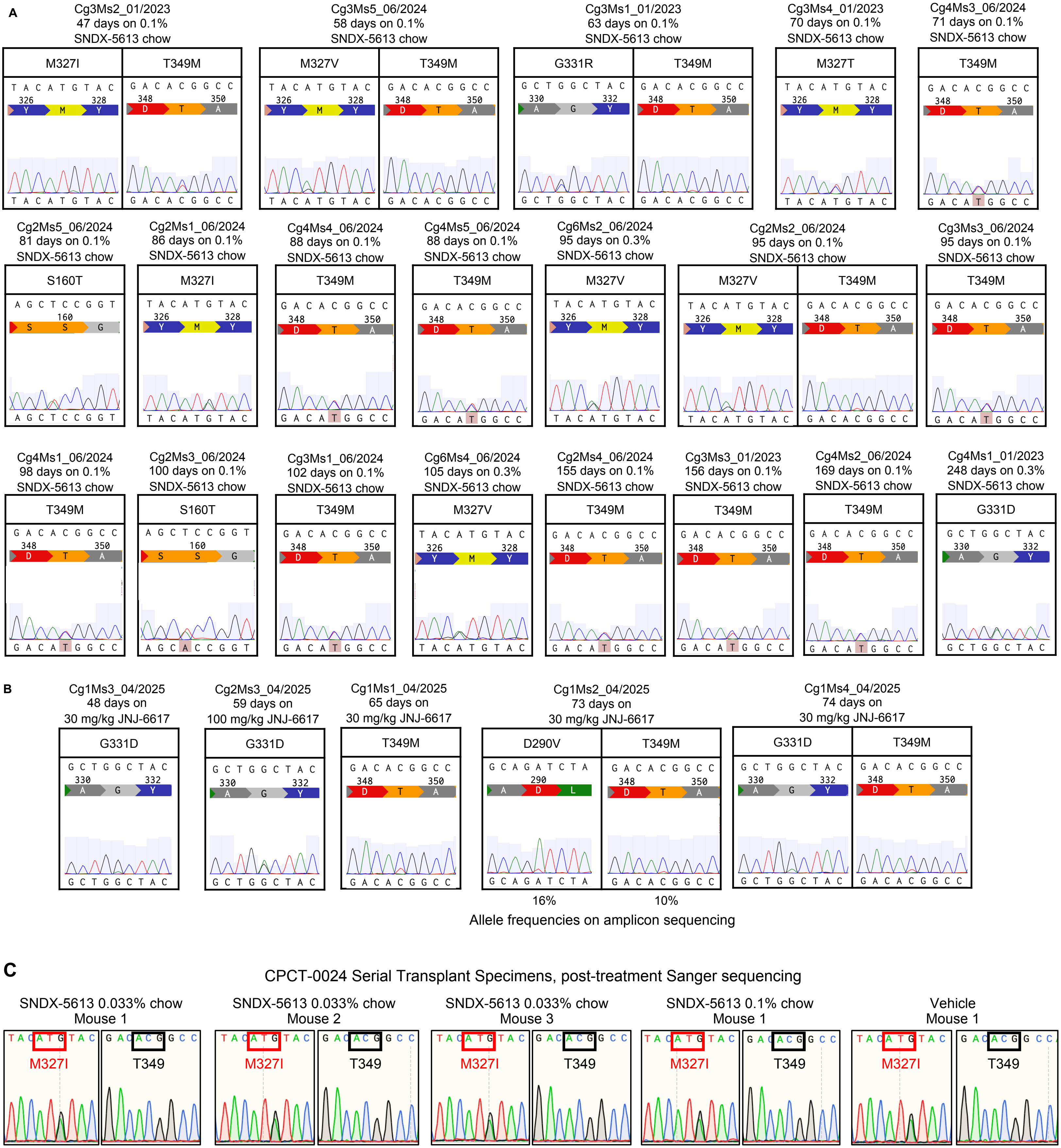

Supplementary Fig. 14: Gating strategies used in this manuscript

A. Example gating strategy used to quantify live cells, defined as DAPI (4′,6-diamidino-2-phenylindole)-negative, that is, BV421-negative cells, live RFP-positive cells, that is, PE-positive cells, and the percentage of live cells that were RFP-positive. Cells were first gated by SSC-A and FSC-A, then for BV421-negative cells to identify viable cells, and then for PE-positive cells to identify sgRNA-containing cells. This gating strategy was used for Main Figure 2B (live RFP-positive cells), Main Figure 5A and 5D (live cells), Supplementary Fig. 1B (live RFP-positive cells), Supplementary Fig. 4B and 4C (percentage of live cells that were RFP-positive), Supplementary Fig. 5B and 5E (live cells), and Supplementary Fig. 11A and 11B (live cells).

B. Example gating strategy used to quantify the percentage of human leukemia cells in a mixed population of human and mouse cells. Cells were first gated by SSC-A and FSC-A, then for singlets using FSC-H and FSC-A, then for BV421-negative cells to identify viable cells, and then for human CD45 PE-positive cells, representing patient-derived xenograft leukemia cells, or mouse CD45 APC-Cy7-positive cells, representing mouse cells. This gating strategy was used for Main Figures 6A, 6B, 6D, and 6E.

A

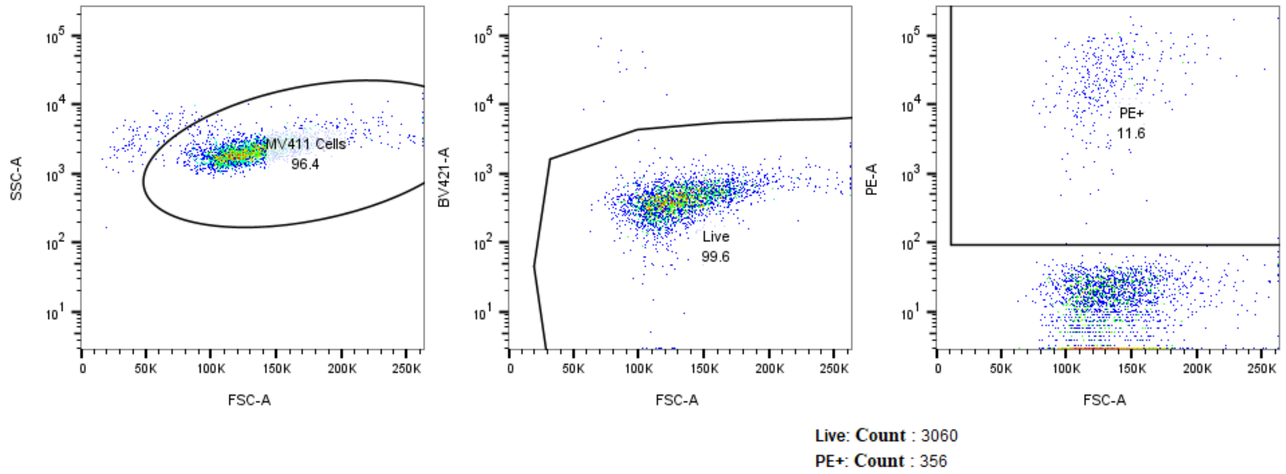

B

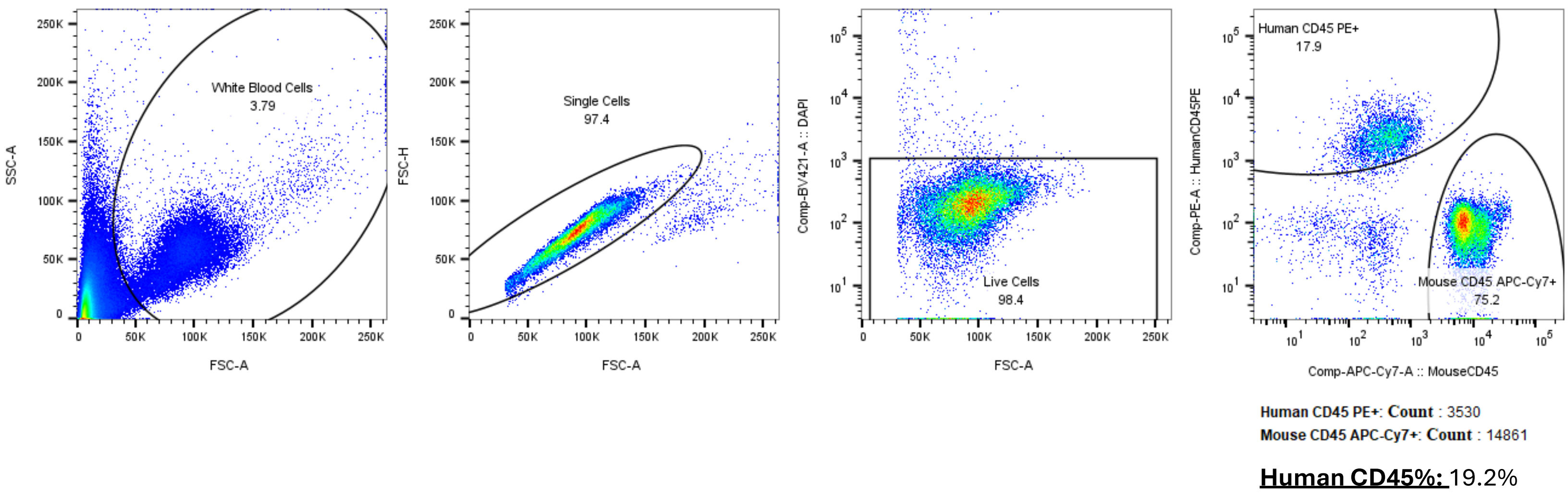

Supplementary Table 1: Data collection and refinement statistics for X-ray crystal structures reported in this study								
**Data Collection**	**Menin +JNJ-6617**	**[M322I]Menin +JNJ-6617**	**Menin +DSP-5336**	**[M322I]Menin +DSP-5336**	**Menin +KO-539**	**[M322I]Menin +KO-539**	**Menin +DS-1594**	**[M322I]Menin +DS-1594**
**PDB ID**	**9WKU**	**9WKV**	**9WN9**	**9WNA**	**9WNI**	**9WNJ**	**9WKW**	**9WKX**
Space Group	$P\ 1\ 2_1\ 1$	$P2_12_12_1$	$P\ 1\ 2_1\ 1$	$P2_12_12_1$	$P\ 1\ 2_1\ 1$	$P2_12_12_1$	$P\ 1\ 2_1\ 1$	$P2_12_12_1$
a (Angs)	70.16	58.87	70.43	58.70	70.09	58.67	69.79	57.63
b (Angs)	78.36	86.73	78.55	86.06	78.85	82.11	78.65	88.04
c (Angs)	92.43	202.74	93.01	201.89	93.34	203.75	92.61	196.66
α (degs)	90.00	90.00	90.00	90.00	90.00	90.00	90.00	90.00
β (degs)	101.62	90.00	101.64	90.00	101.54	90.00	101.51	90.00
γ (degs)	90.00	90.00	90.00	90.00	90.00	90.00	90.00	90.00
Wavelength (Angs)	0.96546	0.95373	0.95374	0.95373	0.95365	0.95374	0.95374	0.95374
Resolution	42.24 – 1.90	48.76 – 1.80	42.41 – 1.50	48.54 – 2.00	48.34 – 2.40	47.78 – 2.00	48.12 – 1.63	48.26 – 2.25
R_{merge}	0.093	0.151	0.091	0.150	0.166	0.100	0.077	0.295
$I/\sigma(I)$	8.0	13.8	11.5	14.4	5.8	16.1	16.0	8.7
Completeness (%)	99.6	99.6	100.0	99.7	99.0	100.0	97.9	99.9
Redundancy	3.4	13.5	6.9	13.6	3.5	13.5	7.0	13.4
**Refinement**								
Resolution	42.24 – 1.90	48.76 – 1.80	42.41 – 1.50	48.54 – 2.00	48.34 – 2.40	47.78 – 2.00	48.12 – 1.63	48.26 – 2.25
Number of used Reflections	73216	92238	150358	66389	37227	64083	114372	45923
R_{work} / R_{free}	0.1741 / 0.2042	0.1677 / 0.1983	0.1844 / 0.2138	0.1728 / 0.2124	0.2079 / 0.2747	0.1660 / 0.2069	0.1538 / 0.1763	0.1892 / 0.2387
Number of atoms (All)	7904	8088	8102	7845	7723	7830	8218	7703
Protein	7312	7399	7271	7383	7198	7350	7393	7382
Water	485	565	721	358	425	372	682	220
Ligands	107	124	110	104	100	108	143	101
Avg B-factors (A**2) (Overall)	20	25	17	30	35	29	15	34
**RMS deviations**								
Bond lengths (Angs)	0.0096	0.011	0.012	0.009	0.006	0.010	0.013	0.008
Bond angles (degs)	1.595	1.672	1.787	1.578	1.443	1.617	1.802	1.502
**Ramachandran Statistics**								
Residues in favoured regions (%)	98	98	98	98	96	98	98	96
Residues in allowed regions (%)	2	2	2	2	4	2	2	4
Residues in disallowed regions (%)	0	0	0	0	0	0	0	0

Supplementary Table 2: IC_{50} values and fold-change relative to wild-type menin for inhibitors tested against wild-type and mutant menin in TR-FRET assays

Raw IC_{50} values

	TR-FRET FITC-KMT2A-4-43 probe				TR-FRET FITC-KMT2B-15-48 probe			
Compound	Wild-type	M327I	T349M	G331R	Wild-type	C334R	E368K	V372A
DS-1594	1.7	74.0	120.6	40.2	8.5	>468.0	3.1	5.5
JNJ-6617	0.5	8.3	8.6	0.4	8.5	4.3	1.9	5.0
KO-539	1.6	292.1	6.9	1.2	40.9	29.4	7.6	>862.4
SNDX-5613	6.7	908.9	266.2	41.5	26.4	8.6	2.4	40.6
DSP-5336	7.7	202.6	81.5	4.2	33.4	12.7	>619.9	21.8

IC_{50} fold change: mutant/wild-type

	TR-FRET FITC-KMT2A-4-43 probe				TR-FRET FITC-KMT2B-15-48 probe			
Compound	Wild-type	M327I	T349M	G331R	Wild-type	C334R	E368K	V372A
DS-1594	1.0	42.7	69.6	23.2	1.0	>55.3	0.4	0.6
JNJ-6617	1.0	18.2	18.9	1.0	1.0	0.5	0.2	0.6
KO-539	1.0	183.2	4.3	0.7	1.0	0.7	0.2	>21.0
SNDX-5613	1.0	135.1	39.6	6.2	1.0	0.3	0.1	1.5
DSP-5336	1.0	26.3	10.6	0.5	1.0	0.4	>18.5	0.7

Number of experimental replicates, each with two technical replicates

	TR-FRET FITC-KMT2A-4-43 probe				TR-FRET FITC-KMT2B-15-48 probe			
Compound	Wild-type	M327I	T349M	G331R	Wild-type	C334R	E368K	V372A
DS-1594	8	6	6	6	3	3	3	3
JNJ-6617	10	6	6	6	3	3	3	3
KO-539	11	7	7	6	3	3	3	3
SNDX-5613	155	85	85	87	3	3	3	3
DSP-5336	9	7	7	6	3	3	3	3

IC_{50} values in nM

IC_{50} shift is mutant / wild-type

In KMT2B experiments, ">" means that at least one experimental replicate had an IC_{50} greater than the assay limit, so the experimental replicate was capped at the highest dose utilized (1000 nM)

Supplementary Table 3. Analysis of the binding kinetics/affinity of the FITC-KMT2A-4-43 peptide to human menin using the Cytiva T200 SPR system.[a]

PARAMETER	580 RU HIS-Menin	1250 RU HIS-Menin
k_{on}	2.8×10^{6} M^{-1} s^{-1}	3.9×10^{6} M^{-1} s^{-1}
k_{off}	4.9×10^{-4} s^{-1}	7.9×10^{-4} s^{-1}
K_D	0.17 nM	0.20 nM
R_{max}	37 RU	90 RU

[a] Data represent the mean of duplicate experiments. The k_{on}, k_{off} and K_d values were generated by the Cytiva machine software.

Supplementary Table 4. Off-rate kinetics of FITC–KMT2A(4–43) dissociation from WT and mutant menin measured by HTRF and parameters used for IC50-to-Ki conversion								
	WT		M327I		G331R		T349M	
	koff, min-1	t1/2, min	koff, min-1	t1/2, min	koff, min-1	t1/2, min	koff, min-1	t1/2, min
Replicate 1	0.092	7.5	0.054	12.8	0.169	4.1	0.061	11.4
Replicate 2	0.091	7.6	0.059	11.8	0.148	4.7	0.045	15.3
Replicate 3	0.085	8.2	0.063	10.9	0.171	4.1	0.042	16.4
Replicate 4	0.095	7.3	0.055	12.6	0.199	3.5	0.056	12.4
Mean	0.091	7.663	0.058	12.041	0.172	4.079	0.051	13.871
SD	0.004	0.360	0.004	0.851	0.021	0.490	0.009	2.358
Relative Kd (koff ratio)		1.00		0.64		1.90		0.56

Basis for adjusting IC50 to Ki conversion				
	Relative Kd (koff ratio)	Kd (SPR, average)	Kd adjustment based on the off-rate	Kd
WT	1	185 pM	1x	185 pM
M327I	0.64		0.64x	117 pM
G331R	1.9		1.90x	352 pM
T349M	0.56		0.56x	102 pM

Ki = IC50 / (1 + [FITC-KMT2A(4-43)] / KD)
