# Supplementary material for: CRISPR base editor screening identifies spectrum of MEN1 mutations impacting menin inhibitors in clinical trials
Source: Nat Commun. 2026 May 9;17:6265. doi: 10.1038/s41467-026-72685-1 (PMC13377036; doi:10.1038/s41467-026-72685-1)
Supplement: Supplementary file 2 — Descriptions of Additional Supplementary Files [file 41467_2026_72685_MOESM2_ESM.pdf]

Description of Additional Supplementary Files

Supplementary Data 1: Menin Inhibitor Analytic Data
Zip files containing menin inhibitor analytic data. This contains summaries of the results of the NMR data resolving the purity/identity of the compounds that we used in our study.

Supplementary Data 2: Base Editor Screen Annotation and Results
Excel file with annotation of CRISPR base editor screen with Log_2FCs for each drug condition relative to DMSO control, presented as the average of three technical replicates, individual replicate performance, and MAGeCK_MLE annotation and analysis.

Supplementary Data 3: Mapping of Base Editor Screen Hits onto Protein Structures
Zip file with mapping and visualization of base-editing readouts (or "hits") onto protein structures via the Genomics 2 Proteins portal.

Supplementary Data 4: Analysis of the binding kinetics/affinity of the FITC-KMT2A-4-43 peptide to human menin using the Cytiva T200 Surface Plasmon Resonance (SPR) system.
This data underlies Supplementary Table 3.

Supplementary Data 5: Off-rate kinetics of the FITC-KMT2A-4-43 dissociation from WT and mutant menin by HTFR assays and parameters used for IC50-to-Ki conversion.
This data underlies Supplementary Table 4.

Supplementary Data 6: Electron Density Map
Image of a portion of the electron density map (including contour level) for each crystal structure.

Supplementary Data 7: Omit Map
Zip file with omit maps (including contour level) for all ligand-bound crystal structures.
