# Supplementary material for: CRISPR base editor screening identifies spectrum of MEN1 mutations impacting menin inhibitors in clinical trials
Source: Nat Commun. 2026 May 9;17:6265. doi: 10.1038/s41467-026-72685-1 (PMC13377036; doi:10.1038/s41467-026-72685-1)
Supplement: Supplementary file 3 — Supplementary Data 1 [file 41467_2026_72685_MOESM3_ESM.zip › SNDX-0060981 (DS-1594).pdf]

CONFIDENTIAL

FS_SYE2102122_28_D424132

BRUKER

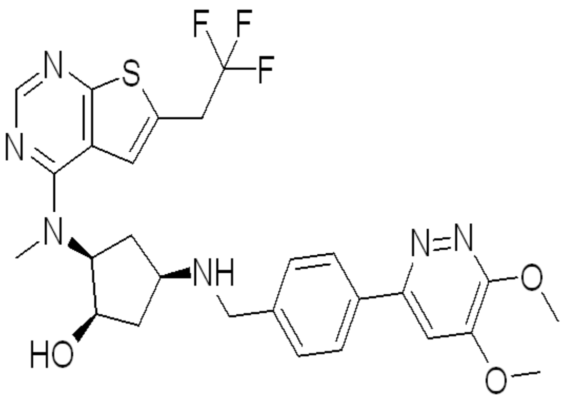

Chemical Formula: $C_{27}H_{29}F_3N_6O_3S$

Molecular Weight: 574.62

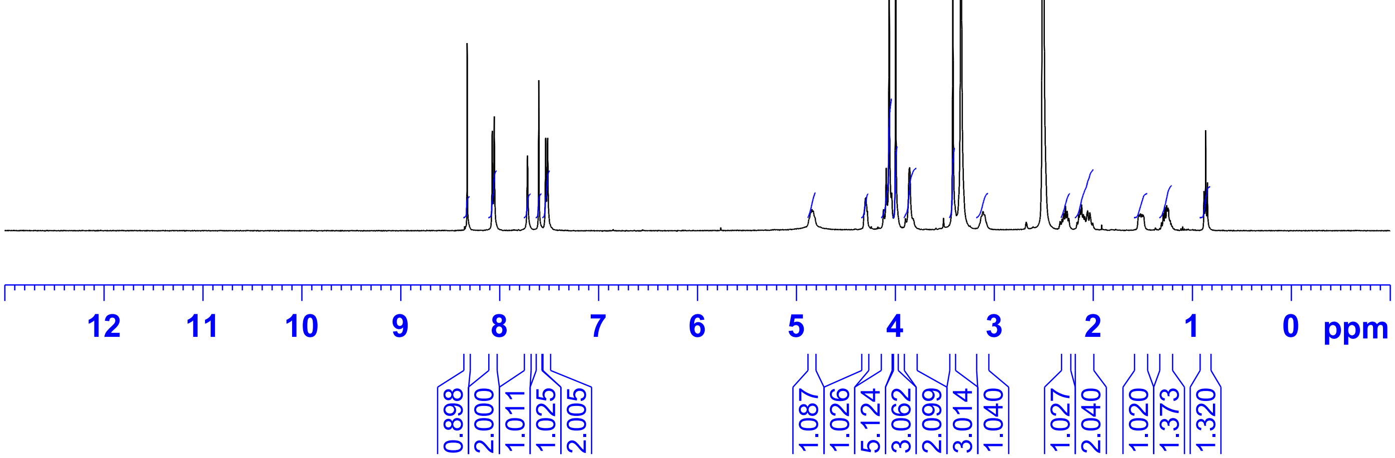

Current Data Parameters

NAME	FS_SYE2102122_28_D424132
EXPNO	1
PROCNO	1

F2 - Acquisition Parameters

Date_	20220117	
Time	16.26	h
INSTRUM	Avance	
PROBHD	Z104450_0002 (	
PULPROG	zg30	
TD	32768	
SOLVENT	DMSO	
NS	16	
DS	2	
SWH	8196.722	Hz
FIDRES	0.500288	Hz
AQ	1.9988480	sec
RG	101	
DW	61.000	usec
DE	12.74	usec
TE	300.3	K
D1	2.00000000	sec
TD0	1	
SFO1	400.3724723	MHz
NUC1	1H	
P0	4.90	usec
P1	14.70	usec
PLW1	13.30000019	W

F2 - Processing parameters

SI	65536	
SF	400.3700000	MHz
WDW	EM	
SSB	0	
LB	0.30	Hz
GB	0	
PC	1.00	

SYNGENE INTERNATIONAL LTD.
SC/AD/01-005

CONFIDENTIAL

FS_SYE2102122_28_D424132

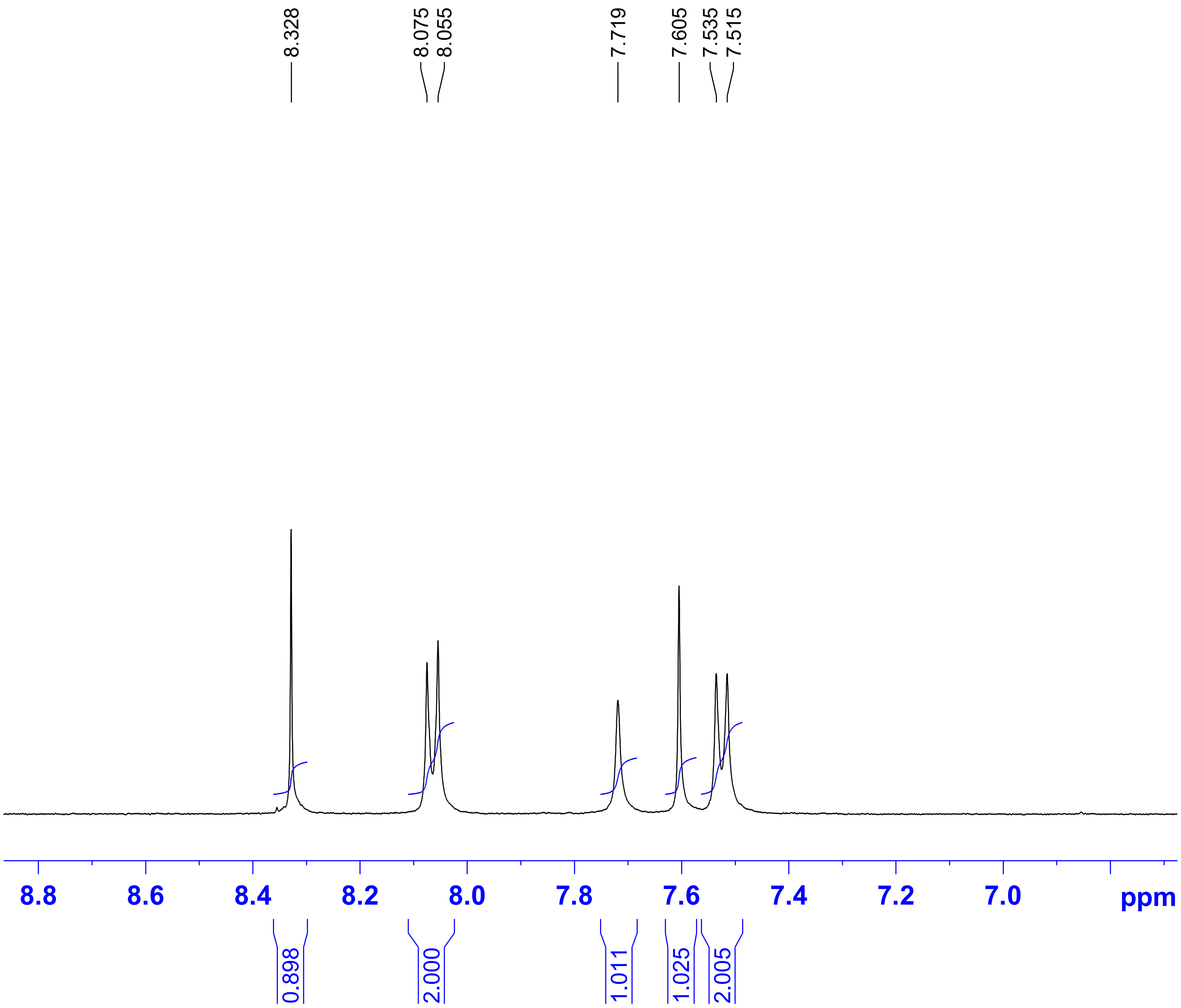


```
Current Data Parameters
NAME        FS_SYE2102122_28_D424132
EXPNO                      1
PROCNO                     1

F2 - Acquisition Parameters
Date_               20220117
Time                   16.26 h
INSTRUM               Avance
PROBHD    Z104450_0002 (
PULPROG                 zg30
TD                     32768
SOLVENT                 DMSO
NS                        16
DS                         2
SWH                 8196.722 Hz
FIDRES              0.500288 Hz
AQ                 1.9988480 sec
RG                       101
DW                    61.000 usec
DE                     12.74 usec
TE                     300.3 K
D1                2.00000000 sec
TD0                        1
SFO1             400.3724723 MHz
NUC1                      1H
P0                      4.90 usec
P1                     14.70 usec
PLW1             13.30000019 W

F2 - Processing parameters
SI                     65536
SF               400.3700000 MHz
WDW                       EM
SSB       0
LB                      0.30 Hz
GB        0
PC                      1.00
```

SYNGENE INTERNATIONAL LTD.
SC/AD/01-005

CONFIDENTIAL

FS_SYE2102122_28_D424132

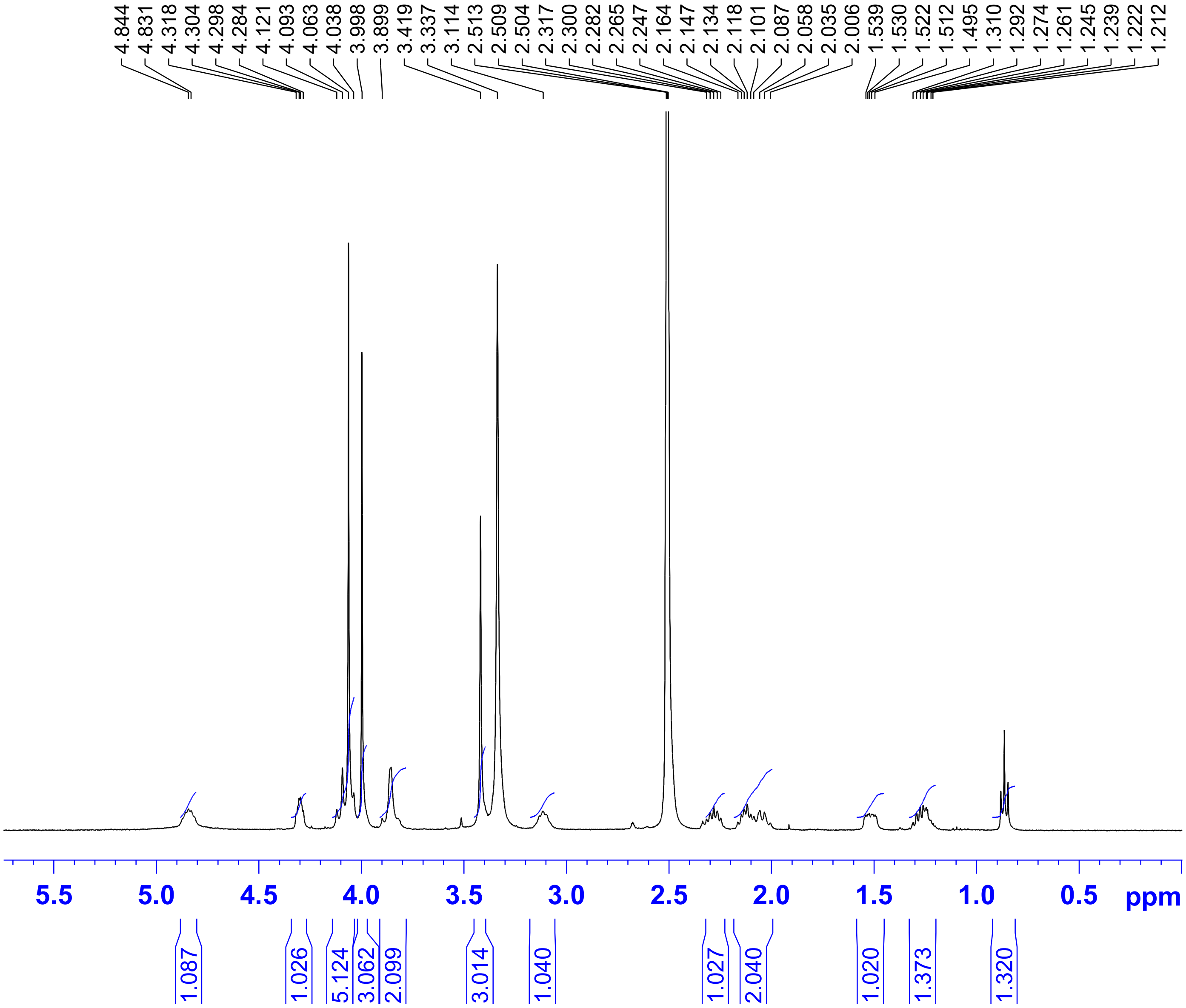

Current Data Parameters
NAME FS_SYE2102122_28_D424132
EXPNO 1
PROCNO 1

F2 - Acquisition Parameters
Date_ 20220117
Time 16.26 h
INSTRUM Avance
PROBHD Z104450_0002 (
PULPROG zg30
TD 32768
SOLVENT DMSO
NS 16
DS 2
SWH 8196.722 Hz
FIDRES 0.500288 Hz
AQ 1.9988480 sec
RG 101
DW 61.000 usec
DE 12.74 usec
TE 300.3 K
D1 2.00000000 sec
TD0 1
SFO1 400.3724723 MHz
NUC1 1H
P0 4.90 usec
P1 14.70 usec
PLW1 13.30000019 W

F2 - Processing parameters
SI 65536
SF 400.3700000 MHz
WDW EM
SSB 0
LB 0.30 Hz
GB 0
PC 1.00

SYNGENE INTERNATIONAL LTD.
SC/AD/01-005

CONFIDENTIAL

FS_SYE2102122_28_D424132-D2O

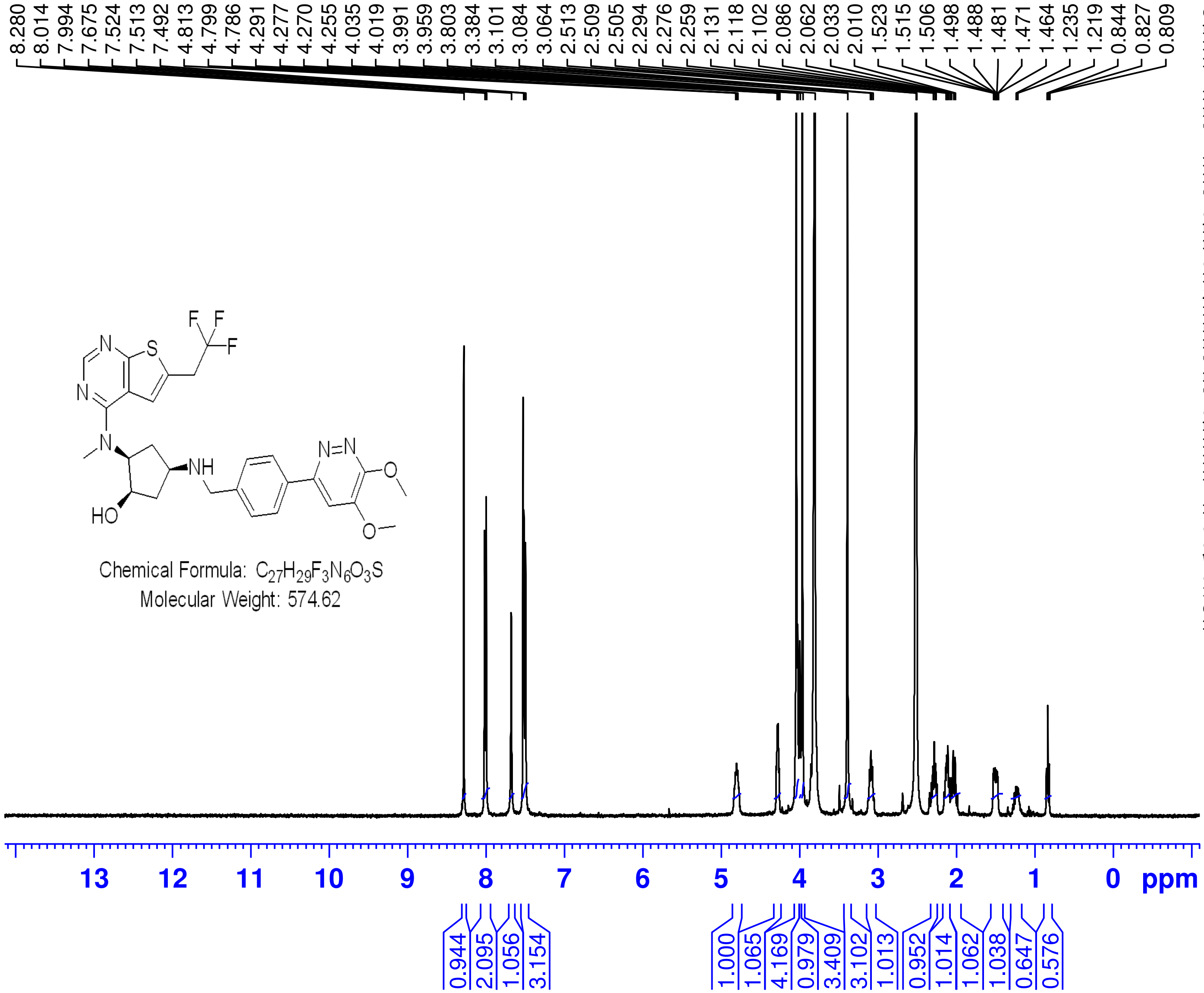

Chemical Formula: $C_{27}H_{29}F_3N_6O_3S$

Molecular Weight: 574.62

Current Data Parameters

NAME	FS_SYE2102122_28_D424132-D2O
EXPNO	1
PROCNO	1

F2 - Acquisition Parameters

Date_	20220117	
Time	20.00	h
INSTRUM	Avance	
PROBHD	Z104450_0002 (	
PULPROG	zg30	
TD	32768	
SOLVENT	DMSO	
NS	16	
DS	2	
SWH	8196.722	Hz
FIDRES	0.500288	Hz
AQ	1.9988480	sec
RG	101	
DW	61.000	usec
DE	12.74	usec
TE	300.3	K
D1	2.00000000	sec
TD0	1	
SFO1	400.3724723	MHz
NUC1	1H	
P0	4.90	usec
P1	14.70	usec
PLW1	13.30000019	W

F2 - Processing parameters

SI	65536	
SF	400.3700000	MHz
WDW	EM	
SSB	0	
LB	0.30	Hz
GB	0	
PC	1.00	

SYNGENE INTERNATIONAL LTD.
SC/AD/01-005

==

Data file :C:\CHEM32\1\DATA\Y2022\JAN\17012022 2022-01-17 14-57-14\D424132.D
Acq Method :C:\CHEM32\1\METHODS\XB_0595TF.M
Injection Date :17-Jan-2022 Vial No. :P2-C-09
Injection Time :15:01:29 Injection vol :2.000
Sample Name :SYE2102122-28

==

Method info : Column:X-Bridge C8 (50 x 4.6) mm 3.5ìm
Mobile Phase 'A':0.1% TFA in Water
Mobile Phase 'B': ACN
Flow : 2.0ml\min

Time(min)	%B
0.0	05
8.0	100
8.1	100
8.5	05
10.0	05

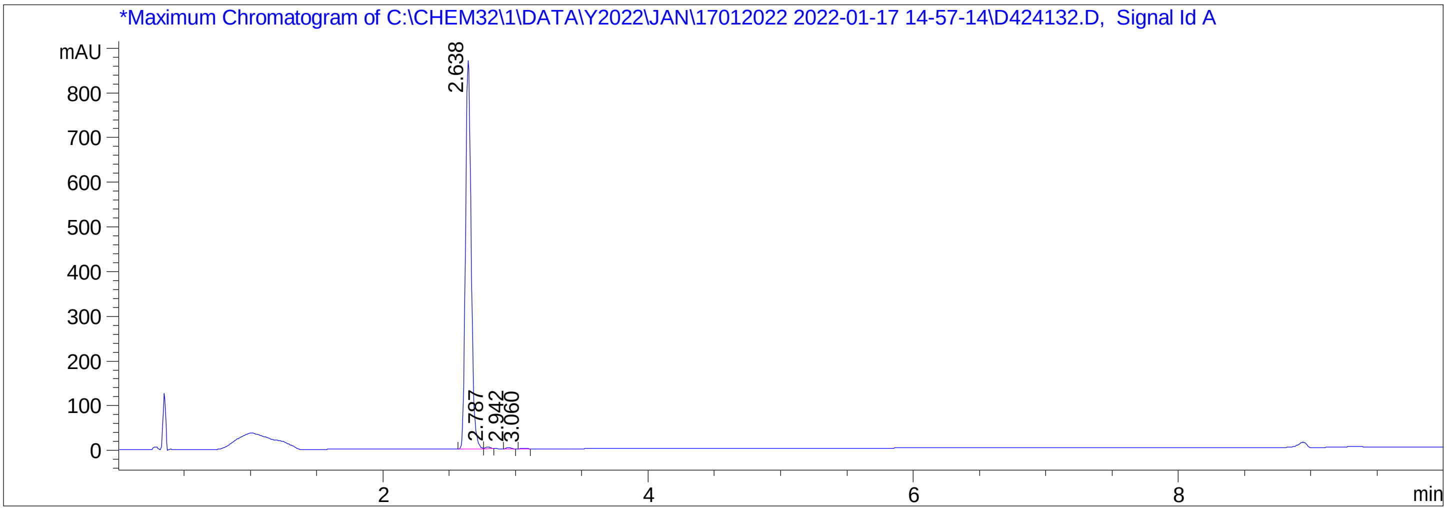

Peak	RT min	Height	Area	Area %
1	2.638	876.258	2602.085	99.246
2	2.787	3.333	8.421	0.321
3	2.942	2.897	7.593	0.290
4	3.060	1.339	3.751	0.143

==

End of report

Analysed By : Checked By :

Data file : D:\DATA\JAN2022\19012022 2\D424132B.D
Acq Method : D:\DATA\JAN2022\19012022 2\XB_595TFA_6MIN.M
Injection Date : 19 -Jan-2022 Vial No. : D1F-C9
Injection Time : 10:36:22 Injection vol :1.0 µL
Sample Name : SYE2102122-28

Method info : Column :XBridge C8 (50x4.6mm) 3.5 µm
Mobile phase :A :0.1% TFA in H2O
Mobile phase :B: 100% ACN
Flow Rate :1.5ml/min

Time (min)	%B
0.0	5
2.5	95
4.0	95
4.5	5
5.5	5

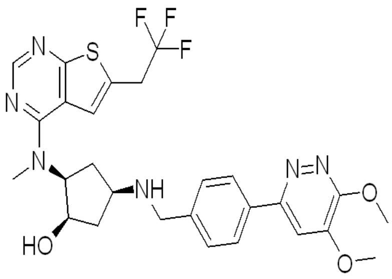

Chemical Formula: $C_{27}H_{29}F_3N_6O_3S$
Molecular Weight: 574.62

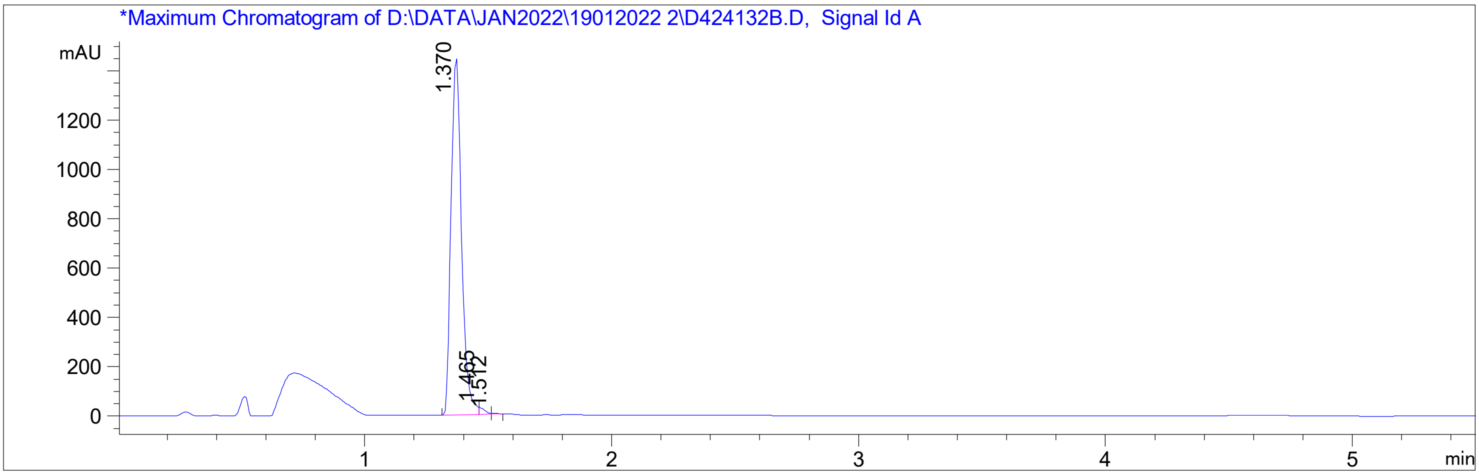

Peak No	RT min	Area	Area %
1	1.370	4291.022	99.072
2	1.465	35.721	0.825
3	1.512	4.466	0.103

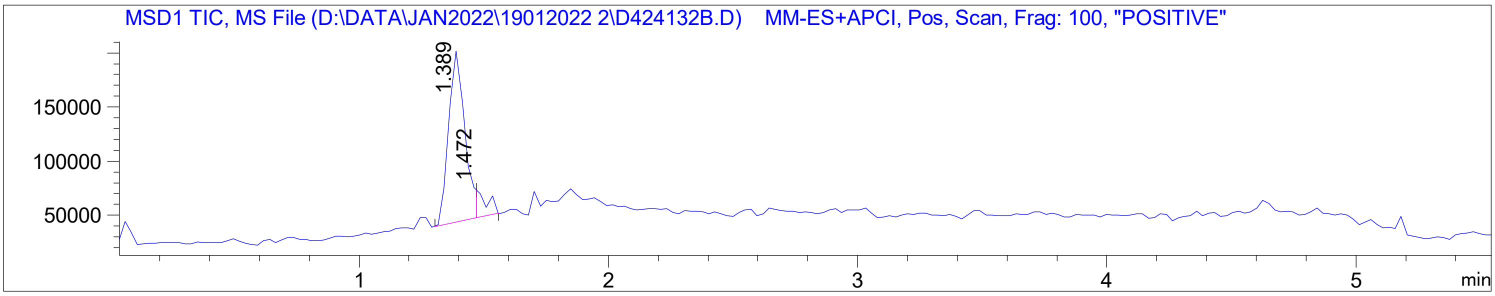

Analysed by : Checked by:

Instrument Code : S_DC_ARD_17-041

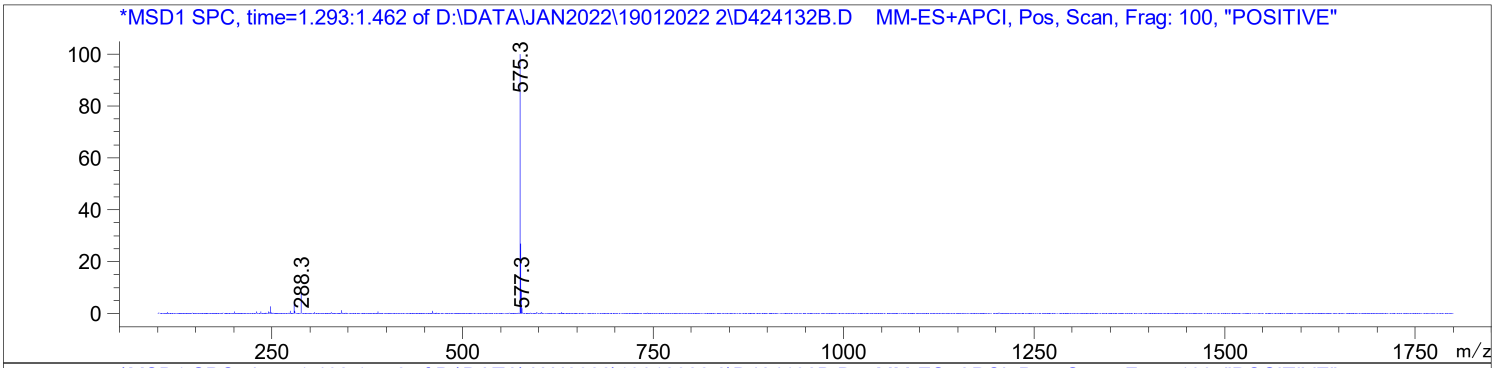

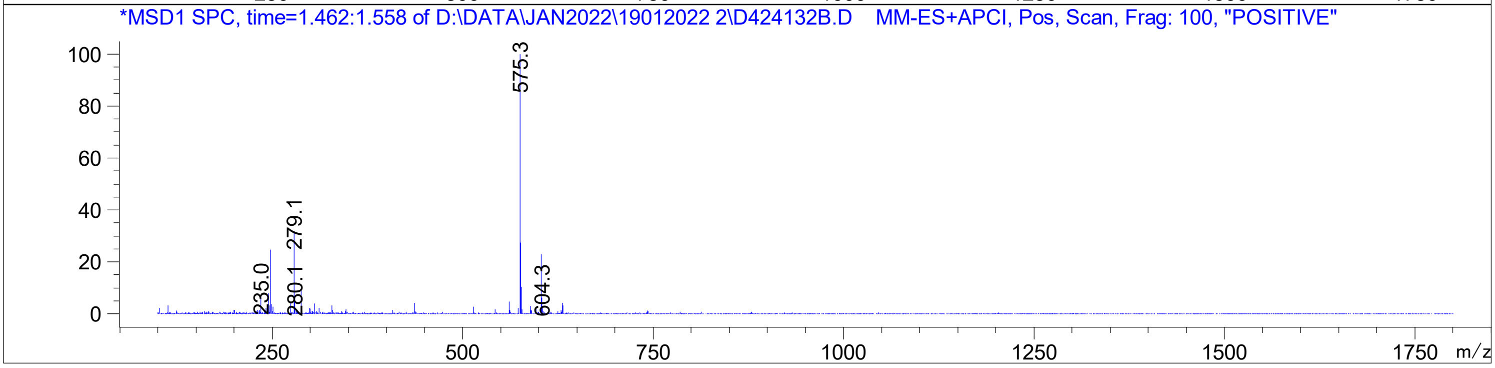

Analysed by :

Checked by:

Instrument Code : S_DC_ARD_17-041

Confidential
SFC Analytical Chromatogram

Syngene

Date : 17 Jan 2022 - 17:21	Username :
Method : C:\Pic Solution\Méthode\Analytical\Analytical\SFC-5-50.met	
Data : C:\Pic Solution\Data\2022\JAN2022\17JAN22\17JAN22A\ D424132_024.dat	
Comments :	
**Elution**	**Injection**
FlowRate : 5 ml/min	Column Name : YMC Cellulose-SC
Co-Solvent : 50%	Sample Name : RB3 : D424132
Co-Solvent Name : 0.5% Isopropylamine in Methanol	Injected Volume : 15 µl
Outlet Pressure: 100 bar	Temperature : 35 °C

Note: This compound was analysed using above method and found as single peak. It does not confirm that the compound has only single isomer. A racemic mixture or reference method need to be provided to confirm the presence of single isomer.

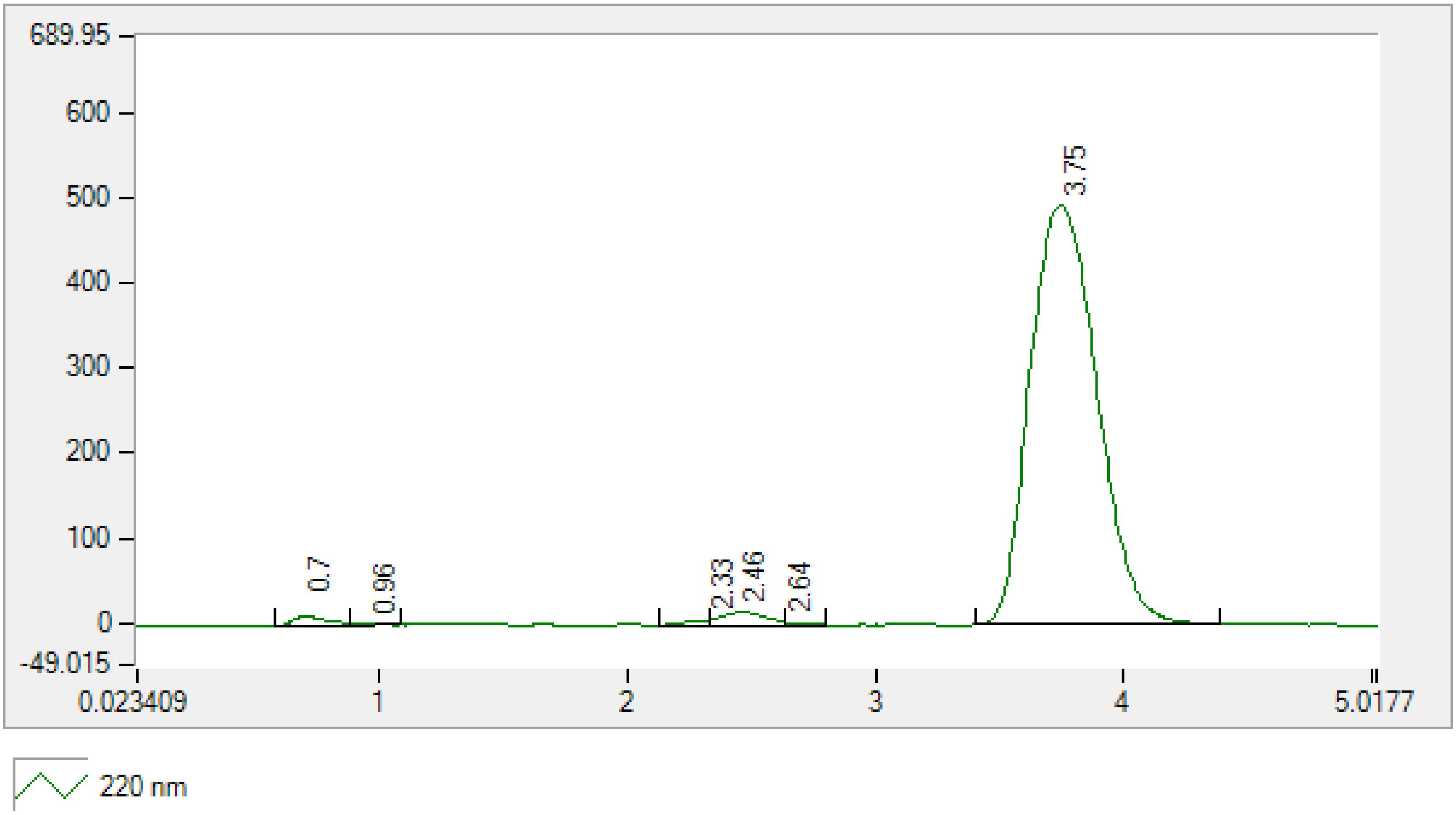

Results UV2			
Sr.No.	RT	Area	Area %
1	0.70	89.246	0.931
2	0.96	17.651	0.184
3	2.33	31.247	0.326
4	2.46	187.081	1.951
5	2.64	10.369	0.108
6	3.75	9252.190	96.500

Analysed By:	Instrument Code: S/DC/ARD/22-014	Checked By:
