# Supplementary material for: CRISPR base editor screening identifies spectrum of MEN1 mutations impacting menin inhibitors in clinical trials
Source: Nat Commun. 2026 May 9;17:6265. doi: 10.1038/s41467-026-72685-1 (PMC13377036; doi:10.1038/s41467-026-72685-1)

Confidential

FS_SYN2401479_78_G390281

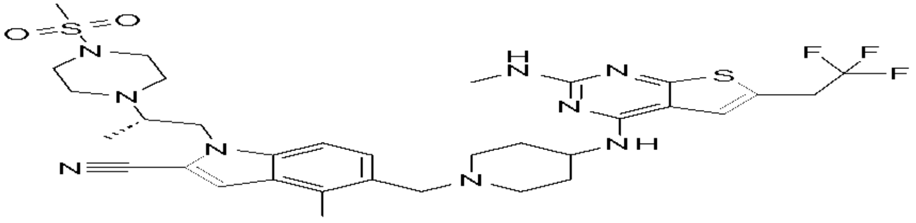

Chemical Formula: $C_{33}H_{42}F_3N_9O_2S_2$
Molecular Weight: 717.88

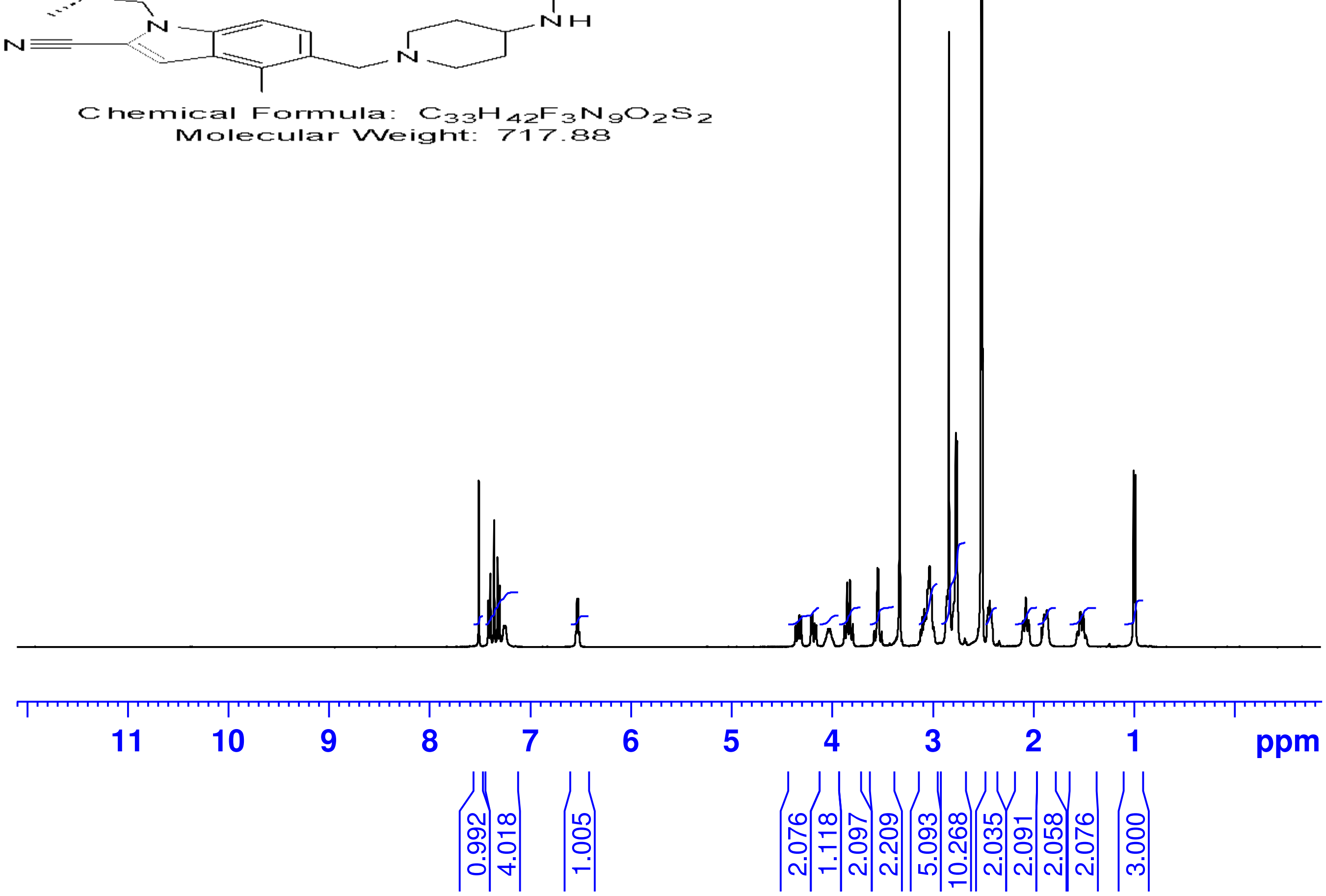

Current Data Parameters

NAME	FS_SYN2401479_78_G390281
EXPNO	1
PROCNO	1

F2 - Acquisition Parameters

Date_	20250606
Time	11.42 h
INSTRUM	Avance Neo Nanobay
PROBHD	Z163739_0807
PULPROG	zg30
TD	32768
SOLVENT	DMSO
NS	64
DS	0
SWH	8196.721 Hz
FIDRES	0.500288 Hz
AQ	1.9988480 sec
RG	101
DW	61.000 usec
DE	13.89 usec
TE	298.5 K
D1	2.00000000 sec
TD0	1
SFO1	400.4024725 MHz
NUC1	1H
P0	2.67 usec
P1	8.00 usec
PLW1	22.37000000 W

F2 - Processing parameters

SI	65536
SF	400.4000000 MHz
WDW	EM
SSB	0
LB	0.30 Hz
GB	0
PC	1.00

SYNGENE SCIENTIFIC SOLUTIONS LTD.
S/DC/ARD/01-010

Confidential

FS_SYN2401479_78_G390281

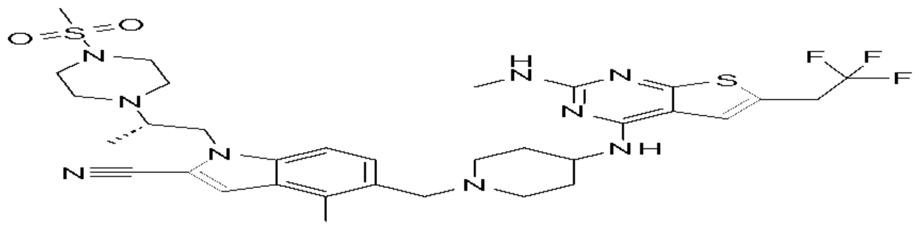

Chemical Formula: $C_{33}H_{42}F_3N_9O_2S_2$
Molecular Weight: 717.88

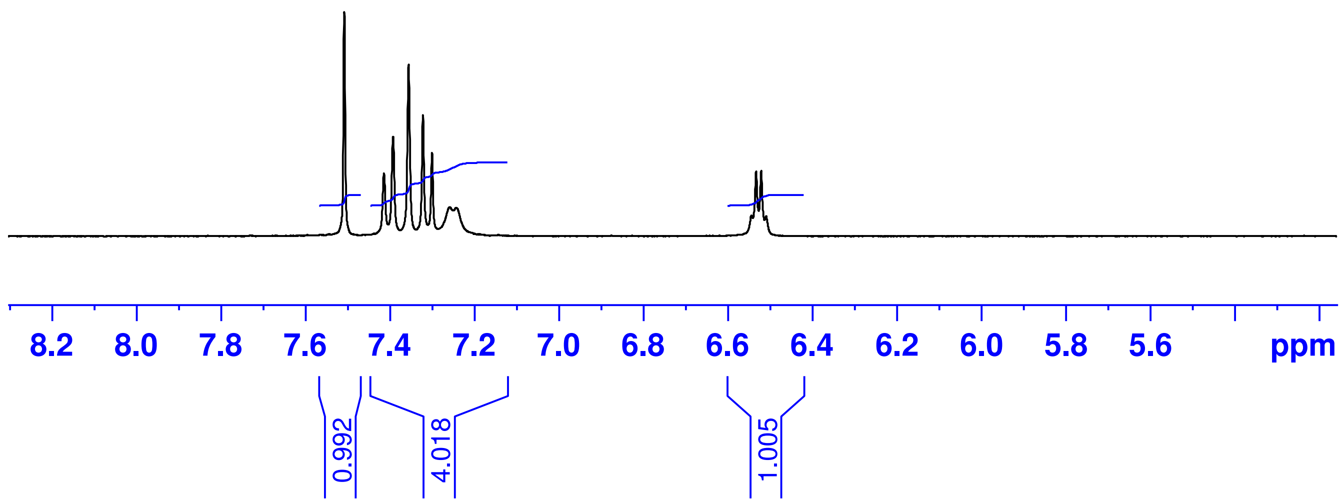


```
Current Data Parameters
NAME          FS_SYN2401479_78_G390281
EXPNO                        1
PROCNO                       1

F2 - Acquisition Parameters
Date_                 20250606
Time                     11.42 h
INSTRUM       Avance Neo Nanobay
PROBHD             Z163739_0807
PULPROG                   zg30
TD                       32768
SOLVENT                   DMSO
NS                          64
DS                           0
SWH                   8196.721 Hz
FIDRES                0.500288 Hz
AQ                   1.9988480 sec
RG                         101
DW                      61.000 usec
DE                       13.89 usec
TE                       298.5 K
D1                  2.00000000 sec
TD0                          1
SFO1               400.4024725 MHz
NUC1                        1H
P0                        2.67 usec
P1                        8.00 usec
PLW1               22.37000000 W

F2 - Processing parameters
SI                       65536
SF                 400.4000000 MHz
WDW                         EM
SSB                          0
LB                        0.30 Hz
GB                           0
PC                        1.00
```

SYNGENE SCIENTIFIC SOLUTIONS LTD.
S/DC/ARD/01-010

Confidential

FS_SYN2401479_78_G390281

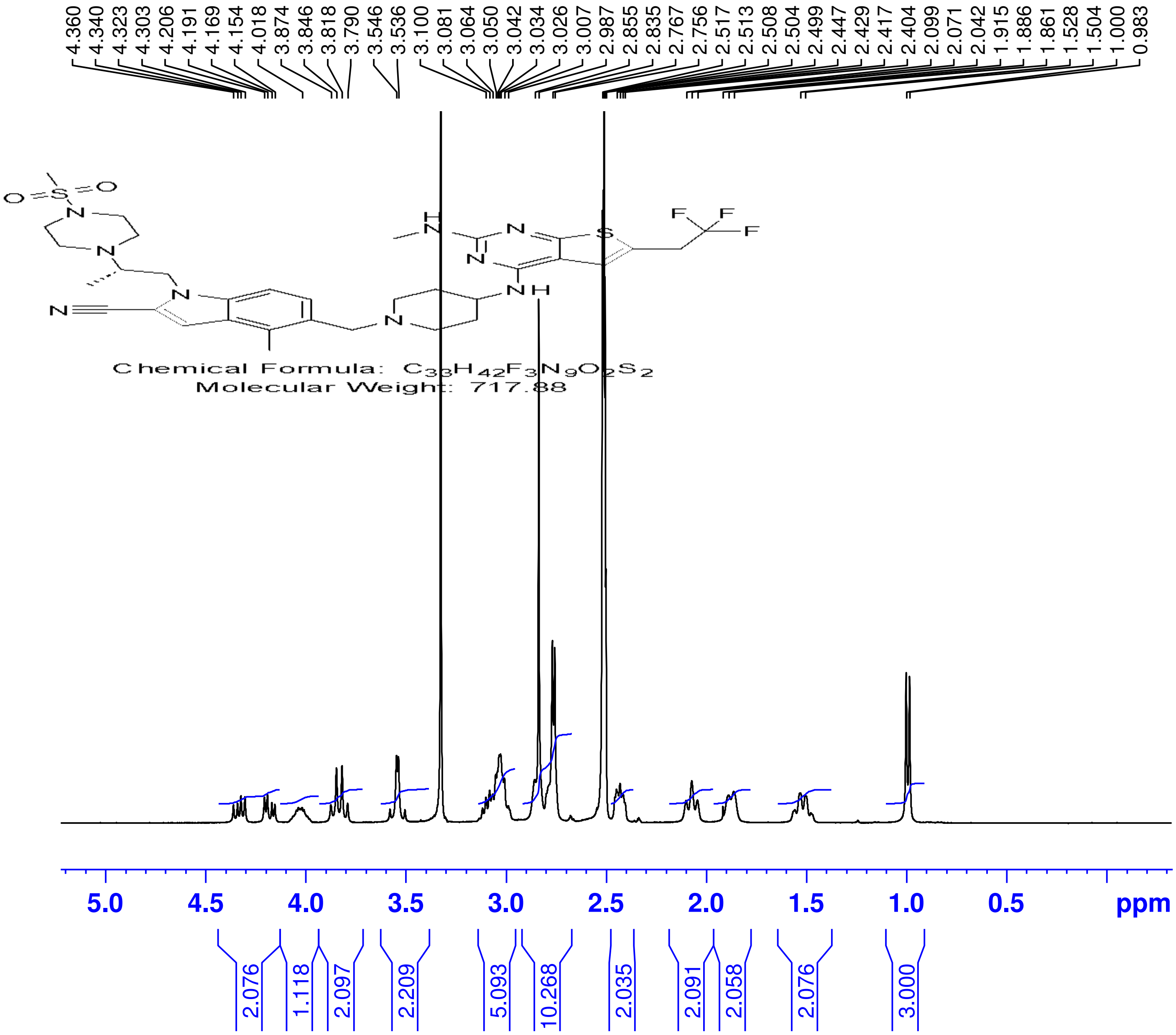

Current Data Parameters

NAME	FS_SYN2401479_78_G390281
EXPNO	1
PROCNO	1

F2 - Acquisition Parameters

Date_	20250606
Time	11.42 h
INSTRUM	Avance Neo Nanobay
PROBHD	Z163739_0807
PULPROG	zg30
TD	32768
SOLVENT	DMSO
NS	64
DS	0
SWH	8196.721 Hz
FIDRES	0.500288 Hz
AQ	1.9988480 sec
RG	101
DW	61.000 usec
DE	13.89 usec
TE	298.5 K
D1	2.00000000 sec
TD0	1
SFO1	400.4024725 MHz
NUC1	1H
P0	2.67 usec
P1	8.00 usec
PLW1	22.37000000 W

F2 - Processing parameters

SI	65536
SF	400.4000000 MHz
WDW	EM
SSB	0
LB	0.30 Hz
GB	0
PC	1.00

Confidential

FS_SYN2401479_78_G390281-VT
AT 80°C

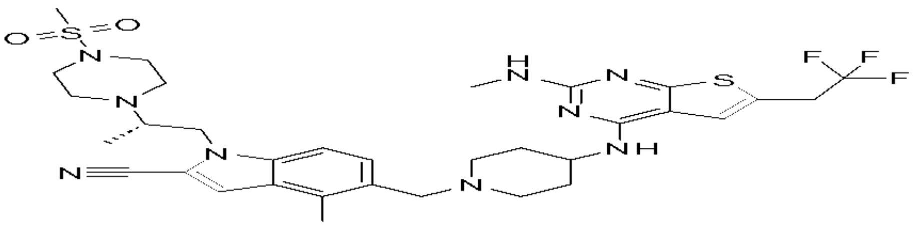

Chemical Formula: $C_{33}H_{42}F_3N_9O_2S_2$
Molecular Weight: 717.88

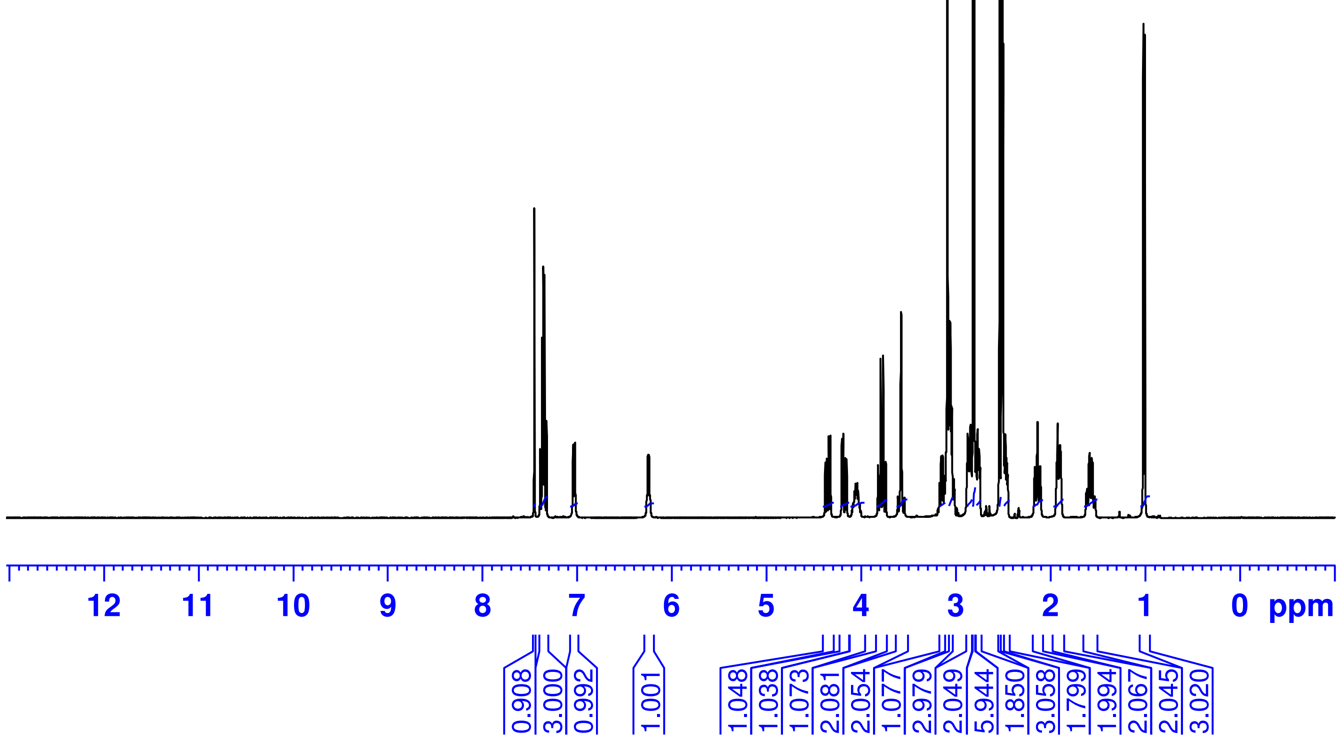


```
Current Data Parameters
NAME      FS_SYN2401479_78_G390281-VT
EXPNO                    1
PROCNO                   1

F2 - Acquisition Parameters
Date_             20250606
Time                 18.41 h
INSTRUM      Avance Neo 400
PROBHD       Z167430_0043 (
PULPROG               zg30
TD                   32768
SOLVENT               DMSO
NS                      64
DS                       0
SWH               8620.689 Hz
FIDRES            0.526165 Hz
AQ               1.9005440 sec
RG                     101
DW                  58.000 usec
DE                   12.45 usec
TE                   353.1 K
D1              1.00000000 sec
TD0                      1
SFO1           400.2324714 MHz
NUC1                    1H
P0                    4.00 usec
P1                   12.00 usec
PLW1            9.80620003 W

F2 - Processing parameters
SI                   65536
SF             400.2300000 MHz
WDW                     EM
SSB                      0
LB                    0.30 Hz
GB                       0
PC                    1.00
```

SYNGENE SCIENTIFIC SOLUTIONS LT
S/DC/ARD/01-014

Confidential

FS_SYN2401479_78_G390281-VT
AT 80°C

Chemical Formula: $C_{33}H_{42}F_3N_9O_2S_2$
Molecular Weight: 717.88

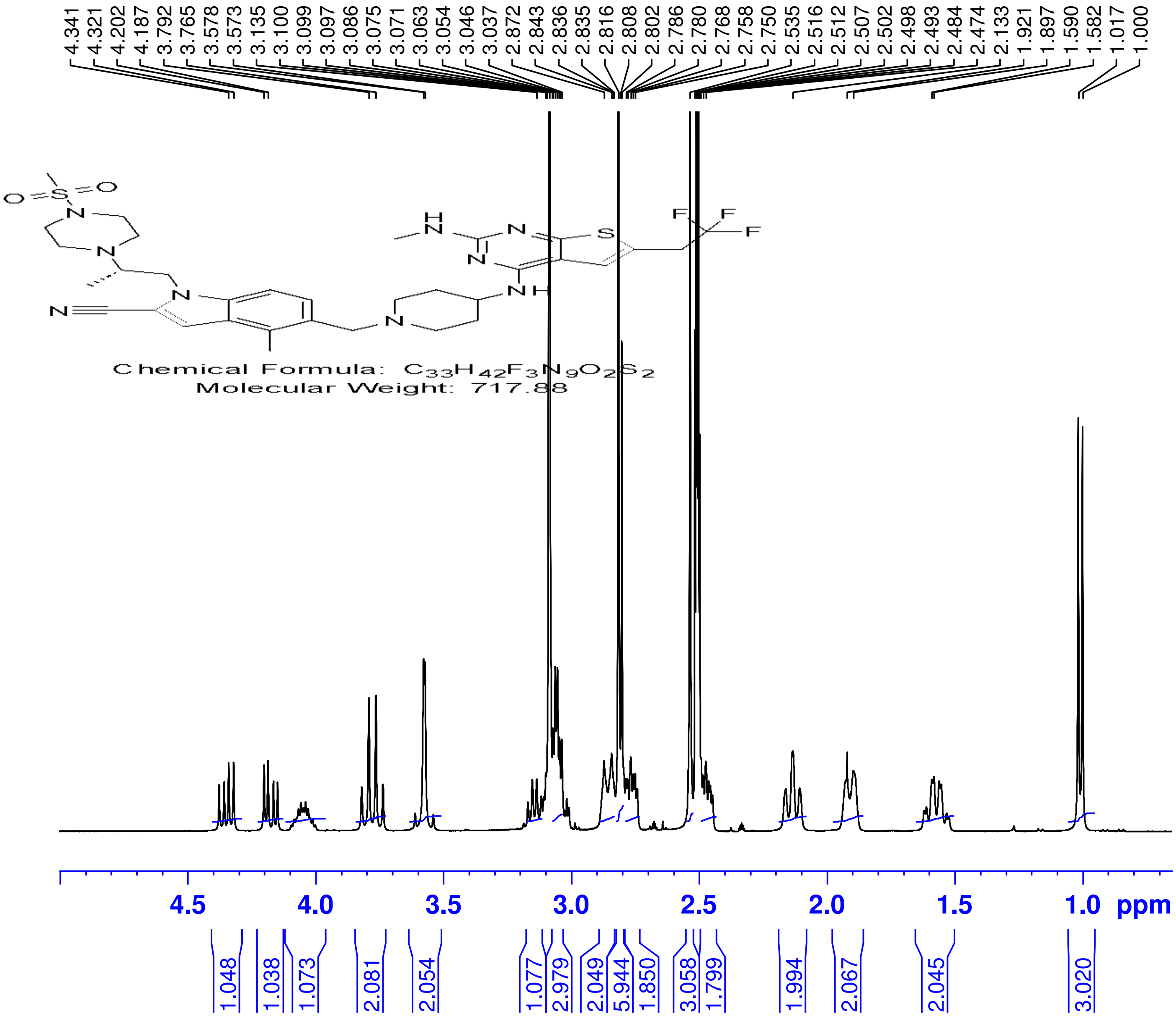

Current Data Parameters
NAME FS_SYN2401479_78_G390281-VT
EXPNO 1
PROCNO 1

F2 - Acquisition Parameters
Date_ 20250606
Time 18.41 h
INSTRUM Avance Neo 400
PROBHD Z167430_0043 (
PULPROG zg30
TD 32768
SOLVENT DMSO
NS 64
DS 0
SWH 8620.689 Hz
FIDRES 0.526165 Hz
AQ 1.9005440 sec
RG 101
DW 58.000 usec
DE 12.45 usec
TE 353.1 K
D1 1.00000000 sec
TD0 1
SFO1 400.2324714 MHz
NUC1 1H
P0 4.00 usec
P1 12.00 usec
PLW1 9.80620003 W

F2 - Processing parameters
SI 65536
SF 400.2300000 MHz
WDW EM
SSB 0
LB 0.30 Hz
GB 0
PC 1.00

SYNGENE SCIENTIFIC SOLUTIONS LT.
S/DC/ARD/01-014

Confidential

FS_SYN2401479_78_G390281-VT
AT 80°C

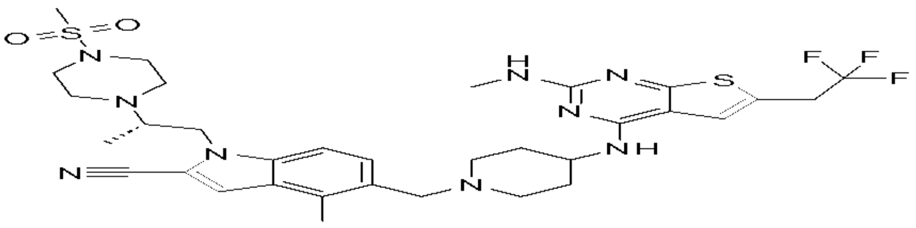

Chemical Formula: $C_{33}H_{42}F_3N_9O_2S_2$
Molecular Weight: 717.88

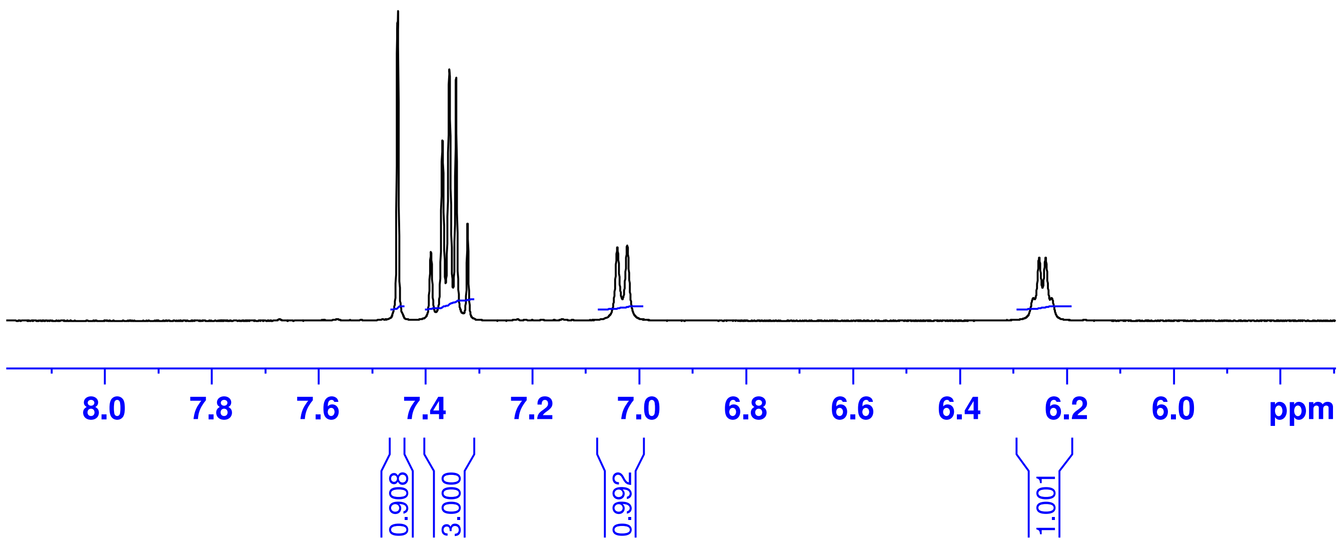

Current Data Parameters
NAME FS_SYN2401479_78_G390281-VT
EXPNO 1
PROCNO 1

F2 - Acquisition Parameters

Date_	20250606
Time	18.41 h
INSTRUM	Avance Neo 400
PROBHD	Z167430_0043 (
PULPROG	zg30
TD	32768
SOLVENT	DMSO
NS	64
DS	0
SWH	8620.689 Hz
FIDRES	0.526165 Hz
AQ	1.9005440 sec
RG	101
DW	58.000 usec
DE	12.45 usec
TE	353.1 K
D1	1.00000000 sec
TD0	1
SFO1	400.2324714 MHz
NUC1	1H
P0	4.00 usec
P1	12.00 usec
PLW1	9.80620003 W

F2 - Processing parameters

SI	65536
SF	400.2300000 MHz
WDW	EM
SSB	0
LB	0.30 Hz
GB	0
PC	1.00

SYNGENE SCIENTIFIC SOLUTIONS LT.
S/DC/ARD/01-014

Syngene Scientific Solutions Ltd
HPLC Report

<Sample Information>

G390281.lcd

Sample Name	: SYN2401479-078-P
Sample ID	: G390281
Data Filename	: G390281.lcd
Method Filename	: X-BRIDGE_ABC_20min.lcm
Batch Filename	: 06JUN2025.lcb
Vial #	: 1-39
Injection Volume	: 1 uL
Date Acquired	: 6/6/2025 10:26:10 AM
Date Processed	: 6/6/2025 1:39:12 PM

<Method Information>

Column :XBridge C18_ABC (4.6X150mm,3.5µm)
MobilephaseA : 10MM ABC in H20
MobilephaseB : ACN
Flowrate :1.2ml/min
Column Temaprature : 35°C
Run Time :20min

Time(min)	%B
0.01	10
2.00	10
12.0	100
17.0	100
18.0	10
20.0	10
20.01	Stop

<Chromatogram>

Chromatogram
G390281.lcd

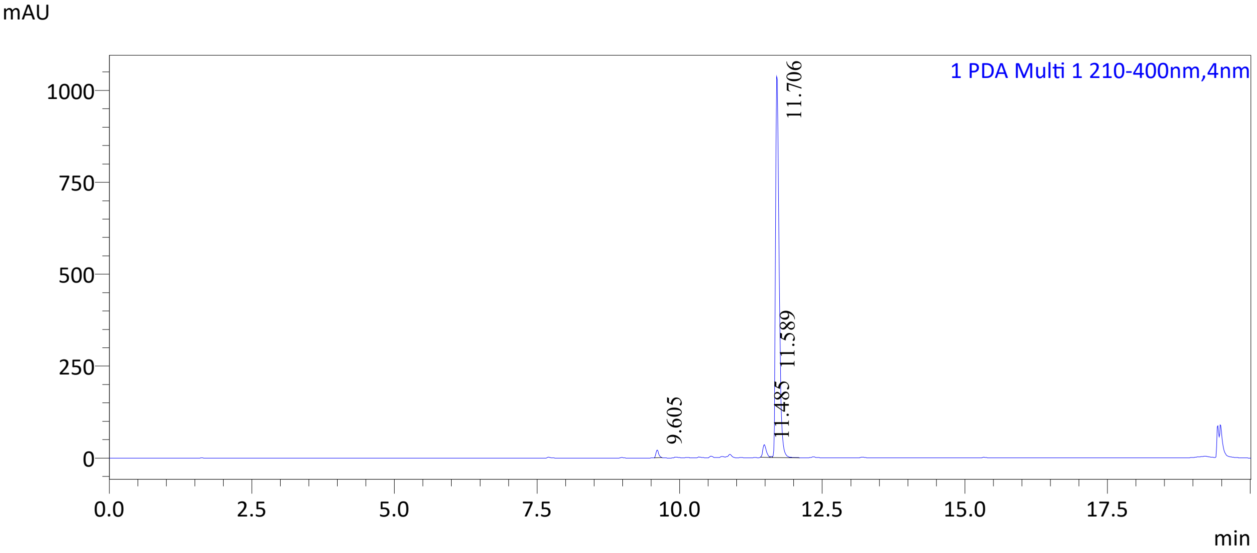

<Peak Table>

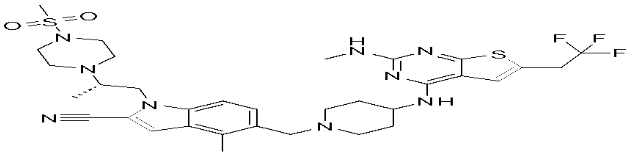

Chemical Formula: $C_{33}H_{42}F_3N_9O_2S_2$
Molecular Weight: 717.88

G390281.lcd

PDA Ch1 210-400nm

Peak#	Ret. Time	Height	Area	Area%
1	9.605	20278	70056	1.486
2	11.485	34282	147607	3.131
3	11.589	2388	5725	0.121
4	11.706	1035916	4490891	95.261
Total		1092863	4714279	100.000

Analysed by

Checked by

Confidential

Syngene scientific solutions Ltd
UPLC-MS Report

Data File name	: G390281.lcd
Method	: PHEN_ABC_4MIN_OCT_NEW.lcm
Sample Name	: SYN2401479-078-P
AR Number	:G390281.lcd
Data Aquired	: 6/6/2025, 1:44:18 PM
Injection Volume	: 1µL
Vial Number	: 94
Instrument ID	: S/DC/ARD/17-050

Method Information

Column	: PhenomenexKinetex EVOC18(3.0x50)mm,2.6 µ	
Mobile phase A	: 5 mM Ammonium Bicarbonate in Water	
Mobile phase B	: ACN	
Column Temparature	: 40.0° C	
Flow rate	: 0.8 mL/min	
Gradient Program	: Time(min)	%B
	0.01	5
	0.20	5
	2.50	95
	3.55	95
	3.60	5
	4.00	5

Chromatogram
D:\2025\JUNE-2025\G390281.lcd

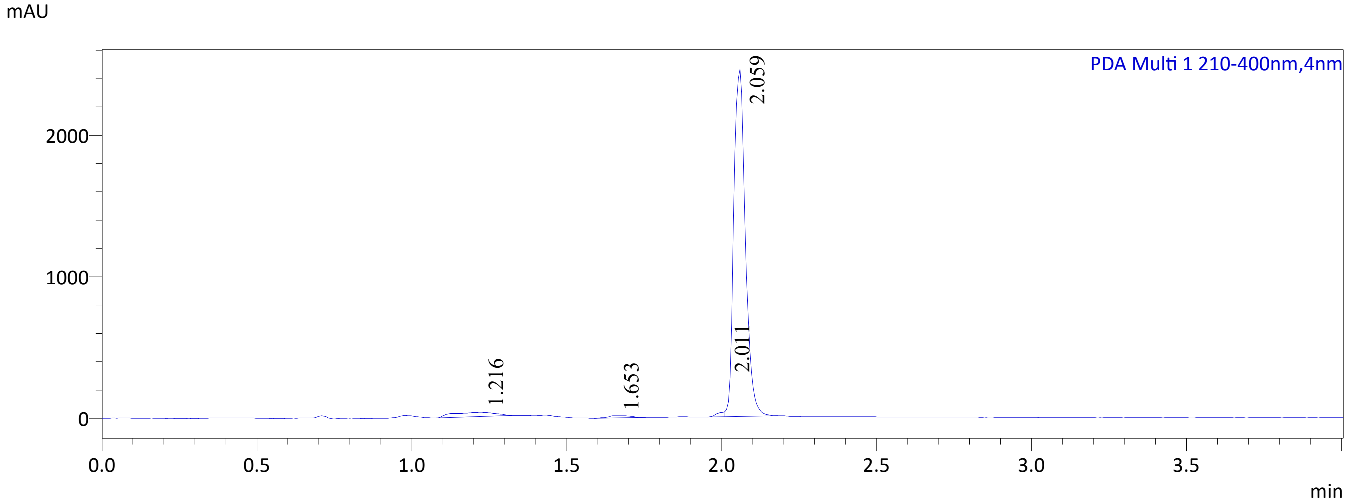

Peak Table G390281.lcd

PDA Ch1 210-400nm

Peak#	Ret. Time	Area	Area%
1	1.216	306038	4.474
2	1.653	79030	1.155
3	2.011	57216	0.836
4	2.059	6398524	93.535
Total		6840809	100.000

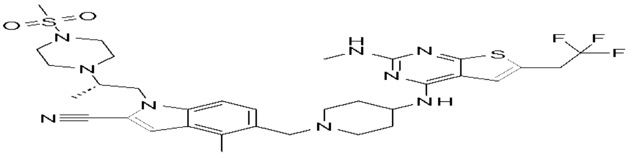

Chemical Formula: $C_{33}H_{42}F_3N_9O_2S_2$
Molecular Weight: 717.88

MS Chromatogram
G390281.lcd

Segment#1

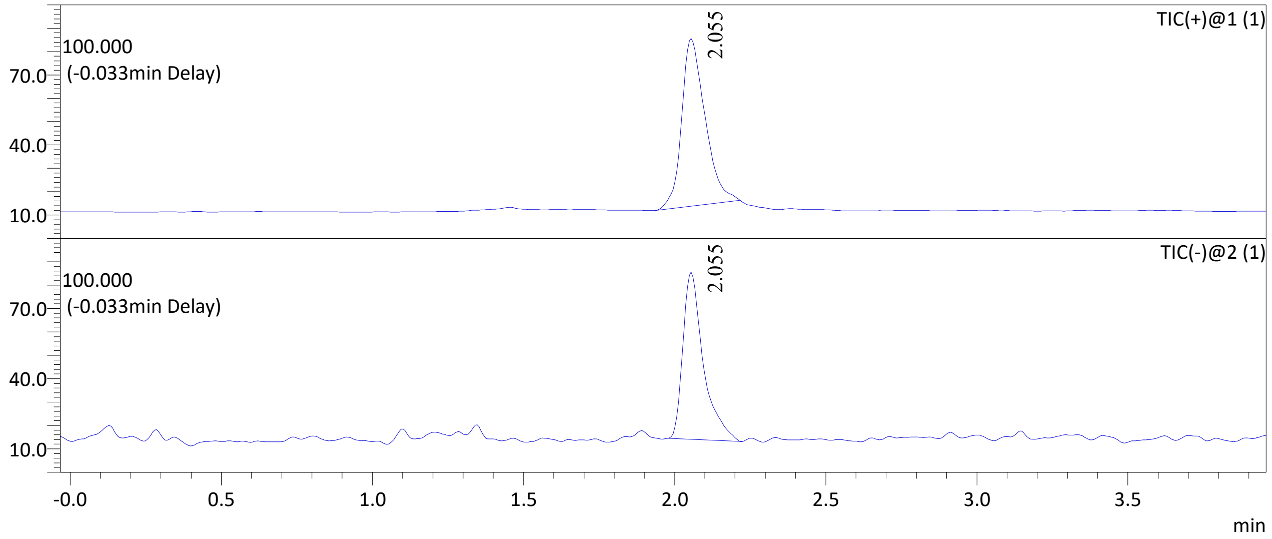

MS Spectrum
G390281.lcd

PeakNo:1
Polarity : Negative
RT : 2.057
Intensity:187607

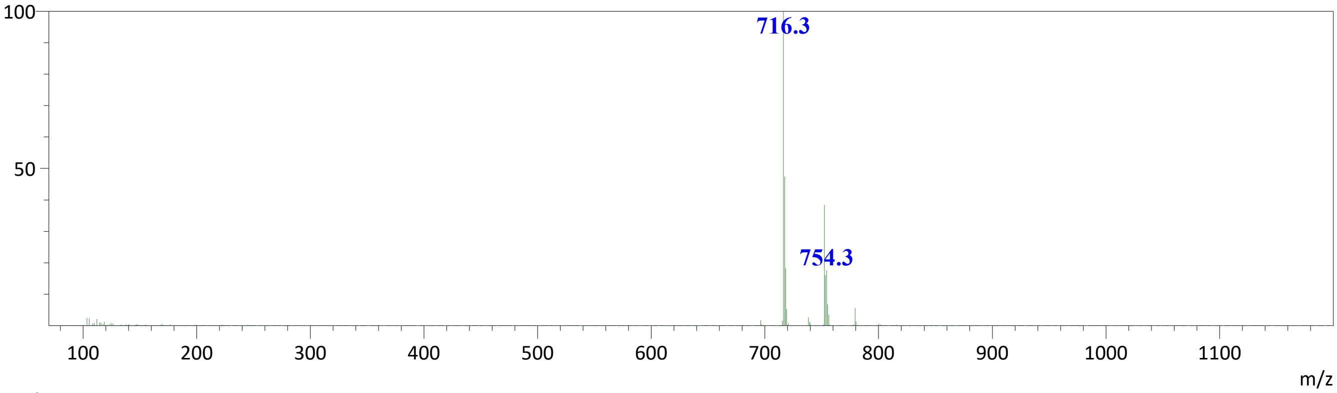

PeakNo:1
Polarity : Positive
RT : 2.055
Intensity:3017857

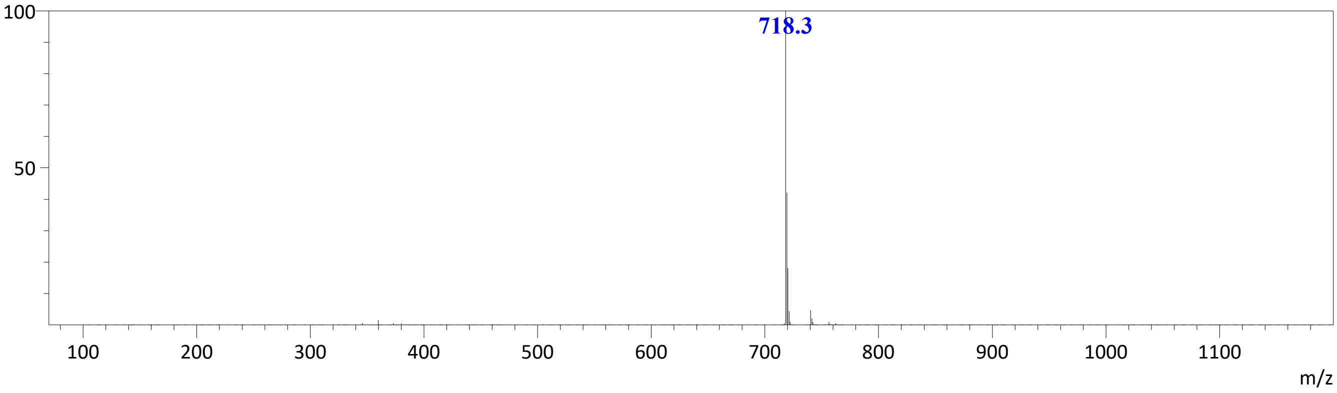

Supplement: Supplementary file 3 — Supplementary Data 1 [file 41467_2026_72685_MOESM3_ESM.zip › SNDX-0060284 (KO-539).pdf]
