# Supplementary material for: CRISPR base editor screening identifies spectrum of MEN1 mutations impacting menin inhibitors in clinical trials
Source: Nat Commun. 2026 May 9;17:6265. doi: 10.1038/s41467-026-72685-1 (PMC13377036; doi:10.1038/s41467-026-72685-1)
Supplement: Supplementary file 3 — Supplementary Data 1 [file 41467_2026_72685_MOESM3_ESM.zip › SNDX-0060363 (DSP-5336).pdf]

CONFIDENTIAL

IS_SYE2000983_7_C718243

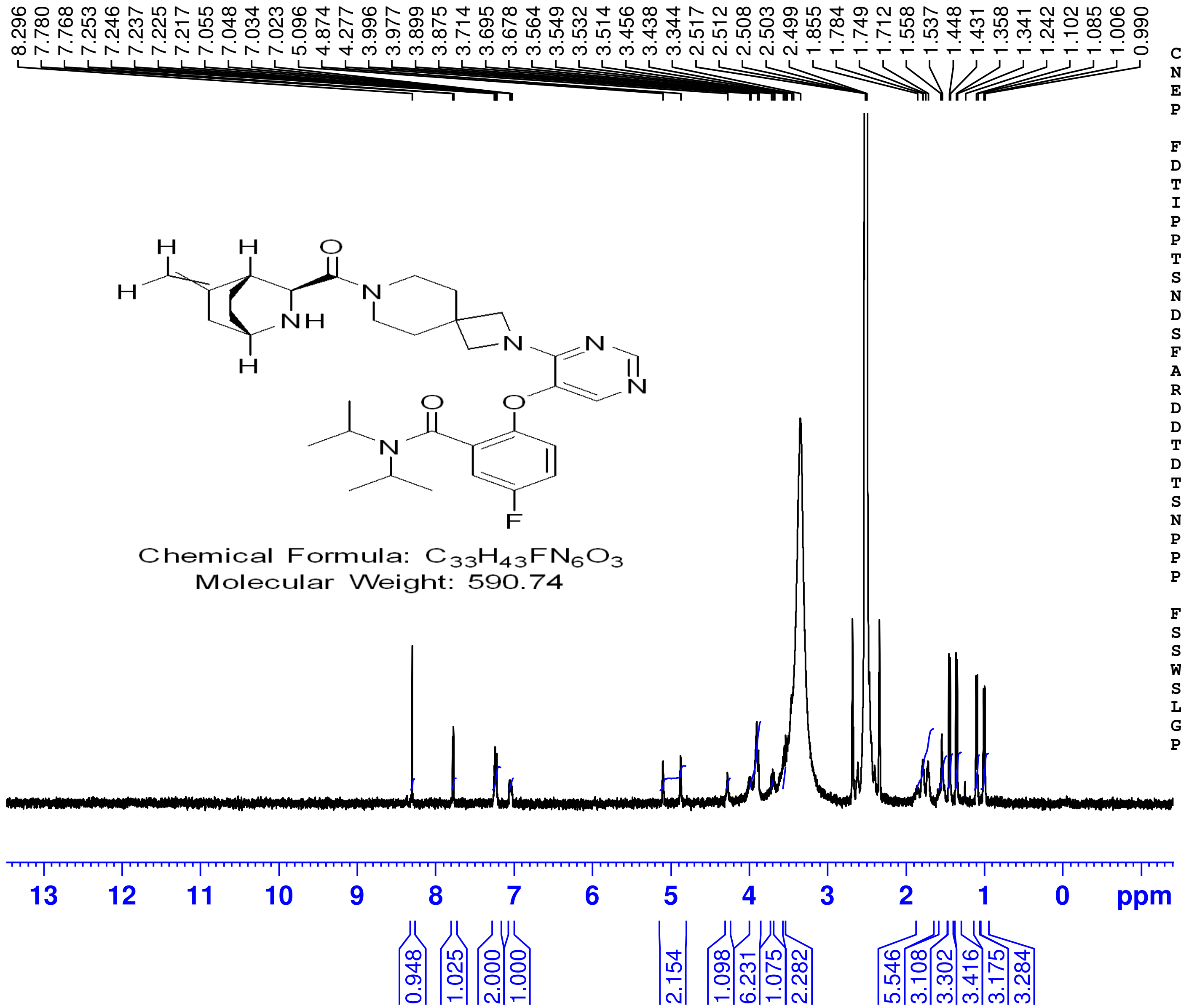

Chemical Formula: $C_{33}H_{43}FN_6O_3$
Molecular Weight: 590.74

BRUKER

Current Data Parameters

NAME	IS_SYE2000983_7_C718243
EXPNO	1
PROCNO	1

F2 - Acquisition Parameters

Date_	20210128
Time	15.45 h
INSTRUM	Avance
PROBHD	Z104450_0002 (
PULPROG	zg30
TD	32768
SOLVENT	DMSO
NS	16
DS	2
SWH	8196.722 Hz
FIDRES	0.500288 Hz
AQ	1.9988480 sec
RG	101
DW	61.000 usec
DE	12.74 usec
TE	-20.7 K
D1	2.00000000 sec
TD0	1
SFO1	400.3724723 MHz
NUC1	1H
P0	4.90 usec
P1	14.70 usec
PLW1	13.30000019 W

F2 - Processing parameters

SI	65536
SF	400.3700000 MHz
WDW	EM
SSB	0
LB	0.30 Hz
GB	0
PC	1.00

SYNGENE INTERNATIONAL LTD.
SC/AD/01-005

CONFIDENTIAL

IS_SYE2000983_7_C718243

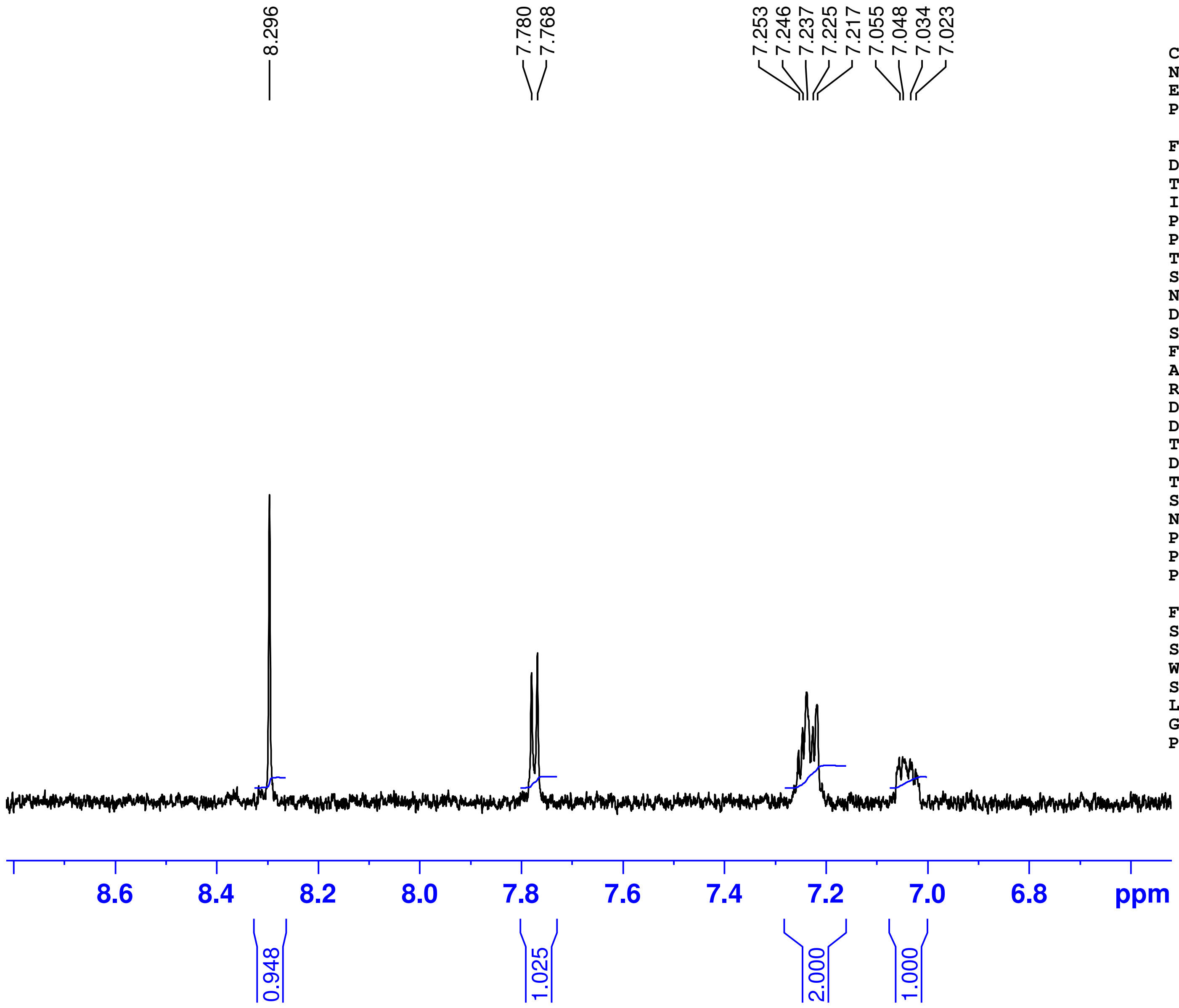

BRUKER

```
Current Data Parameters
NAME      IS_SYE2000983_7_C718243
EXPNO                       1
PROCNO                      1

F2 - Acquisition Parameters
Date_              20210128
Time                  15.45 h
INSTRUM              Avance
PROBHD    Z104450_0002 (
PULPROG                zg30
TD                    32768
SOLVENT                DMSO
NS                       16
DS                        2
SWH                8196.722 Hz
FIDRES             0.500288 Hz
AQ                1.9988480 sec
RG                      101
DW                   61.000 usec
DE                    12.74 usec
TE                    -20.7 K
D1               2.00000000 sec
TD0                       1
SFO1            400.3724723 MHz
NUC1                     1H
P0                     4.90 usec
P1                    14.70 usec
PLW1            13.30000019 W

F2 - Processing parameters
SI                    65536
SF              400.3700000 MHz
WDW                      EM
SSB       0
LB                     0.30 Hz
GB        0
PC                     1.00
```

SYNGENE INTERNATIONAL LTD.
SC/AD/01-005

CONFIDENTIAL

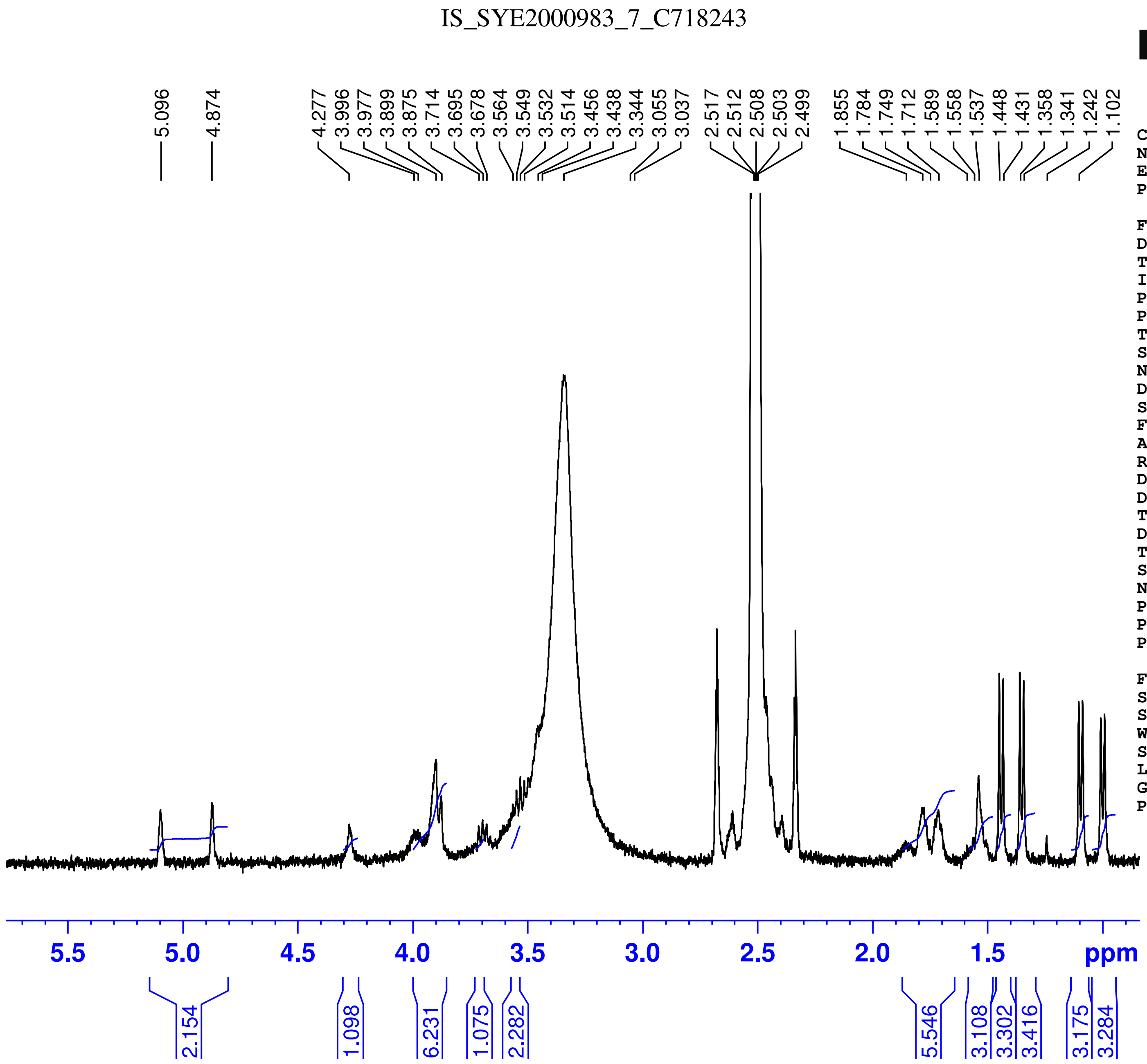

Current Data Parameters
NAME IS_SYE2000983_7_C718243
EXPNO 1
PROCNO 1

F2 - Acquisition Parameters

Date_	20210128	
Time	15.45	h
INSTRUM	Avance	
PROBHD	Z104450_0002 (	
PULPROG	zg30	
TD	32768	
SOLVENT	DMSO	
NS	16	
DS	2	
SWH	8196.722	Hz
FIDRES	0.500288	Hz
AQ	1.9988480	sec
RG	101	
DW	61.000	usec
DE	12.74	usec
TE	-20.7	K
D1	2.00000000	sec
TD0	1	
SFO1	400.3724723	MHz
NUC1	1H	
P0	4.90	usec
P1	14.70	usec
PLW1	13.30000019	W

F2 - Processing parameters

SI	65536	
SF	400.3700000	MHz
WDW	EM	
SSB	0	
LB	0.30	Hz
GB	0	
PC	1.00	

SYNGENE INTERNATIONAL LTD.
SC/AD/01-005

Data file	:C:\CHEM32\1\DATA\Y2021\JAN\28012021 3\C718243.D
Acq Method	:C:\Chem32\1\Methods\XB_0595TF.M
Injection Date	:28-Jan-2021
Vial No.	: P1-C4
Injection Time	:12:28:16
Injection vol	:4ul
Sample Name	:IS_SYE2000983_7_C718243

Method info : Column:X-Bridge C8(50X4.6)mm,3.5µm
Mobile phase:A:0.1% TFA in water
Mobile phase:B:ACN,
Flow:2.0mL/min

TIME(min)	%B
0.0	05
8.0	100
8.1	100
8.5	05
10.0	05

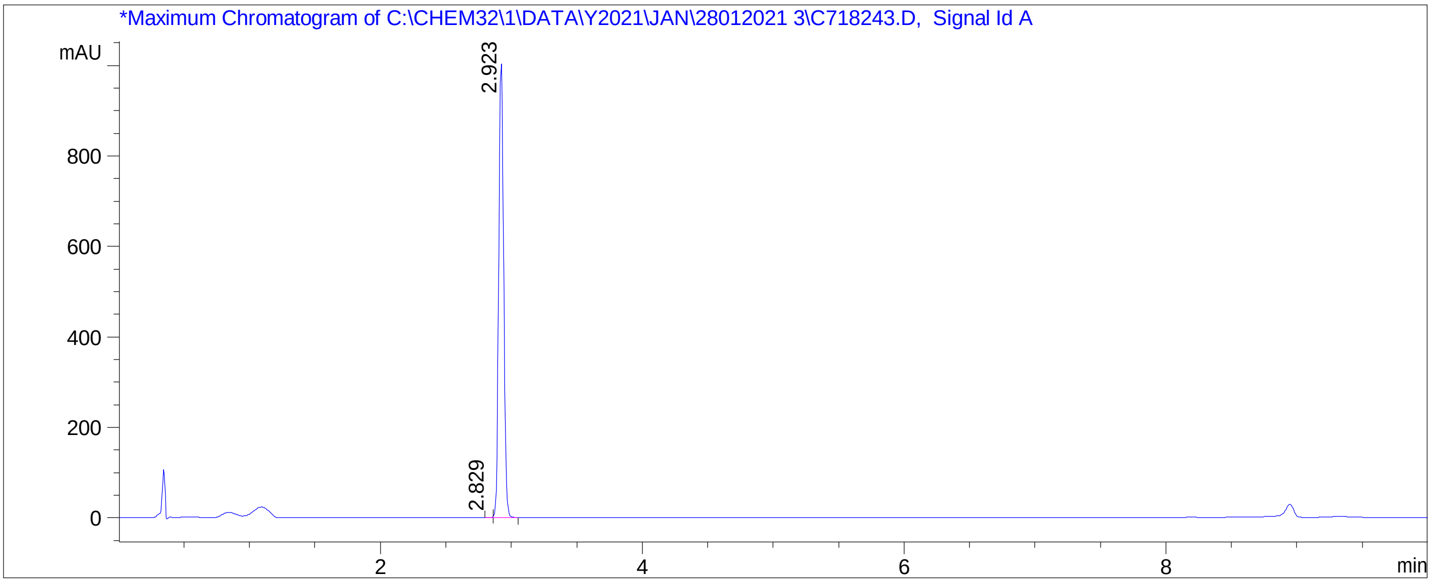

Peak	RT min	Height	Area	Area %
1	2.829	1.698	2.183	0.082
2	2.923	1.010e3	2663.234	99.918

End of report

Analysed By :

Checked by :

Instrument Code : S_DC_ARD_04_134

Data file	:	D:\DATA\Y2021\JAN2021\28012021 4\C718243.D		
Acq Method	:	D:\DATA\Y2021\JAN2021\28012021 4\ZX_595FA.M		
Injection Date	:	28 -Jan-2021	Vial No.	: P1-C8
Injection Time	:	12:32:04	Injection vol	: 1.0 µL
Sample Name	:	Analysis of J1321-13753		

Method info : Column :ZORBAX XDB C18 (50x4.6mm) 5 µm
Mobile phase :A :0.1% HCOOH in H2O:ACN (95:5)
Mobile phase: B: ACN
Flow Rate :1.5ml/min

Time (min)	%B
0.0	5
2.5	95
4.0	95
4.5	5
5.5	5

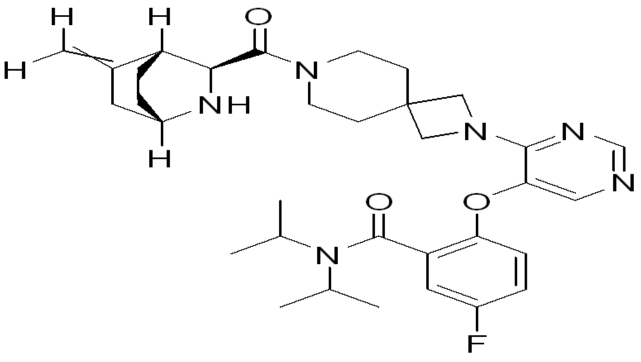

Chemical Formula: $C_{33}H_{43}FN_6O_3$
Molecular Weight: 590.74

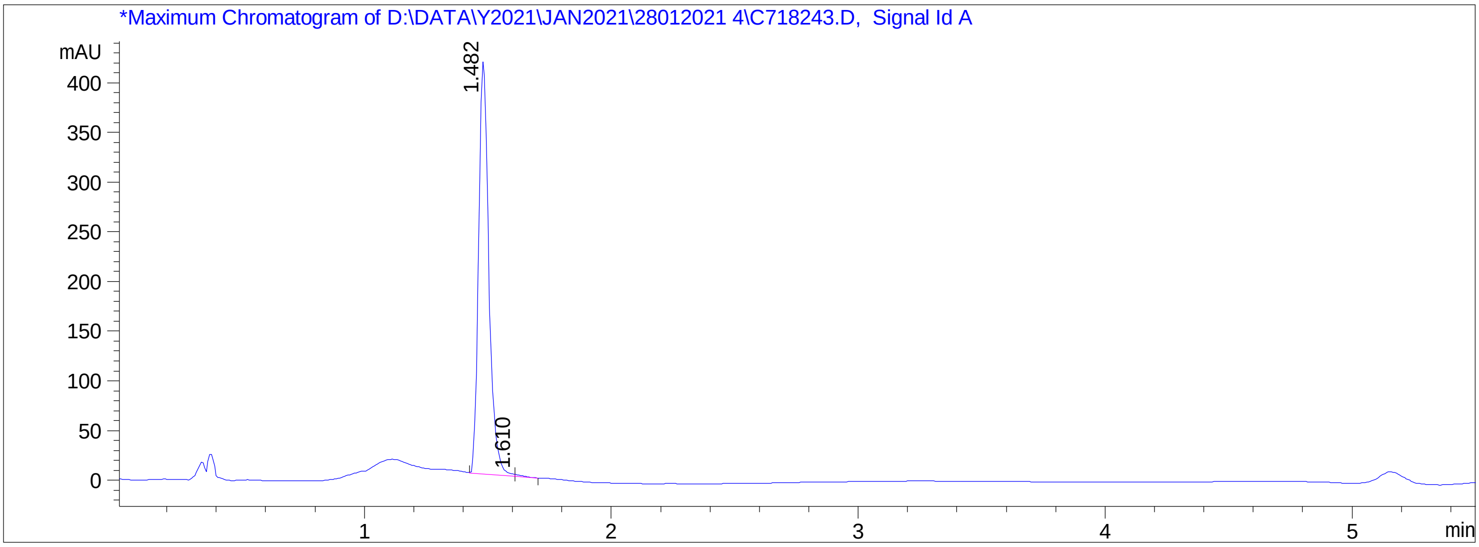

Peak No	RT min	Area	Area %
1	1.482	1203.205	99.644
2	1.610	4.300	0.356

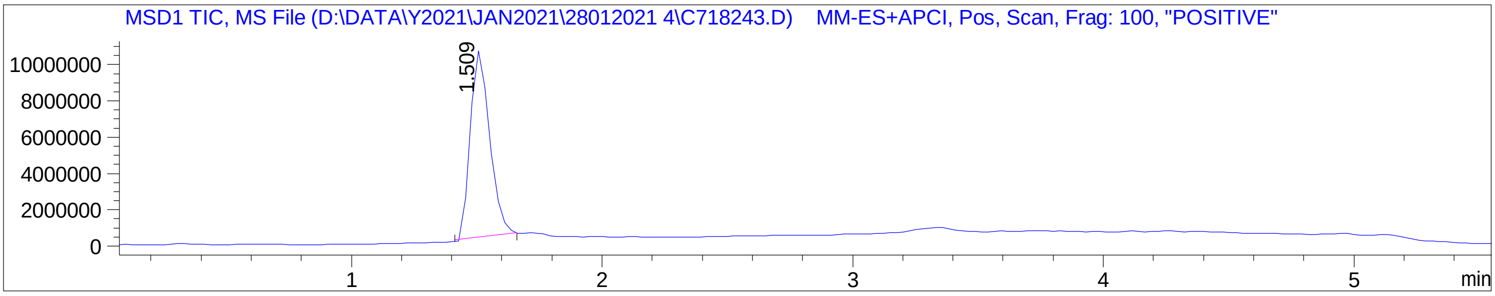

Analysed by : Checked by:

Instrument Code : S-DC-ARD-17-19

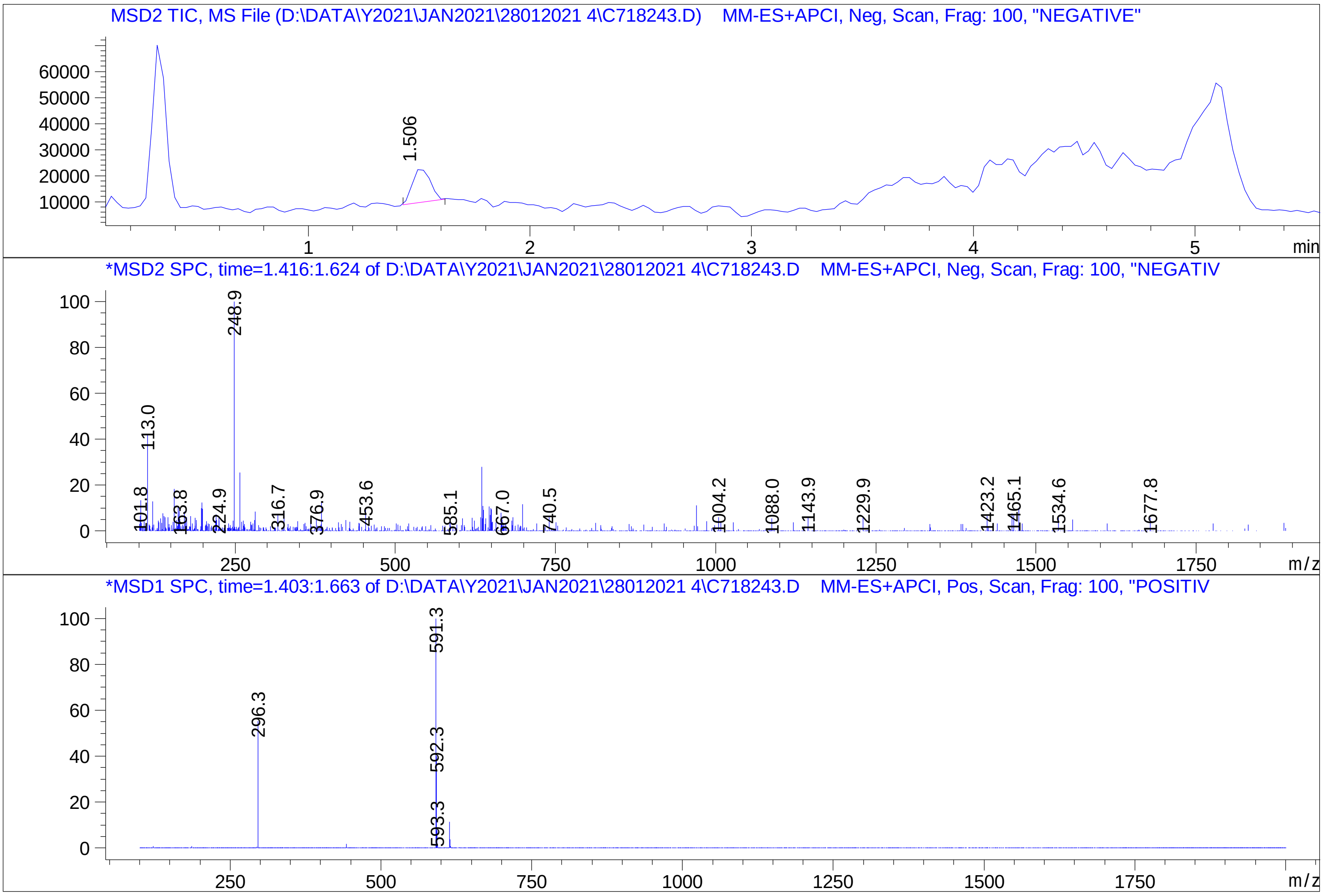

Analysed by : Checked by:

Instrument Code : S-DC-ARD-17-19
