# Supplementary material for: CRISPR base editor screening identifies spectrum of MEN1 mutations impacting menin inhibitors in clinical trials
Source: Nat Commun. 2026 May 9;17:6265. doi: 10.1038/s41467-026-72685-1 (PMC13377036; doi:10.1038/s41467-026-72685-1)
Supplement: Supplementary file 3 — Supplementary Data 1 [file 41467_2026_72685_MOESM3_ESM.zip › SNDX-0060867 (JNJ-75276617).pdf]

CONFIDENTIAL

FS_SYE2101692_24_D315233

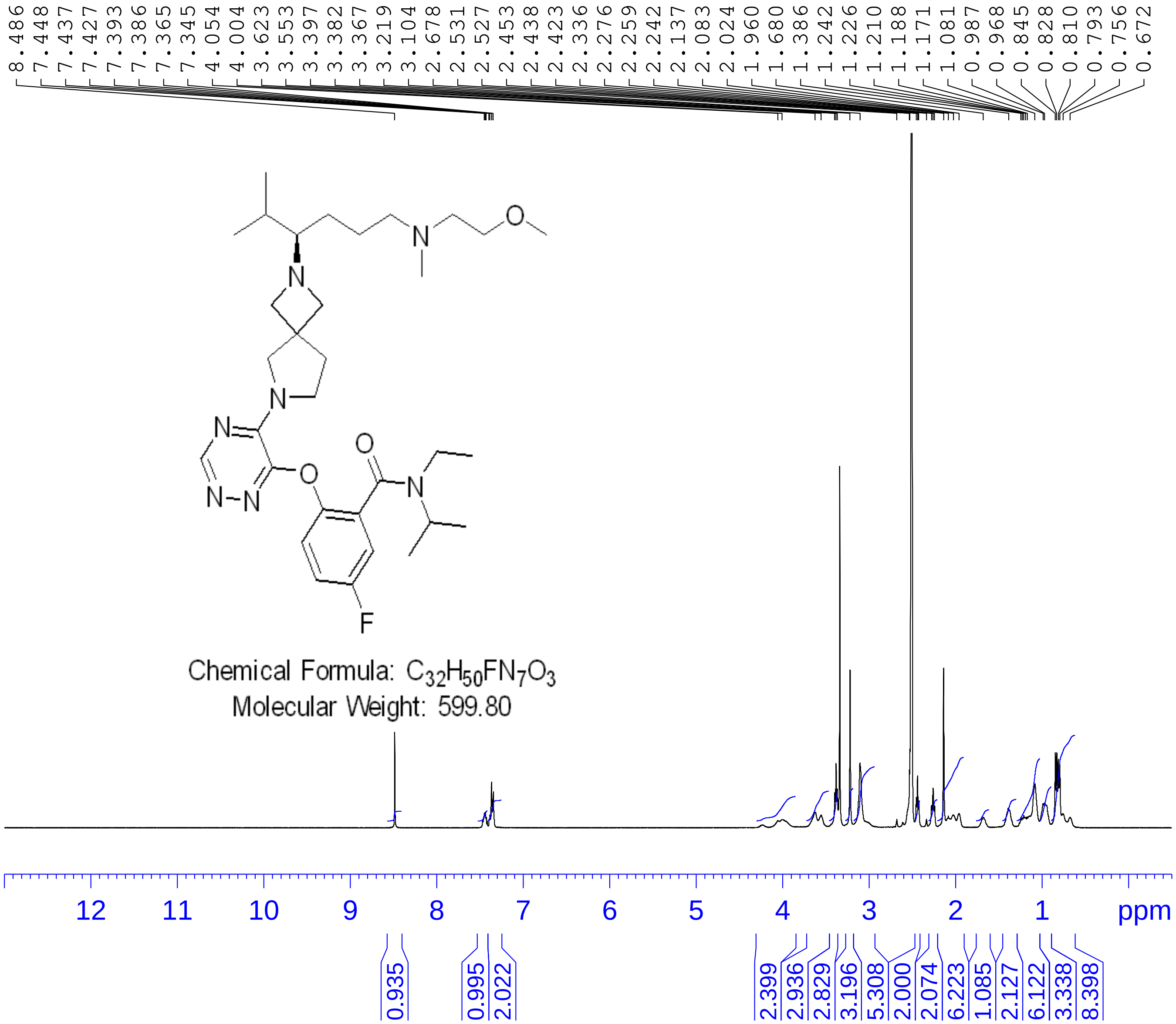

BRUKER

Current Data Parameters

NAME	FS_SYE2101692_24_D315233
EXPNO	1
PROCNO	1

F2 - Acquisition Parameters

Date_	20211126
Time	16.39
INSTRUM	spect
PROBHD	5 mm PABBO BB-
PULPROG	zg30
TD	32768
SOLVENT	DMSO
NS	16
DS	2
SWH	8012.820 Hz
FIDRES	0.244532 Hz
AQ	2.0447233 sec
RG	456
DW	62.400 usec
DE	6.50 usec
TE	298.0 K
D1	2.00000000 sec
TD0	1

======== CHANNEL f1 ========

SFO1	400.3124721 MHz
NUC1	1H
P1	14.50 usec
PLW1	14.00000000 W

F2 - Processing parameters

SI	65536
SF	400.3100000 MHz
WDW	EM
SSB	0
LB	0.30 Hz
GB	0
PC	1.00

SYNGENE INTERNATIONAL LTD
SC/AD/01-004

CONFIDENTIAL

FS_SYE2101692_24_D315233

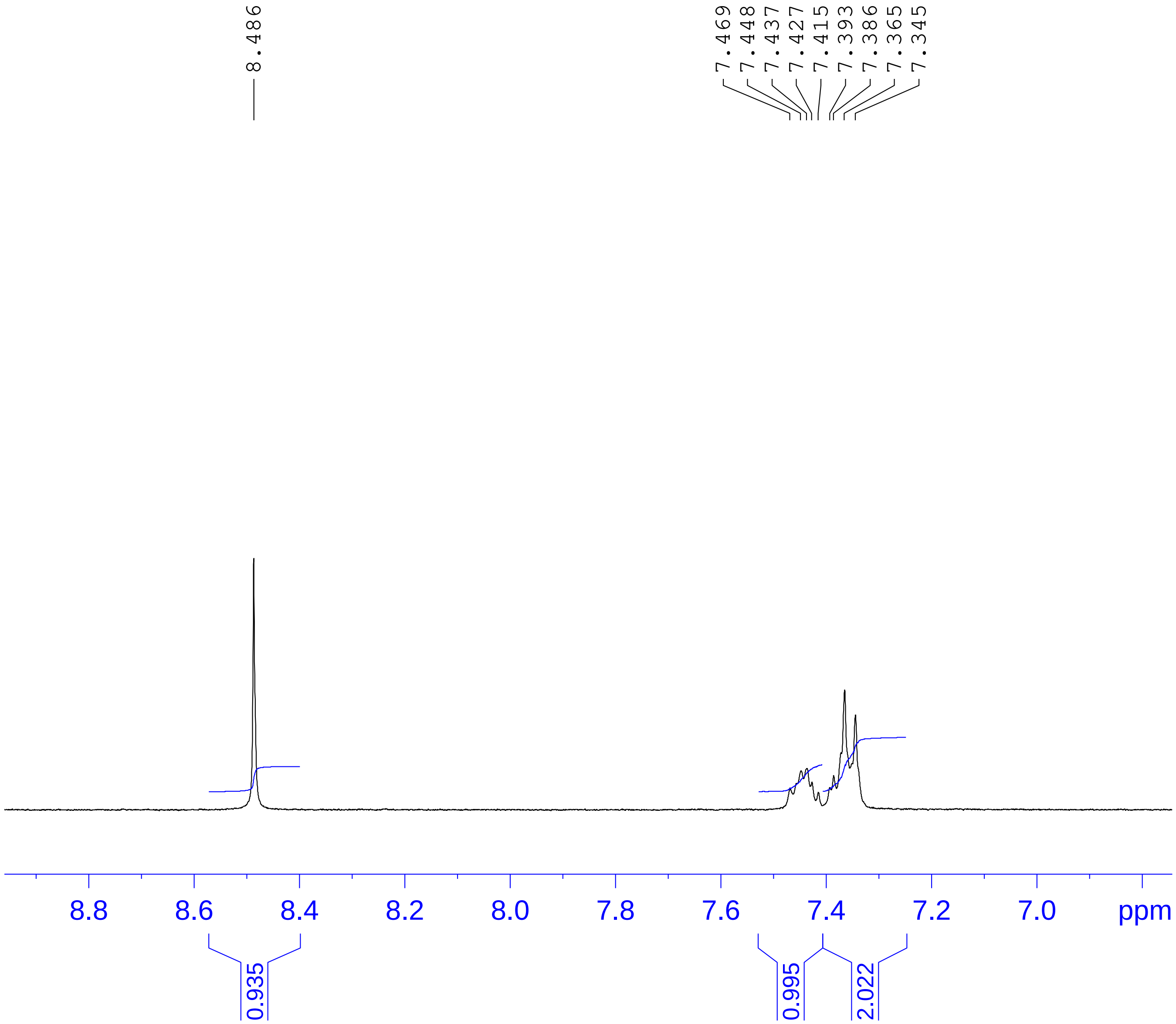

BRUKER

Current Data Parameters
NAME FS_SYE2101692_24_D315233
EXPNO 1
PROCNO 1

F2 - Acquisition Parameters
Date_ 20211126
Time 16.39
INSTRUM spect
PROBHD 5 mm PABBO BB-
PULPROG zg30
TD 32768
SOLVENT DMSO
NS 16
DS 2
SWH 8012.820 Hz
FIDRES 0.244532 Hz
AQ 2.0447233 sec
RG 456
DW 62.400 usec
DE 6.50 usec
TE 298.0 K
D1 2.00000000 sec
TD0 1

======== CHANNEL f1 ========
SFO1 400.3124721 MHz
NUC1 1H
P1 14.50 usec
PLW1 14.00000000 W

F2 - Processing parameters
SI 65536
SF 400.3100000 MHz
WDW EM
SSB 0
LB 0.30 Hz
GB 0
PC 1.00

SYNGENE INTERNATIONAL LTD
SC/AD/01-004

CONFIDENTIAL

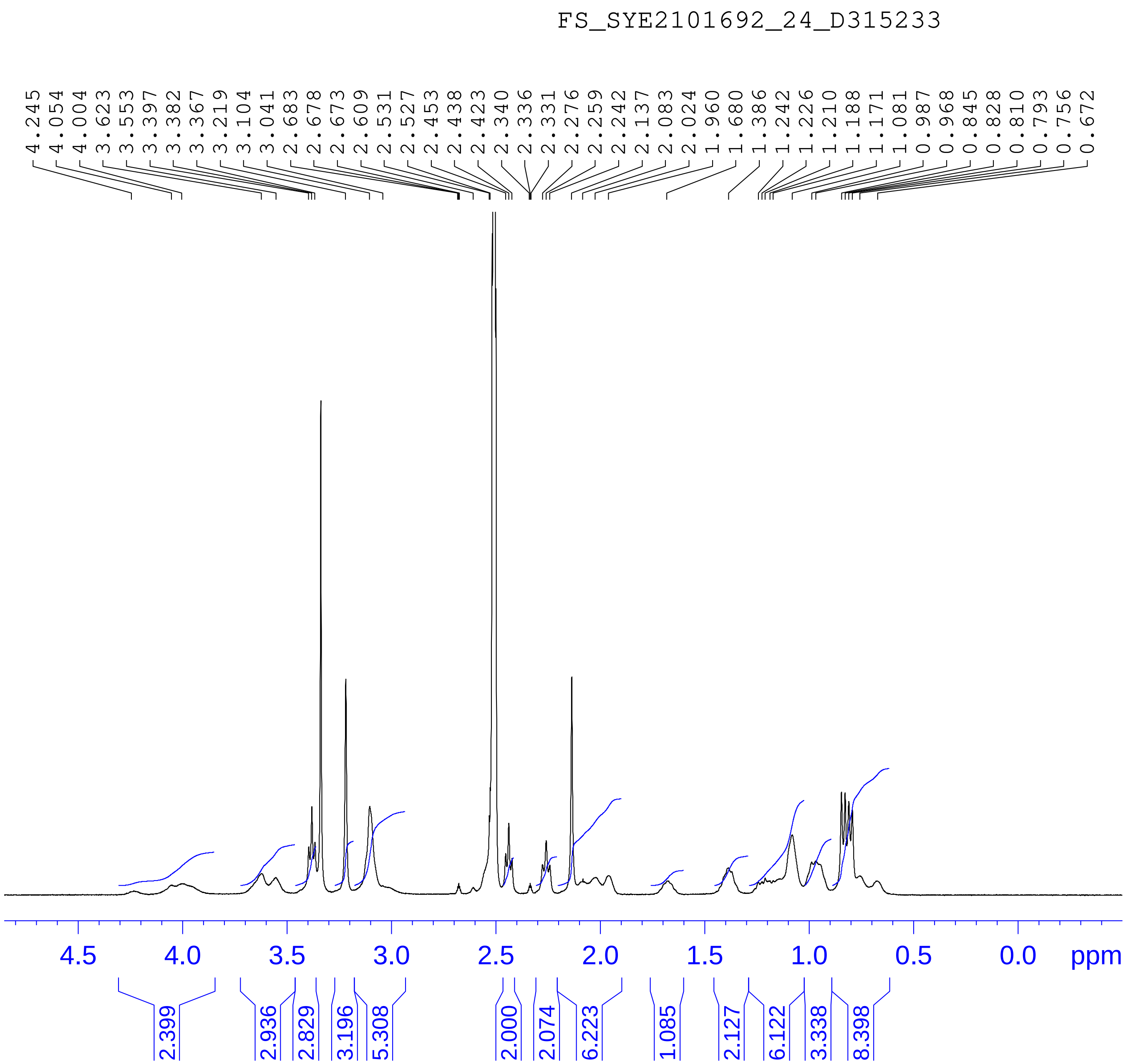


```
Current Data Parameters
NAME      FS_SYE2101692_24_D315233
EXPNO                    1
PROCNO                   1

F2 - Acquisition Parameters
Date_             20211126
Time                 16.39
INSTRUM              spect
PROBHD    5 mm PABBO BB-
PULPROG               zg30
TD                   32768
SOLVENT               DMSO
NS                      16
DS                       2
SWH               8012.820 Hz
FIDRES            0.244532 Hz
AQ               2.0447233 sec
RG                     456
DW                  62.400 usec
DE                    6.50 usec
TE                   298.0 K
D1              2.00000000 sec
TD0                      1

======== CHANNEL f1 ========
SFO1           400.3124721 MHz
NUC1                    1H
P1                   14.50 usec
PLW1           14.00000000 W

F2 - Processing parameters
SI                   65536
SF             400.3100000 MHz
WDW                     EM
SSB       0
LB                    0.30 Hz
GB        0
PC                    1.00
```

SYNGENE INTERNATIONAL LTD
SC/AD/01-004

Data file	:C:\CHEM32\1\DATA\Y2021\NOV\26112021 2021-11-26 14-58-29\D315233a.D
Acq Method	:C:\CHEM32\1\METHODS\XB_0595TF.M
Injection Date	:26- Nov-2021
Injection time	:15:42:20
Sample Name	:jnj-27
Vial No.	: P2-D-09
Injection vol	: 2.0 µl

Method info : Column: X-Bridge C8 (50 x 4.6) mm 3.5µm
Mobile Phase 'A':0.1% TFA in Water
Mobile Phase 'B':ACN
Flow :2.0 ml\min

Time(min)	%B
0.0	05
8.0	100
8.1	100
8.5	05
10.0	05

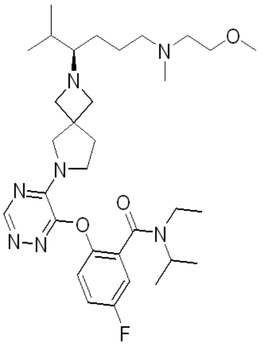

Chemical Formula: $C_{32}H_{50}FN_7O_3$
Molecular Weight: 599.80

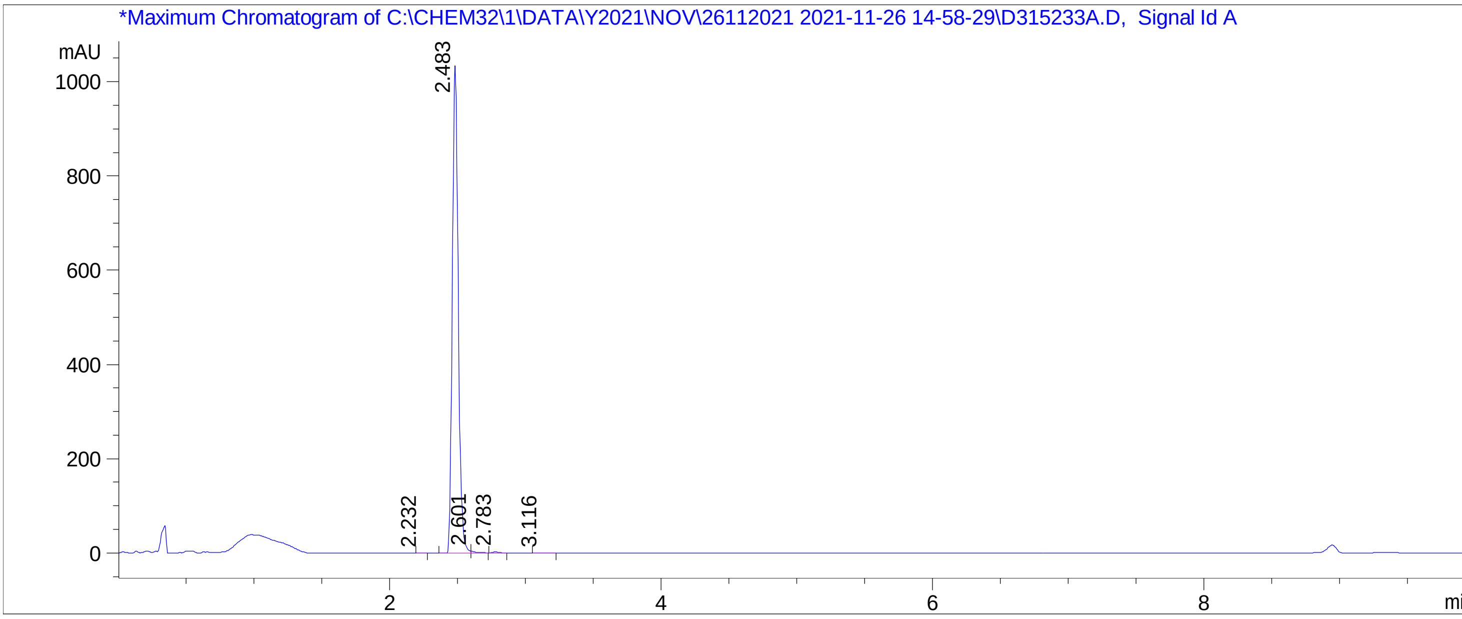

Peak	RT min	Height	Area	Area %
1	2.232	0.923	2.136	0.067
2	2.483	1.040e3	3148.423	99.249
3	2.601	3.977	9.753	0.307
4	2.783	2.287	7.578	0.239
5	3.116	0.943	4.365	0.138

End of report

Analysed By :

Checked By :

Data file : D:\DATA\Y2021\NOV2021\26112021 6\D315233.D
Acq Method : D:\DATA\Y2021\NOV2021\26112021 6\ZX_595FA.M
Injection Date : 26 -Nov-2021 Vial No. : P1-D4
Injection Time : 15:35:51 Injection vol : 1.0 µL
Sample Name : jnj-27

Method info : Column :ZORBAX XDB C18 (50x4.6mm) 3.5 µm
Mobile phase :A :0.1% HCOOH in H2O
Mobile phase: B: ACN
Flow Rate :1.5ml/min

Time (min)	%B
0.0	5
2.5	95
4.0	95
4.5	5
5.5	5

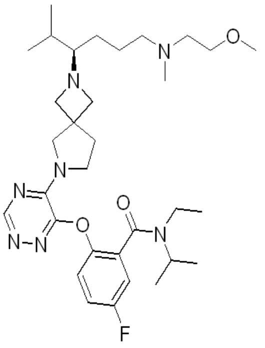

Chemical Formula: $C_{32}H_{50}FN_7O_3$
Molecular Weight: 599.80

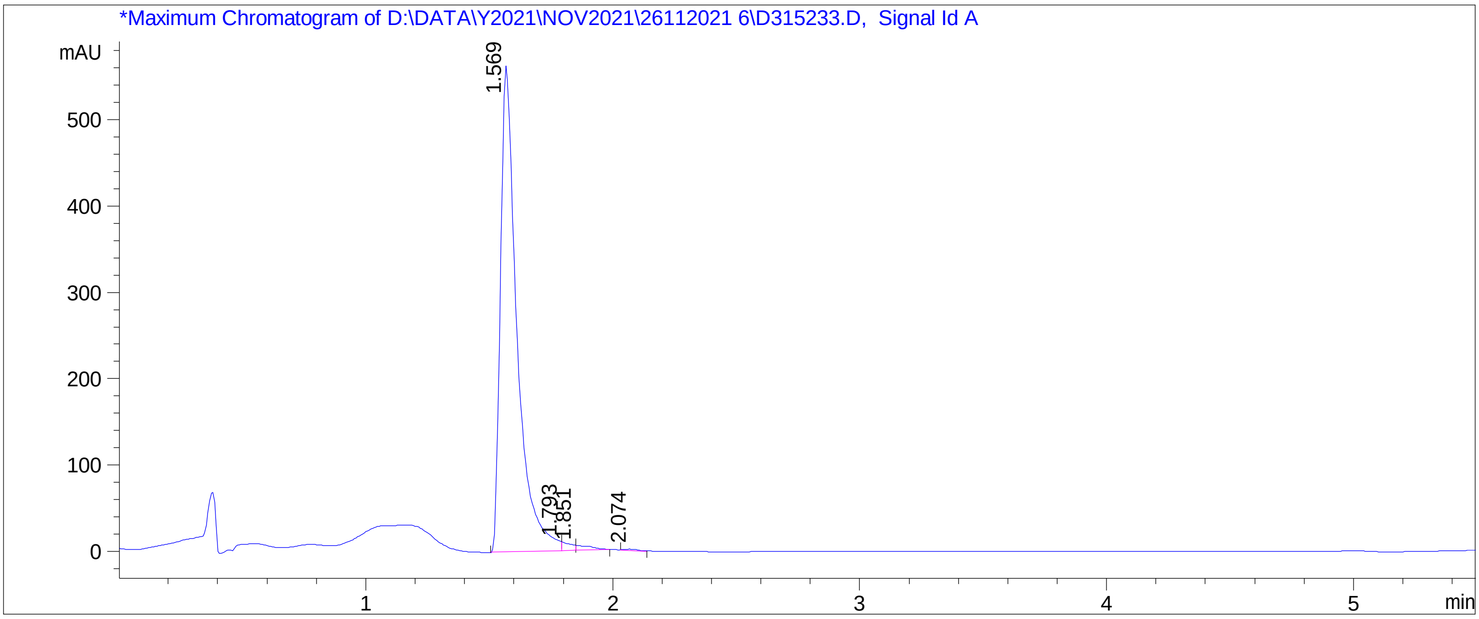

Peak No	RT min	Area	Area %
1	1.569	2.626e3	97.978
2	1.793	25.725	0.960
3	1.851	22.774	0.850
4	2.074	5.691	0.212

Analysed by : Checked by:

Instrument Code : S-DC-ARD-17-19

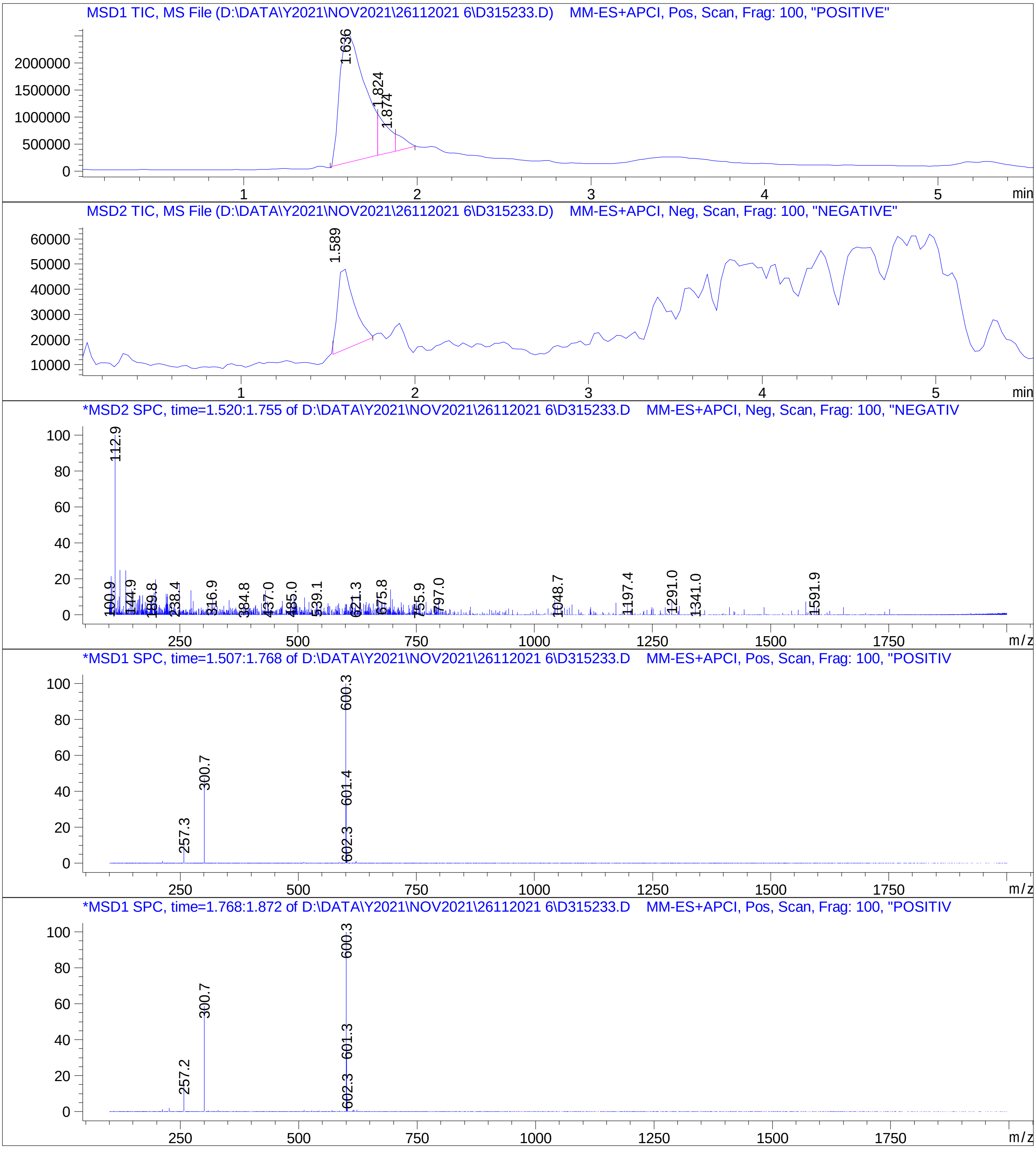

Analysed by : Checked by:

Instrument Code : S-DC-ARD-17-19

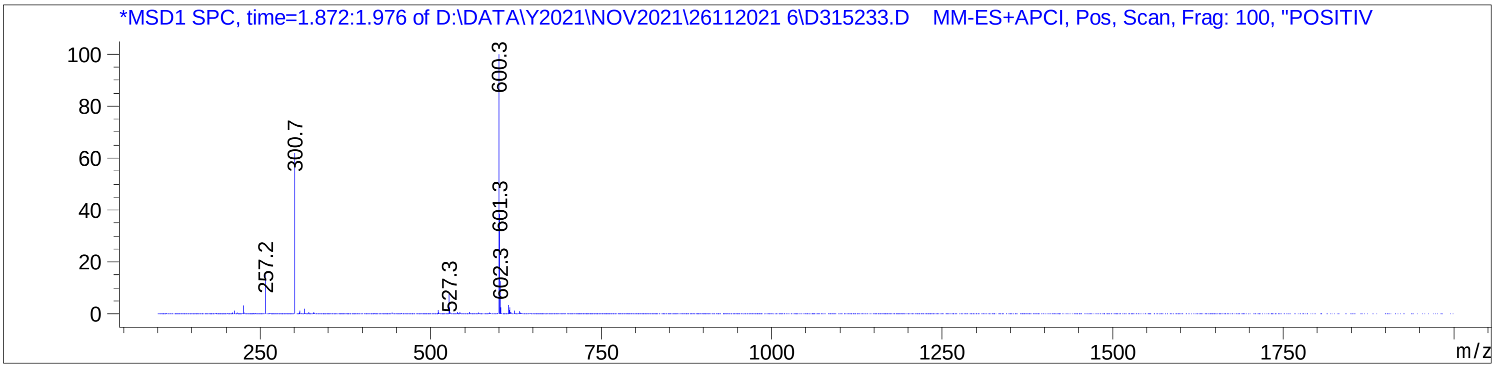

Analysed by :

Checked by:

Instrument Code : S-DC-ARD-17-19

Confidential
SFC Analytical Chromatogram

Syngene

Date : 26 Nov 2021 - 23:14	Username :
Method : C:\Pic Solution\Méthode\Analytical\SFC-5-45.met	
Data : C:\Pic Solution\Data\2021\NOV2021\26NOV21\26NOV21A\D315233_010.dat	
Comments :	
**Elution**	**Injection**
FlowRate : 5 ml/min	Column Name : Lux C2
Co-Solvent : 45%	Sample Name : LB6 : D315233
Co-Solvent Name : 0.5% Isopropylamine in Methanol	Injected Volume : 15 µl
Outlet Pressure: 100 bar	Temperature : 35 °C

Note: This compound was analysed using above method and found as single peak. It does not confirm that the compound has only single isomer. A racemic mixture or reference method need to be provided to confirm the presence of single isomer.

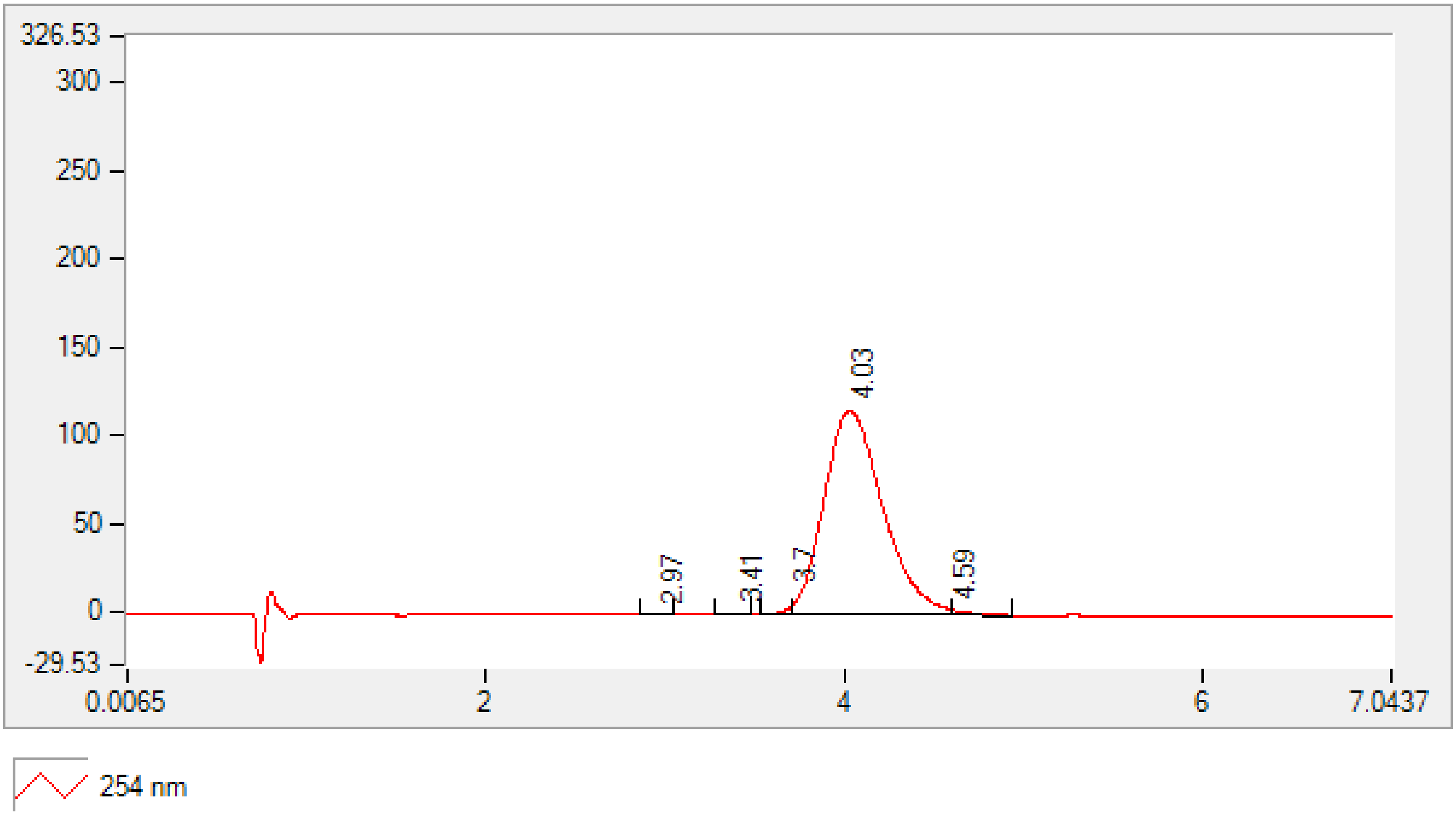

Results UV3			
Sr.No.	RT	Area	Area %
1	2.97	5.653	0.211
2	3.41	2.069	0.077
3	3.70	10.835	0.404
4	4.03	2643.696	98.473
5	4.59	22.429	0.835

Analysed By:	Instrument Code: S/DC/ARD/22-009	Checked By:
