# Supplementary material for: CRISPR base editor screening identifies spectrum of MEN1 mutations impacting menin inhibitors in clinical trials
Source: Nat Commun. 2026 May 9;17:6265. doi: 10.1038/s41467-026-72685-1 (PMC13377036; doi:10.1038/s41467-026-72685-1)
Supplement: Supplementary file 8 — Supplementary Data 6 [file 41467_2026_72685_MOESM8_ESM.pptx]

## Slide 1
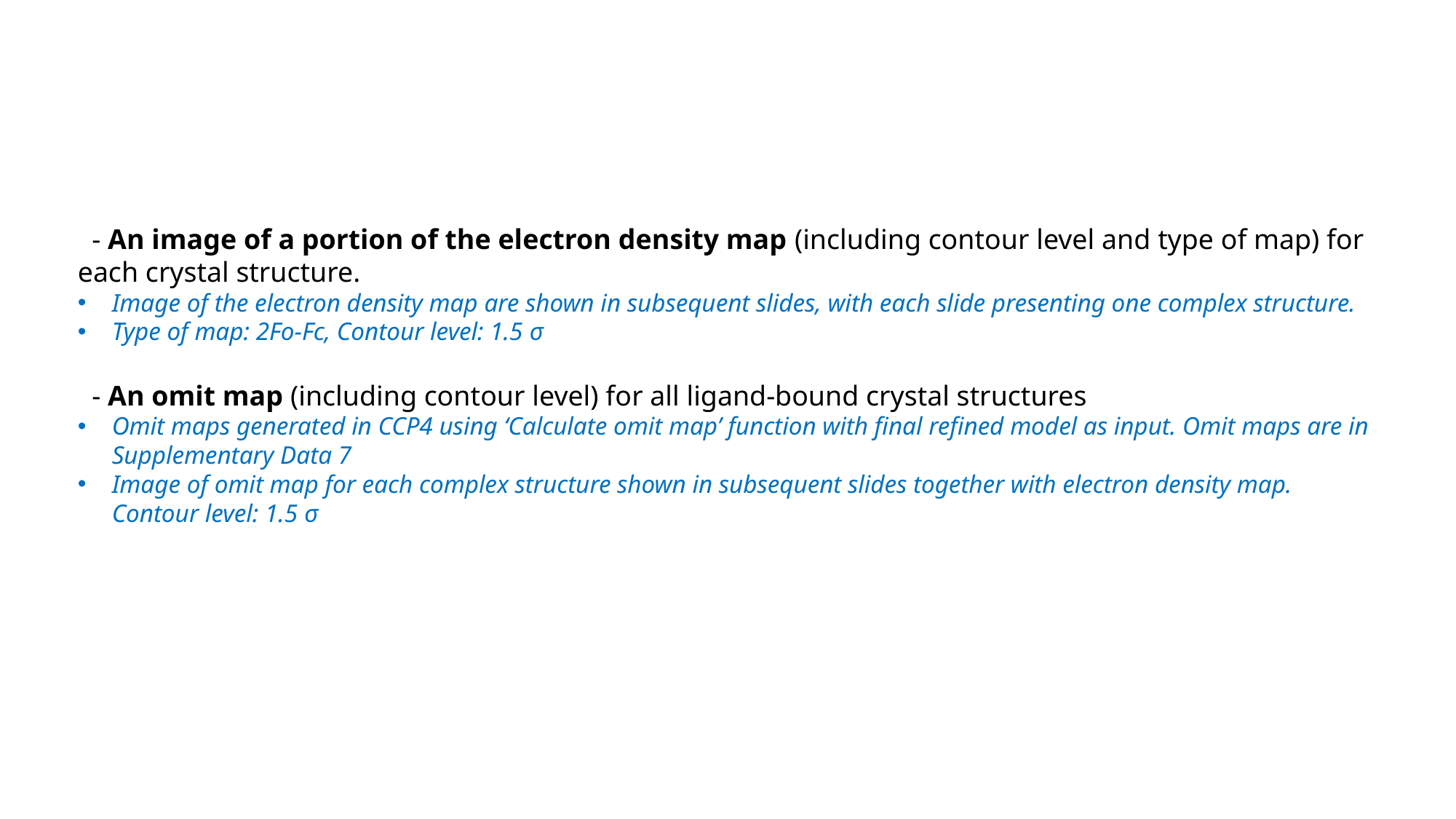

- An image of a portion of the electron density map (including contour level and type of map) for each crystal structure.
Image of the electron density map are shown in subsequent slides, with each slide presenting one complex structure.
Type of map: 2Fo-Fc, Contour level: 1.5 σ
  - An omit map (including contour level) for all ligand-bound crystal structures
Omit maps generated in CCP4 using ‘Calculate omit map’ function with final refined model as input. Omit maps are in Supplementary Data 7
Image of omit map for each complex structure shown in subsequent slides together with electron density map. Contour level: 1.5 σ

## Slide 2
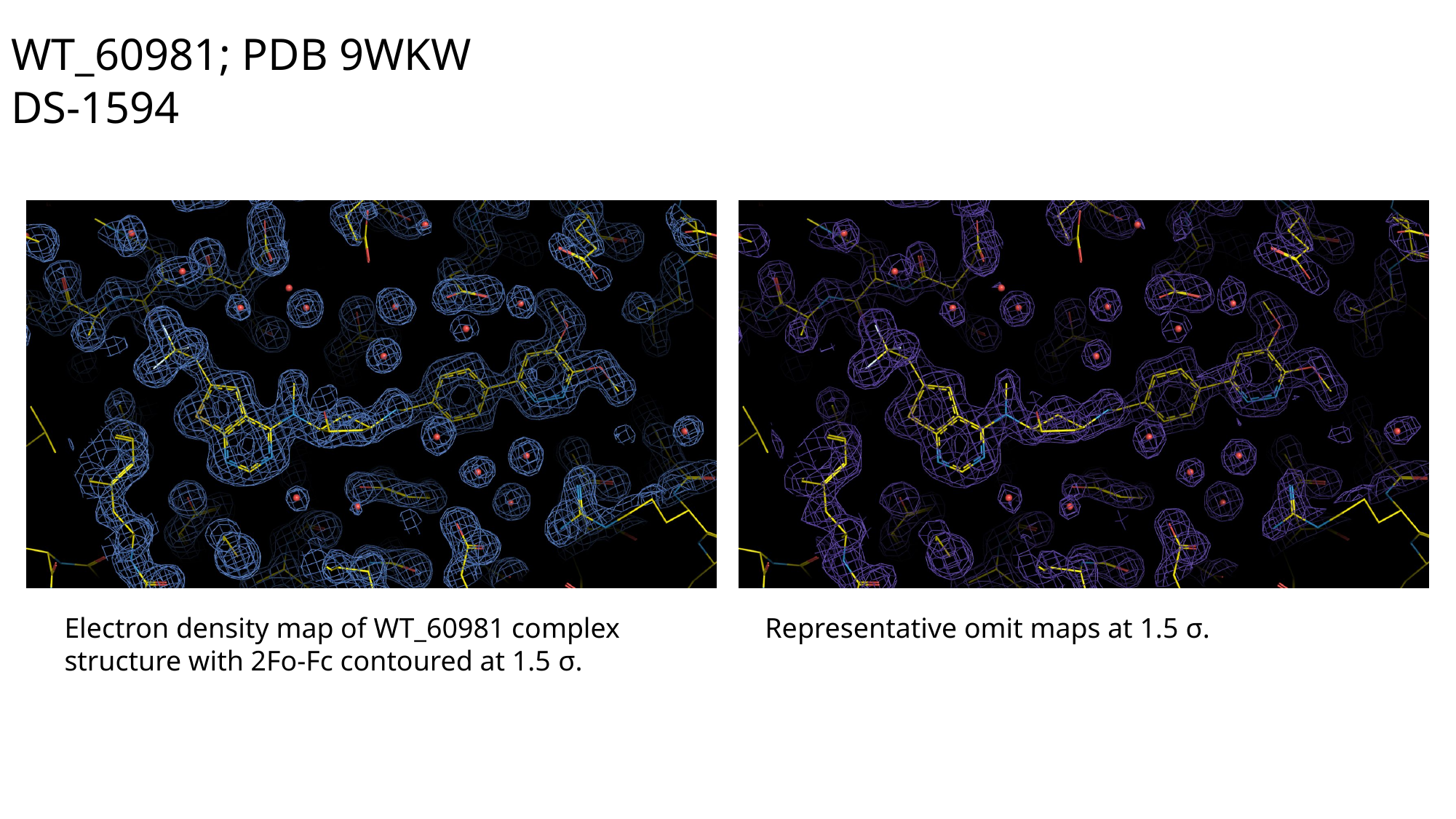

WT_60981; PDB 9WKW
DS-1594
 Representative omit maps at 1.5 σ.
Electron density map of WT_60981 complex structure with 2Fo-Fc contoured at 1.5 σ.

## Slide 3
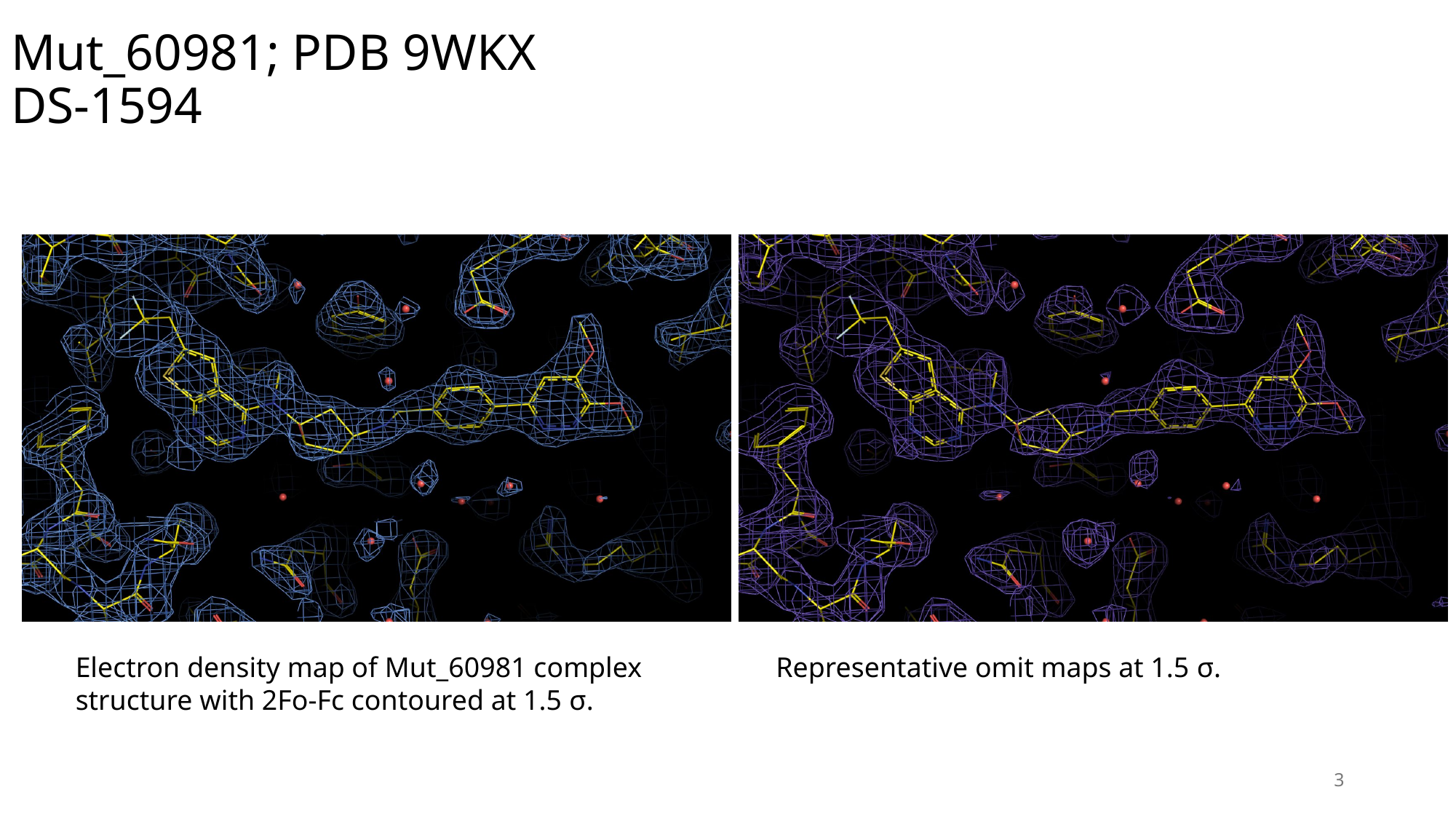

Mut_60981; PDB 9WKX
DS-1594
 Representative omit maps at 1.5 σ.
Electron density map of Mut_60981 complex structure with 2Fo-Fc contoured at 1.5 σ.
3

## Slide 4
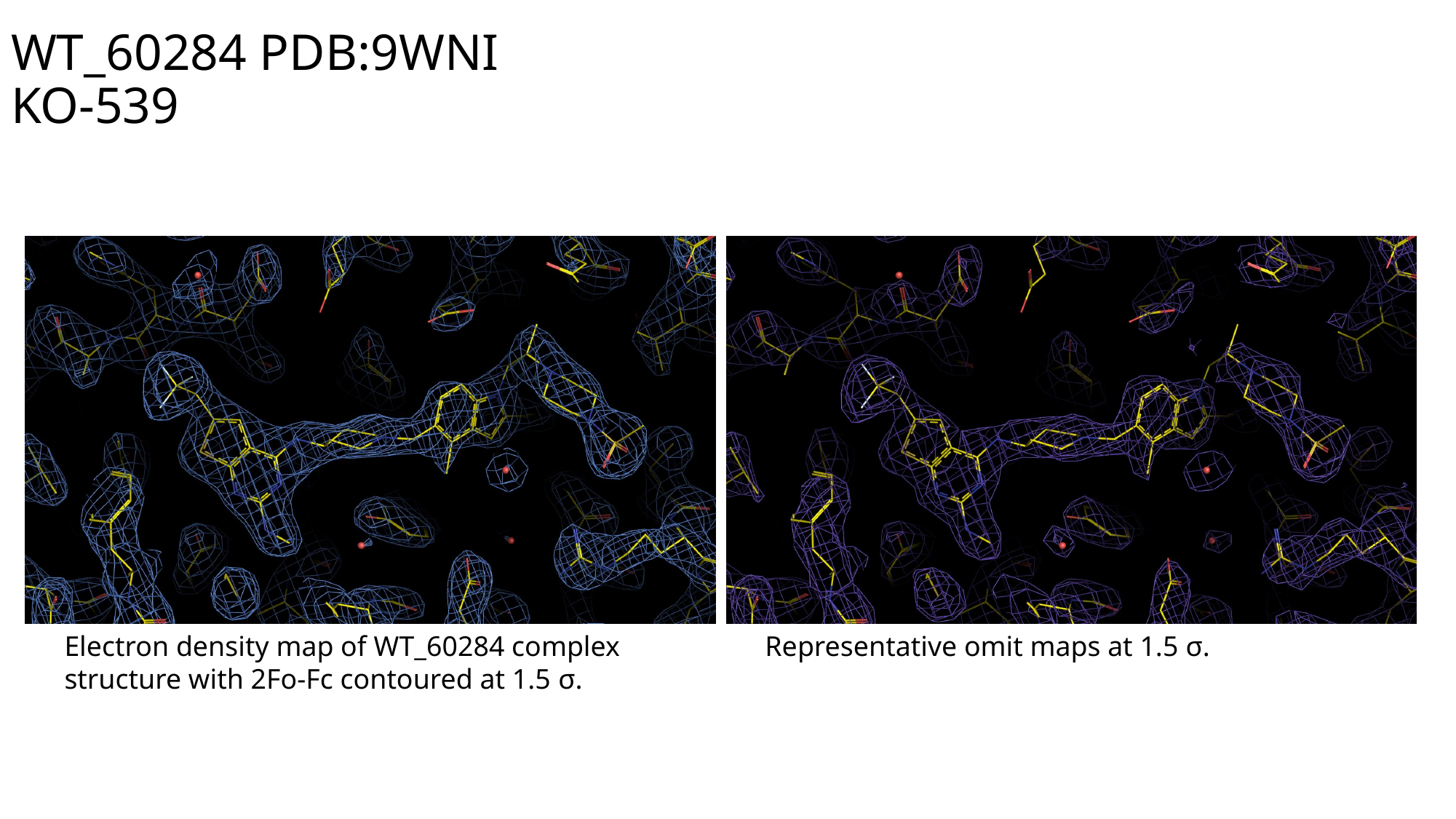

WT_60284 PDB:9WNI
KO-539
 Representative omit maps at 1.5 σ.
Electron density map of WT_60284 complex structure with 2Fo-Fc contoured at 1.5 σ.

## Slide 5
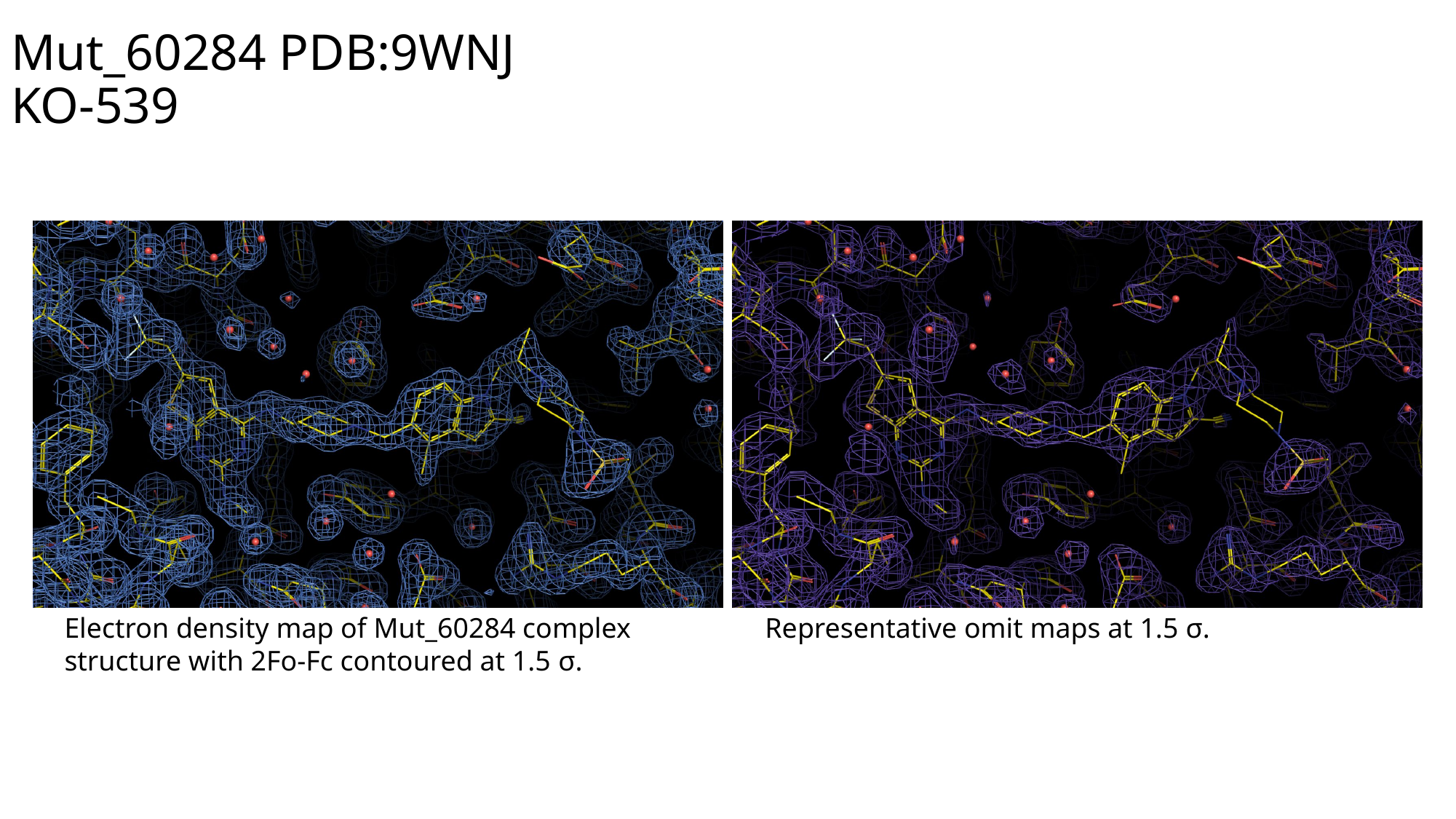

Mut_60284 PDB:9WNJ
KO-539
 Representative omit maps at 1.5 σ.
Electron density map of Mut_60284 complex structure with 2Fo-Fc contoured at 1.5 σ.

## Slide 6
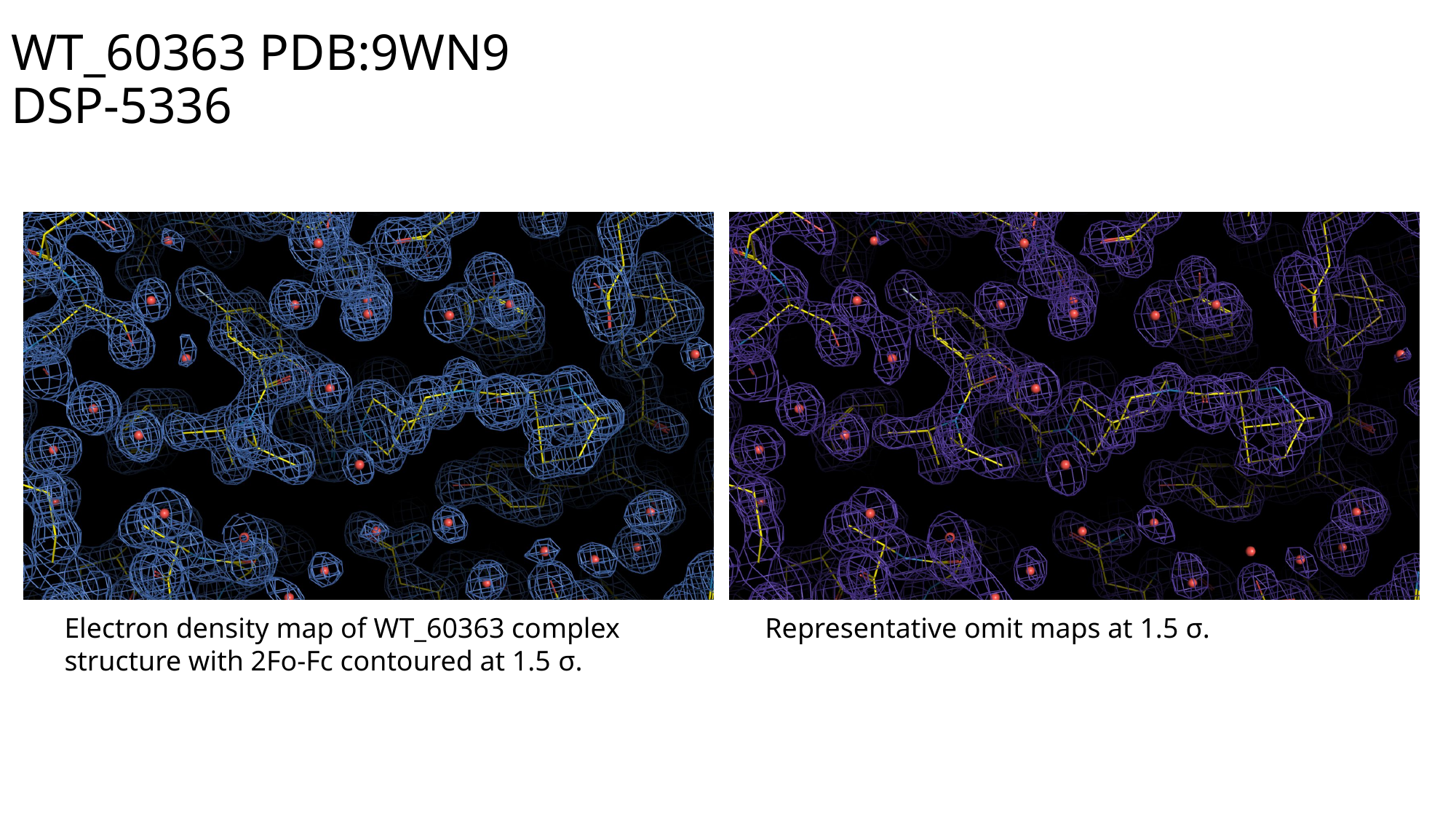

WT_60363 PDB:9WN9
DSP-5336
 Representative omit maps at 1.5 σ.
Electron density map of WT_60363 complex structure with 2Fo-Fc contoured at 1.5 σ.

## Slide 7
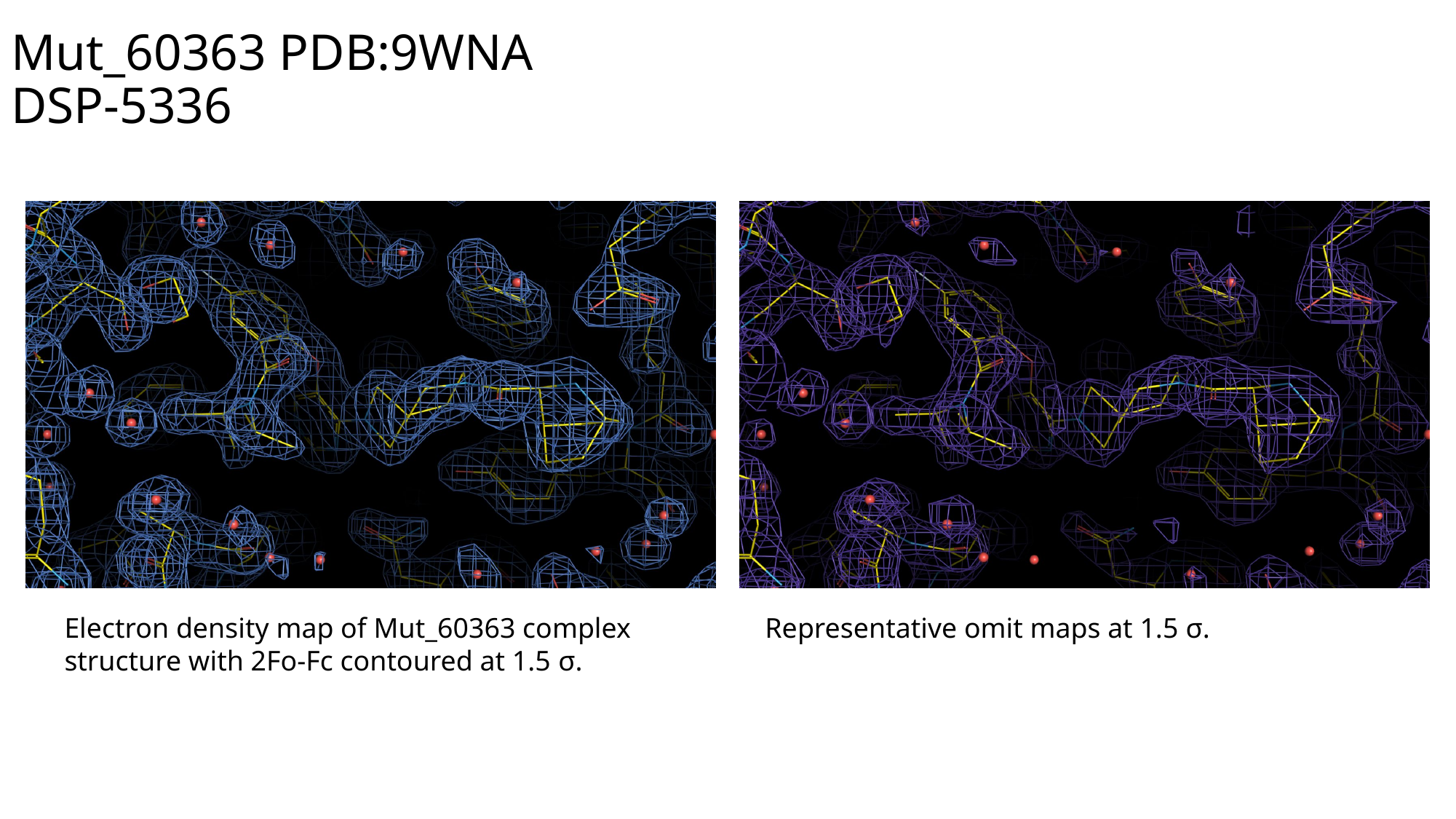

Mut_60363 PDB:9WNA
DSP-5336
 Representative omit maps at 1.5 σ.
Electron density map of Mut_60363 complex structure with 2Fo-Fc contoured at 1.5 σ.

## Slide 8
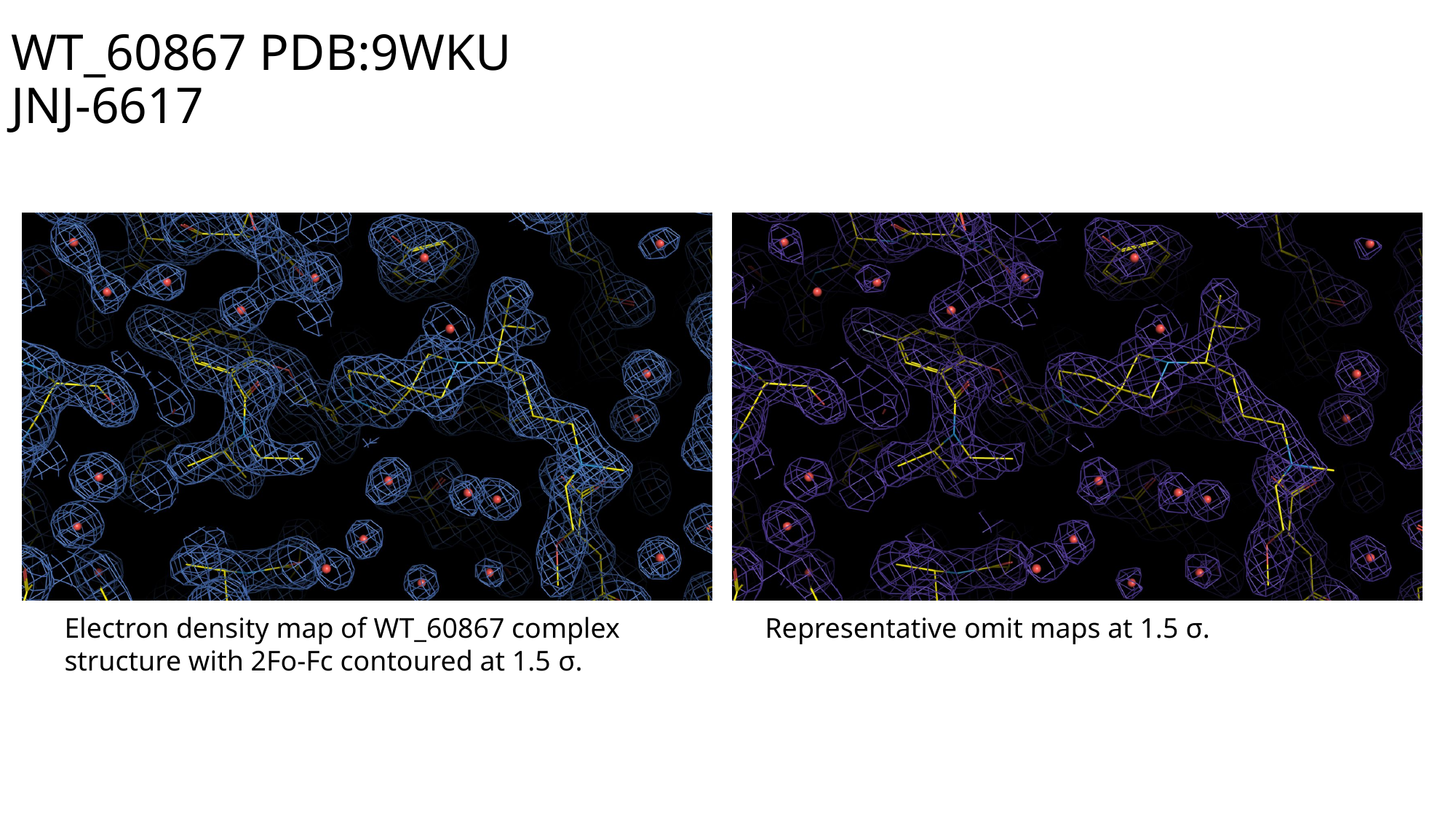

WT_60867 PDB:9WKU
JNJ-6617
 Representative omit maps at 1.5 σ.
Electron density map of WT_60867 complex structure with 2Fo-Fc contoured at 1.5 σ.

## Slide 9
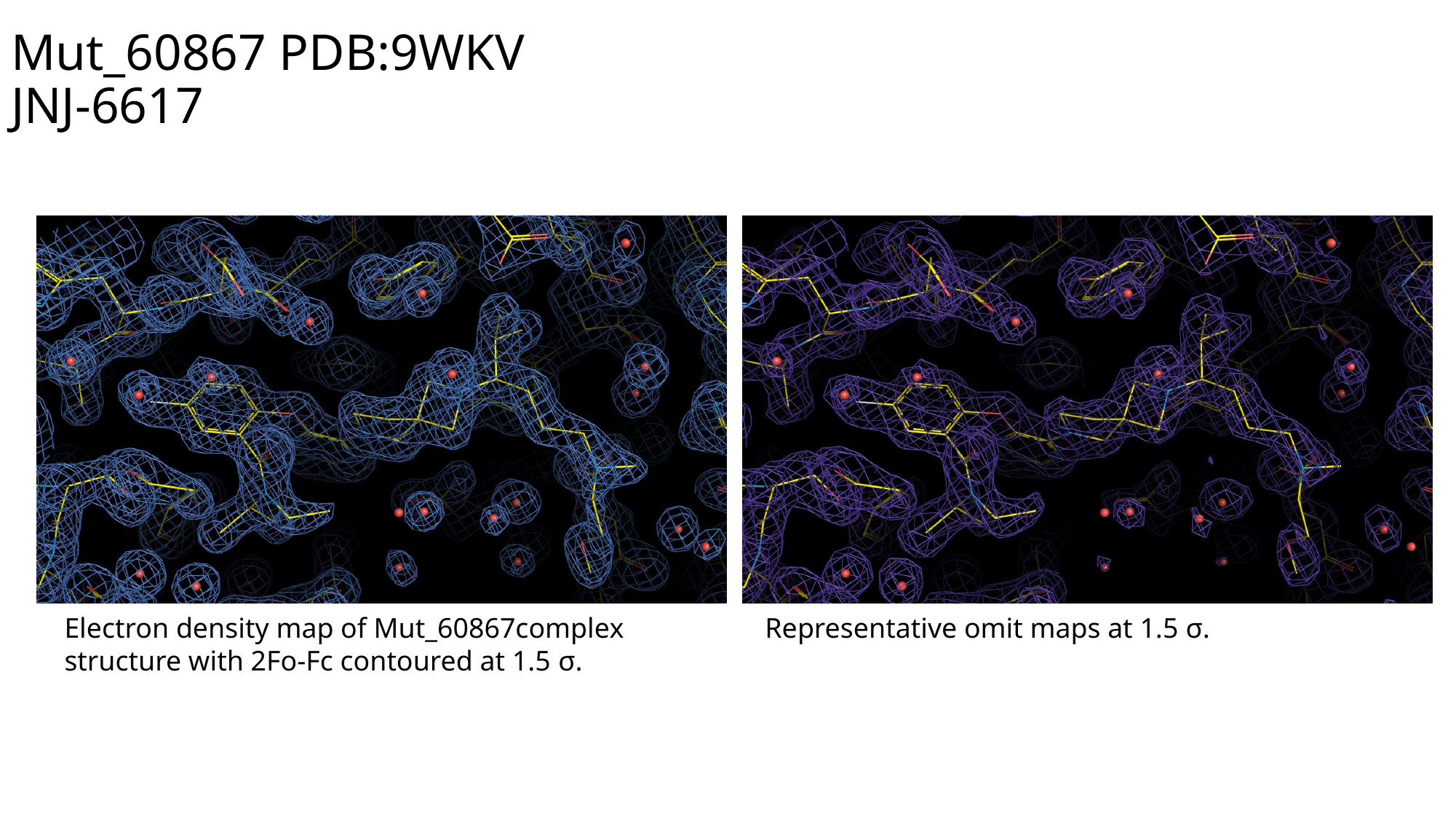

Mut_60867 PDB:9WKV
JNJ-6617
 Representative omit maps at 1.5 σ.
Electron density map of Mut_60867complex structure with 2Fo-Fc contoured at 1.5 σ.
