# Supplementary material for: CRISPR base editor screening identifies spectrum of MEN1 mutations impacting menin inhibitors in clinical trials
Source: Nat Commun. 2026 May 9;17:6265. doi: 10.1038/s41467-026-72685-1 (PMC13377036; doi:10.1038/s41467-026-72685-1)
Supplement: Supplementary file 10 — Reporting Summary [file 41467_2026_72685_MOESM10_ESM.pdf]

nature portfolio

Corresponding author(s): Jevon A. Cutler and Scott A. Armstrong

Last updated by author(s): 2026/03/04

Reporting Summary

Nature Portfolio wishes to improve the reproducibility of the work that we publish. This form provides structure for consistency and transparency in reporting. For further information on Nature Portfolio policies, see our Editorial Policies and the Editorial Policy Checklist.

Statistics

For all statistical analyses, confirm that the following items are present in the figure legend, table legend, main text, or Methods section.

n/a	Confirmed	
☐	☒	The exact sample size (*n*) for each experimental group/condition, given as a discrete number and unit of measurement
☐	☒	A statement on whether measurements were taken from distinct samples or whether the same sample was measured repeatedly
☐	☒	The statistical test(s) used AND whether they are one- or two-sided<br>*Only common tests should be described solely by name; describe more complex techniques in the Methods section.*
☒	☐	A description of all covariates tested
☐	☒	A description of any assumptions or corrections, such as tests of normality and adjustment for multiple comparisons
☐	☒	A full description of the statistical parameters including central tendency (e.g. means) or other basic estimates (e.g. regression coefficient) AND variation (e.g. standard deviation) or associated estimates of uncertainty (e.g. confidence intervals)
☐	☒	For null hypothesis testing, the test statistic (e.g. *F*, *t*, *r*) with confidence intervals, effect sizes, degrees of freedom and *P* value noted<br>*Give P values as exact values whenever suitable.*
☒	☐	For Bayesian analysis, information on the choice of priors and Markov chain Monte Carlo settings
☒	☐	For hierarchical and complex designs, identification of the appropriate level for tests and full reporting of outcomes
☐	☒	Estimates of effect sizes (e.g. Cohen's *d*, Pearson's *r*), indicating how they were calculated

Our web collection on statistics for biologists contains articles on many of the points above.

Software and code

Policy information about availability of computer code

Data collection	Flow cytometry data were acquired using BD FACSDiva Software (v9.2), and plate-based luminescence data were collected and analyzed using MARS Data Analysis Software (v3.41, build 25; BMG LABTECH).
Data analysis	Graphs and statistical analysis: GraphPad Prism (version 10.3.0)<br>Flow cytometry analysis: FlowJo (Version 10.10)<br><br>RNA-sequencing data:<br>Raw Illumina sequencer output was converted to FASTQ format using bcl2fastq (v2.20.0.422).<br>Reads (paired-end 37-mers) were aligned to the human (Gencode GRCh38/hg38) reference genome using STAR (v2.7.5a, sorted and duplicates marked/removed with picard pipeline tools (v2.9.4)).<br>Final “deduped” .BAM files were indexed using SAMtools (v1.95).<br>RNA-seq data visualizations were produced using IGVtools (TDF signal pileups; v2.3.75).<br><br>CRISPR screening and sequencing utilized an internal software tool from the Broad Institute, PoolQ, to deconvolute and quantitate perturbation barcodes.<br><br>Crystal Structure Analysis: XDS (v. 18Dec2014), CCP4 (v. 7.1.018) and CCP4 suite, REFMAC5 (v. 5.8.0267). Crystal structure visualizations and overlays were prepared using ICM Chemist Pro software from MolSoft, Inc. (https://www.molsoft.com) and PyMOL 31 by Schrödinger https://www.pymol.org/.

For manuscripts utilizing custom algorithms or software that are central to the research but not yet described in published literature, software must be made available to editors and reviewers. We strongly encourage code deposition in a community repository (e.g. GitHub). See the Nature Portfolio guidelines for submitting code & software for further information.

Data

Policy information about availability of data

All manuscripts must include a data availability statement. This statement should provide the following information, where applicable:

- Accession codes, unique identifiers, or web links for publicly available datasets
- A description of any restrictions on data availability
- For clinical datasets or third party data, please ensure that the statement adheres to our policy

Data availability
The co-crystal structures of SNDX-5613 bound menin proteins have previously been uploaded to PDB with the following accession numbers Protein Data Bank (PDB) ID# 7UJ4 (wild-type) and PDB ID#8E90 (I327) and are analyzed in this study. Co-crystal structures for other inhibitors have also been uploaded to PDB with the following accession numbers, DS-1594 bound menin ID# 9WKW (wild-type) and ID# 9WKX (I327), DSP-5336 bound menin ID# 9WN9 (wild-type) and ID# 9WNA (I327), JNJ-6617 bound menin ID# 9WKU (wild-type) and ID# 9WKV (I327), and KO-539 bound menin ID# 9WNI (wild-type) and ID# 9WNJ (I327).

RNA-sequencing data is available under the following GEO accession code: GSE294623.

The CRISPR base editing screen data is available under the following GEO accession code: GSE294624.

Source data are provided with this paper.

Research involving human participants, their data, or biological material

Policy information about studies with human participants or human data. See also policy information about sex, gender (identity/presentation), and sexual orientation and race, ethnicity and racism.

Reporting on sex and gender	N/A, no human participants in this study.
Reporting on race, ethnicity, or other socially relevant groupings	N/A, no human participants in this study.
Population characteristics	N/A, no human participants in this study.
Recruitment	N/A, no human participants in this study.
Ethics oversight	N/A, no human participants in this study.

Note that full information on the approval of the study protocol must also be provided in the manuscript.

Field-specific reporting

Please select the one below that is the best fit for your research. If you are not sure, read the appropriate sections before making your selection.

☒ Life sciences ☐ Behavioural & social sciences ☐ Ecological, evolutionary & environmental sciences

For a reference copy of the document with all sections, see nature.com/documents/nr-reporting-summary-flat.pdf

Life sciences study design

All studies must disclose on these points even when the disclosure is negative.

Sample size	The patient derived xenograft experiment in Figure 6A was repeated twice, with n ≥ 5 for each treatment group in each experiment. Statistical testing was not performed to determine sample size, but rather cohort size was selected based on the propensity to develop MEN1 mutations (i.e., the largest number of mice was n = 20 receiving 0.1% SNDX-5613 chow). Smaller sample sizes were used for Figure 6B as the aim was too assess for the type of mutations that arose on menin inhibitor therapy, as opposed to making conclusions about treatment efficacy or survival comparisons. For the experiment in Figure 6D, each treatment group had n = 5 mice. Statistical testing to determine sample size was not performed, but rather cohort size was selected to have sufficient number of mice alive after 28 days to perform RNA-sequencing from n = 2 mice per cohort. 2 mice per cohort for RNA-sequencing were chosen for cost, feasibility, and the scope of looking for MEN1 variants in this region with adequate sequencing coverage. For the PDX experiment in Figure 6E, vehicle control had n = 3 mice and the other groups had n = 4 mice per group. Statistical testing for group size was not performed. Cohort sizes were chosen for feasibility and conclusions were descriptive in nature.<br><br>Sample sizes for in vitro experiments were performed in at least technical triplicates.
Data exclusions	Source data has been made available for all experiments. In some 8-point dose-response curves for viability assays (Figure 3B and Supplemental Figure 2E), data points that were identified as technical outliers were omitted, with at least 5 individual data points across two biological replicates still present for all data with 8-point dose response curves. Likewise, for TR-FRET data, technical outliers were excluded. Data points that have been excluded can be clearly visualized in the source data, where omitted data is left empty.

Replication	Biological replication in independent experiments was performed when feasible. Screening viability assays (Figure 3A and Extended Data 2D), CRISPR screening (Figure 2B and 2C, Extended Data 1B and 1C), competition experiments (Extended Data 2B+C), and some PDX experiments (Figure 6B+C) were not performed with biological replication due to the size and scale of experimental approach. In the instances where only technical replication has been performed, we have noted this specifically in the figure legends and methods. The degree of replication is clearly indicated for each figure and supplementary figure.
Randomization	For in vivo experiments, mice receiving menin inhibitor therapy were randomized to treatment based on their leukemic burden in the peripheral blood prior to starting treatment.
Blinding	Blinding was not performed primarily due to feasibility. The majority of experiments involved in vitro assays and quantitative readouts where blinding is less of an issue. In vivo experiments were conducted using predefined objective endpoints, and animal health and humane endpoints were independently monitored by veterinary and technical staff in addition to the non-blinded study team.

Reporting for specific materials, systems and methods

We require information from authors about some types of materials, experimental systems and methods used in many studies. Here, indicate whether each material, system or method listed is relevant to your study. If you are not sure if a list item applies to your research, read the appropriate section before selecting a response.

Materials & experimental systems

n/a	Involved in the study
☐	☒ Antibodies
☐	☒ Eukaryotic cell lines
☒	☐ Palaeontology and archaeology
☐	☒ Animals and other organisms
☒	☐ Clinical data
☒	☐ Dual use research of concern
☒	☐ Plants

Methods

n/a	Involved in the study
☒	☐ ChIP-seq
☐	☒ Flow cytometry
☒	☐ MRI-based neuroimaging

Antibodies

Antibodies used	For flow cytometry monitoring of leukemia burden, PE-conjugated anti-human CD45 (BioLegend, 304058 or 304039, 1 µl antibody per 100 µl solution) and APC-Cy7-conjugated anti-mouse CD45 (BioLegend, 304014 or 103116, 1 µl antibody per 100 µl solution) were used.
Validation	Each lot of antibody is quality control tested by BioLegend using immunofluorescent staining with flow cytometric analysis. https://www.biolegend.com/ja-jp/products/pe-anti-human-cd45-antibody-708, https://www.biolegend.com/fr-lu/products/apc-cyanine7-anti-human-cd45-antibody-1914.

Eukaryotic cell lines

Policy information about cell lines and Sex and Gender in Research

Cell line source(s)	Cell lines were purchased from DSMZ (Deutsche Sammlung von Mikroorganismen und Zellkulturen) or American Type Culture Collection (ATCC): MOLM13 (DSMZ, ACC-554), MV4;11 (ATCC, CRL-9591), OCI-AML3 (DSMZ, ACC-582), 293T (ATCC CRL-3216).
Authentication	Cell lines were originally purchased from suppliers (DSMZ and ATCC) and responded to menin inhibitor treatments as previously reported by the Armstrong Laboratory: Ucklemann HJ et al., Science, 2020, PMID: 32001657; Krivstov AV et al., Cancer Cell, 2019, PMID: 31821784; Perner F et al., Nature, 2023, PMID: 36922589.
Mycoplasma contamination	Routine mycoplasma testing (Venor™ GeM Mycoplasma Detection Kit, PCR-based; Sigma-Aldrich: Product No. MP0025) was performed and cells were negative for mycoplasma.
Commonly misidentified lines (See ICLAC register)	No commonly misidentified cell lines were used in this study.

Animals and other research organisms

Policy information about studies involving animals; ARRIVE guidelines recommended for reporting animal research, and Sex and Gender in Research

Laboratory animals	For patient derived xenograft (PDX) experiments, female NOD.Cg-PrkdcscidIl2rgtm1Sug/JicTac mice were purchased from Taconic or NOD.Cg-Prkdcscid Il2rgtm1Wjl/SzJ from The Jackson Laboratory. PDX samples were obtained from the Center for Pediatric Cancer Therapeutics at DFCI or Public Repository of Xenografts (ProXe).

Wild animals	No wild animals were used during the study.
Reporting on sex	Mice used in the study were female and mouse sex was not considered in the study design.
Field-collected samples	No field-collected animals were used during the study.
Ethics oversight	Animal experiments were approved by Dana-Farber Cancer Institute's Institutional Animal Care and Use Committee (protocol number 16-021).

Note that full information on the approval of the study protocol must also be provided in the manuscript.

Plants

Seed stocks	No plants were used in this study.
Novel plant genotypes	No plants were used in this study.
Authentication	No plants were used in this study.

Flow Cytometry

Plots

Confirm that:

☒ The axis labels state the marker and fluorochrome used (e.g. CD4-FITC).

☒ The axis scales are clearly visible. Include numbers along axes only for bottom left plot of group (a 'group' is an analysis of identical markers).

☒ All plots are contour plots with outliers or pseudocolor plots.

☒ A numerical value for number of cells or percentage (with statistics) is provided.

Methodology

Sample preparation	For in vivo studies:<br>The leukemia burden in the peripheral blood was monitored by submandibular bleeding, calculating the percentage of mouse CD45+ and human CD45+ cells that were human CD45+. Red blood cell lysis was performed using RBC Lysis Buffer 10X (BioLegend Cell Staining Buffer Cat. No. 420201) before staining with PE-conjugated anti-human CD45 (BioLegend, 304058 or 304039, 1 µl antibody per 100 µl solution) and APC-Cy7-conjugated anti-mouse CD45 (BioLegend, 304014 or 103116, 1 µl antibody per 100 µl solution). Antibodies were diluted in PBS with 2% fetal bovine serum. At time of death, leukemic burden was confirmed and quantified in the bone marrow, spleen, and peripheral blood. Burden of disease analysis in the spleen and bone marrow was performed by homogenizing the spleen and crushing the bones (femurs, tibia, fibula, pelvic bones, and vertebral bodies), passing samples through a 40 µm filter, and then staining with human and mouse CD45 antibodies as detailed above. Following 30 minutes of antibody staining, samples were washed with PBS with 2% fetal bovine serum and resuspended in PBS with 2% fetal bovine serum and a 1:5000 diltution of DAPI (4',6-diamidino-2-phenylindole)) for dead cell exclusion.<br><br>For in vitro studies:<br>Cells were diluted with PBS containing a 1:500 ratio of DAPI (4',6-diamidino-2-phenylindole) staining of negative cells.
Instrument	BD FACS LSR-Fortessa
Software	FlowJo_v10.10.0
Cell population abundance	N/A, cells were not sorted.

Gating strategy

For in vitro studies, cells were first gated with FSC/SSC as above. Dead cells were excluded through DAPI (4',6-diamidino-2-phenylindole) staining of negative cells, gating on the BV421 negative cells. For experiments where PE+ cells were quantified, subsequent gating on PE+ cells was performed. An example of this gating strategy is now included in Supplementary Fig. 1H..

For in vivo studies, cells were gated using FSC/SSC. Doublets were then excluded using FSC-A and FSC-H. Dead cells were excluded through DAPI (4',6-diamidino-2-phenylindole) staining of negative cells, gating on the BV421 negative cells. Human PE CD45 cells and Mouse APC-Cy7 CD45 cells were then gated and the percentage of all total CD45 cells that were human CD45 positive was calculated. An example of this gating strategy is now included in Supplementary Fig. 5F.

☒ Tick this box to confirm that a figure exemplifying the gating strategy is provided in the Supplementary Information.
