# Supplementary material for: CRISPR base editor screening identifies spectrum of MEN1 mutations impacting menin inhibitors in clinical trials
Source: Nat Commun. 2026 May 9;17:6265. doi: 10.1038/s41467-026-72685-1 (PMC13377036; doi:10.1038/s41467-026-72685-1)
Supplement: Supplementary file 11 — Transparent Peer Review file [file 41467_2026_72685_MOESM11_ESM.pdf]

nature portfolio

Peer Review File

CRISPR base editor screening identifies spectrum of MEN1 mutations impacting menin inhibitors in clinical trials

Corresponding Author: Dr Scott Armstrong

This file contains all reviewer reports in order by version, followed by all author rebuttals in order by version.

Parts of this Peer Review File have been redacted as indicated to remove third-party material.

Version 0:

Reviewer comments:

Reviewer #1

(Remarks to the Author)
The manuscript by Bourgeois et al describes CRISPR-Cas9-based screening to predict the mutations in the MEN1 gene that would affect activity of five clinical menin inhibitors. Similarities and differences in the mutation pattern of MEN1 have been identified for each of these drugs in leukemia cells. The co-crystal structures of the menin WT/menin M327I mutant with these drugs were solved and used to rationalize the resistant mechanism induced by the mutations. In vivo studies with Revumenib were performed in PDX models to support in vitro finding. Overall, this is an interesting study with extensive in vitro data generated in leukemia cell lines to predict the resistance mutations to the clinical menin inhibitors. Thus, these findings could possibly serve to predict the mutations that might occur in leukemia patients. The novelty of this study is, however, somewhat limited due to the previous work published by the same group in Nature (Perner et al., 2023) focused on identification of MEN1 mutations in leukemia patients upon treatment with Revumenib menin inhibitor, many of which overlap with the mutations identified in the current work. Furthermore, in vivo validation of MEN1 mutations in mouse models is very limited (data are presented only for Revumenib, which was already described in the Nature 2023 paper from this group), while no in vivo studies validating the MEN1 resistant mutations identified by CRISPR-Cas9 for other drugs are included in this manuscript. Besides, crystallography data are missing critical validation. Overall, this study is interesting and could be valuable, but additional work to support these findings for other menin inhibitors (besides Revumenib) would be needed to validate the predictions made based on the results in leukemia cell lines.
Specific comments:
- The in vivo studies in the PDX models to validate mutations from the CRISPR-Cas9 screen were performed only with Revumenib. It would be important to perform similar studies either in PDXs or at least in xenograft models for other menin inhibitors reported here to validate whether the results from in vitro (in cells) studies can represent a predictor of in vivo conditions and could be used as a predictor of mutations in patients. Oral administration should be used (rather than chow as used for Revumenib) to reflect better the PK parameters and conditions relevant for leukemia patients.
- In vivo studies with Revumenib were performed by administering Revumenib in chow (in the food) at different drug levels (0.033%, 0.1% and 0.3%), with majority of mice in the 0.3% dose cohort not developing resistance. This way of drug administration, however, raises a question about: (1) the amount of drug being delivered to mice (e.g. is this controlled/equal within the group); (2) PK parameters with this way of drug administration due to constant access/consumption of the drug. This is important in the context of translating the preclinical data to predict the mutations that might occur in patients who are taking the drug orally once- or twice-daily. In vivo studies should be performed using oral drug administration (either in PDXs or at least xenograft models) to resemble the conditions to the clinical set up.
- The authors report the co-crystal structures of menin WT or M327I mutant with each inhibitor (Figures 1B, 2D, 4C-G (for menin WT) and Extended Figure 3B (for M327I mutant)) and utilize these data to provide the mechanistic insight into the impact of mutations on inhibitor binding. However, the coordinates and electron density maps for these complexes (both for menin WT and menin mutants in complex with inhibitors reported here) have not been deposited in PDB. This needs to be done and PDB codes need to be provided in the manuscript before acceptance.
- In line with the above, Tables reporting quality of the collected X-ray data for all menin WT and menin mutant-inhibitor complexes and the refinement statistics need to be provided in the manuscript for revision. This is a standard approach required to validate the quality of the crystal structures reported. This is particularly important here since the mechanism of sensitivity or resistance to the specific mutant is being discussed based on the crystal structures. At this point it is not even clear what is the resolution of these X-ray data and other critical crystallography parameters are missing.
- Presentation of the crystal structures and their discussion in the result section should be improved/more detailed to be more informative. For example, comparison of the menin WT and M327I mutant with specific drugs is shown in the Extended

Figure 3B and the displacement of inhibitors upon mutation is discussed in the text. However, “the displacement” or “substantial movement” referred in the Result section are not clearly demonstrated in the Figures and there are no details in the text what is the extend of the displacement and which interactions are affected. More details need to be provided to explain the molecular basis of compromised inhibitory activity upon menin mutations.
- The synthesis of drugs is not provided in the method section, but instead the authors refer to some patents. However, some of the references cite PubChem source (e.g. for Enzomenib, reference 17) rather than specific patent or publication. The authors should either report the entire synthesis of each drug or clearly indicate which publication or patent was used to prepare a specific drug. In any case, the analytical data for final products (e.g. each specific menin inhibitor) from the synthesis should be provided (NMR, HRMS) to confirm the identity and purity of these compounds. - The RNA-seq data should be deposited to GEO before re-submission and the entries should be provided to the reviewers. - The MEN 1-focused CRISP-Cas9 screen revealed various mutations in MEN 1, either unique or common for each drug. However, the in vitro studies to validate the impact of mutations on inhibitor binding in the TR-FRET assay are only presented for M327I and T349M mutants. Other mutants (e.g. Gly331, Asp 348, Glu336, etc.), which affect inhibitory activity for majority or some of these drugs, should also be characterized using in vitro biochemical assay.

Reviewer #2

(Remarks to the Author)
Manuscript describes systematic studies evaluating activity and resistance mechanisms to 5 clinical stage menin inhibitors. By employing CRISPR-Cas9 base editing approach they discovered multiple mutations in MEN1 arising to all tested menin inhibitors. To explain effect of mutations, they determined multiple crystal structure of menin with small molecule inhibitors. They also evaluated activity of menin inhibitors against various cell lines with MEN1 mutations and some animal models. Overall, these studies are very systematic, comprehensive and high quality. Authors demonstrate that similar mutations will likely occur to all menin inhibitors which are currently in clinical studies or to FDA approved revumenib. Extensive mutational data might inform on potential direction for development of the next generation of menin inhibitors to minimize impact of MEN1 mutations. Another important conclusion is that better potency of menin inhibitor might limit or even prevent the occurrence of MEN1 mutations. However, the key limitation of this manuscript is limited novelty. Activity of many tested menin inhibitors have been already reported in original publications describing discovery of these drugs. Same authors previously published CRISPR editing mutagenesis showing generation of mutations in MEN1 resulting in resistance to revumenib (Perner et al 2023).

This manuscript could be strengthened by addressing following points:
- one of the most consequential conclusions is that more potent menin inhibitor can decrease the rate or even limit occurrence of MEN1 resistant mutations. This conclusion is based on cell-based studies and I wonder whether authors could confirm this in vivo, by treating PDX mice with two different inhibitors. This could significantly increase the novelty. - Authors show the results of the in vivo experiment in PDX model CBAM-44728, where continuous treatment with SNDX-5613 administered in chow, results in occurrence of MEN1 mutations in dose dependent manner. However, patient treatment is different, and the drugs are administered q.d. or b.i.d., which when compared to chow based administration, leads to different PK profiles, such as absorption, Cmax, half-life, clearance etc. In consequence, drug exposure and menin inhibition profile might significantly differ from q.d. or b.i.d. administration, and thus impact development of MEN1 mutations. It would be valuable for the current study to evaluate development of MEN1 mutations using drug administration that is similar to patient treatment.
- authors describe comparison of 5 menin inhibitors and conclude that potency of these inhibitors plays a role in development of MEN1 resistant mutations; it would be important to include additional discussion how drug-like properties such as PK, dose limiting toxicity might impact development of drug resistant MEN1 mutations.
- another important question that has not been addressed in this study is whether these MEN1 mutations are de-novo resulting from drug treatment, or are already present as minor clones, which are selected upon drug treatment
- Presentation of figures showing binding modes of menin inhibitors makes is difficult to see key interactions with menin. In addition, crystal structure of menin complexes should be deposited in PDB.

Reviewer #3

(Remarks to the Author)
Review of Bourgeois et al NatComms

Summary
Bourgeois et al use CBE and ABE base editing screens of MEN1 to investigate potential mechanisms of resistance to 5 MEN1 inhibitors for the treatment of leukemia. The authors validate selected resistance hits with cell proliferation, biophysics and infer mechanism on the disruption of inhibitor binding with the use of protein crystal structures. Importantly, the authors highlight key residues in MEN1 where resistance differs between drugs – some of which are found in PDX models. A weakness of the paper analysis of the base editing screen and the lack of mechanistic insight. The authors could strengthen the paper by considering the following points.

Main points
- The authors use log fold change throughout, which is fine, but due to the noise in the data, they should add statistics.

DrugZ or MAGeCK would be appropriate to include FDR and p-values.
• Extended data 1C shows that the average log2fold change in the presence of drug is around -2? That suggests most mutations are strongly deleterious. Without proper comparison to controls in the library such as intergenic/non-targeting guides or positive controls, it is hard to know whether this signal is biological or technical.
• Figure 3 – 37 out of 80 cell lines validate the screens, what is this in numbers of gRNAs? For drug resistance (positive selection), this seems a low rate of validation – can the authors comments why this might be? Recall of known lethal variants or known resistance variants is often done to understand sensitivity.
• The drug-sensitising variants such as MEN1 E368K seem interesting and similar drug-sensitising variants were described recently in another base editing paper (PMID: 39424923 – EGFR, PARP). How are these working in MEN1? Some mechanistic understanding could be useful to improve future MEN1 inhibitors.
• Although some selected validation is performed in Fig 3, it will be important to summarise for the reader how many and which variants from the screen have been validated (in vitro, in vivo etc), and which have been NGS verified. This is important for the usefulness of the data clinically.
• The authors state that the degree to which... "these MEN1 mutations attenuates menin inhibitor activity cannot be inferred from the genetic screening data". This is contentious if the MAVE assay is a true readout of menin activity. Can the authors test this in 4B by plotting LFC vs Ki shift?
• With the base editing dataset, can the authors now speculate on a combination of menin inhibitors that would be effective and prevent or slow drug resistance? Do the authors think that it is feasible to combine them in vivo or in patients? Would this be more effective than high dose inhibitor? Some testing of menin combinations (and perhaps benchmarking to high dose of one) would be a valuable addition.
• Could the authors comment on potential off-target resistance mechanisms in the Discussion i.e. not in MEN 1? This seems a potential limitation of the study.

Minor points
• Analysis code should be on GitHub otherwise we cannot review this.
• Why are there gaps in the sequence of amino acids in Fig. 2c?
• Fig.4c-g is not very clear for the reader. Perhaps distil this information into a couple of structures or ensemble, highlighting key residues? Currently simply referred to as Fig4c-g without any signposting.
• I recommend the authors deposit their data on MAVEdatabase to aid data sharing.
• The screens are referred to as CRISPR-Cas9 screens throughout. They are CRISPR base editing screens, not Cas9 screens (cut). This needs to be rephrased for clarity.
• Can the authors speculate on why some mutations might appear spontaneously and in vivo, and others not? Is it a function of effect size, or something else?
• Figure 6A is poorly presented. I suggest showing the data more carefully by segregating different doses of the drug, for example. What's happening in the WT cancer cells? Are there other mutations, not in MEN1 or just below detection?

Reviewer #4

(Remarks to the Author)
The study by Bourgeois et al and the Armstrong group is an in-depth analysis of acquired MEN1 mutations in the context of different MEN1 inhibitors under clinical evaluation. This is an extension from their previous study in Nature evaluating clinical samples from the AUGMENT trial and a prior base editing screening approach that utilized base editors capable of only C>T nucleotide transitions and SpCas9 75 nickases that required an NGG protospacer adjacent motif (PAM) site. This paper now uses MV4;11 cell line with C>T and A>G editors along with 5 reversible, non-covalent menin inhibitors that had entered clinical trials: DS-1594 (emilumenib, Daiichi Sankyo), JNJ-75276617 90 (bleximenib, Janssen Pharmaceuticals, referred to as JNJ-6617 hereafter), KO-539 (ziftomenib, Kura 91 Oncology), SNDX-5613 (revumenib, Syndax Pharmaceuticals), and DSP-5336 (enzomenib, Sumitomo 92 Pharma). In addition to the identification of the spectrum of acquired resistance mutation at high resolution using this updated CRISPR base editing strategy, they support their findings with co-crystal structures of each inhibitor interacting with menin and identified similarities and differences between the binding of each inhibitor. They extensively cross-validated the identified mutations with the variety of MEN1 inhibitors and then generated 80 mutant MV4;11 cell lines for testing. Spontaneous development of these mutations was tested in a different KMT2Ar cell line and with PDX samples. They then nicely the impact of different concentrations of SDNX using supplemented chow to induce mutations and determined that it was significantly less likely at high concentrations. Overall, this is a rigorous study of high translational impact. It would be helpful to the field to understand if the levels of SNDX achieved in their high-dose chow are achievable in patients. And does the degree of MEN1 inhibition at high dose result in undesirable clinical side effects or functional defects in normal HSPCs? Beyond this, I have no other concerns about this study, which will be highly impactful to the field.

Reviewer #5

(Remarks to the Author)
I co-reviewed this manuscript with one of the reviewers who provided the listed reports. This is part of the Nature Communications initiative to facilitate training in peer review and to provide appropriate recognition for Early Career Researchers who co-review manuscripts.

Version 1:

Reviewer comments:

Reviewer #1

(Remarks to the Author)
The revised manuscript by Bourgeois et al. addresses most of the points initially raised through additional experiments and further data analysis. Importantly, data has been uploaded to the appropriate databases (PDB, GEO), making them available to the scientific community. There are, however, several minor points that still need to be addressed: - The rationale beyond using KMT2B-derived peptide and a different assay (TR-FRET) for characterization of the activity of inhibitors with additional menin mutants included in the revised manuscript (R334, K368, A372) instead of KMT2A-derived peptide utilized in FP competition experiments with mutant initially included (I327, M349, R331) is not clear. Comparison of Ki values for all menin inhibitors with WT menin obtained from both assays indicates ~2-orders of magnitude (~100x) difference between values from FP (with KMT2A 4-43 probe, Supplementary Figure 3A top) versus TR-FRET (with KMT2B 15-48 probe, Supplementary Figure 3A bottom), which might introduce some inconsistency. To compensate for this discrepancy, it would be important to provide a Table with: (1) IC50 values for WT and every mutant with each compound (specifying which method was used for IC50 measurements), and even more importantly (2) provide the fold difference in IC50 values for each mutant compared with WT menin for every compound.
- The IC50 values are missing for menin WT in the Supplemental Figure 3B (first graph on the top) – only “compound names: nM” are shown, but the actual values are missing. These values (collected from FP assay) need to be added as they are important for comparison with the menin-WT replicates 1-3 measured using TR-FRET method.
- The crystal structures presented at the Main Figure 4C-F include some of the hydrogen atoms on the protein, ligands or water molecules. Based on the resolution of these structures (>1.5A), the atomic coordinates for these hydrogen atoms are unlikely to be derived from the crystal structures (experimental data) and should be removed from these figures with only heavy atoms shown.
- The title of panel in Main Fig.1C “Menin-KMT2A peptide binding” is misdealing as this figure shows activity (titration curves from FP and IC50) for inhibitors
- Right panel of Supplementary Figure 2 is labeled “MV4;11 MEN1 WT vs sgRNA K367K, E368K”, which seems incorrect. Is this a single mutant or double mutant cells line (K367K seems incorrect/ not needed)?
- In the revised methods section (KMT2A/B-menin displacement assay), the KD values used for calculation of Ki were measured by SPR – as stated in the manuscript: “KD values were determined for menin proteins using surface plasmon resonance (data not shown)” – sentence added to the revised manuscript. Since the authors use Ki for comparison of the fold difference between menin WT and mutants for each inhibitor, it could be worth including the SPR data in the supplement (especially since the data already exists). Alternatively, IC50 values could be used instead of Ki to calculate fold difference for mutants vs. WT for each compound.
- Can the authors define “L” in the Ki equation (methods: KMT2A/B-menin displacement assay section).

Reviewer #2

(Remarks to the Author)
Manuscript by Bourgeois et al describes comprehensive analysis of mutations arising in MEN1 upon exposure to various menin inhibitors. This is an important and timely research. Authors addressed all my comments and significantly improved the manuscript. I have couple minor points to address:

Comment 1.
Finding that more potent menin inhibitor reduces rate of MEN1 mutations and the outgrowth of resistant leukemia clones is one of the most consequential conclusions from this manuscript. Authors strengthened this finding in the revised manuscript by including new in vivo data showing that increasing the dose of different menin inhibitor such as JNJ-6617 effectively suppresses the development of MEN1 mutations.
Additional new results examine the effect of combination of two menin inhibitors (JNJ-6617 and DSP-5336). Although these experiments are in cell culture, they strengthen the overall conclusions regarding emergence of MEN1 mutations. A limitation of this approach is that the combination was tested at doubled total inhibitor concentration (25 nM DSP-5336 plus 25 nM JNJ-6617) compared with single-agent concentrations (25 nM), which likely contributes to the absence of resistant clones. This point should be acknowledged in the text.
Minor point: concentration of DSP-5336 is missing in Supplementary Fig 4 legend:
“In these two independent experiments (A+B), 12.5 million MOLM13 cells were seeded per replicate, with 5 flasks per experiment treated with JNJ-6617 25 nM, 5 flasks per experiment treated with DSP-5336 25 nM, and 6 flasks per experiment treated with both JNJ-6617 25 nM and DSP-5336 nM.”

Comment 4.
The authors’ response regarding the possibility that MEN1 mutations may be pre-existing is very relevant. It would be worth mentioning in the text that several MEN1 mutant clones were detected prior to day 28 of treatment, suggesting the possibility that these mutations were pre-existing.

Minor point: Figure 6 legend refers to Supplementary Fig 4C but there is no such figure, please correct that.

Final question to the authors – were any double mutations in MEN1 observed in these screens or in vivo?

Reviewer #3

(Remarks to the Author)
The authors have significantly improved the clarity and reporting in the revised manuscript. I particularly value the additio nal work on combining MEN1 inhibitors.
Relating to my points on the screen analysis:

- The depletion of intergenic control guides suggests there has been some bottlenecking and therefore coverage of the library in cell culture may not be sufficient. This is leading to noise in the L2FC vlaues and may be a source of false positives. This is an important point as the authors make claims on drug-sensitizing variants.
- Screens analyzed using MAGeCK RRA can generate FDR and pvalues for specific gRNAs when used in guide mode (not gene aggregated PMID: 25476604), which for gene editing screens, normally gives significant FDR and p-values for individual gRNAs (e.g. PMID: 35165384, PMID: 40845844, PMID: 38134886 – base, CRISPR and prime screens). In their response, the authors cite another base editing screen as an example to justify only z-scores (PMID: 39424923), but this study provides MAGeCK pvalues and FDRs. I would include these statistics to ensure the reader is aware of technical variability.

It will be important to see L2FC correlation plots of replicates to understand variation in their data leading to the lack of significant gRNA hits.
The authors have addressed my other comments.

Reviewer #4

(Remarks to the Author)
I have no further comments for the authors. They have rigorously addressed the issues that were raised.

Reviewer #5

(Remarks to the Author)
I co-reviewed this manuscript with one of the reviewers who provided the listed reports. This is part of the Nature Communications initiative to facilitate training in peer review and to provide appropriate recognition for Early Career Researchers who co-review manuscripts.

Version 2:

Reviewer comments:

Reviewer #1

(Remarks to the Author)
My concerns were fully addressed in the revised version and I do not have any further requests.

Reviewer #3

(Remarks to the Author)
The authors have improved data reporting and transparency by the inclusion of Supp Fig 1F and 1G, demonstrating high replicate correlation in the base editing screens.
I thank the authors for the inclusion of Supplementary Dataset 2, which is an important statistical summary of the screen results. However, in its current form, there is no key in one tab that allows the reader to check the p-value for each amino acid hit/sgRNA. Please revise this table to make it more useful and avoid readers manually searching for each sgRNA between spreadsheets.
Given this new statistical summary, the authors should add a comment in the text to summarise how they selected the validation sgRNA set. We chose 40 sgRNAs (line 40) with L2FC>4. Please comment on how many of these were significant.
The authors have addressed my remaining concerns, and I wouldsupport publication of the revised article in Nature Communications.

Open Access This Peer Review File is licensed under a Creative Commons Attribution 4.0 International License, which permits use, sharing, adaptation, distribution and reproduction in any medium or format, as long as you give appropriate credit to the original author(s) and the source, provide a link to the Creative Commons license, and indicate if changes were made.
In cases where reviewers are anonymous, credit should be given to 'Anonymous Referee' and the source.
The images or other third party material in this Peer Review File are included in the article's Creative Commons license, unless indicated otherwise in a credit line to the material. If material is not included in the article's Creative Commons license and your intended use is not permitted by statutory regulation or exceeds the permitted use, you will need to obtain permission directly from the copyright holder.
To view a copy of this license, visit https://creativecommons.org/licenses/by/4.0/

We thank all reviewers for their comments, which we believe has helped strengthen our manuscript. We have responded to reviewer comments below in red, and tracked changes in the manuscript with all changes made.

REVIEWER COMMENTS

Reviewer #1 (Remarks to the Author):

The manuscript by Bourgeois et al describes CRISPR-Cas9-based screening to predict the mutations in the MEN1 gene that would affect activity of five clinical menin inhibitors. Similarities and differences in the mutation pattern of MEN1 have been identified for each of these drugs in leukemia cells. The co-crystal structures of the menin WT/menin M327I mutant with these drugs were solved and used to rationalize the resistant mechanism induced by the mutations. In vivo studies with Revumenib were performed in PDX models to support in vitro finding. Overall, this is an interesting study with extensive in vitro data generated in leukemia cell lines to predict the resistance mutations to the clinical menin inhibitors. Thus, these findings could possibly serve to predict the mutations that might occur in leukemia patients. The novelty of this study is, however, somewhat limited due to the previous work published by the same group in Nature (Perner et al., 2023) focused on identification of MEN1 mutations in leukemia patients upon treatment with Revumenib menin inhibitor, many of which overlap with the mutations identified in the current work. Furthermore, in vivo validation of MEN1 mutations in mouse models is very limited (data are presented only for Revumenib, which was already described in the Nature 2023 paper from this group), while no in vivo studies validating the MEN1 resistant mutations identified by CRISPR-Cas9 for other drugs are included in this manuscript. Besides, crystallography data are missing critical validation. Overall, this study is interesting and could be valuable, but additional work to support these findings for other menin inhibitors (besides Revumenib) would be needed to validate the predictions made based on the results in leukemia cell lines.

We thank Reviewer #1 for their comments and thoughtful feedback. Regarding the novelty of our study relative to Perner et al. (2023), the prior CRISPR base-editor screen used only a preclinical analogue of SNDX-5613 (VTP-50469) and lacked single-guide validation. Moreover, the previous Cas9 nickase and base-editor required an NGG PAM and was restricted to C>T transitions, meaning critical amino acid residues like M327 were not captured. Our current dual-base-editing approach (A>G and C>T), combined with a less restrictive NG PAM requirement, enabled interrogation of approximately 4x more assayable menin residues. We applied this approach across five clinical menin inhibitors. This study addresses a key outstanding question at a pivotal time: whether other menin inhibitors are affected by the same or distinct *MEN1* mutations. Prior studies of other menin inhibitors have primarily evaluated activity against a limited subset of SNDX-5613-associated mutations. For example, Kwon et al., *Blood* 2024 (PMID: 38905635), showed that JNJ-6617 (bleximenib) displayed superior activity against M327I and T349M compared with SNDX-5613, while Wang et al., *Lancet Oncol.* 2024 (PMID: 39362248), demonstrated that KO-539 (ziftomenib) retained binding to T349M but not M327I. There have been no studies since the Perner et al, 2023 manuscript describing the *MEN1* mutations likely to arise with other menin inhibitors.

In this resubmission, we strengthen the connection between our predictive screen and spontaneous resistance observed under drug pressure, both *in vitro* and *in vivo*. Based in part on Reviewer #1's

feedback, we have provided further evidence of spontaneous mutation generation *in vitro* and *in vivo* without genetic editing. For 3 of the 5 inhibitors in this study, we have demonstrated that the top hit(s) from the CRISPR base editor screen spontaneously arose with drug treatment either *in vitro* or *in vivo*: for DSP-5336, E368K and E368V mutations arose spontaneously *in vitro*; for JNJ-6617, M327V, T349M, and G331D mutations arose spontaneously *in vitro* and G331D, T349M, and D290V mutations arose spontaneously *in vivo*; and for SNDX-5613 we demonstrated all clinically reported mutations arose spontaneously *in vivo*. This orthogonal validation supports the relevance the predictions from the screen to likely clinically relevant resistance mutations, underscoring both the novelty and translational significance of the present study.

Specific comments:

Reviewer #1, Comment 1. The in vivo studies in the PDX models to validate mutations from the CRISPR-Cas9 screen were performed only with Revumenib. It would be important to perform similar studies either in PDXs or at least in xenograft models for other menin inhibitors reported here to validate whether the results from in vitro (in cells) studies can represent a predictor of in vivo conditions and could be used as a predictor of mutations in patients. Oral administration should be used (rather than chow as used for Revumenib) to reflect better the PK parameters and conditions relevant for leukemia patients.

We thank the reviewer for this important comment about using additional menin inhibitors to evaluate *MEN1* mutation generation *in vivo*. To address this, we performed a new experiment that we believe strengthens the novelty and impact of this work. We used the same PDX model (CBAM-44728) in which *MEN1* mutations previously arose with SNDX-5613 (Main Figure 6A). We treated mice with two oral-gavage doses of JNJ-6617 (30 mg/kg and 100 mg/kg daily), both previously used in PDX models demonstrating the compound's preclinical efficacy (Kwon MC et al., *Blood*, 2024, PMID: 38905635). We selected JNJ-6617 because it is the only agent besides SNDX-5613 with published murine pharmacokinetic parameters.

Notably, *MEN1* mutations were detected in 4 of 4 mice treated at 30 mg/kg daily and 1 of 4 mice treated at 100 mg/kg daily. The peripheral blood leukemia burden annotated with *MEN1* mutations identified from bone marrow specimens by Sanger sequencing at death is displayed below. This is new Main Figure 6B in our revised submission.

B CBAM-44728 (*KMT2A::AF10*) AML PDX, peripheral blood leukemia

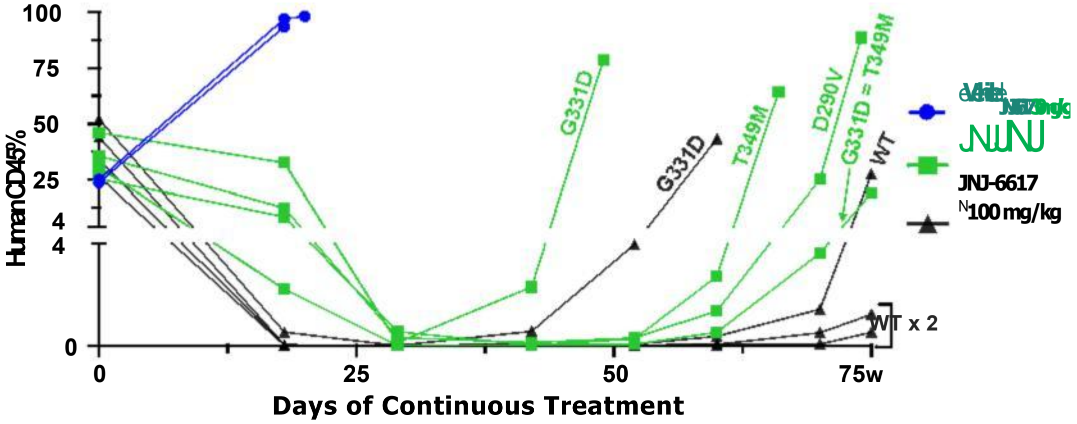

These *MEN1* mutations emerged during oral-gavage dosing, directly addressing the reviewer's concern that mutation development be demonstrated with oral administration. Two mice developed dominant G331D mutations, one mouse a dominant T349M mutation, one mouse developed a dominant D290V mutation, and one mouse had a similarly sized G331D and T349M mutation by Sanger sequencing. This represents the first preclinical demonstration of *MEN1* mutations arising in a PDX model treated with a menin inhibitor other than SNDX-5613, and it identifies a novel *MEN1* mutation not previously observed with SNDX-5613 (D290V).

The *MEN1* mutations identified in this experiment were illuminating with regards to our CRISPR base editor screen and validation. A guide targeting T349 scored in our screen and shifted the absolute IC_{50} ~10-fold in our confirmatory viability assays (Main Figure 3B), which makes it the second most deleterious mutation for JNJ-6617 predicted by our CRISPR base editor screen and validation.

In contrast, the only guide targeting D290 in the sgRNA library shifted the absolute IC_{50} "only" 4.0x and the most deleterious guide targeting G331 shifted the absolute IC_{50} "only" 3.1x for JNJ-6617 in viability assays (Main Figure 3A+B). While these shifts are modest, they were among the top 9 most deleterious residues in our screening viability assays for JNJ-6617 (in decreasing order of absolute IC_{50} shift): M327, T349, A247, S160, M233, D290, D136, P65, and G331. We hypothesized that specific amino acid substitutions at D290 and G331 might be required to attenuate JNJ-6617 activity, and we sought to test this in individual base editor cell lines harboring mutations at these residues. These results are presented in new Main Figures 3E-H and Supplementary Fig. 2H-M, and are described in the manuscript in the italics below.

*"We hypothesized that MEN1 residues with modest absolute IC_{50} shifts in viability assays might have greater impact with alternate amino acid substitutions. To test this, we exposed MV4;11 C>T cells (MEN1 wild-type, sgRNA D290-targeting, or sgRNA A330/G331-targeting) to DMSO, JNJ-6617 (25 nM), or KO-539 (25 nM) for 20 days (**Supplementary Fig. 2H+K**). Without drug, these guides primarily generated D290N (79.7% of alleles) and G331N (50.9% of alleles) mutations (**Figures 3E+G, Supplementary Fig. 2I+L**). Both MEN1-mutant lines continued to grow under MI pressure, and amplicon sequencing after 20 days showed that JNJ-6617 selected for D290H (5.5→64.2%) > D290Y (3.9→10.1%) > D290N (79.7→25.6%) and G331D (0.27→66.5%) > G331N (50.9→9.7%), versus KO-539 which selected for D290Y > D290H (3.9→35.1% and 5.5→21.3%) and G331N (50.9→89.1%). After drug washout, D290H cells showed a >14-fold absolute IC_{50} shift for JNJ-6617, with no other inhibitor shifting >5-fold (**Figure 3F**). G331D conferred pan-resistance, shifting DS-1594 >194x, SNDX-5613 >88x, KO-539 117x, JNJ-6617 16x, and DSP-5336 5.5x (**Figure 3H, Supplementary Fig. 2J**). In contrast, G331N largely retained sensitivity to JNJ-6617 (1.4x shift), showed increased sensitivity to DSP-5336 (0.5x), and strong resistance to DS-1594 (65x), KO-539 (37x), and SNDX-5613 (22x) (**Supplementary Fig. 2M**). These results underscore that the particular amino acid substitution at a given residue can critically influence the magnitude of MI resistance."*

Main Figures 3E-H:

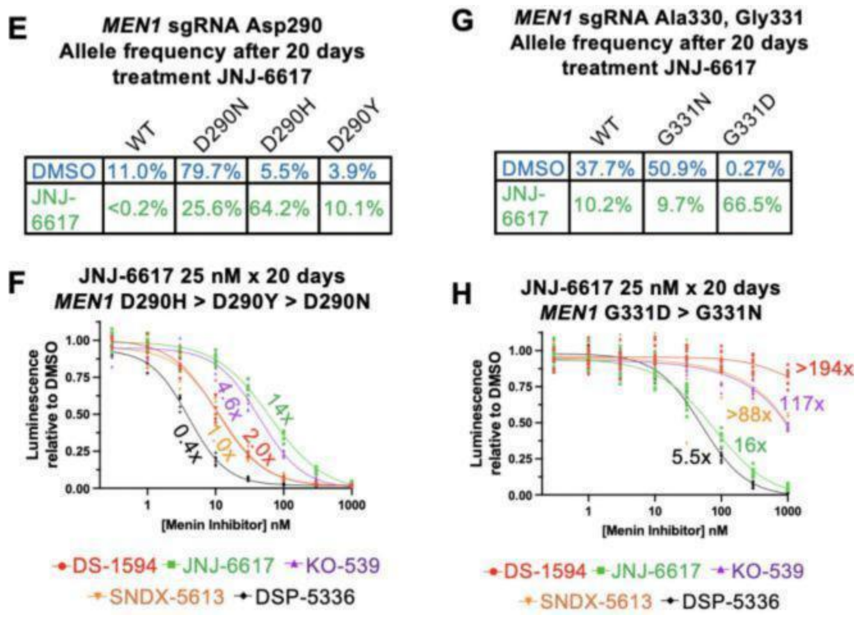

Supplementary Figures 2H-M:

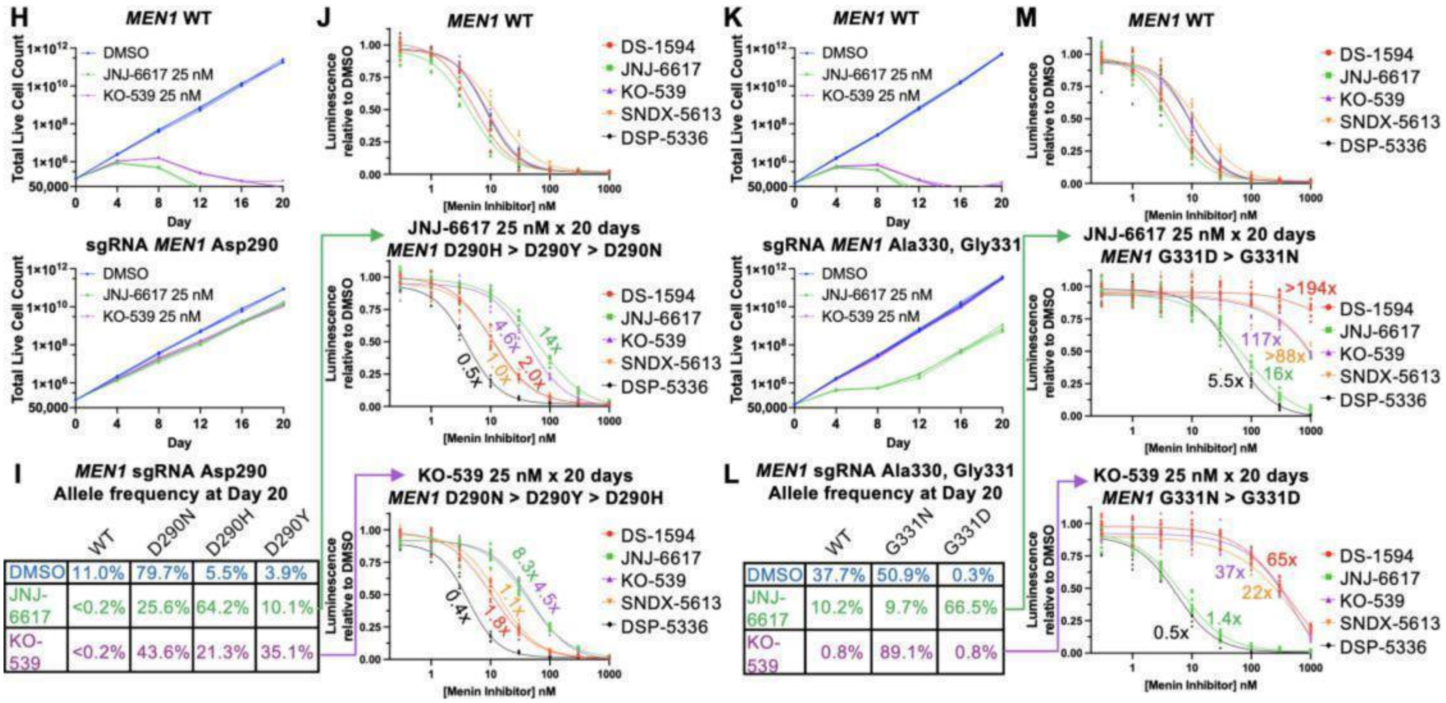

Taken together, these experiments demonstrate that the precise amino acid substitution at a given residue can be critically important in determining resistance. A limitation of our base editing approach is its inability to generate every possible amino acid substitution at a given site. This limitation underscores the importance of using orthogonal validation strategies to fully define the spectrum of *MEN1* mutations influencing sensitivity to each compound. Through this complementary approach, we identified G331D as a pan-resistance mutation across menin inhibitors and specific D290 substitutions as uniquely deleterious to JNJ-6617.

Reviewer #1, Comment 2. In vivo studies with Revumenib were performed by administering Revumenib in chow (in the food) at different drug levels (0.033%, 0.1% and 0.3%), with majority of mice in the 0.3% dose cohort not developing resistance. This way of drug administration, however, raises a question about: (1) the amount of drug being delivered to mice (e.g. is this controlled/equal within the group); (2) PK parameters with this way of drug administration due to constant access/consumption of the drug. This is important in the context of translating the preclinical data to predict the mutations that might occur in patients who are taking the drug orally once- or twice-daily. In vivo studies should be performed using oral drug administration (either in PDXs or at least xenograft models) to resemble the conditions to the clinical set up.

We thank the reviewer for this comment and agree that delivering a dose to mice that is clinically relevant is essential. Prior work from our laboratory, in collaboration with Syndax Pharmaceuticals, established a method for embedding SNDX-5613 in mouse chow to enable continuous drug delivery. Pharmacokinetic analyses in both mice and humans have demonstrated that 0.1% chow produces plasma concentrations in mice closely matching those observed in patients on the AUGMENT-101 trial treated at the recommended phase 2 dose (in combination with a CYP3A4 inhibitor). The figures below, adapted from Issa et al., *Nature* 2023 (PMID: 36922593), illustrate this close correspondence. Horizontal, blue lines highlight the equivalent plasma concentrations between mice receiving chow (left) and patients in clinical trials (right). Notably, due to diurnal variation, drug levels in mice fluctuate modestly and reach a nadir near 2 PM, a pattern that parallels the temporal variation in patients.

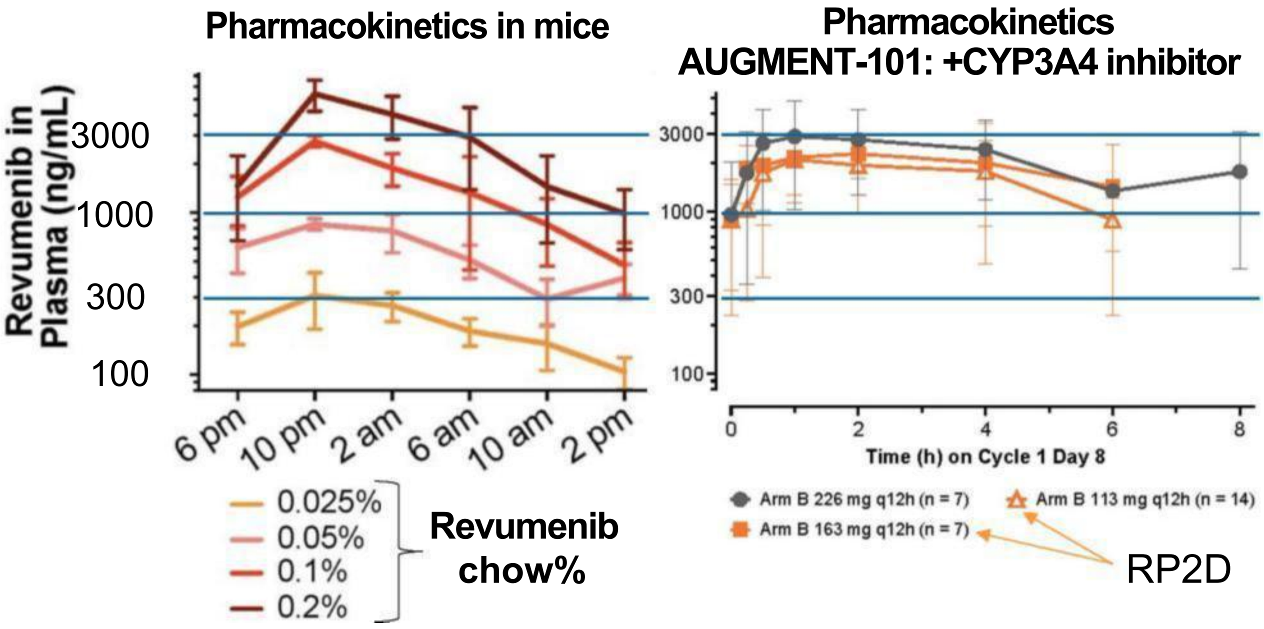

The close alignment between pharmacokinetic parameters in mice and humans likely explains why similar resistance mutations emerge in both settings. Our data suggest that higher dose menin inhibitor therapy (0.3% chow) can prevent or suppress the outgrowth of *MEN1* mutant clones. Although such drug levels may not be achievable with revumenib due to QTc prolongation (the dose-[KMT2-0]limiting toxicity that established the recommended phase 2 dose) other menin inhibitors lacking this
M.X
liability may achieve similarly high degrees of target inhibition.
D

Additional pharmacokinetic analyses in patients confirm that the administered clinical dose produces steady-state plasma levels comparable to those observed in mice receiving 0.1% SNDX-5613 chow

(data presented at the 15th American Conference on Pharmacometrics [ACoP], November 10–13, 2024, Phoenix, AZ; Lin *et al.*, 2024, poster link)

PUBLISHED DATA FIGURE REDACTED

Years of development went into formulating SNDX-5613 in chow in order to recapitulate drug levels observed in patients, with the 0.1% chow dose used in the following publications from our laboratory (either SNDX-5613 or VTP-50469): Krivtsov A et al, *Cancer Cell*, 2019 (PMID: 31821784); Ucklemann H et al, *Science*, 2020 (PMID: 32001657); Heikamp E et al, *Blood*, 2022 (PMID: 34582559); Perner F et al, *Nature*, 2023 (PMID: 36922589).

Syndax Pharmaceuticals has partnered with other laboratories, and this 0.1% chow dose has also been used in publications from outside of our laboratory as well: Carter, B.Z. et al, *Blood*, 2021 (PMID: 34232981); Libbrecht C, et al., *Leukemia*, 2021 (PMID: 33542482); Rasoulie M et al., *Hemasphere*, 2023 (PMID: 37520776)

Alternatively, oral dosing in mice often results in substantially higher peak plasma concentrations than those achieved in humans, followed by much lower trough levels. For example, the preclinical analogue of the menin inhibitor KO-539 (ziftomenib), MI-3454, illustrates this phenomenon (Klossowski S, et al, *J Clin Invest.*, 2020, PMID: 31855575). Below, the peak plasma concentration obtained in mice was nearly 8 uM and the drug entirely cleared by about 18 hours.

PUBLISHED DATA FIGURE REDACTED

In contrast, KO-539/ziftomenib is administered only once daily in patients and the maximal concentration approaches, but does not far surpass, 1 uM: (Wang E, et al, *Lancet Oncology,* 2024, PMID: 39362248). RP2D = recommended phase 2 dose.

PUBLISHED DATA FIGURE REDACTED

1000 ng/mL ziftomenib = 1.4 uM ziftomenib

We include this to illustrate the limitations of oral gavage dosing and to contrast it with the chow-based SNDX-5613 formulation, which achieves plasma levels that closely mirror those in patients.

Reviewer #1, Comment 3. The authors report the co-crystal structures of menin WT or M327I mutant with each inhibitor (Figures 1B, 2D, 4C-G (for menin WT) and Extended Figure 3B (for M327I mutant)) and utilize these data to provide the mechanistic insight into the impact of mutations on inhibitor binding. However, the coordinates and electron density maps for these complexes (both for menin WT and menin mutants in complex with inhibitors reported here) have not been deposited in PDB. This needs to be done and PDB codes need to be provided in the manuscript before acceptance.

We thank the reviewer for these insights and have uploaded these structures and provide PDB ID numbers. We have modified the Methods sections with the following text that addresses the resolution of the x-ray data and provides the PDB ID numbers:

"The co-crystal structures of SNDX-5613 bound menin proteins have previously been uploaded to PDB with the following accession numbers Protein Data Bank (PDB) ID# 7UJ4 (wild-type) and PDB ID#8E90 (I327) and are analyzed here. Co-crystal structures for other inhibitors have also been uploaded to PDB with the following accession numbers, DS-1594 bound menin ID# 9WKW (wild-type) and ID# 9WKX (I327), DSP-5336 bound menin ID# 9WN9 (wild-type) and ID# 9WNA (I327), JNJ-6617 bound menin ID# 9WKU (wild-type) and ID# 9WKV (I327), and KO-539 bound menin ID# 9WNI (wild-type) and ID# 9WNJ (I327)."

Reviewer #1, Comment 4. In line with the above, Tables reporting quality of the collected X-ray data for all menin WT and menin mutant-inhibitor complexes and the refinement statistics need to be provided in the manuscript for revision. This is a standard approach required to validate the quality of the crystal structures reported. This is particularly important here since the mechanism of sensitivity or resistance to the specific mutant is being discussed based on the crystal structures. At this point it

is not even clear what is the resolution of these X-ray data and other critical crystallography parameters are missing.

We agree with this observation and have addressed this concern. To that end, we have added a new Supplementary Table 1 summarizing data collection and refinement statistics, with coordinates and electron density maps deposited in the PDB. We have modified the Methods sections with the following text that describes the resolution achieved:

*"The majority of the structures are of high resolution. Specifically, 6 out of the 8 structures have a resolution of ≤ 2.0 Å, with the best resolution being 1.5 Å for one of the structures (**Supplementary Table 1**). Overall, the ligand and the specific mutation site are well-resolved and show a good fit in the electron density maps."*

Reviewer #1, Comment 5. Presentation of the crystal structures and their discussion in the result section should be improved/more detailed to be more informative. For example, comparison of the menin WT and M327I mutant with specific drugs is shown in the Supplementary Figure 3B and the displacement of inhibitors upon mutation is discussed in the text. However, "the displacement" or "substantial movement" referred in the Result section are not clearly demonstrated in the Figures and there are no details in the text what is the extend of the displacement and which interactions are affected. More details need to be provided to explain the molecular basis of compromised inhibitory activity upon menin mutations.

We thank the reviewer for this comment and have substantially rewritten the paragraph with more details and more precise language. We also improved the visualization of compound displacement in what was previously Supplementary Fig. 3B (now Supplementary Fig. 3C in revised manuscript) using ICM Chemist Pro (MolSoft) to render the x-ray data, which provides clearer visualization.

The new paragraph is in italics below:
*"Given the binding and viability assay data demonstrating that M327I conferred resistance to all five inhibitors, though with varying degrees of resistance, we co-crystallized the I327 mutant menin protein bound to each menin inhibitor to gain structural insights into these differential resistance effects (**Supplementary Fig. 3C, Supplementary Data Table 1**). Superimposing M327 and I327 ligand-bound structures revealed that the backbone of both mutant and wild-type menin protein remains largely unchanged with Root Mean Square Deviation (RMSD) values below 0.4 Å. The b-branching methyl substituent of I327 pointed directly toward the inhibitors in all co-crystal structures similar to what was observed with SNDX-5613 bound to I327,[15] however, adjustments in the relative positions of the inhibitors compared to wild-type varied. DS-1594, KO-539, and SNDX-5613 all bind in an extended conformation, reaching across the M327 position toward the distal end of the binding pocket, which results in more pronounced distortion of the binding pose seen in wild-type (**Supplementary Fig. 3D-H**). Neither JNJ-6617 nor DSP-5336 showed significant changes to their overall binding pose. Consistent with previous observations about JNJ-6617,[6] and now seen with DSP-5336, both bind in conformations that avoid M327, which likely explains why the I327 protrusion causes much less displacement and binding similar to wild-type. This structural analysis suggests that the magnitude of M327I resistance depends on how each inhibitor uniquely engages the binding pocket, with KO-539 and SNDX-5613 most affected, followed by DS-1594, and JNJ-6617 and DSP-*

*5336 least affected, consistent with the hierarchy observed in our binding and cellular systems (**Figure 4B**).”*

Previous Supplementary Fig. 3B, now Supplementary Fig. 3C:

***3C.** Co-crystal structure overlays of menin inhibitors bound to wild-type menin (M327) and mutant menin (I327).*

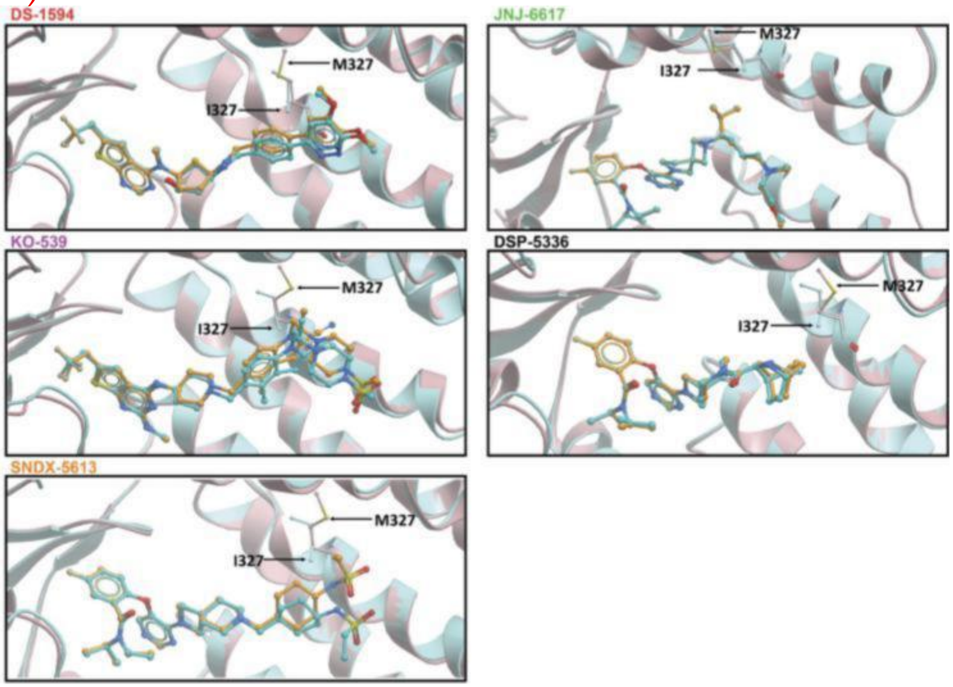

Additionally, we added a new analysis that shows the measurement distances between the mutant ligand pose and the wild-type pose to further display the positional changes we were able to resolve in the x-ray data.

New supplementary Figures 3D-H:

***3D-H**. Co-crystal structure overlays of menin inhibitors bound to wild-type menin (M327) and mutant menin (I327) either **D.** JNJ-6617, **E.** DSP-5336, **F.** DS-1594, **G.** KO-539, or **H.** SNDX-5613, with quantitative coloring reflecting the magnitude of positional changes due to the M327I mutation mapped onto the atoms (excluding hydrogens) of each inhibitor. Mapping is only displayed on inhibitors bound to the I327 mutant structures and the wild-type bound position is translucently colored in grey to display the positional changes. Only amino acid side chains I327 (magenta) and M327 (grey) from the corresponding proteins are displayed. DS-1594 bound menin ID# 9WKW (wild-type) and ID# 9WKX (I327), DSP-5336 bound menin ID# 9WN9 (wild-type) and ID# 9WNA (I327), JNJ-6617 bound menin ID# 9WKU (wild-type) and ID# 9WKV (I327), and KO-539 bound menin ID# 9WNI (wild-type) and ID# 9WNJ (I327).*

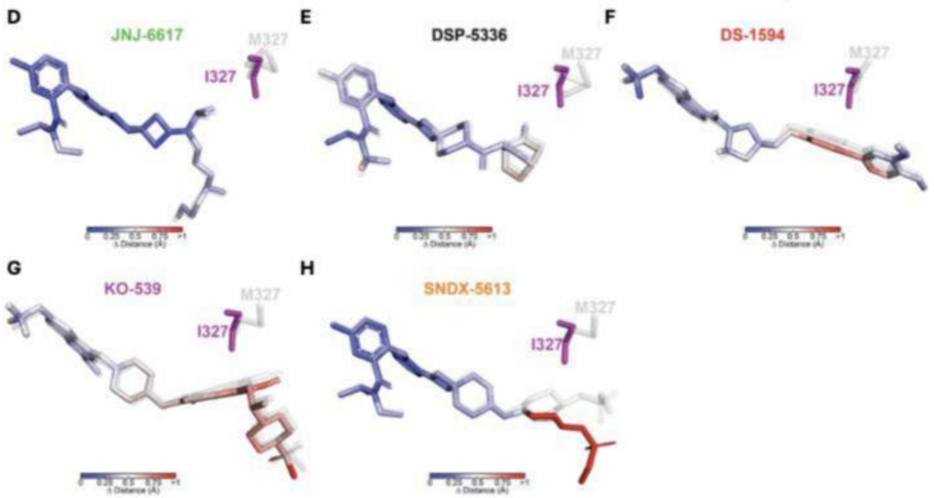

We will also add the following sentences to the methods section describing this analysis:

"M327 vs. I327 menin inhibitor positional analysis and visualization was performed in PyMOL (Schrödinger, LLC). Each unique menin inhibitor bound M327 and I327 protein co-crystal structure pairings were aligned using the 'align' command. These alignments compared the positions of all Cα atoms deriving Root Mean Square Deviation (RMSD) values to quantify backbone similarity. Derived RMSD values for M327-I327 pairs were as follows: KO-539/0.368 Å, DSP-5336/0.273 Å, DS-1594/0.401 Å, SNDX-5613/0.338 Å, and JNJ-6617/0.321 Å. To enable menin inhibitor positional comparisons due the M327I substitution, distances between the matched atom pairs of the bound ligands were measured in Euclidean distance (Å) using the coordinates derived from the 'cmd.get_atom_coords' function. For Supplementary Fig. 3D-H these values were used to generate heatmap coloring of the mutant bound ligand, using the 'spectrum b, blue white_red' function with a minimum of 0 Å and maximum of 1 Å (values greater than 1 Å were capped to enhance smaller displacements) and then the wild-type bound ligands were colored with the 'set stick_transparency' function to illustrate the movement in the pocket."

Reviewer #1, Comment 6. The synthesis of drugs is not provided in the method section, but instead the authors refer to some patents. However, some of the references cite PubChem source (e.g. for Enzomenib, reference 17) rather than specific patent or publication. The authors should either report the entire synthesis of each drug or clearly indicate which publication or patent was used to prepare a specific drug. In any case, the analytical data for final products (e.g. each specific menin inhibitor) from the synthesis should be provided (NMR, HRMS) to confirm the identity and purity of these compounds.

We thank the reviewer for this comment. We have included the analytical data for all compounds synthesized (**Supplementary Dataset 1**) that has summaries of the results of the NMR data resolving the purity/identity of the compounds that we used in our study. We have cited the patent in which the synthesis of the DSP-5336 compound is described as well: Fukuda, Y., Yata, K., Hayashi, K., et al. Menin inhibitors and methods of use. U.S. Patent Application US20200157114A1. Published May 21, 2020.

The manuscript text now reads: "*All menin inhibitors used in this study were prepared based on synthetic routes described in the patent applications for the respective compounds, with synthesis routes for DS-1594, JNJ-6617, and KO-539 subsequently published (**Supplementary Dataset 1**).*" The citations listed are for the publications describing the synthesis of each compound and then the patent application where DSP-5336 synthesis was found.

Reviewer #1, Comment 7. The RNA-seq data should be deposited to GEO before re-submission and the entries should be provided to the reviewers.

We thank the reviewer for this comment and the GEO reviewer access code for the RNA-seq data is listed here: GSE294623. We have added this GEO code to the methods section as well.

Reviewer #1, Comment 8. The MEN1-focused CRISP-Cas9 screen revealed various mutations in MEN1, either unique or common for each drug. However, the in vitro studies to validate the impact of mutations on inhibitor binding in the TR-FRET assay are only presented for M327I and T349M mutants. Other mutants (e.g. Gly331, Asp 348, Glu336, etc.), which affect inhibitory activity for majority or some of these drugs, should also be characterized using in vitro biochemical assay.

We thank the reviewer for suggesting additional biochemical assays for additional *MEN1* mutations. We have included data for the G331R mutation, which has arisen in patients (Perner et al, *Nature*, 2023) as well as in our orthogonal validation experiments (Main Figure 6A). This data was obtained using a KMT2A(MLL1)-FITC probe, in the same manner the previous data pertaining to M327I and T349M was obtained in our initial submission.

Additionally, in collaboration with the laboratory of Eric Fischer, we performed new TR-FRET experiments to biochemically confirm loss of binding affinity for selected novel mutations identified in the CRISPR base editor screen. Using site-directed mutagenesis of the wild-type pERT-Menin-Avi-StrepII bacterial expression vector, we generated three constructs harboring single amino acid substitutions, C334R, E368K, or V372A. Mutant menin proteins were purified and subjected to TR-FRET analysis using methods and experimental conditions that differed slightly from those used in earlier experiments, as described in detail in the updated Methods section. For these assays, we used a previously published KMT2B(MLL2)(15–48)-FITC probe, which we have shown serves as an appropriate surrogate for a KMT2A(MLL1)-FITC probe, as reported by Perner et al., *Nature*, 2023. Probe affinities, K_d, for each menin protein were determined by counter-titration with an unlabeled KMT2B(15–48) peptide, and these values were subsequently used to estimate binding affinities for all menin inhibitors evaluated in this study. All data were normalized to the corresponding wild-type protein to enable comparison of multiple mutations on a shared axis. Because our primary objective is to quantify relative binding differences between wild-type and mutant menin proteins, we consider it appropriate to combine datasets generated using these distinct experimental conditions. Accordingly, we present the results as delta K_i values, defined as the ratio of mutant K_i to wild-type K_i, using the experiment-specific wild-type K_i values as the denominator.

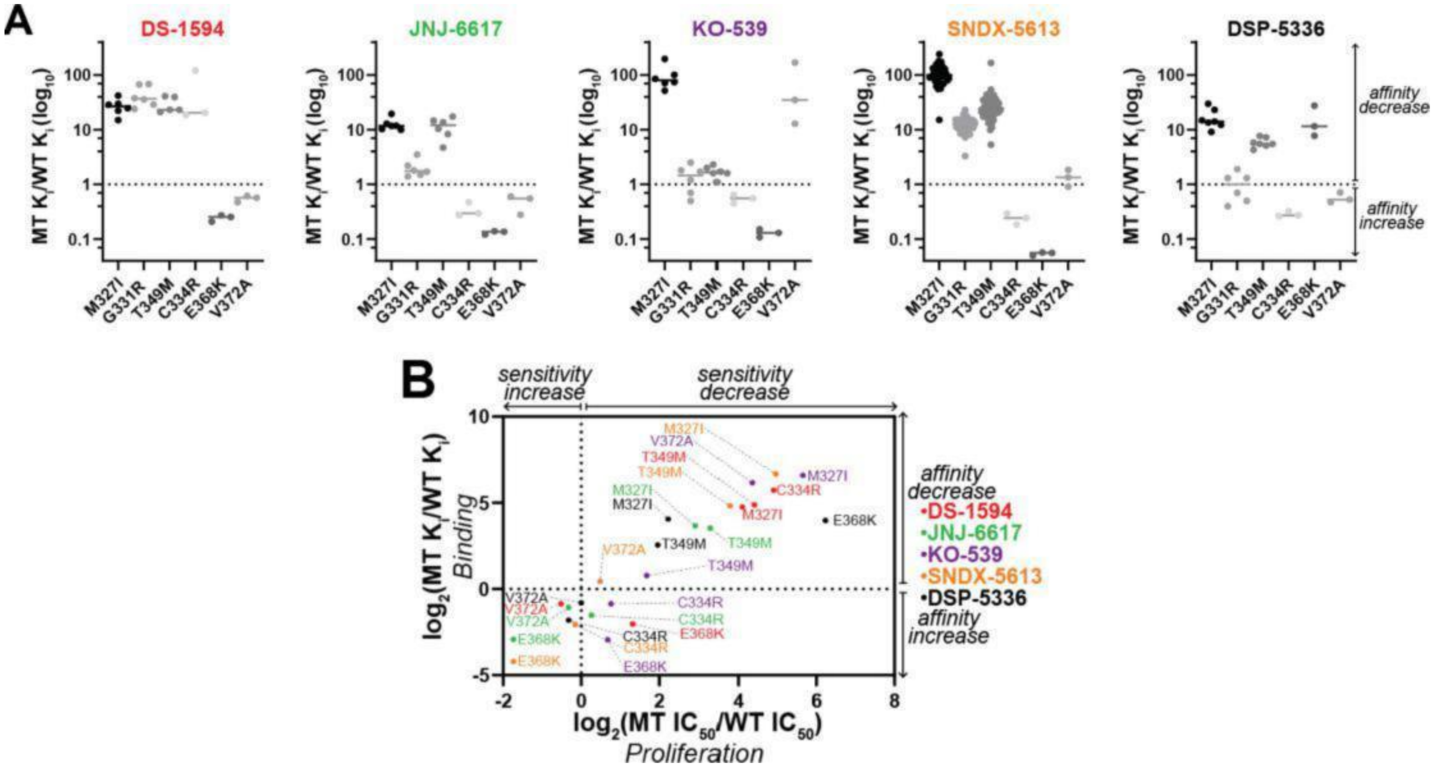

We have also included new graphs in Supplementary Figure 3A that display wild-type and mutant proteins stratified by experimental design, together with a statistical analysis better suited for biochemical affinity comparisons in pKi, specifically log transformation of Ki values followed by one-way ANOVA on the transformed data.

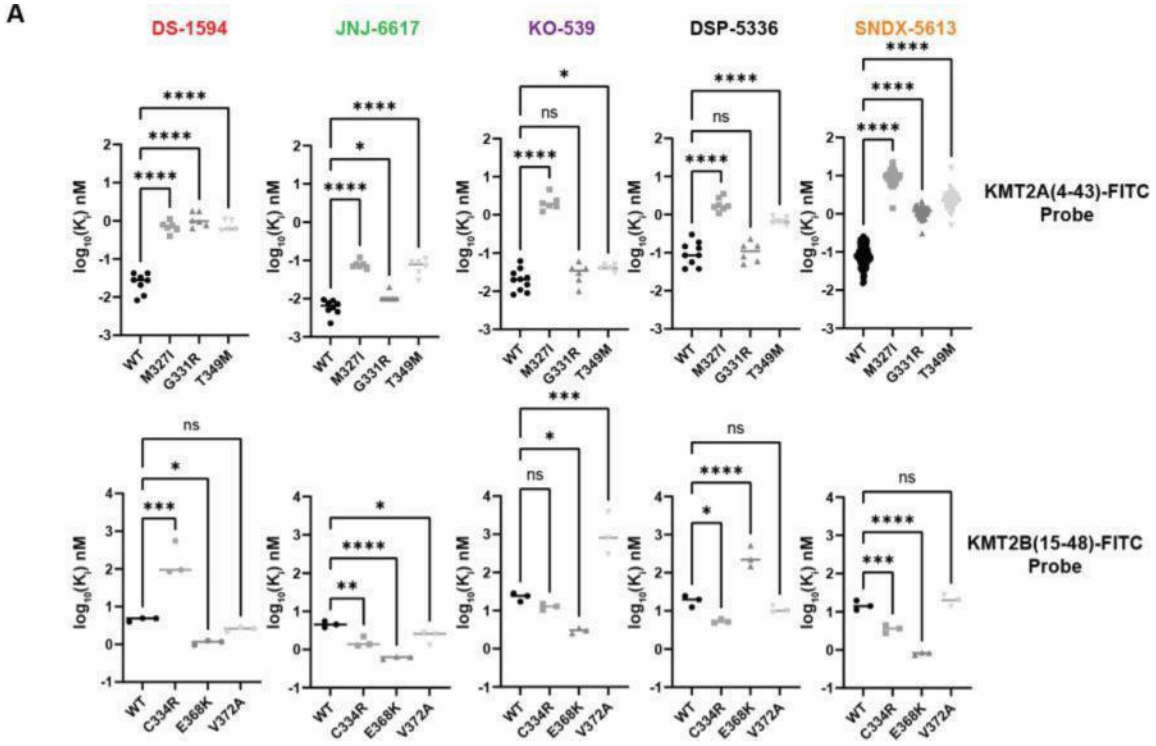

Additionally, we have provided dose-response curves that were used to calculate IC_{50} values for subsequent K_i calculations. Data generated in collaboration with Syngene Ltd. (WT, M327I, T349M, and G331R) are presented as percent inhibition, with all replicates displayed on a single graph. Data generated in collaboration with the Fischer laboratory (WT, E368K, V372A, and C334R) are presented with the three independent experimental runs shown separately.

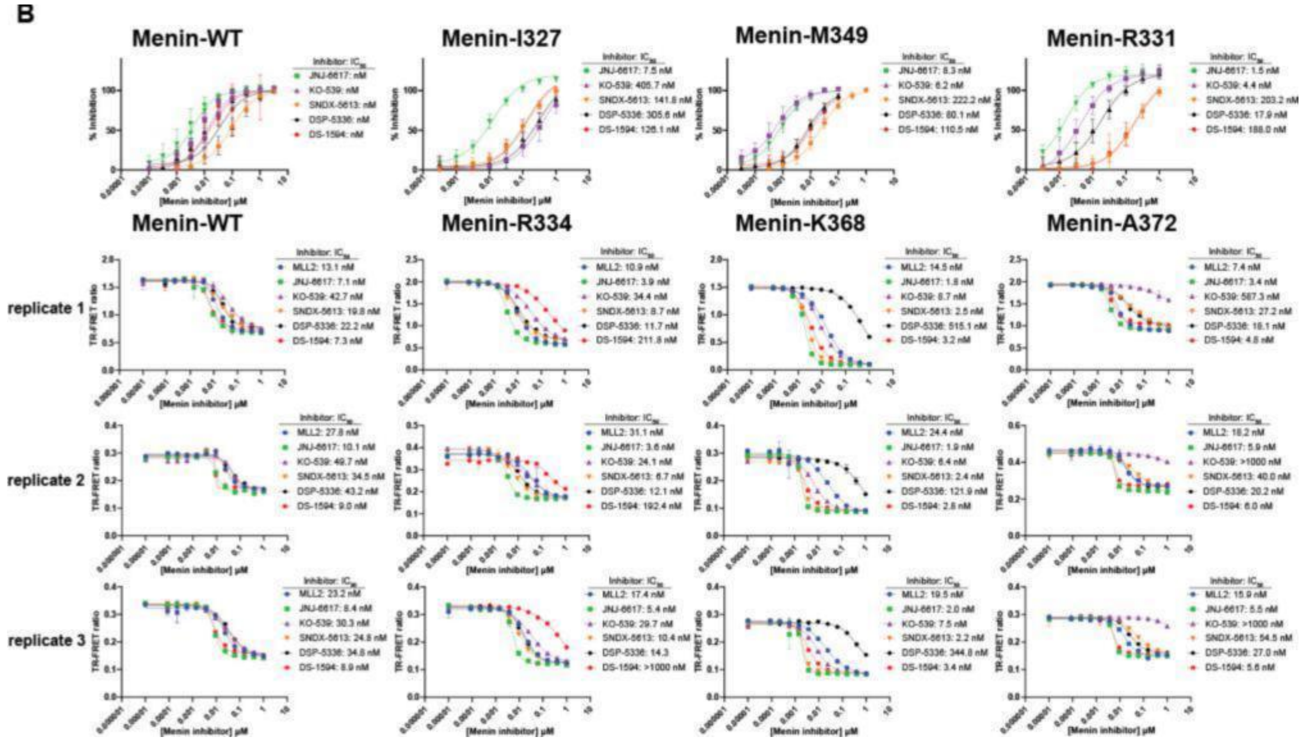

Finally, we have updated the Methods section to include detailed descriptions of these experiments.

Reviewer #2 (Remarks to the Author):

Manuscript describes systematic studies evaluating activity and resistance mechanisms to 5 clinical stage menin inhibitors. By employing CRISPR-Cas9 base editing approach they discovered multiple mutations in MEN1 arising to all tested menin inhibitors. To explain effect of mutations, they determined multiple crystal structure of menin with small molecule inhibitors. They also evaluated activity of menin inhibitors against various cell lines with MEN1 mutations and some animal models. Overall, these studies are very systematic, comprehensive and high quality. Authors demonstrate that similar mutations will likely occur to all menin inhibitors which are currently in clinical studies or to FDA approved revumenib. Extensive mutational data might inform on potential direction for development of the next generation of menin inhibitors to minimize impact of MEN1 mutations. Another important conclusion is that better potency of menin inhibitor might limit or even prevent the occurrence of MEN1 mutations. However, the key limitation of this manuscript is limited novelty. Activity of many tested menin inhibitors have been already reported in original publications describing discovery of these drugs. Same authors previously published CRISPR editing mutagenesis showing generation of mutations in MEN1 resulting in resistance to revumenib (Perner et al 2023).

We thank Reviewer #2 for their comments, which we have responded to with additional experiments below that we believe strengthens the paper.

With regards to the limited novelty as it pertains to the CRISPR editing mutagenesis screen, please see our response in the "Reviewer #1 (Remarks to the Author):" section above.

This manuscript could be strengthened by addressing following points:

Reviewer #2, Comment 1. - one of the most consequential conclusions is that more potent menin inhibitor can decrease the rate or even limit occurrence of MEN1 resistant mutations. This conclusion

is based on cell-based studies and I wonder whether authors could confirm this in vivo, by treating PDX mice with two different inhibitors. This could significantly increase the novelty.

We thank the reviewer for this comment and agree with the reviewer that higher doses suppressing *MEN1* mutation outgrowth is a key finding with translational potential. In our initial submission, we showed one PDX experiment where high-dose SNDX-5613 prevented *MEN1* mutation development (CBAM-44728), and another in which a PDX harboring a patient-acquired *MEN1* M327I mutation was able to be cured with high-dose SNDX-5613. In our resubmission, we have strengthened this finding with an experiment demonstrating that JNJ-6617 also suppresses *MEN1* mutation development at higher doses *in vivo* (see **Reviewer #1, Comment 1**; see new Main Figure 6B+C). At a lower dose of JNJ-6617 (30 mg/kg/day), 4 of 4 mice developed *MEN1* mutations after up to 75 days on treatment, versus only 1 of 4 mice treated with a higher dose of JNJ-6617 (100 mg/kg/day).

We did not combine menin inhibitors *in vivo*. As discussed in **Reviewer #1, Comment 2**, only JNJ-6617 has published pharmacokinetic values, and the price of purchasing menin inhibitors other than SNDX-5613 is quite expensive. Given the high cost, we prioritized evaluating whether JNJ-6617 would generate mutations predicted by our base editor screen *in vivo* in a PDX model. We included the higher dose cohort (100 mg/kg/day) in part to further test the hypothesis that higher dose menin inhibitor therapy can suppress *MEN1* mutation development.

We further attempted to demonstrate the effect of combinations of menin inhibitors to suppress *MEN1* mutations *in vitro*, leveraging our ability to generate spontaneous *MEN1* mutations when exposing large numbers of cells to drug. In each of two independent experiments, we seeded >12.5 million cells per replicate, treating 5 replicates per experiment with either JNJ-6617 25 nM or DSP-5336 25 nM in addition to 6 replicates with the combination of the two menin inhibitors at 25 nM. We picked JNJ-6617 and DSP-5336 because JNJ-6617 retains substantial activity against E368 mutations that markedly attenuate the DSP-5336 compound while DSP-5336 is the most active against M327 mutations, G331D mutations, and T349M mutations that all affect JNJ-6617 (Main Figure 3A+B, 3E-H). In these experiments, we saw outgrowth of one *MEN1* resistant clone, a *MEN1* G331D mutation arising with JNJ-6617 25 nM. We saw two clones outgrow when treated with DSP-5336, both of which were *MEN1* WT. No resistant clones emerged with the combination of both menin inhibitors. This data is included as new Supplementary Fig. 4A+B.

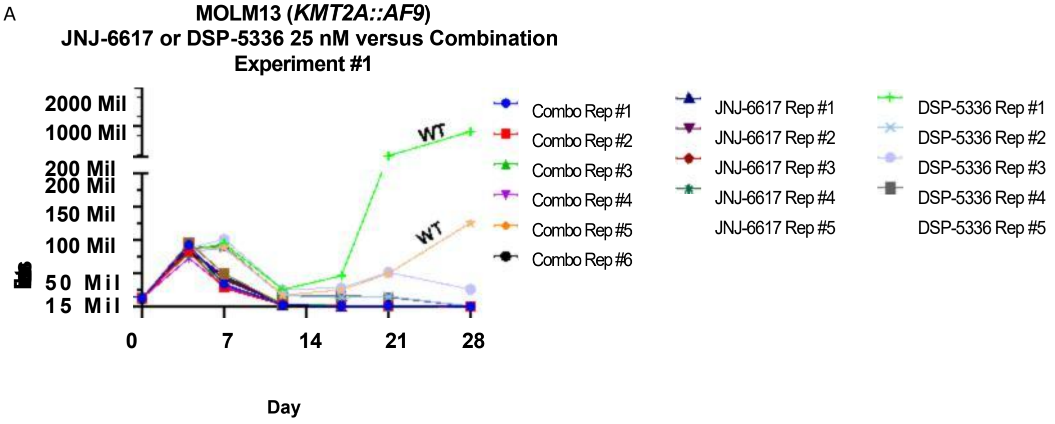

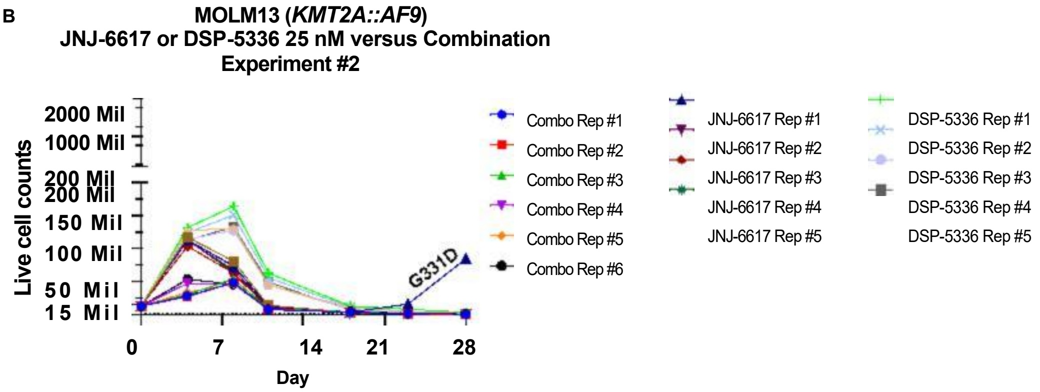

Further, the G331D mutation exhibited pan-resistance to all menin inhibitors, and this data is included within Main Figure 5F.

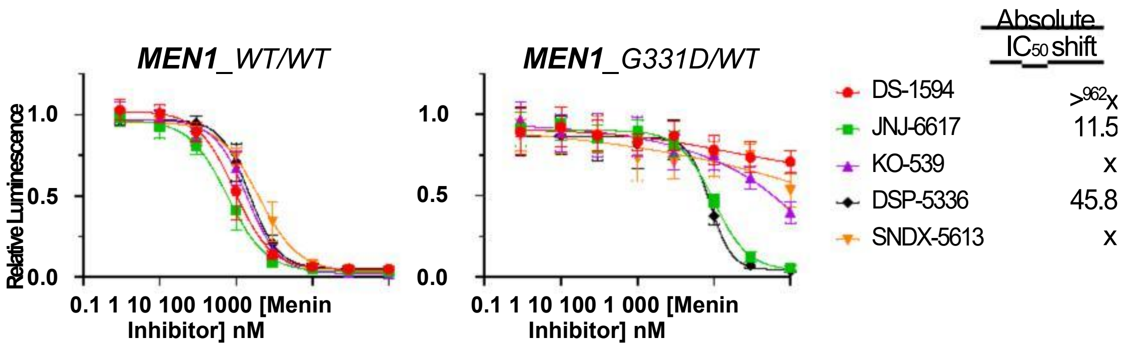

Considering these data alongside our previous results in Main Figure 5, treatment with JNJ-6617 (25 nM) led to *MEN1* mutations in 4 of 22 clones by day 28 (M327V ×2, T349M, G331D), while DSP-5336 (25 nM) produced *MEN1* mutations in 2 of 22 clones (E368K, E368V) and two additional clones without *MEN1* mutations. In contrast, none of the 12 replicates treated with the JNJ-6617 and DSP-5336 combination developed resistant clones. We have noted this ability of menin inhibitor combinations to suppress outgrowth of resistant clones, stating: "*In contrast to menin inhibitor monotherapy selection of resistant clones, we also treated twelve replicates with the combination of JNJ-6617 25 nM and DSP-5336 25 nM, finding no outgrowth of resistant clones (**Supplementary Fig. 4A+B**).*"

Reviewer #2, Comment 2. - Authors show the results of the in vivo experiment in PDX model CBAM-44728, where continuous treatment with SNDX-5613 administered in chow, results in occurrence of MEN1 mutations in dose dependent manner. However, patient treatment is different, and the drugs are administered q.d. or b.i.d., which when compared to chow based administration, leads to different PK profiles, such as absorption, Cmax, half-life, clearance etc. In consequence, drug exposure and menin inhibition profile might significantly differ from q.d. or b.i.d. administration, and thus impact development of MEN1 mutations. It would be valuable for the current study to evaluate development of MEN1 mutations using drug administration that is similar to patient treatment.

Certainly, matching the dose levels received by mice in PDX studies to that seen in patients is essential. Please see **Reviewer #1, Comment 2** above where we address the remarkable ability of SNDX-5613 embedded in menin inhibitor chow to recapitulate the pharmacokinetic parameters observed in patients. Briefly, we believe that the dose of 0.1% chow very closely approximates the dose levels observed in patients. Additionally, we demonstrated that *MEN1* mutations arise with a

different menin inhibitor dosed with oral gavage (JNJ-6617), see **Reviewer #1, Comment 1** above and new Main Figure 6B+C**.**

Reviewer #2, Comment 3. - authors describe comparison of 5 menin inhibitors and conclude that potency of these inhibitors plays a role in development of MEN1 resistant mutations; it would be important to include additional discussion how drug-like properties such as PK, dose limiting toxicity might impact development of drug resistant MEN1 mutations.

We thank the reviewer for this comment and in our discussion we have addressed this point, adding the following language to the discussion: *"Whether sufficiently high dose menin-KMT2A inhibition to suppress MEN1 mutation development remains to be seen. Our work suggests that higher dose levels may be helpful with regards to suppressing MEN1 mutation-driven resistance."*

Interestingly, in an analogous setting of ABL kinase domain mutations arising during tyrosine kinase inhibitor (TKI) therapy for chronic myeloid leukemia, Jabbour et al. (*Blood*, 2008; PMID: 19060245) showed that patients experiencing cytogenetic failure could regain cytogenetic responses when the imatinib dose was doubled, despite the presence of certain ABL kinase domain mutations. However, this benefit was tempered by toxicities, particularly hematologic effects and fluid retention, that often required dose reduction.

Reviewer #2, Comment 4. - another important question that has not been addressed in this study is whether these MEN1 mutations are de-novo resulting from drug treatment, or are already present as minor clones, which are selected upon drug treatment

We thank the reviewer for highlighting this important question of whether these mutations are pre-existing or selected during drug treatment. In prior analyses using droplet digital PCR (ddPCR) to detect *MEN1* T349M mutations in patients and PDX models, including the CBAM44728 model, these mutations were not detected (Perner F et al., *Nature*, 2023; PMID: 36922589).

Given that ddPCR did not previously detect T349M mutations, despite this being the most common mutation observed in the CBAM44728 model, we did not pursue additional ddPCR analyses. Instead, we sequenced amplicons covering *MEN1* mutation hotspots (S160, D290, M327, G331, T349) from the CBAM44728 model using the Oxford Nanopore Technologies platform (Plasmidsaurus) in two vehicle-treated mice from the same PDX. At a sensitivity threshold of 0.1% of all reads, no mutant sequences were detected. The number of reads exceeding 0.1% frequency at each hotspot in each mouse is shown below.

Reads per mouse per hotspot ≥0.1% of all reads in sample	S160	D290	M327	G331	T349
Vehicle mouse 1	3998	4194	3293	3293	3233
Vehicle mouse 2	4137	4279	3755	3755	3699

In CML, many papers support the notion that ABL kinase domain mutations are pre-existing prior to starting treatment. Paradoxically, this has not been shown to correlate with patient outcomes (Jabbour E, et al, *Hematol Oncol Clin North Am*, 2011; PMID: 22054730).

Further, it is informative to consider the possibility of pre-existing mutations in light of our in vitro MEN1 mutation generation approach, which involves exposing large numbers of cells to drug. Across several experiments, we identified six *MEN1* mutant clones comprising five distinct nucleotide changes prior to Day 28, including M327V (n = 2), G331D (n = 1), T349M (n = 1), E368K (n = 1), and E368V (n = 1). Given the observed cellular doubling rates, these mutations were either pre-existing within the starting population or arose very early after initiation of drug treatment.

Whether these mutations are pre-existing and whether they affect eventual patient responses is certainly an area of future inquiry.

Reviewer #2, Comment 5. - Presentation of figures showing binding modes of menin inhibitors makes is difficult to see key interactions with menin. In addition, crystal structure of menin complexes should be deposited in PDB.

The crystal structures are now deposited to PDB. For further explanation see our response to **Reviewer #1, Comment 4**. We have remade many of the structural figures to improve clarity based on this helpful feedback, see **Reviewer #1, Comment 5.**

Reviewer #3 (Remarks to the Author):

Review of Bourgeois et al NatComms

Summary

Bourgeois et al use CBE and ABE base editing screens of MEN1 to investigate potential mechanisms of resistance to 5 MEN1 inhibitors for the treatment of leukemia. The authors validate selected resistance hits with cell proliferation, biophysics and infer mechanism on the disruption of inhibitor binding with the use of protein crystal structures. Importantly, the authors highlight key residues in MEN1 where resistance differs between drugs – some of which are found in PDX models. A weakness of the paper analysis of the base editing screen and the lack of mechanistic insight. The authors could strengthen the paper by considering the following points.

Main points

Reviewer #3, Comment 1. • The authors use log fold change throughout, which is fine, but due to the noise in the data, they should add statistics. DrugZ or MAGeCK would be appropriate to include FDR and p-values.

Thank you for the feedback regarding the presentation of the screen and the suggestion to consider statistical testing. We have re-analyzed the screen using the MAGeCK pipeline, the results of which are shown below on the right, with our initial screen results shown below on the left as a comparison.

***MEN1* CRISPR-Cas9 base editor screen sgRNA abundance**
Day 21 vs Day 0, relative to DMSO control

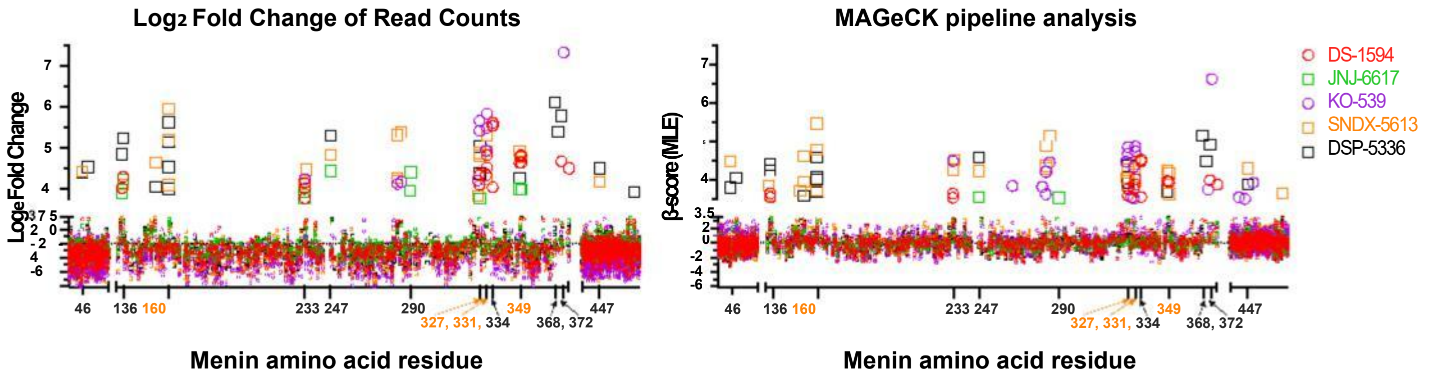

The results from the two analyses were highly consistent. As suggested, this comparison enabled calculation of FDR and p-values for each individual reagant. When applying these metrics, however, only one guide per drug condition met both the $p < 0.05$ AND $FDR < 0.05$ thresholds. While dozens of guides met the p-value criterion, only a single guide per condition reached significance after FDR correction. This outcome reflects the distinction between gene-focused screens, which MaGECK and DrugZ were designed to analyze, and the design of this screen, in which each guide necessarily serves as an independent replicate rather than multiple guides targeting the same gene. Unlike genome-wide CRISPR screens, where several guides target a single gene, each guide in this base-editing screen produces distinct edits at unique genomic positions. Pooling guides predicted to edit the same residue was not feasible, as not all guides edited the intended residue and sometimes targeted adjacent positions. Thus, because guides are not true replicates and cannot be pooled, statistical significance would be expected only for the most extreme outliers, which is precisely what we observed. Indeed, other base editor screens in the examining drug resistance, such as a recent study by Coelho and colleagues (Coehlo MA, et al., *Nat Genet.* 2024; PMID: 39424923) also do not apply such statistical tests, likely for the same reason.

			MAGeCK Pipeline Analysis				Analysis in paper: Log2 Fold Changes (Log2 FC)	
Menin Inhibitor	Predicted amino acid edit	A>G or C>T screen	Beta score relative to DMSO	p-value	FDR	Rank amongst positively-selected Beta-scores	Log2FC value	Rank amongst positively-selected Log2FCs
DS-1594	Cys334Arg	A>G	4.53	0	0	1	5.62	1
	Gly331Asn	C>T	4.04	0	0	1	4.82	1
JNJ-6617	Ser160Pro	A>G	2.07	0	0	1	2.78	1
	Ala247Thr	C>T	3.57	0	0	1	4.45	1
KO-539	Val372Ala	A>G	6.63	0	0	1	7.34	1
	Met327Ile	C>T	4.85	0	0	1	5.66	2
SNDX-5613	Ser160Pro	A>G	5.47	0	0	1	5.95	1
	Gly331Asn	C>T	4.43	0	0	1	5.29	1
DSP-5336	Ser160Pro	A>G	4.59	0	0	1	5.63	1
	Lys367Lys, Glu368Lys	C>T	5.14	0	0	1	6.12	1

We did not include this alternate analysis of the base editor screen in the manuscript, although it is reassuring that it yielded the same top hits. The rationale for this is that we selected individual guides for validation based on the Log_2 fold change (guides with a Log_2 fold change ≥4) and performed

extensive follow-up studies introducing individual guides into cell lines. The resulting phenotypes were evaluated through 9-day competition assays (Supplementary Fig. 2C), measurement of absolute IC_{50} shifts (Main Fig. 3A+B), and orthogonal validation of multiple predicted hits in independent experiments (Fig. 5, Fig. 6A, new Fig. 6B). Together, these data provide strong evidence that the screen accurately predicts mutations capable of arising *in vivo* and attenuating menin inhibitor activity.

Reviewer #3, Comment 2. • Extended data 1C shows that the average log2fold change in the presence of drug is around -2? That suggests most mutations are strongly deleterious. Without proper comparison to controls in the library such as intergenic/non-targeting guides or positive controls, it is hard to know whether this signal is biological or technical.

We thank the reviewer for their question regarding the average $\log_2$ fold change in the screen (approximately -2) and for suggesting inclusion of data on the performance of positive-control and intergenic guides. We have incorporated this analysis into the revised manuscript.

The number of guides with large negative scores by Log_2 fold change (Log_2FC) primarily reflects a technical artifact resulting from the strong drug pressure and the duration of the screen. As shown in Supplementary Fig. 1C, no guide in the DMSO condition depleted more than $\text{Log}_2\text{FC} < -1.34$, and only five fell below -1. A guide targeting menin that confers neither a positive nor negative effect on drug resistance will rapidly deplete under drug treatment due to the drug's cytotoxicity. As shown in Figures 5A and 5D, cells treated with menin inhibitors at concentrations comparable to those used in the screen are largely nonviable and nonproliferative by day 14. Consequently, by the end of the 21-day screen, only guides conferring some degree of drug resistance avoid large negative Log_2FC values.

We appreciate the reviewer's suggestion to include the performance of the positive and negative control guides. These analyses are now provided in Supplementary Figs. 1D and 1E. The results further support that strong drug selection, rather than guide-specific effects, drives dropout in this screen.

In Supplementary Fig. 1D, we compare the performance of all *MEN1*-targeting guides in the DMSO arm of the A>G and C>T screens with that of intergenic control guides and positive-control guides targeting essential genes (*RPA3, PCNA, DBR1, PLK1, RPL3, GAPDH, KIF11, PSMB1, EEF2,* and *POLR2B*). Under DMSO conditions, when comparing day 21 to day 0, intergenic controls behave similarly to *MEN1*-targeting guides but lack the modest tail of negatively depleting guides beyond roughly Log_2FC -0.25 observed among the *MEN1* guides. The positive-control guides targeting essential genes show mild negative enrichment, consistent with limited depletion, which is likely attributable to the base-editing enzymes' editing efficiency.

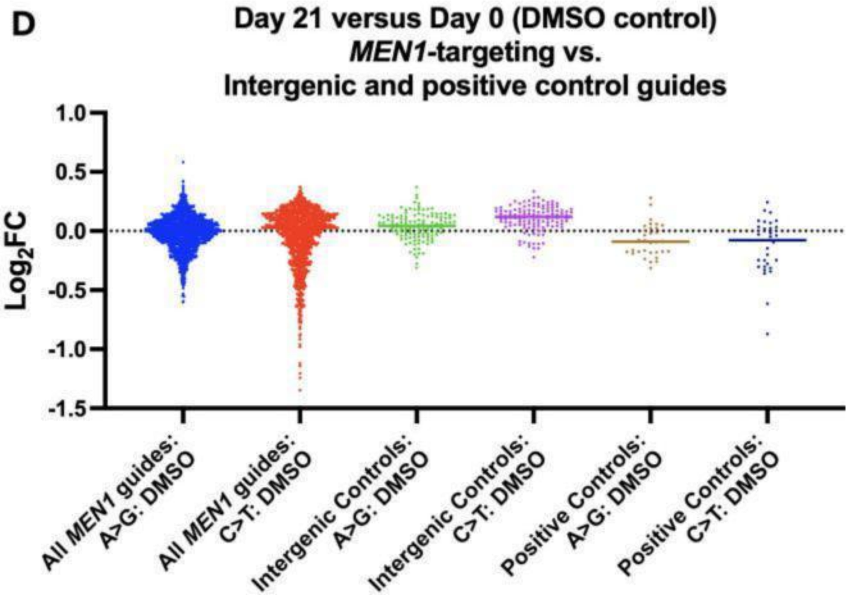

We next examined the performance of guides targeting intergenic controls and positive controls in the drug-treated A>G and C>T arms of the screen, shown below and now presented as Supplementary Fig. 1E.

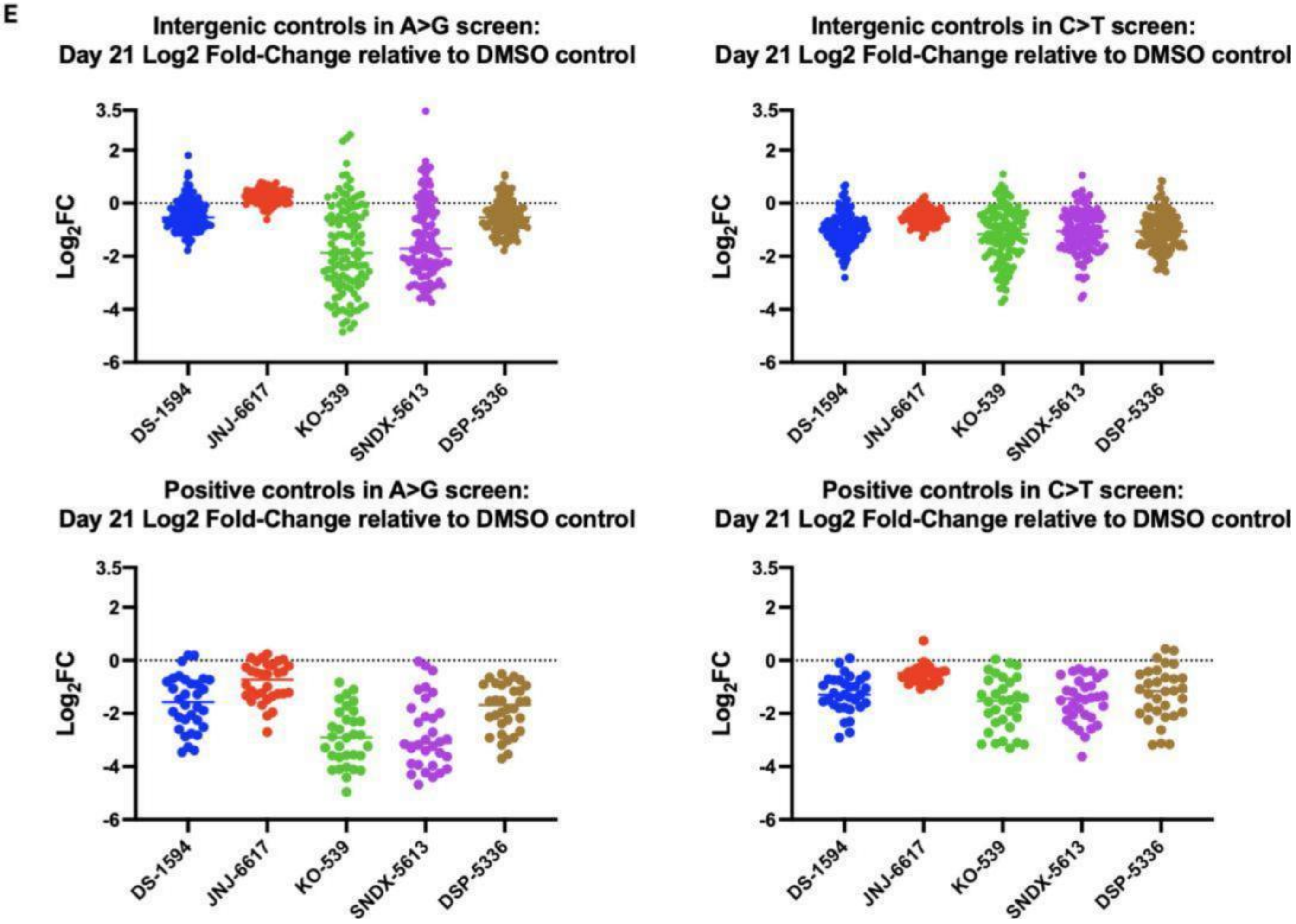

Depletion of intergenic controls was greatest with KO-539 and least with JNJ-6617, most notably in the A>G arm of the screen. This likely reflects two factors: the intensity of drug pressure and the

number of positively selected guides. In the A>G arm of the screen for JNJ-6617, no guide enriched more than Log$_2$FC +3, and only 13 guides exceeded Log$_2$FC +2. In contrast, in the KO-539 A>G arm, one guide enriched above Log$_2$FC +7, four above +4, five above +3, and 16 above +2. Regarding selective pressure, the drug effect was weakest in the JNJ-6617 A>G arm and strongest in the KO-539 A>G arm, despite efforts to normalize potency across conditions. By day 11, the JNJ-6617 A>G arm showed 12.7-fold growth relative to day 0 with 58.3% cell viability, whereas the KO-539 A>G arm showed only 4.8-fold growth and 38.8% viability. Thus, the KO-539 arm experienced a stronger bottleneck driven by both higher drug potency, as reflected by reduced growth and viability, and a larger number of guides showing pronounced Log$_2$FC enrichment.

Reviewer #3, Comment 3. • Figure 3 – 37 out of 80 cell lines validate the screens, what is this in numbers of gRNAs? For drug resistance (positive selection), this seems a low rate of validation – can the authors comments why this might be? Recall of known lethal variants or known resistance variants is often done to understand sensitivity.

We thank the reviewer for this comment and agree that our description of the number of validating cell lines was unclear. We have clarified this in both the Supplementary Fig. 2C legend and the manuscript text. In brief, 40 individual guides showing strong enrichment (Log$_2$FC > 4.0) were selected for validation as single guides in independent cell lines. Some scored in the A>G editor arm, others in the C>T arm, and several in both. Rather than testing guides only in the arm where they initially scored, we transduced all 40 guides into both the A>G AND C>T editor arms. Only 8 of the 40 did not meet our predefined enrichment thresholds for enrichment of *MEN1* mutant cells compared to wild-type cells (starting from a mixture of 20% mutant cells and 80% wild-type cells): ≥3x enrichment (>60% *MEN1* mutant, <40% *MEN1* WT on day 9) with at least two menin inhibitors, or ≥4.25x enrichment (>85% *MEN1* mutant, <15% *MEN1* WT on day 9) with one inhibitor. These criteria are relatively stringent for a 9-day assay, especially given that the screen itself was conducted over 21 days. Some guides that did not meet the threshold may have produced low-frequency edits requiring longer exposure to enrich. Accordingly, some guides that failed to validate in the 9-day competition assay may have scored with longer treatment durations. Supporting this, when we relaxed the enrichment threshold to a twofold increase for at least one drug, only 3 guides failed to validate in either the A>G or C>T arm.

We have edited this sentence in the manuscript for clarification:
<u>Original:</u> "Of the 80 MV4;11 *MEN1* mutant cell lines, 37 outcompeted the non-edited WT control cells to a threshold of ≥ 3x enrichment (>60% *MEN1* mutant, <40% *MEN1* WT on day 9) with at least two menin inhibitors or ≥ 4.25x enrichment (>85% *MEN1* mutant, <15% *MEN1* WT on day 9) with one menin inhibitor (**Supplementary Fig. 2B+C**)."

<u>Revised:</u> "From the 40 highly enriching sgRNAs from the base editor screen, 32 outcompeted the non-edited WT cells in either MV4;11 A>G or C>T cell lines (and 5 sgRNAs outcompeted in both the A>G and C>T cell lines)..."

Reviewer #3, Comment 4. • The drug-sensitising variants such as MEN1 E368K seem interesting and similar drug-sensitising variants were described recently in another base editing paper (PMID:

39424923 – EGFR, PARP). How are these working in MEN1? Some mechanistic understanding could be useful to improve future MEN1 inhibitors.

This is an interesting question, and certainly several of our mutations seem to enhance menin inhibitor efficacy with menin inhibitor treatment:

- E368K:
    - >75x absolute IC_{50} shift for DSP-5336
    - 0.3x absolute IC_{50} shift for JNJ-6617 and SNDX-5613:
- V372A:
    - 20.5x absolute IC_{50} shift for KO-538
    - 0.7x absolute IC_{50} shift fpr DS-1594 and 0.8x for JNJ-6617
- A247T:
    - 6.3x, 5.9x, and 3.8x absolute IC_{50} shifts for JNJ-6617, SNDX-5613, and DSP-5336
    - 0.3x absolute IC_{50} shift for DS-1594 and SNDX-5613)
- D290 mutations:
    - See **Reviewer #1, Response #1** for more data on D290 mutations that we characterized *in vitro* and *in vivo* as part of this revision process, as well as Main Figure 3G
    - D290H:
        - 14x absolute IC_{50} shift of 14x for JNJ-6617
        - 0.5x absolute IC50 shift for DSP-5336

Our speculation is that some of these mutations could lead to better binding of menin inhibitors in the KMT2A binding pocket of menin, thus reducing the absolute IC_{50} given enhanced menin-menin inhibitor binding. This is difficult to definitively prove without extensive biochemical studies, but nevertheless we attempted to provide some mechanistic understanding towards the enhanced sensitivity by characterizing the E368K and V372A mutations more rigorously. We used two approaches: 1. Reduction of *MEIS1* expression as an orthogonal output of menin inhibitor efficacy, 2. TR-FRET binding assays.

We hypothesized that if the increased sensitivity seen with certain menin inhibitors for specific *MEN1* mutations is related to enhanced drug binding, *MEIS1* expression should be suppressed to a greater extent than wildtype control. We examined *MEIS1* expression after 72 hours of treatment in MV4;11 C>T *MEN1* WT cells line (control) and compared this to expression levels in the MV4;11 C>T cell line that makes a predominant E368K mutation and the A>G cell line that makes a predominant V372A mutation. For E368K, we evaluated DSP-5336 and SNDX-5613 (see above for absolute IC_{50} shifts). As expected, *MEIS1* expression was maintained despite DSP-5336 treatment in the presence of an E368K mutation. In contrast, with both SNDX-5613 10 nM and 50 nM, there was a greater reduction in *MEIS1* expression in the presence of an E368K mutation compared to wild-type. For V372A, we evaluated KO-539 and JNJ-6617 (see above for absolute IC_{50} shifts). As expected, *MEIS1* expression was maintained despite KO-539 treatment in the presence of a V372A mutation. In contrast, at JNJ-6617 10 nM there was a greater reduction in *MEIS1* expression in the presence of a V372A mutation compared to wild-type. A trend towards a difference was seen at JNJ-6617 50 nM, however at this dose *MEIS1* expression is so markedly suppressed in the wild-type setting that it was difficult to

outperform in the mutant setting. The data below is pooled from three independent experimental experiments and is now part of Supplementary Fig. 3D.

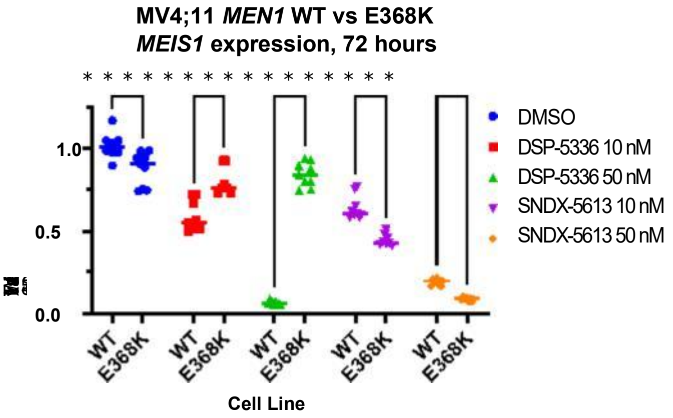

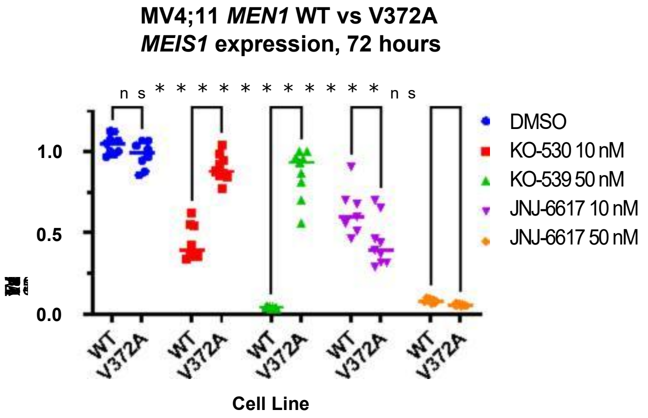

We have added the following language to the manuscript to describe these results: "*This increased sensitivity, i.e., lower absolute IC_{50}s, was also seen in competition assays: MEN1 E368K cells depleted faster than wild-type with JNJ-6617 and SNDX-5613, and MEN1 V372A cells depleted faster with DS-1594 and JNJ-6617 (**Supplementary Fig. 2B+C**). Consistent with this, MEIS1 expression was more strongly reduced in E368K mutants than wild-type cells after SNDX-5613 treatment (10 or 50 nM) and in V372A mutants after JNJ-6617 treatment (10 nM) (**Supplementary Fig. 2G**).*"

The other way we made mechanistic insights into the drug-sensitizing mutants E368K and V372A is with TR-FRET binding assays, which we have described above in **Reviewer #1, Comment 8**. We have included the performance of these novel, drug-sensitizing mutants alongside the previously characterized *MEN1* mutants. The K_i values appear similar or slightly decreased for SNDX-5613 in the E368K mutant and for JNJ-6617 in the V372A context. Definitive evidence of enhanced binding would require further biochemical analyses, which may be a focus of future work.

Reviewer #3, Comment 5. • Although some selected validation is performed in Fig 3, it will be important to summarise for the reader how many and which variants from the screen have been validated (in vitro, in vivo etc), and which have been NGS verified. This is important for the usefulness of the data clinically.

We thank the reviewer for this comment and agree that identifying the exact mutation(s) generated by each guide is critical for interpreting resistance. For the 37 cell lines in which we performed screening dose-response curves (Figure 3A; corresponding to those showing enrichment in the competition assays, Supplementary Fig. 2C), we conducted Sanger sequencing to determine the specific edits introduced. We also performed amplicon sequencing for 11 of the 12 cell lines used in confirmatory viability assays (Figure 3B), with the sole exception being a cell line carrying a near-homozygous Ala247Thr substitution resulting from a single base-pair edit without bystander mutations.

Accordingly, we have expanded and revised Supplementary Fig. 2F to include all 37 cell lines for which dose-response curves were generated, annotating the *MEN1* mutations introduced, displaying the corresponding Sanger sequencing chromatograms at each edited site, and, where applicable,

showing amplicon sequencing results confirmed by CRISPResso analysis. Representative examples from 5 of the 37 cell lines are shown below; the remaining 32 are presented in Supplementary Fig. 2F.

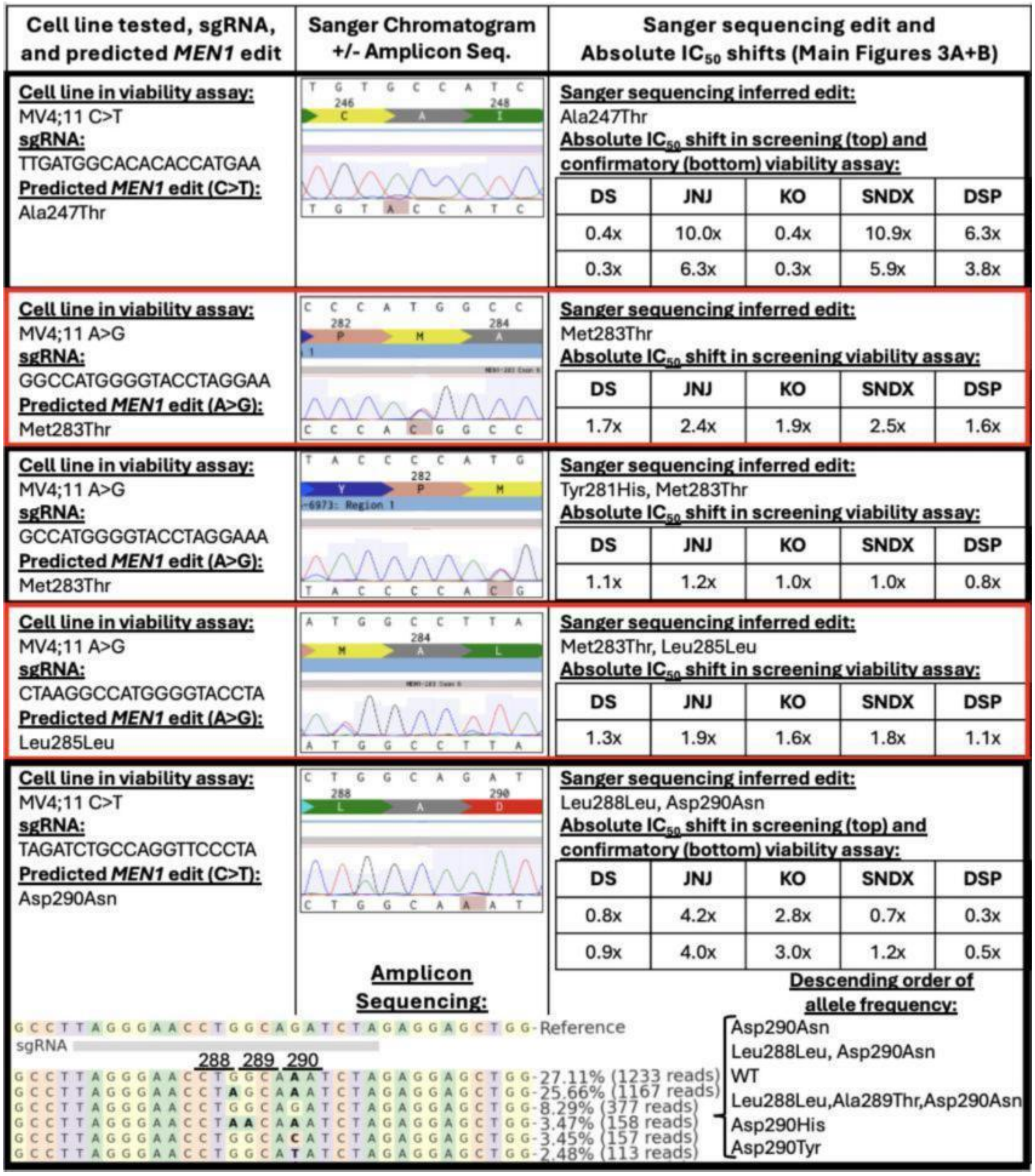

Based on this feedback, we have slightly changed the abstract to more specifically capture the pan-resistant and unique *MEN1* mutations that impact MIs in this study.

Original: Menin inhibitors have entered clinical trials for *KMT2A*-rearranged and *NPM1* mutant acute leukemias and are demonstrating promising activity. CRISPR-Cas9 base editor screening previously predicted several *MEN1* mutations that have arisen in patients receiving SNDX-5613 and confer menin inhibitor resistance. The extent to which *MEN1* mutations will impact each menin inhibitor is currently unknown. We leveraged advances in CRISPR-Cas9 base editing technology to predict the *MEN1* mutation profile that will impact five different menin inhibitors in clinical trials. We found key similarities (M327) and differences (C334, E368, V372) in the profile of *MEN1* mutations that affect each compound. The co-crystal structure of menin bound to each compound suggests resistance mechanisms related to how each menin inhibitor binds in the KMT2A binding pocket of menin. Our *in*

vitro and *in vivo* validation suggests that the *MEN1* mutations identified and validated with this approach are likely to arise and impact all menin inhibitors.

<u>Revised:</u> Menin inhibitors (MIs) have entered clinical trials for *KMT2A*-rearranged and *NPM1* mutant acute leukemias and are demonstrating promising activity. CRISPR base editor screening previously predicted several *MEN1* mutations that have arisen in patients receiving SNDX-5613 and confer MI resistance. The extent to which *MEN1* mutations will impact each MI is unknown. We leveraged advances in CRISPR base editing technology to screen for the *MEN1* mutations that will impact five different MIs in clinical trials. We found key similarities (M327I/V, G331D) and differences (C334R, E368K/V, V372A) in the profile of *MEN1* mutations that affect each MI. The co-crystal structure of menin bound to each MI suggests resistance mechanisms related to how each MI binds in the KMT2A binding pocket of menin. Orthogonal *in vitro* and *in vivo MEN1* mutation generation under therapeutic pressure suggests the *MEN1* mutations identified with CRISPR base editor screening are likely to arise and impact all MIs.

Reviewer #3, Comment 6. • The authors state that the degree to which... "these MEN1 mutations attenuates menin inhibitor activity cannot be inferred from the genetic screening data". This is contentious if the MAVE assay is a true readout of menin activity. Can the authors test this in 4B by plotting LFC vs Ki shift?

We thank the reviewer for this comment and have removed this sentence. We agree that a higher Log_2FC likely corresponds to a greater shift in absolute IC_{50}; however, without validating individual guides and confirming their specific effects on menin inhibitor activity, it is difficult to precisely link a given genetic perturbation to the resulting mutation and its impact on inhibitor efficacy.

As the reviewer suggested, below we have plotted the K_i for a given *MEN1* mutation along with the guide from the base editor screen that targeted the same residue and produced the most deleterious effect. Here the correlation between the Log_2FC in the screen for the most deleterious guide targeting the residue and the K_i for a mutation at a given residue is strong.

Binding (Ki) versus Log2FC

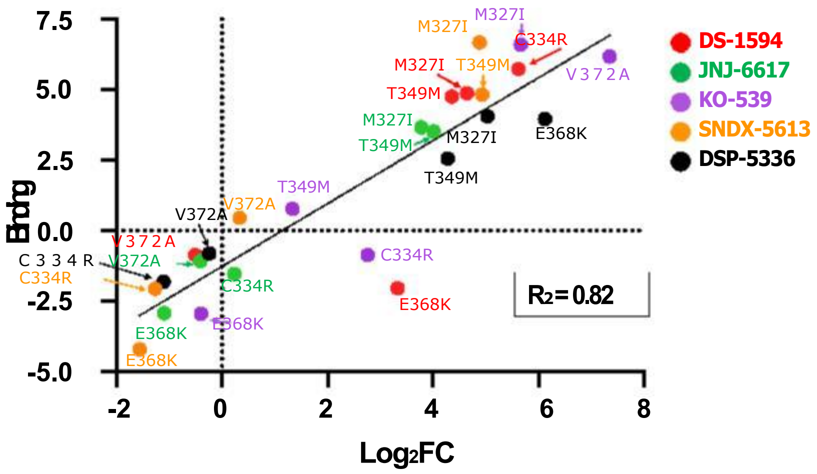

However, this analysis is biased because we selected the Log_2FC for the most deleterious guide targeting each residue for which we had corresponding binding shifts (Ki). When a more unbiased approach is taken and all guides targeting the given residue are considered, the correlation

diminishes substantially. For instance, four guides in the screen were predicted to edit Methionine 327, a residue recurrently mutated in patients treated with SNDX-5613.

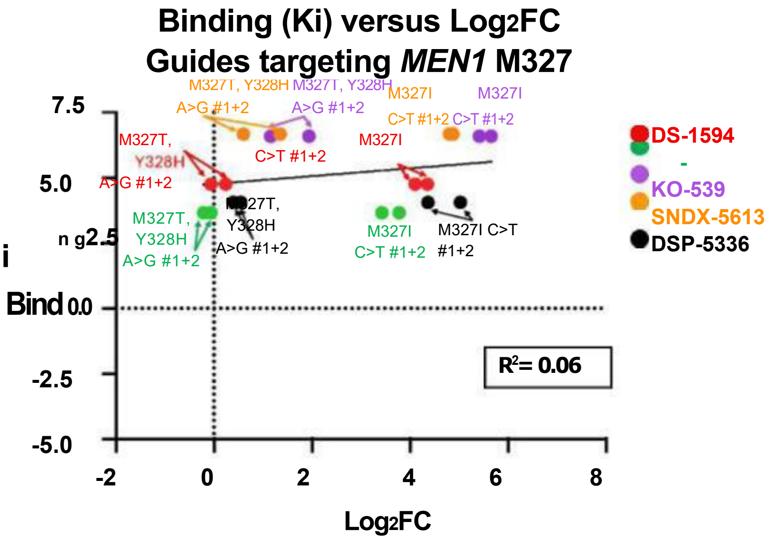

As seen above, the correlation here is very weak, and that is because there is minimal Log2FC for the guides targeting M327T, Y328H in the A>G cell line. We examined one of these two guides predicted to make an M327T, Y328H edit in the A>G line and the editing efficiency was quite low (below, left). Accordingly, there was minimal enrichment of this guide in 9-day competition assays. In contrast, a guide that made a near homozygous M327I edit in the C>T base editor line enriched rapidly over the course of 9 days and had high Log2FCs in the base editor screen.

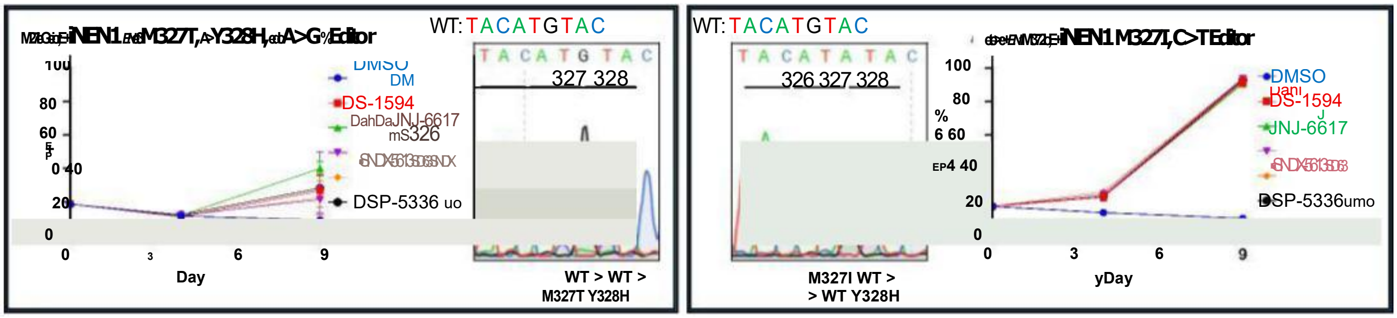

We appreciate the reviewer's point that strongly positive Log2FC values likely reflect greater attenuation of menin inhibitor binding to menin. However, confirming this relationship based on the screen alone is challenging without single-guide validation, given variability in editing efficiency among guides and uncertainty regarding the exact mutation generated by each.

%

+

Reviewer #3, Comment 7. • With the base editing dataset, can the authors now speculate on a combination of menin inhibitors that would be effective and prevent or slow drug resistance? Do the authors think that it is feasible to combine them in vivo or in patients? Would this be more effective

than high dose inhibitor? Some testing of menin combinations (and perhaps benchmarking to high dose of one) would be a valuable addition.

We agree with the reviewer that, in principle, combinations of menin inhibitors could prevent the outgrowth of i mutant resistant clones. However, with the experimental models currently available to us, this is difficult to test directly. Only JNJ-6617 and SNDX-5613 have publicly available murine pharmacokinetic data, and the cost and scale required to rigorously test whether combinations of

menin inhibitors reduce mutation emergence relative to monotherapy would be prohibitive (See Reviewer #1, Comment #1 for further explanation).

Nevertheless, we sought to address this question *in vitro* using two complementary approaches. The first approach involved treating menin inhibitor-resistant cell lines with combinations of menin inhibitors. We tested these combinations in MV4;11 C>T cells harboring an sgRNA targeting E368, which predominantly generated an E368K mutation (Supplementary Fig. 2F), MV4;11 A>G cells harboring an sgRNA targeting V372, which predominantly generated a V372A mutation (Supplementary Fig. 2F), and a wild-type control line. Eight-point dose-response curves were generated for each inhibitor individually and for the combination, with single-agent concentrations ranging from 1000 to 0.3 nM and combination concentrations ranging from 500 to 0.15 nM per inhibitor, corresponding to a total combined dose range of 1000 to 0.3 nM. In both mutant lines, the absolute IC_{50} was lower with combination treatment than with either single agent, although the magnitude of the IC_{50} shift did not exceed that observed for either inhibitor alone

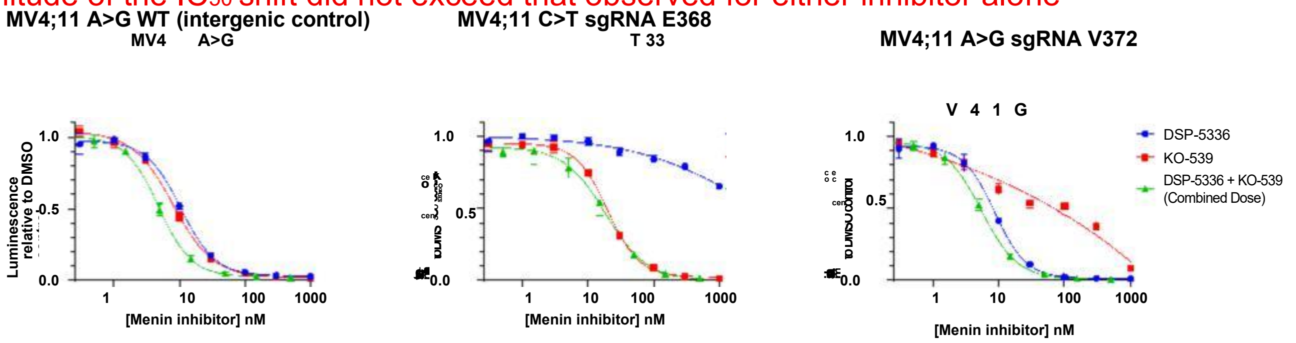

Absolute IC50 (nM)			Absolute IC50 (nM)			Absolute IC50 (nM)		
DSP-5336		KO-539 (Combined Dose	DSP-5336	KO-539c	Combined Dose	DSP-5336	KO-539c	Combined Dose
10.5 nM	8.8 nM	5.0 nM	>1000 nM	18.8 nM	15.5 nM	7.8 nM	78.8 nM	5.2 nM

			Absolute IC50 shift			Absolute IC50 shift		
				>95x	3.1x	0.7x	9.0x	1.0x

We also combined these cell lines to generate a mixed population consisting of one-third wild-type cells, one-third cells with sgRNA E368, and one-third cells with sgRNA V372. In this mixed population, the absolute IC_{50} shift was similar between KO-539 alone and the combination treatment; however, the combination achieved a lower overall viability relative to the DMSO control.

MV4;11 WT, sgRNA E368, sgRNA V372 combined

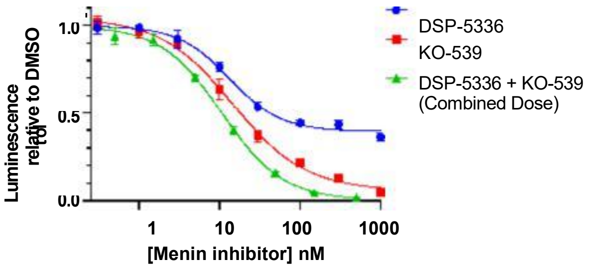

Absolute IC_{50} (nM)

DSP-5336	KO-539	Combined Dose
42.8 nM	18.3 nM	10.7 nM

Absolute IC_{50} shift

4.1x	2.1x	2.1x

Taken together, these results show that the combination of menin inhibitors applied to a polyclonal *MEN1* mutant pool could be helpful in treating each of the clones at the same time. Of note, many patients with *MEN1* mutations have more than one *MEN1* mutation identified (Perner et al, *Nature*, 2023). We have not included this approach in the revised manuscript.

The second approach to discern the effects of menin inhibitor combinations in overcoming menin inhibitor resistance was to turn back to our methods of spontaneous *MEN1* mutation generation through seeding large number of cells and exposing to 25 nM of menin inhibitor. See **Reviewer #2, Comment #1** above for further details. Briefly, we returned to our methods of seeding large numbers of cells to assess for outgrowth of resistant, *MEN1* mutant clones (Main Figure 5A+D). Across several experiments, we saw 4 *MEN1* mutant clones outgrow from 22 total replicates in JNJ-6617 25 nM treated cells, 2 *MEN1* mutant clones outgrow from 22 total replicates in DSP-5336 25 nM treated cells (in addition to 2 non-*MEN1* mutant clones), and finally no outgrowth in 12 total replicates of JNJ-6617 25 nM in combination with DSP-5336 25 nM. These combination experiments are now in **Supplementary Fig. 4A+B.**

Reviewer #3, Comment 8. • Could the authors comment on potential off-target resistance mechanisms in the Discussion i.e. not in MEN1? This seems a potential limitation of the study.

Thank you for this comment. Certainly, there are likely to be other forms of resistance to menin inhibitors, and we have added language to the discussion that these non-*MEN1* mutant forms of resistance are likely to impact all menin inhibitors by virtue of their common mechanism of action, and we have cited several papers that touch on these non-*MEN1* mutant forms of menin inhibitor resistance:

1. Zhou X, Zhang L, Aryal S, et al. Epigenetic regulation of noncanonical menin targets modulates menin inhibitor response in acute myeloid leukemia. *Blood*. 2024;144(19):2018-2032. doi:10.1182/blood.2023023644
    a. This paper describes how loss of PRC1.1 complex subunits can mediate resistance to menin inhibitors through epigenetic reactivation of noncanonical targets, such as MYC.
2. Soto-Feliciano YM, Sánchez-Rivera FJ, Perner F, et al. A Molecular Switch between Mammalian MLL Complexes Dictates Response to Menin-MLL Inhibition. *Cancer Discov*. 2023;13(1):146-169. doi:10.1158/2159-8290.CD-22-0416 a. This paper describes a molecular switch between MLL1 -Menin and MLL3/4-UTX chromatin-modifying complexes, whereby MLL1 -Menin disruption triggers MLL3/4-UTX recruitment to tumor suppressor gene promotors. The resultant activation of gene expression programs by MLL3/4-UTX can be therapeutically targeted with CDK4/6 inhibitors.
3. Gordon SJV, Perner F, MacPherson L, et al. Catalytic Inhibition of KAT6/KAT7 Enhances the Efficacy and Overcomes Primary and Acquired Resistance to Menin Inhibitors in MLL Leukemia. *Cancer Discov*. 2025;15(10):2117-2138. doi:10.1158/2159-8290.CD-24-1517 a. This paper describes how KAT7 is a targetable genetic dependency in models of non-*MEN1*-mutation driven resistance.

Future work from our laboratory and others will focus on these non-*MEN1* mutant forms of menin inhibitor resistance, but so far modeling this form of resistance pre-clinically *in vitro* and *in vivo* has been difficult, likely because there are many ways cells can adapt to the on-target activity of menin inhibitors. In support of this, in the initial report on the clinical activity of revumenib from the Phase 1 AUGMENT-101 trial, no co-mutation was clearly associated with response (Issa G et al., *Nature*, 2023, PMID: 36922593). Sequencing further patient samples will be imperative to discern these alternate mechanisms of resistance.

We have added the following language to the end of the discussion, followed by the three citations above: *"While this work focuses on MEN1 mutation-driven resistance, resistance mechanisms without MEN1 mutations are actively being investigated and warrant further characterization in preclinical models and patient samples to inform the rational design of combination therapies to overcome resistance."*

Minor points

Reviewer #3, Comment 9. • Analysis code should be on GitHub otherwise we cannot review this.

We have not generated analysis code for this paper. For the *MEN1* base editor screen, we have supplied **Supplementary Dataset 2** showing the annotation of the screen and results. We have further uploaded the CRISPR base editor screen to the GEO database: GSE294624. At that location, the raw read counts are available as well as the sequencing data. The source data pertaining to the screen in Fig. 2c also contains further raw results for individual replicates.

Reviewer #3, Comment 10. • Why are there gaps in the sequence of amino acids in Fig. 2c?

The x-axis is broken in Figure 2C to allow for better visualization of the menin amino acid residues where the greatest frequency of hits occurred, roughly from amino acids 136 to 372.

Reviewer #3, Comment 11. • Fig.4c-g is not very clear for the reader. Perhaps distil this information into a couple of structures or ensemble, highlighting key residues? Currently simply referred to as Fig4c-g without any signposting.

Reviewer #1 raised similar concerns regarding the general presentation of the X-ray data; please refer to **Reviewer #1, Comments 3-5**, for a detailed description of how we have improved both the submission and presentation of these data. In the same vein, we have revamped Main Figure 4C–G to display only four inhibitors (DSP-5336, KO-539, DS-1594, and JNJ-6617) for clarity, using ICM Chemist Pro (MolSoft, Inc.) to address Reviewer #3's concern. These structural representations are now organized as Main Figure 4C-F (see below). We believe that these revisions more clearly highlight the distinct binding modes of each compound. We have labeled all novel residues representing compound-specific liabilities for DSP-5336 (E368, A247), DS-1594 (C334), KO-539 (V372), and JNJ-6617 (D290, A247) on all structures. We have also displayed S160, M327, G331, and T349, which correspond to sites of *MEN1* mutations observed in patients receiving SNDX-5613.

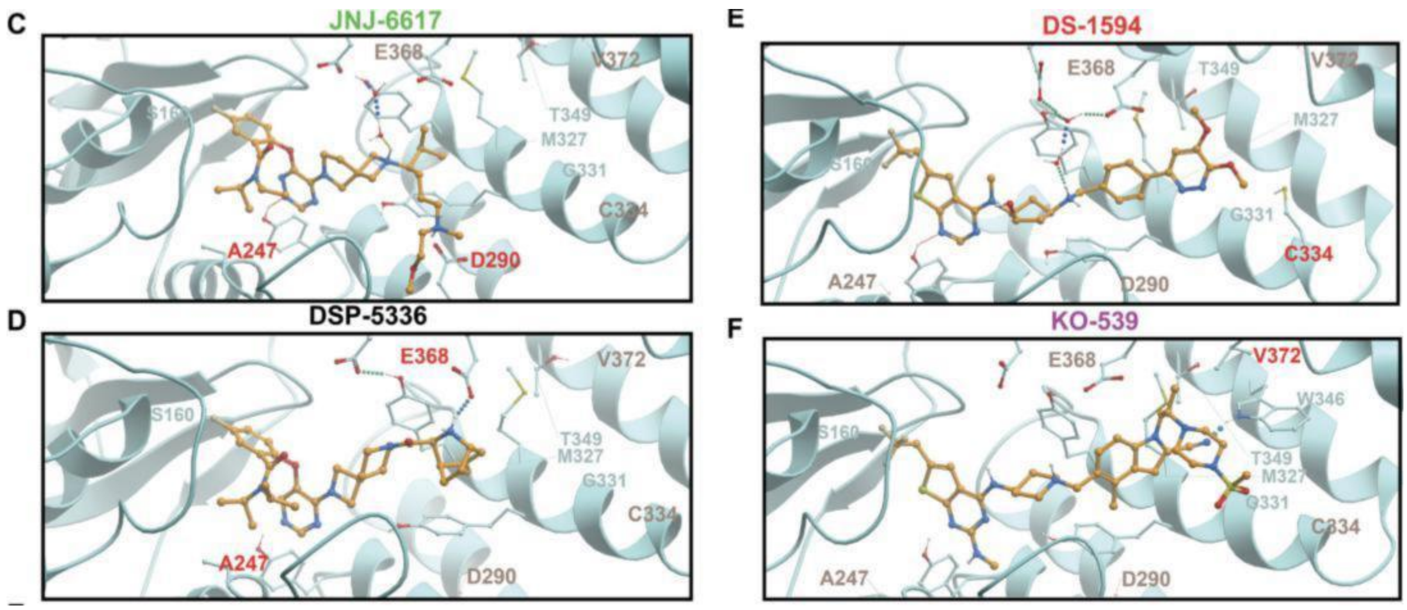

Reviewer #3, Comment 12. • I recommend the authors deposit their data on MAVEdatabase to aid data sharing.

We will aim to do this in the future, however, at the present time the MAVEdatabase does not hold base editor data, which was confirmed by our co-author John Doench. There are plans to allow upload of base editor screening data in the future, and when this is established, we will gladly upload our data.

We have uploaded the CRISPR base editing screen data under the following GEO accession code: GSE294624.

Reviewer #3, Comment 13. • The screens are referred to as CRISPR-Cas9 screens throughout. They are CRISPR base editing screens, not Cas9 screens (cut). This needs to be rephrased for clarity.

We thank the reviewer for this comment and have made this change throughout the manuscript.

Reviewer #3, Comment 14. • Can the authors speculate on why some mutations might appear spontaneously and in vivo, and others not? Is it a function of effect size, or something else?

We thank the reviewer for raising this question regarding why some mutations appear spontaneously *in vivo* whereas others do not. The base editing system can generate certain edits that are unlikely to occur spontaneously in vivo or in patients. For example, the base editing system generated a G331N mutation by editing the codon GGC to GAA. Because this change requires two nucleotide substitutions, it is unlikely to arise spontaneously, which likely explains why this mutation is not observed in vivo. With regard to spontaneous mutation emergence reported thus far in patients, we note that among the 20 mice receiving the most patient-relevant dose of SNDX-5613 (0.1% chow) shown in Main Figure 6A, we observed every *MEN1* mutation reported in patients to date, including S160T, M327I, M327V, G331D, G331R, and T349M. We additionally identified an M327T mutation,

which has not yet been reported in patients. In addition, in the new Main Figure 6B, we identified a D290V mutation arising during JNJ-6617 treatment, further expanding the spectrum of *MEN1* mutations observed without genetic editing. Finally, another consideration for why some mutations may not arise relates to their impact on absolute IC_{50}. In both patients and our PDX experiments, multiple *MEN1* mutant clones are often detected at the time of humane endpoint. Thus, some *MEN1* mutations may arise but subsequently be outcompeted by clones conferring greater resistance.

Reviewer #3, Comment 15. • Figure 6A is poorly presented. I suggest showing the data more carefully by segregating different doses of the drug, for example. What's happening in the WT cancer cells? Are there other mutations, not in MEN1 or just below detection?

We have added a new Main Figure 6C to better annotate the mutations that emerged in the mice in the PDX experiment in Main Figure 6A, as well as a new PDX experiment in Main Figure 6B, segregating by different doses of drug:

C *MEN1* mutations in Fig. 6A+B

	SNDX-5613 0.1% chow	SNDX-5613 0.3% chow	JNJ-6617 30 mg/kg	JNJ-6617 100 mg/kg
WT only	3 mice	10 mice	0 mice	3 mice
S160T	2	0	0	0
D290V	0	0	1	0
M327I	1	0	0	0
M327T	1	0	0	0
M327V	2	2	0	0
G331D	0	1	2	1
G331R	1	0	0	0
T349M	11	0	2	0

We have also provided, in new Supplementary Fig. 5C, the Sanger sequencing chromatograms for all mice in Main Figure 6A+B that developed *MEN1* mutations.

We used Sanger sequencing to find these mutations and we assume that there are some *MEN1* mutants below the detection threshold of detection, as suggested by the reviewer.

We are very curious as well as to what other factors could be driving resistance in mice that had detectable leukemia at the time of death without *MEN1* mutations. This is a line of further inquiry beyond the scope of this paper that we hope to explore in the future.

Reviewer #4 (Remarks to the Author):

The study by Bourgeois et al and the Armstrong group is an in-depth analysis of acquired MEN1 mutations in the context of different MEN1 inhibitors under clinical evaluation. This is an extension from their previous study in Nature evaluating clinical samples from the AUGMENT trial and a prior base editing screening approach that utilized base editors capable of only C>T nucleotide transitions and SpCas9 75 nickases that required an NGG protospacer adjacent motif (PAM) site. This paper now uses MV4;11 cell line with C>T and A>G editors along with 5 reversible, non-covalent menin inhibitors that had entered clinical trials: DS-1594 (emilumenib, Daiichi Sankyo), JNJ-75276617 90 (bleximenib, Janssen Pharmaceuticals, referred to as JNJ-6617 hereafter), KO-539 (ziftomenib, Kura

91 Oncology), SNDX-5613 (revumenib, Syndax Pharmaceuticals), and DSP-5336 (enzomenib, Sumitomo 92 Pharma). In addition to the identification of the spectrum of acquired resistance mutation at high resolution using this updated CRISPR base editing strategy, they support their findings with co-crystal structures of each inhibitor interacting with menin and identified similarities and differences between the binding of each inhibitor. They extensively cross-validated the identified mutations with the variety of MEN1 inhibitors and then generated 80 mutant MV4;11 cell lines for testing. Spontaneous development of these mutations was tested in a different KMT2Ar cell line and with PDX samples. They then nicely the impact of different concentrations of SDNX using supplemented chow to induce mutations and determined that it was significantly less likely at high concentrations.

Reviewer #4, Comment 1. Overall, this is a rigorous study of high translational impact. It would be helpful to the field to understand if the levels of SNDX achieved in their high-dose chow are achievable in patients. And does the degree of MEN1 inhibition at high dose result in undesirable clinical side effects or functional defects in normal HSPCs? Beyond this, I have no other concerns about this study, which will be highly impactful to the field.

Thank you for the feedback on our manuscript. Indeed, the clinical relevance of the high doses of SNDX-5613 chow is an important consideration. We address this point extensively in "**Reviewer #1, Comment 2**". The dose limiting toxicity of SNDX-5613 is QTc prolongation, so it would be difficult to increase the dose of SDNX-5613 further in patients. However, it should be noted, when looking at the distribution in the range of plasma concentrations in patients and comparing it to mice, many patients did attain comparable dose levels to that observed in mice receiving 0.3% SNDX-5613 chow ("**Reviewer #1, Comment 2**"). QTc prolongation appears to be a side effect that is unique to SNDX-5613, and other menin inhibitors have not been impacted by this toxicity. This suggests that other menin inhibitors could attain higher plasma concentrations that might suppress the emergence of *MEN1* mutations, provided that on-target toxicities such as differentiation syndrome are appropriately managed.

The effect on HSPCs is very interesting and beyond the scope of this manuscript. Patients appear to develop some degree of myelosuppression on menin inhibitor therapy, but the long-term effects have not yet been described. Hopefully this data will emerge, particularly as swimmer's plots from various menin inhibitor trials indicate that some patients have been on menin inhibitor therapy for more than a year.

Reviewer #5 (Remarks to the Author): co-reviewed with Reviewer #4

I co-reviewed this manuscript with one of the reviewers who provided the listed reports. This is part of the Nature Communications initiative to facilitate training in peer review and to provide appropriate recognition for Early Career Researchers who co-review manuscripts.

Thank you to all the referees for another review of our manuscript, which we believe have strengthened the manuscript and improved clarity. Our responses are below.

Reviewer #1 (Remarks to the Author):

The revised manuscript by Bourgeois et al. addresses most of the points initially raised through additional experiments and further data analysis. Importantly, data has been uploaded to the appropriate databases (PDB, GEO), making them available to the scientific community. There are, however, several minor points that still need to be addressed:

- The rationale beyond using KMT2B-derived peptide and a different assay (TR-FRET) for characterization of the activity of inhibitors with additional menin mutants included in the revised manuscript (R334, K368, A372) instead of KMT2A-derived peptide utilized in FP competition experiments with mutant initially included (I327, M349, R331) is not clear. Comparison of Ki values for all menin inhibitors with WT menin obtained from both assays indicates ~2-orders of magnitude (~100x) difference between values from FP (with KMT2A 4-43 probe, Supplementary Figure 3A top) versus TR-FRET (with KMT2B 15-48 probe, Supplementary Figure 3A bottom), which might introduce some inconsistency. To compensate for this discrepancy, it would be important to provide a Table with: (1) IC50 values for WT and every mutant with each compound (specifying which method was used for IC50 measurements), and even more importantly (2) provide the fold difference in IC50 values for each mutant compared with WT menin for every compound.

We thank the reviewer for their careful attention to the binding data. We acknowledge that there are limitations associated with using KMT2A-derived and KMT2B-derived peptide probes to assess different menin mutants. Importantly, wild-type menin and menin mutants in this manuscript were characterized using TR-FRET assays; FP competition experiments were not used. The use of different peptide probes reflects that these experiments were performed in two different laboratories. M327I, G331R, and T349M experiments were led by Syndax Pharmaceuticals, whereas C334R, E368K, and V372A experiments were performed in the laboratory of Eric Fischer. Prior work from our group demonstrated that KMT2A and KMT2B peptides are similarly affected by menin mutations (Perner F, Stein E et al., *Nature* 2023; PMID: 36922589). The KMT2B peptide was used in the Fischer laboratory because it produced more robust and reproducible measurements in this assay format.

We appreciate the reviewer's suggestion to provide a table summarizing IC_{50} values and fold changes. We have added a Supplementary Table 2 that displays this data, which is shown below:

Supplementary Table 2: IC_{50} values and fold-change relative to wild-type menin for inhibitors tested against wild-type and mutant menin in TR-FRET assays

Raw IC50 values								
	TR-FRET FITC-KMT2A-4-43 probe				TR-FRET FITC-KMT2B-15-48 probe			
Compound	Wild-type	M327I	T349M	G331R	Wild-type	C334R	E368K	V372A
DS-1594	1.7	74.0	120.6	40.2	8.5	>468.0	3.1	5.5
JNJ-6617	0.5	8.3	8.6	0.4	8.5	4.3	1.9	5.0
KO-539	1.6	292.1	6.9	1.2	40.9	29.4	7.6	>862.4
SNDX-5613	6.7	908.9	266.2	41.5	26.4	8.6	2.4	40.6
DSP-5336	7.7	202.6	81.5	4.2	33.4	12.7	>619.9	21.8

IC50 fold change: mutant/wild-type								
	TR-FRET FITC-KMT2A-4-43 probe				TR-FRET FITC-KMT2B-15-48 probe			
Compound	Wild-type	M327I	T349M	G331R	Wild-type	C334R	E368K	V372A
DS-1594	1.0	42.7	69.6	23.2	1.0	>55.3	0.4	0.6
JNJ-6617	1.0	18.2	18.9	1.0	1.0	0.5	0.2	0.6
KO-539	1.0	183.2	4.3	0.7	1.0	0.7	0.2	>21.0
SNDX-5613	1.0	135.1	39.6	6.2	1.0	0.3	0.1	1.5
DSP-5336	1.0	26.3	10.6	0.5	1.0	0.4	>18.5	0.7

IC50 values in nM
IC50 shift is mutant / wild-type
In KMT2B experiments, ">" means that at least one experimental replicate had an IC50 greater than the assay limit, so the experimental replicate was capped at the highest dose utilized (1000 nM)

Number of experimental replicates, each with two technical replicates								
	TR-FRET FITC-KMT2A-4-43 probe				TR-FRET FITC-KMT2B-15-48 probe			
Compound	Wild-type	M327I	T349M	G331R	Wild-type	C334R	E368K	V372A
DS-1594	8	6	6	6	3	3	3	3
JNJ-6617	10	6	6	6	3	3	3	3
KO-539	11	7	7	6	3	3	3	3
SNDX-5613	155	7	7	6	3	3	3	3
DSP-5336	9	85	85	87	3	3	3	3

In addition, we have included a new Supplementary Figure 3B in which the raw IC50 values and fold changes are displayed together as a dot plot.

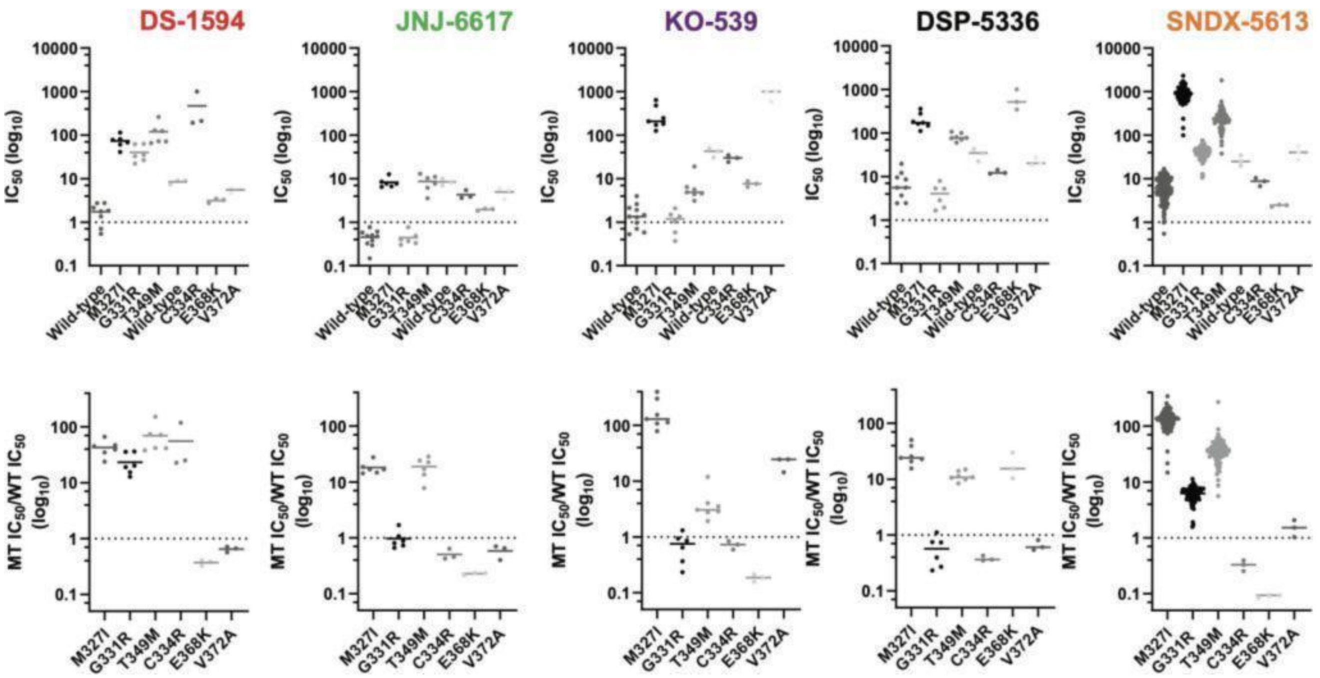

We have further revised our data given Reviewer 1's keen observation regarding the large differences in K_i values between the KMT2A and KMT2B peptides. This prompted us to re-review our data and we identified an error in the calculation used to derive K_i values from our SPR measurements. Correcting this calculation results in K_i values approximately one order of magnitude higher than previously reported. Below is an updated Supplementary Figure 3C that showing the K_i values for each probe for each menin mutation (x axes) for each menin inhibitor (stacked in columns).

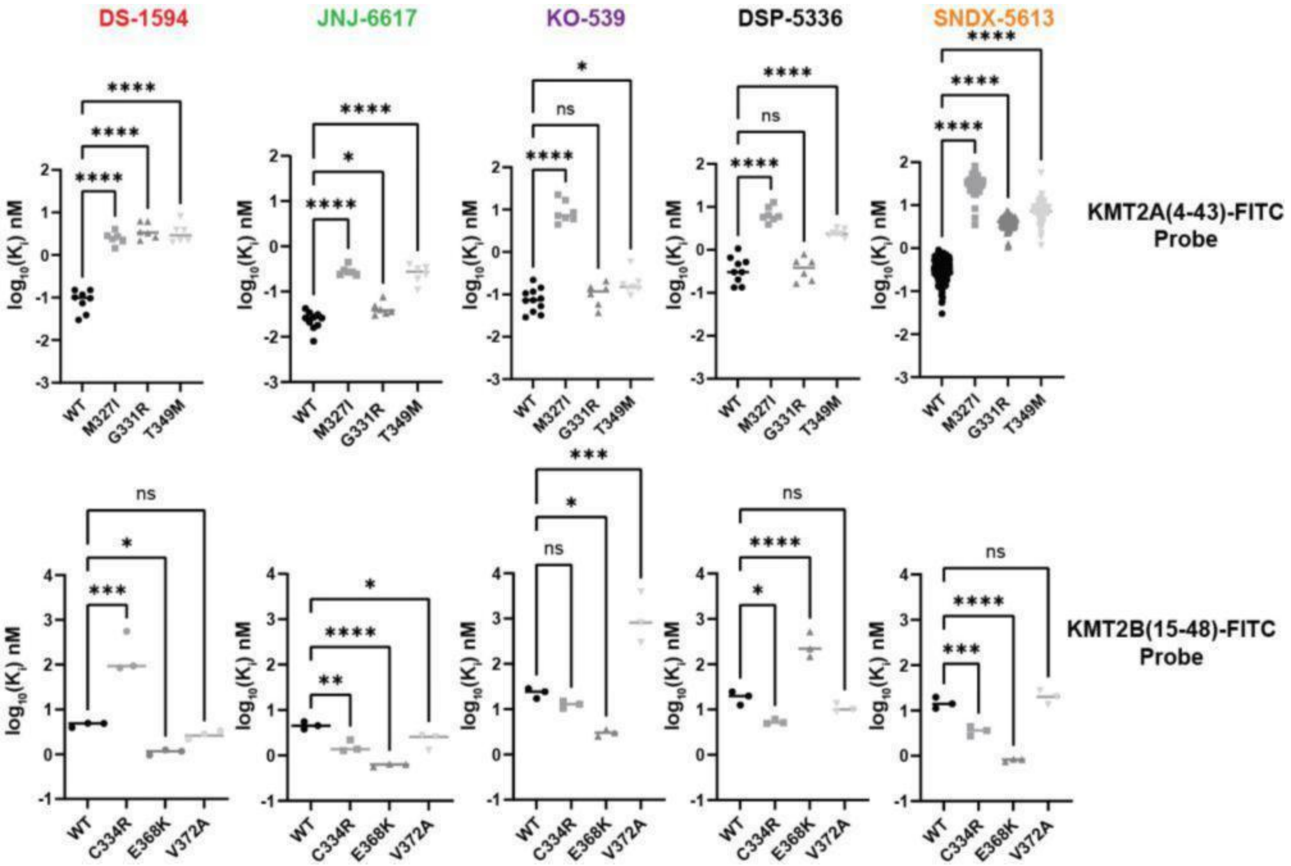

As shown, we still do see magnitude differences in K_i between the two assays. The remaining differences in absolute K_i values between the KMT2B TR-FRET and KMT2A SPR assays likely reflect differences in assay format, detection conditions, and dynamic range rather than true differences in inhibitor binding affinity. Importantly, the rank ordering of inhibitor potency across the different *MEN1* mutants remains consistent with the cellular data.

In summary, we believe both the correction of K_i and inclusion of IC_{50} analysis strengthens the presentation of the data, helping readers more accurately interpret the biochemical data.

- The IC50 values are missing for menin WT in the Supplemental Figure 3B (first graph on the top) – only "compound names: nM" are shown, but the actual values are missing. These values (collected from FP assay) need to be added as they are important for comparison with the menin-WT replicates 1-3 measured using TR-FRET method.

We thank the reviewer for noting this omission. The IC_{50} values for wild-type menin derived from the TR-FRET assay have now been added to Supplementary Figure 3A. During revision, we also corrected several minor issues in the figure, including unit labeling (μM vs nM) and adjustments to the y-axis scaling to ensure that all data points are visible. To improve the clarity of the data and more easily allow comparisons, we now show a representative curve and the corresponding IC_{50}s. The IC_{50} displayed in the legend represents the average IC_{50} across all experimental replicates. For the KMT2A-FITC probe assays, IC_{50} values were derived from at least six independent experiments, each performed with technical duplicates. For the KMT2B-FITC probe assays, IC_{50} values were derived from three independent experiments with technical duplicates. All experimental replicates are provided in the Source Data.

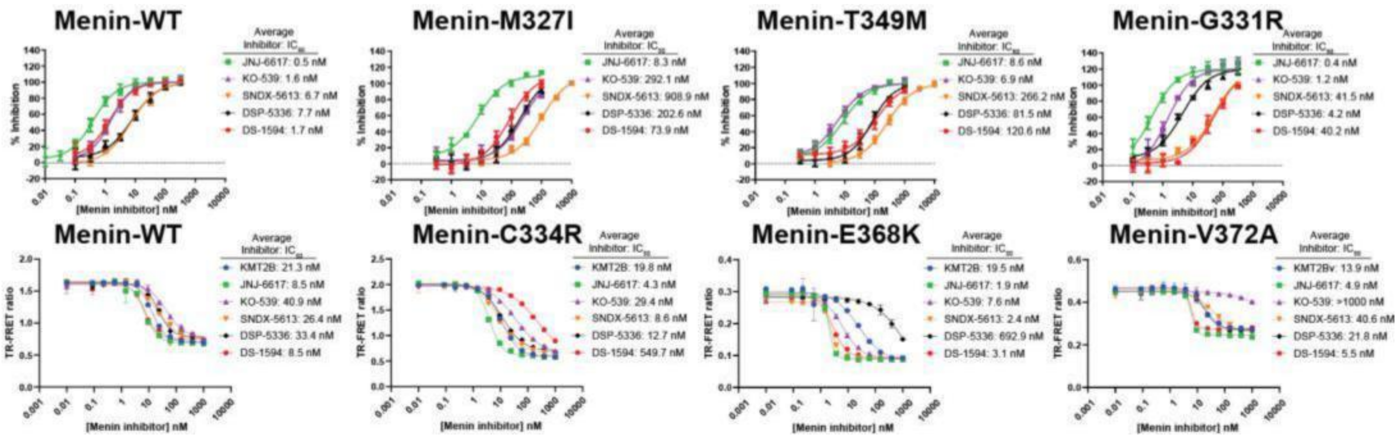

- The crystal structures presented at the Main Figure 4C-F include some of the hydrogen atoms on the protein, ligands or water molecules. Based on the resolution of these structures (>1.5A), the atomic coordinates for these hydrogen atoms are unlikely to be derived from the crystal structures (experimental data) and should be removed from these figures with only heavy atoms shown.

We thank the reviewer for noting this issue. Hydrogen atoms have now been removed from these structural images so that only heavy atoms are displayed. We have also included the heavy atoms of the side chains for residues S160, D290, V372, and C334 to provide consistent structural reference points across Figure 4C-F.

The updated images are shown below.

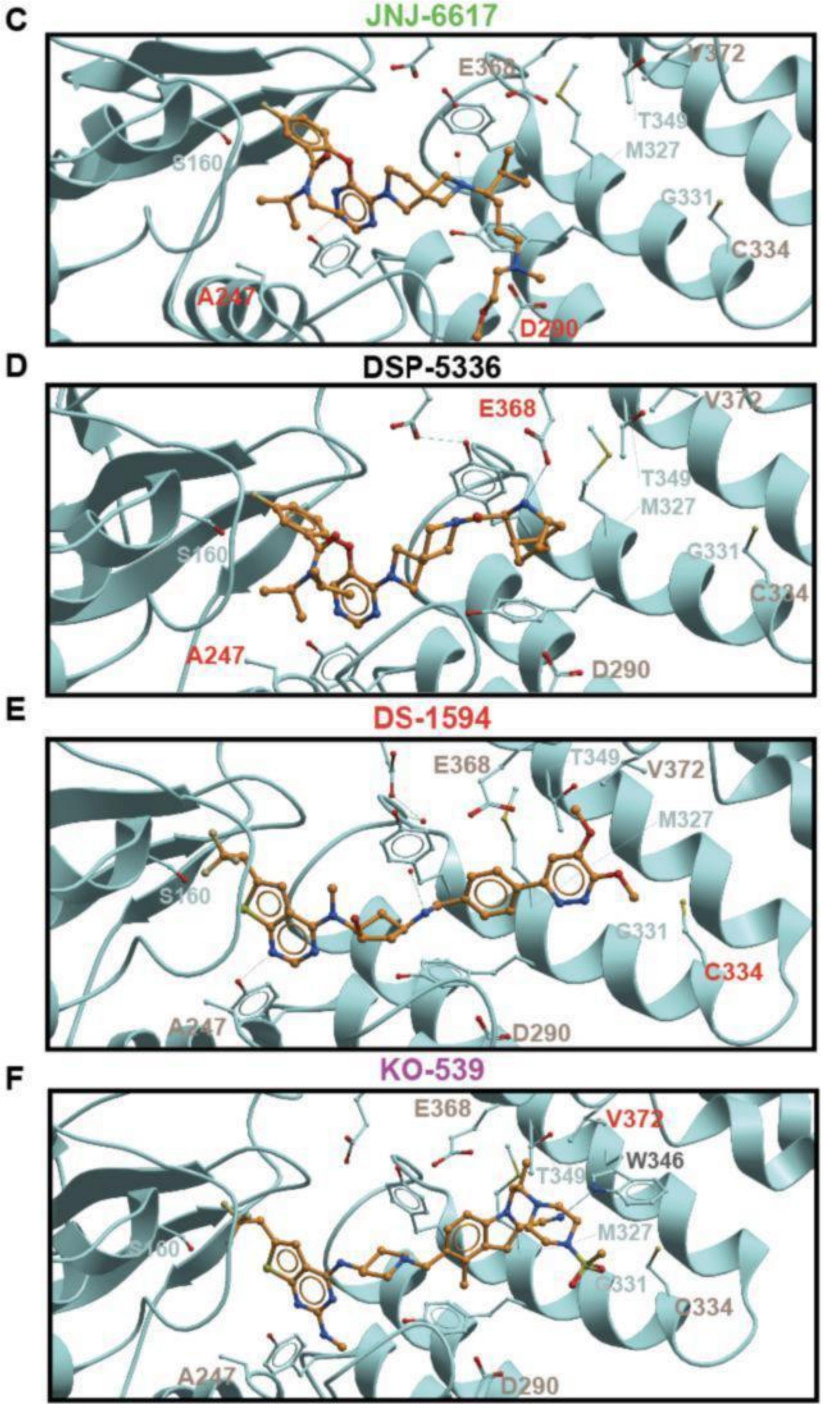

- The title of panel in Main Fig.1C “Menin-KMT2A peptide binding” is misdealing as this figure shows activity (titration curves from FP and IC50) for inhibitors

We thank the reviewer for noting this issue. The panel title has been revised to “Menin inhibitor binding assay”. Of note, this experiment reflects TR-FRET data, which was the approach used in this manuscript.

- Right panel of Supplementary Figure 2 is labeled “MV4;11 MEN1 WT vs sgRNA K367K, E368K”, which seems incorrect. Is this a single mutant or double mutant cells line (K367K seems incorrect/ not needed)?

We thank the reviewer for noting this confusing label. The designation “sgRNA K367K, E368K” was intended to indicate that the guide RNA was predicted to generate a synonymous K367K mutation and a missense E368K mutation. Amplicon sequencing (Main Fig. 3D and Supplementary Fig. 2F) confirmed that approximately 75% of alleles contain both the synonymous K367K change and the E368K mutation. To avoid confusion, the figure label has been revised to “sgRNA K367, E368”, consistent with the labeling used in Main Fig. 3D.

- In the revised methods section (KMT2A/B-menin displacement assay), the KD values used for calculation of Ki were measured by SPR – as stated in the manuscript: "KD values were determined for menin proteins using surface plasmon resonance (data not shown)" – sentence added to the revised manuscript. Since the authors use Ki for comparison of the fold difference between menin WT and mutants for each inhibitor, it could be worth including the SPR data in the supplement (especially since the data already exists). Alternatively, IC50 values could be used instead of Ki to calculate fold difference for mutants vs. WT for each compound.

We thank the reviewer for highlighting the reference to the SPR data. In this revision, we now include the SPR data summarized in a Supplementary Table and in Supplementary Datasets. During revision, we recognized that the estimation of K_D values requires additional clarification. SPR experiments were used to determine the K_D of the KMT2A peptide for wild-type menin.

The SPR measurements are now provided as Supplementary Table 3. The underlying data are provided in Supplementary Dataset 4.

Supplementary Table 3. Analysis of the binding kinetics/affinity of the FITC-KMT2A-4-43 peptide to human menin using the Cytiva T200 SPR system.[a]

PARAMETER	580 RU HIS-Menin	1250 RU HIS-Menin
k_{on}	2.8×10^6 M^{-1} s^{-1}	3.9×10^6 M^{-1} s^{-1}
k_{off}	4.9×10^{-4} s^{-1}	7.9×10^{-4} S^{-1}
K_D	0.17 nM	0.20 nM
R_{max}	37 RU	90 RU

[a] Data represent the mean of duplicate experiments. The k_{on}, $k_{o\%}$ and K_d values were generated by the Cytiva machine software.

We then used off-rate experiments to estimate relative differences between wild-type and mutant menin proteins, which we have included as Supplementary Table 4. The underlying data are provided in Supplementary Dataset 5.

Supplementary Table 4. Off-rate kinetics of FITC–KMT2A(4–43) dissociation from WT and mutant menin measured by HTRF and parameters used for IC50-to-Ki conversion

	WT		M327I		G331R		T349M	
	koff, min-1	t1/2, min	koff, min-1	t1/2, min	koff, min-1	t1/2, min	koff, min-1	t1/2, min
Replicate 1	0.092	7.5	0.054	12.8	0.169	4.1	0.061	11.4
Replicate 2	0.091	7.6	0.059	11.8	0.148	4.7	0.045	15.3
Replicate 3	0.085	8.2	0.063	10.9	0.171	4.1	0.042	16.4
Replicate 4	0.095	7.3	0.055	12.6	0.199	3.5	0.056	12.4
Mean	0.091	7.663	0.058	12.041	0.172	4.079	0.051	13.871
SD	0.004	0.360	0.004	0.851	0.021	0.490	0.009	2.358
Relative Kd (koff ratio)		1.00		0.64		1.90		0.56

Basis for adjusting IC50 to Ki conversion

	Relative Kd (koff ratio)	Kd (SPR, average)	Kd adjstment based on the off-rate	Kd
WT	1	185 pM	1x	185 pM
M327I	0.64		0.64x	117 pM
G331R	1.9		1.90x	352 pM
T349M	0.56		0.56x	102 pM

Ki = IC50 / (1 + [FITC-KMT2A(4-43)] / KD)

- Can the authors define "L" in the Ki equation (methods: KMT2A/B-menin displacement assay section).

We thank the reviewer for catching this. In this equation, L refers to the concentration of the labeled ligand, which in this assay is the FITC-KMT2A(4-43) peptide. The Methods section has been updated to clarify this point and now reads:

"Finally, K_i values were calculated based on the following equation: $K_i = IC_{50} / (1 + [\text{FITC-KMT2A(4-43)}] / K_D)$."

Reviewer #2 (Remarks to the Author):

Manuscript by Bourgeois et al describes comprehensive analysis of mutations arising in MEN1 upon exposure to various menin inhibitors. This is an important and timely research. Authors addressed all my comments and significantly improved the manuscript. I have couple minor points to address:

Comment 1.
Finding that more potent menin inhibitor reduces rate of MEN1 mutations and the outgrowth of resistant leukemia clones is one of the most consequential conclusions from this manuscript. Authors strengthened this finding in the revised manuscript by including new in vivo data showing that increasing the dose of different menin inhibitor such as JNJ-6617 effectively suppresses the development of MEN1 mutations.
Additional new results examine the effect of combination of two menin inhibitors (JNJ-6617 and DSP-5336). Although these experiments are in cell culture, they strengthen the overall conclusions regarding emergence of MEN1 mutations. A limitation of this approach is that the combination was tested at doubled total inhibitor concentration (25 nM DSP-5336 plus 25 nM JNJ-6617) compared with single-agent concentrations (25 nM), which likely contributes to the absence of resistant clones. This point should be acknowledged in the text.
Minor point: concentration of DSP-5336 is missing in Supplementary Fig 4 legend:
"In these two independent experiments (A+B), 12.5 million MOLM13 cells were seeded per replicate, with 5 flasks per experiment treated with JNJ-6617 25 nM, 5 flasks per experiment treated with DSP-5336 25 nM, and 6 flasks per experiment treated with both JNJ-6617 25 nM and DSP-5336 nM."

We thank the reviewer for this thoughtful comment. We agree that testing 25 nM of each compound results in a total menin inhibitor exposure of 50 nM, which may contribute to the suppression of resistant clones observed in the combination arm. We have added the following statement to the manuscript: "*However, it should be noted that the suppression of resistant clones observed with 25 nM of each compound may reflect the higher total menin inhibitor exposure, 50 nM combined, rather than a true benefit from combining two menin inhibitors with different MEN1 mutation resistance profiles.*"

Comment 4.
The authors' response regarding the possibility that MEN1 mutations may be pre-existing is very relevant. It would be worth mentioning in the text that several MEN1 mutant clones were detected prior to day 28 of treatment, suggesting the possibility that these mutations were pre-existing.

We thank the reviewer for this important point. We agree that the detection of resistant clones as early as Day 21 suggests that *MEN1* mutations either arose shortly after initiation of menin inhibitor therapy or were present at very low levels prior to treatment. We have added a sentence to the manuscript reflecting this possibility. *"The early outgrowth of these MEN1 resistant clones suggests that the mutations either arose shortly after initiation of menin inhibitor therapy or were pre-existing at very low levels prior to treatment."*

Minor point: Figure 6 legend refers to Supplementary Fig 4C but there is no such figure, please correct that.

Thank you for identifying this error. The correct reference is Supplementary Fig. 5E, and this has been corrected in the manuscript.

Final question to the authors – were any double mutations in MEN1 observed in these screens or in vivo?

This is an important question. In the initial clinical report (Perner F, Stein E et al., *Nature* 2023; PMID: 36922589), multiple *MEN1* mutations were often detected in individual patients. While some of these could theoretically represent compound mutations within the same clone, the distinct variant allele frequency dynamics observed in that study suggested that many mutations arose in separate clones.

In our PDX experiments, we occasionally detected more than one *MEN1* mutation within a single mouse (Supplementary Fig. 5C+D). However, in all such cases, we did not observe two distinct *MEN1* mutations each exhibiting a clear heterozygous peak by Sanger sequencing, arguing against their co-occurrence within the same cell population.

In the CRISPR base editor system, bystander edits within the editing window can generate multiple nucleotide substitutions from a single guide (Supplementary Fig. 2F). Nevertheless, the screen identified recurrent resistance residues also observed clinically (SNDX-5613), *in vivo* (SNDX-5613 and JNJ-6617), and *in vitro* (JNJ-6617 and DSP-5336).

Reviewer #3 (Remarks to the Author):

The authors have significantly improved the clarity and reporting in the revised manuscript. I particularly value the additional work on combining MEN1 inhibitors.
Relating to my points on the screen analysis:

- The depletion of intergenic control guides suggests there has been some bottlenecking and therefore coverage of the library in cell culture may not be sufficient. This is leading to noise in the L2FC vlaues and may be a source of false positives. This is an important point as the authors make claims on drug-sensitizing variants.

We thank the reviewer for this comment regarding potential bottlenecking. To enrich for sgRNAs generating *MEN1* mutations that confer drug resistance, we applied drug concentrations expected to deplete the majority of non-resistant cells. We maintained ~2000x library coverage throughout the screen, which is within accepted standards for positive selection CRISPR screens. While this design is not optimized for dropout analyses, our conclusions are restricted to positively selected, resistance-conferring variants, for which replicate concordance was high (see below).

We agree that the screen design does not permit robust inference of drug-sensitizing mutations. Statements regarding drug sensitization are based on orthogonal validation experiments, including absolute IC_{50} shifts (Figure 3A+B) and suppression of *MEIS1* expression by quantitative PCR (Supplementary Fig. 3C).

- Screens analyzed using MAGeCK RRA can generate FDR and pvalues for specific gRNAs when used in guide mode (not gene aggregated PMID: 25476604), which for gene editing screens, normally gives significant FDR and p-values for individual gRNAs (e.g. PMID: 35165384, PMID: 40845844, PMID: 38134886 – base, CRISPR and prime screens). In their response, the authors cite another base editing screen as an example to justify only z-scores (PMID: 39424923), but this study provides MAGeCK pvalues and FDRs. I would include these statistics to ensure the reader is aware of technical variability.

It will be important to see L2FC correlation plots of replicates to understand variation in their data leading to the lack of significant gRNA hits.
The authors have addressed my other comments.

Thank you for this comment. We agree that readers should be aware of potential technical variability inherent to pooled screening approaches and the risk of drawing overly broad conclusions from screening data alone. To address this, we performed extensive orthogonal validation of guides that scored in the screen. Each guide was tested individually to confirm resistance phenotypes in competition assays (Supplementary Fig. 2C) and dose-response experiments (Figures 3A+B), and the precise edits generated by each guide were characterized (Supplementary Fig. 2F). These validation experiments ensure that our conclusions are based on reproducible, guide-specific effects rather than screening noise.

To further strengthen the analysis and increase transparency, we have added extra data to “Supplementary Dataset 2: Base Editor Screen Annotation and Results”. First, in addition to the mean Log2 Fold Change of three technical replicates for each guide that we previously reported, we now have columns for each individual replicate.

We further generated sgRNA-level Log2 fold-change replicate concordance plots for each screen and report Pearson correlation coefficients (r) for each comparison. We have made these data new Supplementary Fig. F+G.

Supplementary Figure 1F:

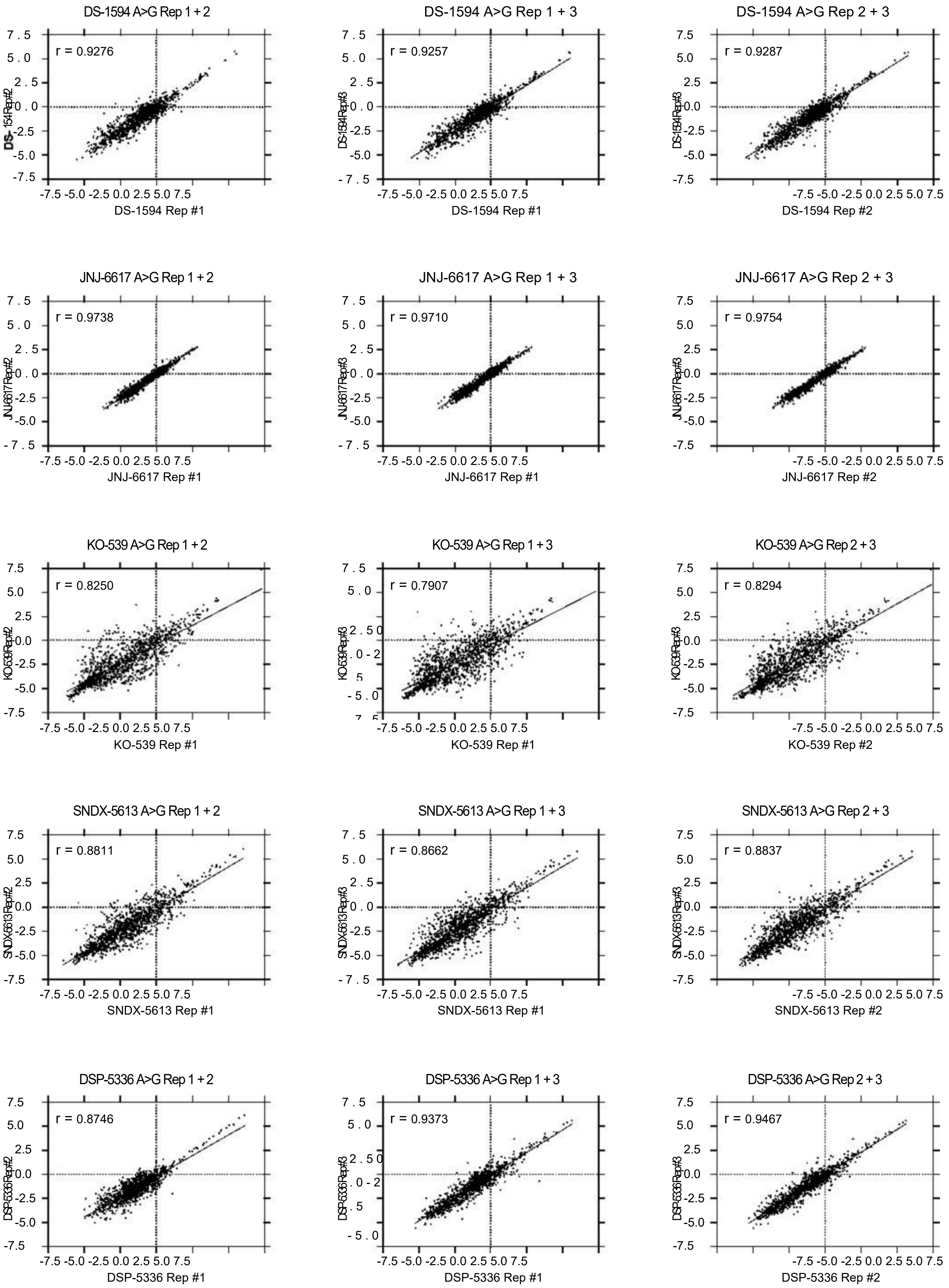

Supplementary Figure 1G:

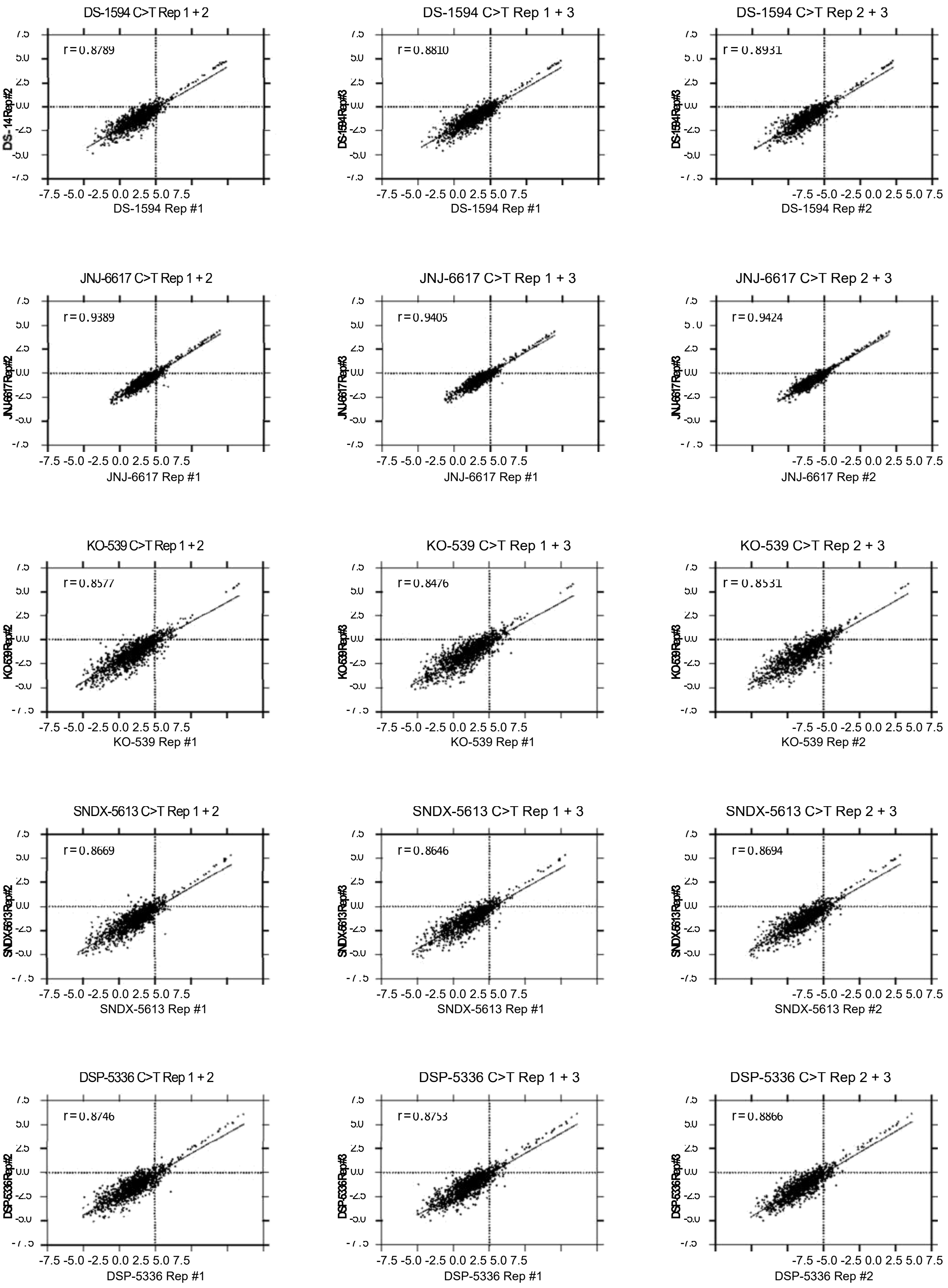

We have added the following text: "*Replicate concordance was high, with an average Pearson correlation coefficient of 0.90 across replicate pairs, and all pairwise correlations ≥ 0.79 (Supplementary Fig. 1F+G).*"

The lowest correlation was observed in the KO-539-treated MV4;11 A>G condition, which exhibited the strongest selective pressure (discussed in prior response to referees). In this condition, 23 sgRNAs demonstrated mean log2 fold-change ≥ +2.0. When analysis was restricted to these enriched guides, replicate concordance was much higher (see below). This suggests that it is the depleting sgRNAs in the presence of strong drug pressure that are driving variance, as compared to the highly enriching guides.

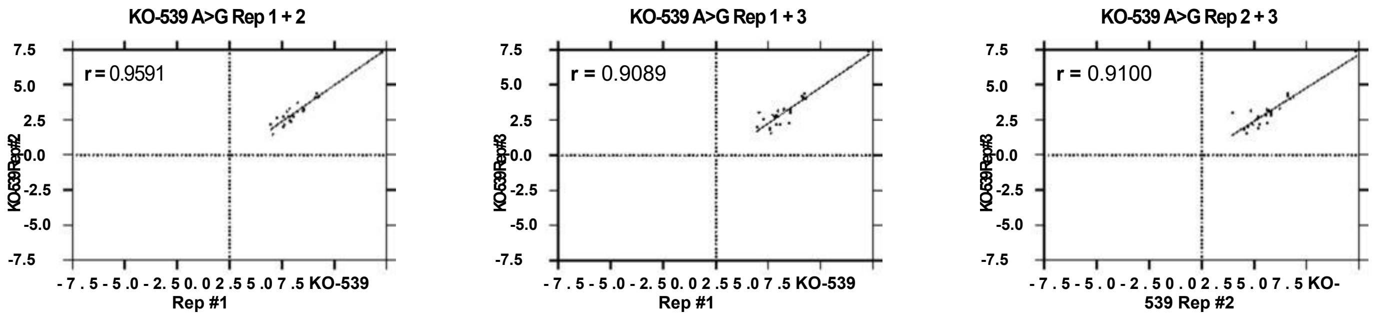

We additionally performed MAGeCK MLE analysis, treating each sgRNA as an independent "gene" to obtain guide-level statistics. For each guide, beta scores, z-scores, Wald P values, and FDR estimates are reported in Supplementary Dataset 2. A description of this analysis has been added to the Methods. We have added language to the text stating, "*Guide-level beta scores and associated statistics from MAGeCK MLE are reported in Supplementary Dataset 2.*"

Reviewer #4 (Remarks to the Author):

I have no further comments for the authors. They have rigorously addressed the issues that were raised.

Thank you for the re-review of our manuscript.

Reviewer #5 (Remarks to the Author):

I co-reviewed this manuscript with one of the reviewers who provided the listed reports. This is part of the Nature Communications initiative to facilitate training in peer review and to provide appropriate recognition for Early Career Researchers who co-review manuscripts.

Reviewer #3 (Remarks to the Author),

The authors have improved data reporting and transparency by the inclusion of Supp Fig 1F and 1G, demonstrating high replicate correlation in the base editing screens.
I thank the authors for the inclusion of Supplementary Dataset 2, which is an important statistical summary of the screen results. However, in its current form, there is no key in one tab that allows the reader to check the p-value for each amino acid hit/sgRNA. Please revise this table to make it more useful and avoid readers manually searching for each sgRNA between spreadsheets.
Given this new statistical summary, the authors should add a comment in the text to summarise how they selected the validation sgRNA set. We chose 40 sgRNAs (line 40) with L2FC>4. Please comment on how many of these were significant.
The authors have addressed my remaining concerns, and I wouldsupport publication of the revised article in Nature Communications.

We thank the reviewer for the comment and their thorough review of our manuscript and our screen. As suggested regarding Supplementary Dataset 2, we have now revised it such that each tab with the MAGeCK data (one tab for A>G screen, one tab for C>T screen) contains the predicted amino acid edit(s) in addition to the test statistics.

We introduced the criteria for guide selection previously as follows:

- "*To validate the results and quantify the effects of each guide that scored in the base editor screen, we identified 40 individual sgRNAs that enriched Log2FC ≥4.0 in either the A>G or C>T arms of the base editor screen.*"

We have now expanded this section, based on the reviewer feedback, to read:

- *"To validate the results and quantify the effects of each guide that scored in the base editor screen, we identified 40 individual sgRNAs that enriched Log2FC ≥4.0 in either the A>G or C>T arms of the base editor screen for at least one drug condition relative to DMSO control. Of note, despite strong correlation between technical replicates in the screen (Supplementary Figures 3A+B), only one positively selecting guide per drug condition reached statistical significance by MAGeCK (p-value <0.05 and FDR <0.05; Supplementary Data 2), highlighting the importance for single guide validation of enriching hits from the screen."*

Reviewer #2 (Remarks to the Author):

Manuscript by Bourgeois et al describes comprehensive analysis of mutations arising in MEN1 upon exposure to various menin inhibitors. This is an important and timely research. Authors addressed all my comments and significantly improved the manuscript. I have couple minor points to address:

Comment 1.
Finding that more potent menin inhibitor reduces rate of MEN1 mutations and the outgrowth of resistant leukemia clones is one of the most consequential conclusions from this manuscript. Authors strengthened this finding in the revised manuscript by including new in vivo data showing that increasing the dose of different menin inhibitor such as JNJ-6617 effectively suppresses the development of MEN1 mutations.
Additional new results examine the effect of combination of two menin inhibitors (JNJ-6617 and DSP-5336). Although these experiments are in cell culture, they strengthen the overall conclusions regarding emergence of MEN1 mutations. A limitation of this approach is that the combination was tested at doubled total inhibitor concentration (25 nM DSP-5336 plus 25 nM JNJ-6617) compared with single-agent concentrations (25 nM),

which likely contributes to the absence of resistant clones. This point should be acknowledged in the text. Minor point: concentration of DSP-5336 is missing in Supplementary Fig 4 legend:
"In these two independent experiments (A+B), 12.5 million MOLM13 cells were seeded per replicate, with 5 flasks per experiment treated with JNJ-6617 25 nM, 5 flasks per experiment treated with DSP-5336 25 nM, and 6 flasks per experiment treated with both JNJ-6617 25 nM and DSP-5336 nM."

We thank the reviewer for this thoughtful comment. We agree that testing 25 nM of each compound results in a total menin inhibitor exposure of 50 nM, which may contribute to the suppression of resistant clones observed in the combination arm. We have added the following statement to the manuscript: "*However, it should be noted that the suppression of resistant clones observed with 25 nM of each compound may reflect the higher total menin inhibitor exposure, 50 nM combined, rather than a true benefit from combining two menin inhibitors with different MEN1 mutation resistance profiles.*"

Comment 4.
The authors' response regarding the possibility that MEN1 mutations may be pre-existing is very relevant. It would be worth mentioning in the text that several MEN1 mutant clones were detected prior to day 28 of treatment, suggesting the possibility that these mutations were pre-existing.

We thank the reviewer for this important point. We agree that the detection of resistant clones as early as Day 21 suggests that *MEN1* mutations either arose shortly after initiation of menin inhibitor therapy or were present at very low levels prior to treatment. We have added a sentence to the manuscript reflecting this possibility. *"The early outgrowth of these MEN1 resistant clones suggests that the mutations either arose shortly after initiation of menin inhibitor therapy or were pre-existing at very low levels prior to treatment."*

Minor point: Figure 6 legend refers to Supplementary Fig 4C but there is no such figure, please correct that.

Thank you for identifying this error. The correct reference in our second revision was Supplementary Fig. 5E, and this had been corrected in the manuscript. In this third resubmission, this is now Supplementary Fig. 13C.

Final question to the authors – were any double mutations in MEN1 observed in these screens or in vivo?

This is an important question. In the initial clinical report (Perner F, Stein E et al., *Nature* 2023; PMID: 36922589), multiple *MEN1* mutations were often detected in individual patients. While some of these could theoretically represent compound mutations within the same clone, the distinct variant allele frequency dynamics observed in that study suggested that many mutations arose in separate clones.

In our PDX experiments, we occasionally detected more than one *MEN1* mutation within a single mouse (Supplementary Fig. 5C+D). However, in all such cases, we did not observe two distinct *MEN1* mutations each exhibiting a clear heterozygous peak by Sanger sequencing, arguing against their co-occurrence within the same cell population.

In the CRISPR base editor system, bystander edits within the editing window can generate multiple nucleotide substitutions from a single guide (Supplementary Fig. 2F). Nevertheless, the screen identified recurrent resistance residues also observed clinically (SNDX-5613), *in vivo* (SNDX-5613 and JNJ-6617), and *in vitro* (JNJ-6617 and DSP-5336).
